# Correction: Directed differentiation of human iPSCs to functional ovarian granulosa-like cells via transcription factor overexpression

**DOI:** 10.7554/eLife.87987

**Published:** 2023-03-22

**Authors:** Merrick D Pierson Smela, Christian C Kramme, Patrick RJ Fortuna, Jessica L Adams, Alina Rui Su, Edward Dong, Mutsumi Kobayashi, Garyk Brixi, Venkata Srikar Kavirayuni, Emma Tysinger, Richie E Kohman, Toshi Shioda, Pranam Chatterjee, George M Church

**Keywords:** Human, Mouse

Pierson Smela MD, Kramme CC, Fortuna PRJ, Adams JL, Su R, Dong E, Kobayashi M, Brixi G, Kavirayuni VS, Tysinger E, Kohman RE, Shioda T, Chatterjee P, Church GM. 2023. Directed differentiation of human iPSCs to functional ovarian granulosa-like cells via transcription factor overexpression. *eLife* **12**:e83291. doi: 10.7554/eLife.83291.

Published 21 February 2023

In the original version of this paper, the images shown as “Mouse Day 32” in Figure 5 were duplicates of the “Human Day 32” images. The correct images were present in the manuscript sent out for peer review, but they were inadvertently swapped during editing for publication. We have now remade Figure 5 with the correct images.

Additionally, in the corrected version of this paper, we have added the Parse scRNA-seq kit to the Key Resources Table. This was previously missing.

The corrected Figure 5 is shown here:

**Figure 1:**
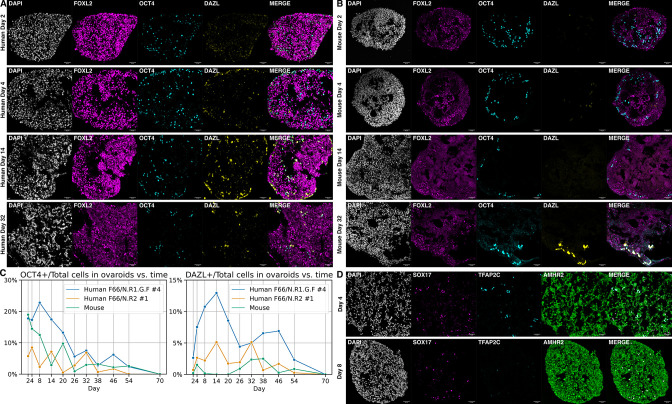


The originally published Figure 5 is shown for reference:

**Figure 2:**
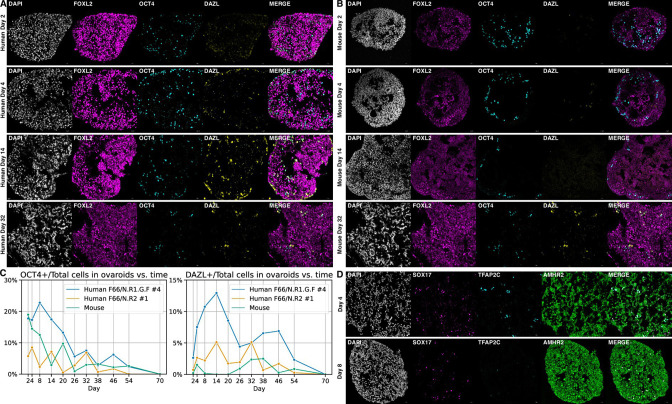


The article has been corrected accordingly.

