# Supplementary figures and images for: Directed differentiation of human iPSCs to functional ovarian granulosa-like cells via transcription factor overexpression (part 1 of 3)
Source: eLife. 2023 Feb 21;12:e83291. doi: 10.7554/eLife.83291 (PMC9943069; doi:10.7554/eLife.83291)

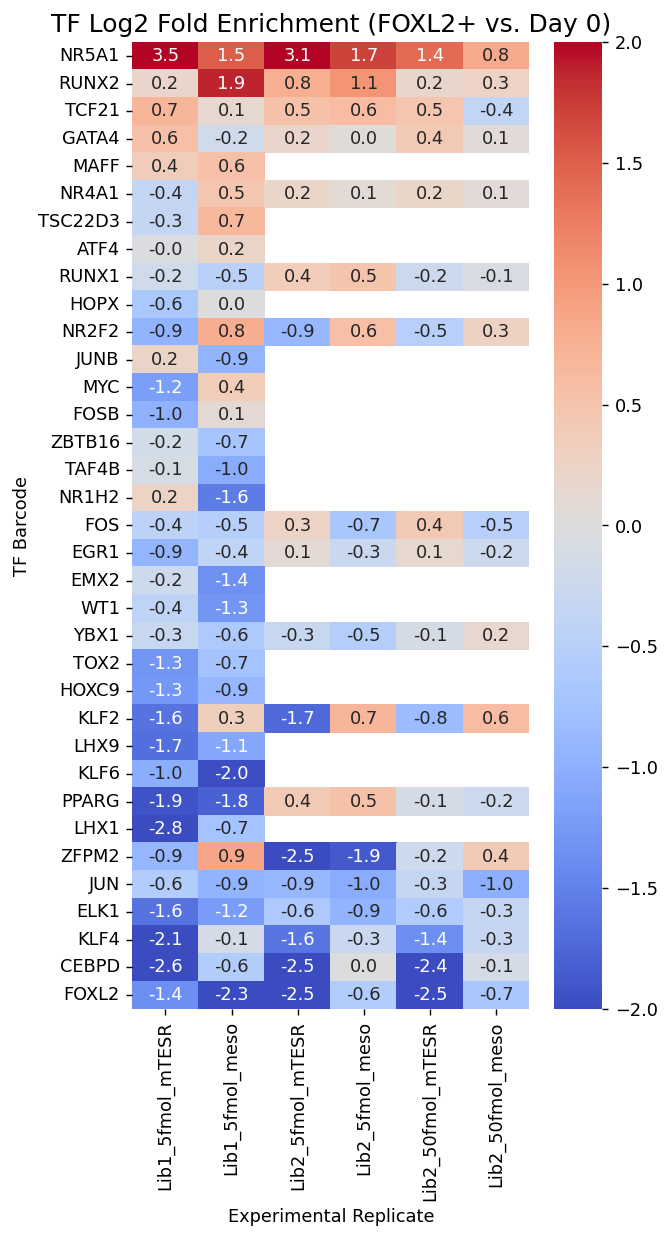

Supplement: Figure 2—source data 1. [file elife-83291-fig2-data1.zip › Figure2_sourcedata/Figure2A_heatmap.png]

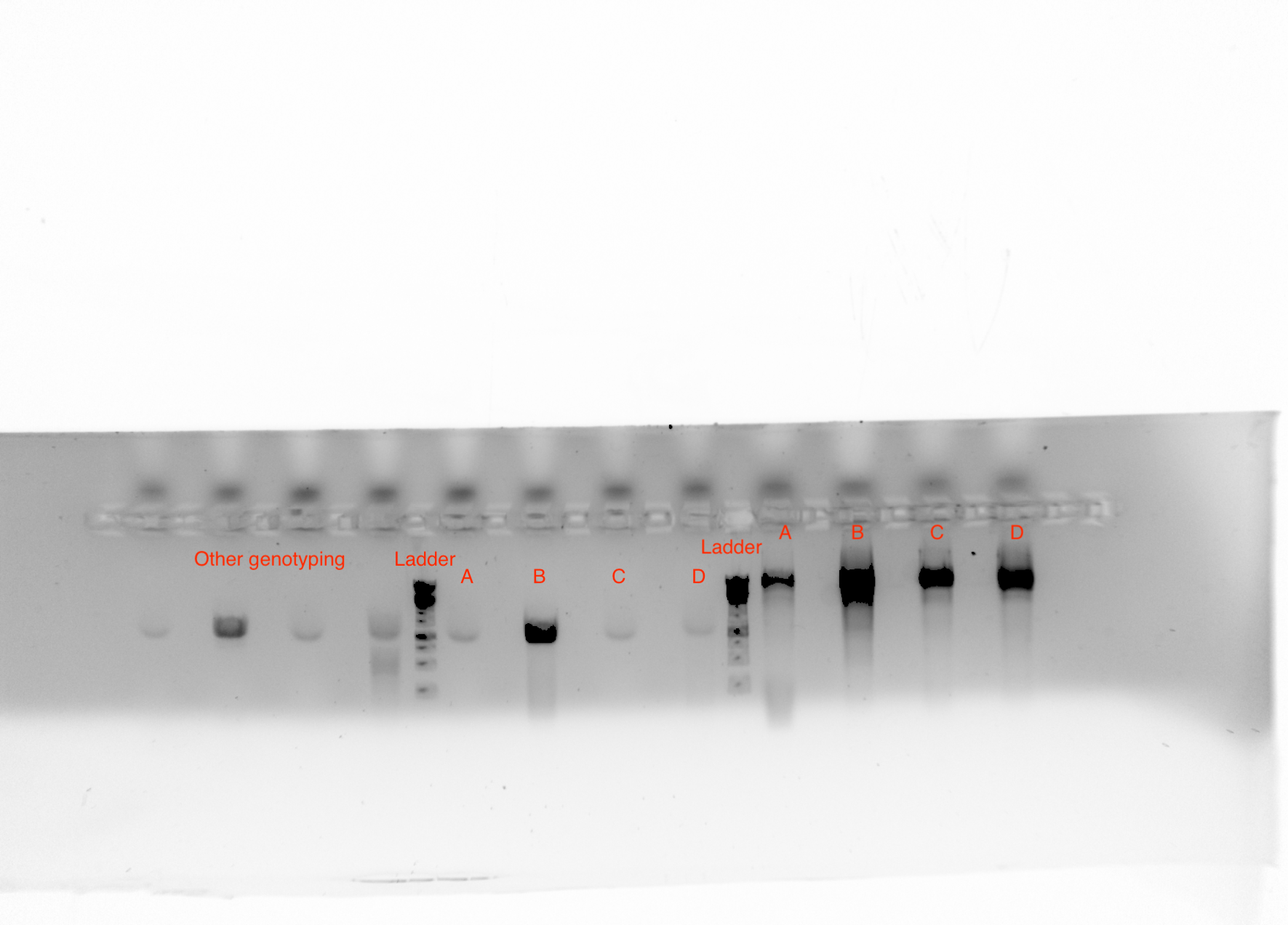

Supplement: Figure 2—figure supplement 1—source data 1. [file elife-83291-fig2-figsupp1-data1.zip › FigS1_source_data/2021-01-23_08h42m34s_127-106 127-128 annotated.tif]

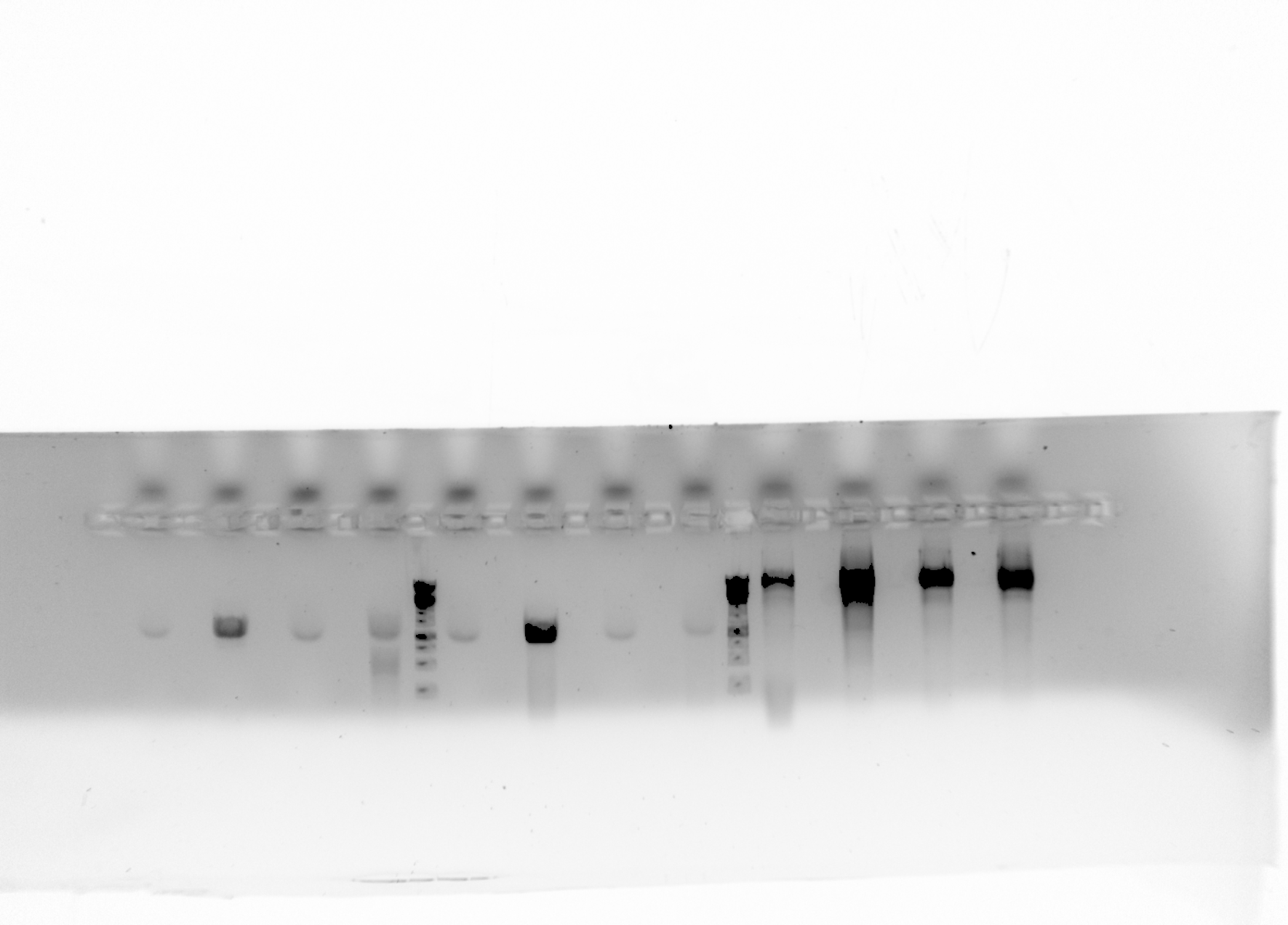

Supplement: Figure 2—figure supplement 1—source data 1. [file elife-83291-fig2-figsupp1-data1.zip › FigS1_source_data/2021-01-23_08h42m34s_127-106 127-128.tif]

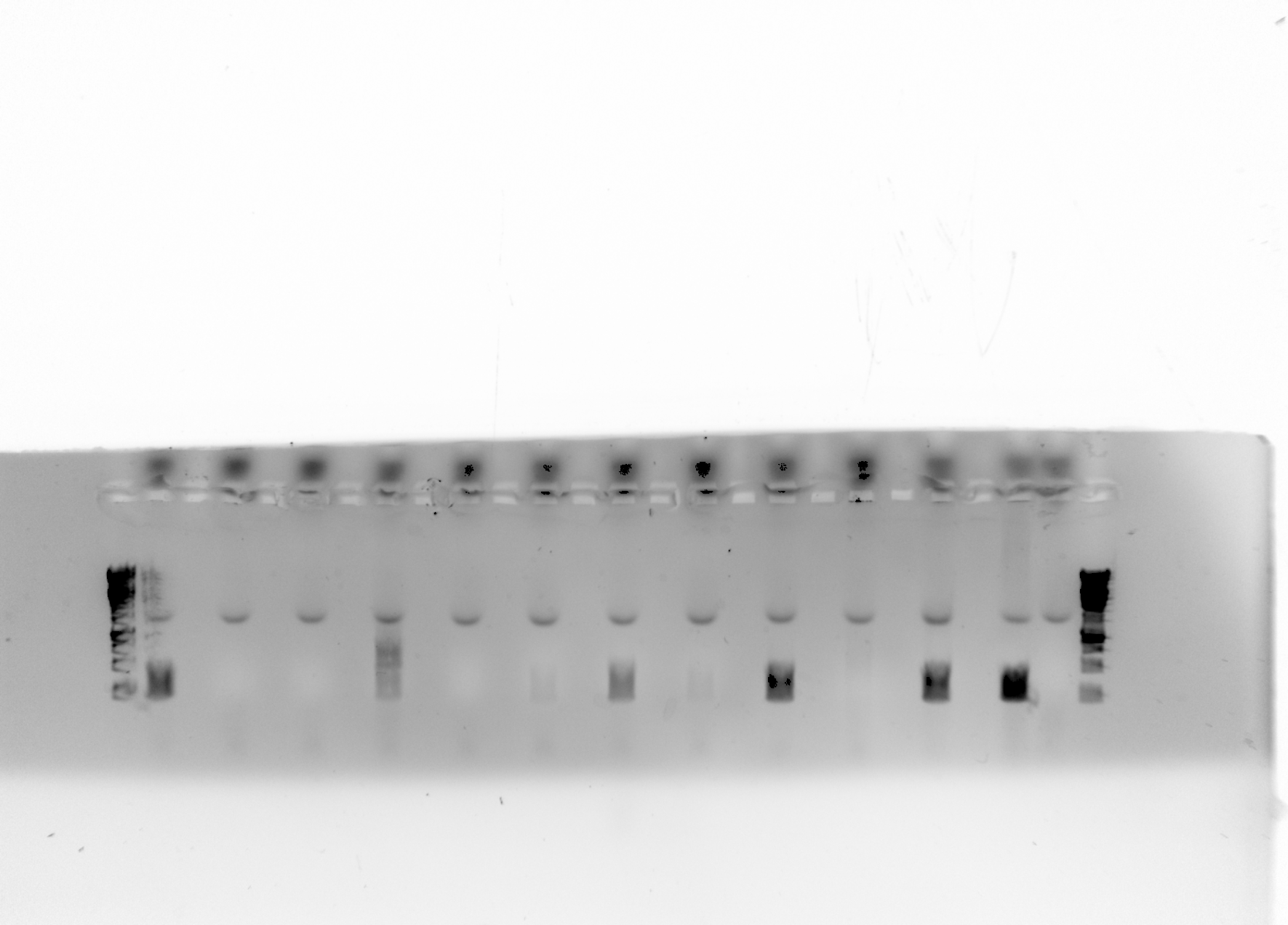

Supplement: Figure 2—figure supplement 1—source data 1. [file elife-83291-fig2-figsupp1-data1.zip › FigS1_source_data/FOXL2_redo_176-177 2021-01-14_12h38m00s.tif]

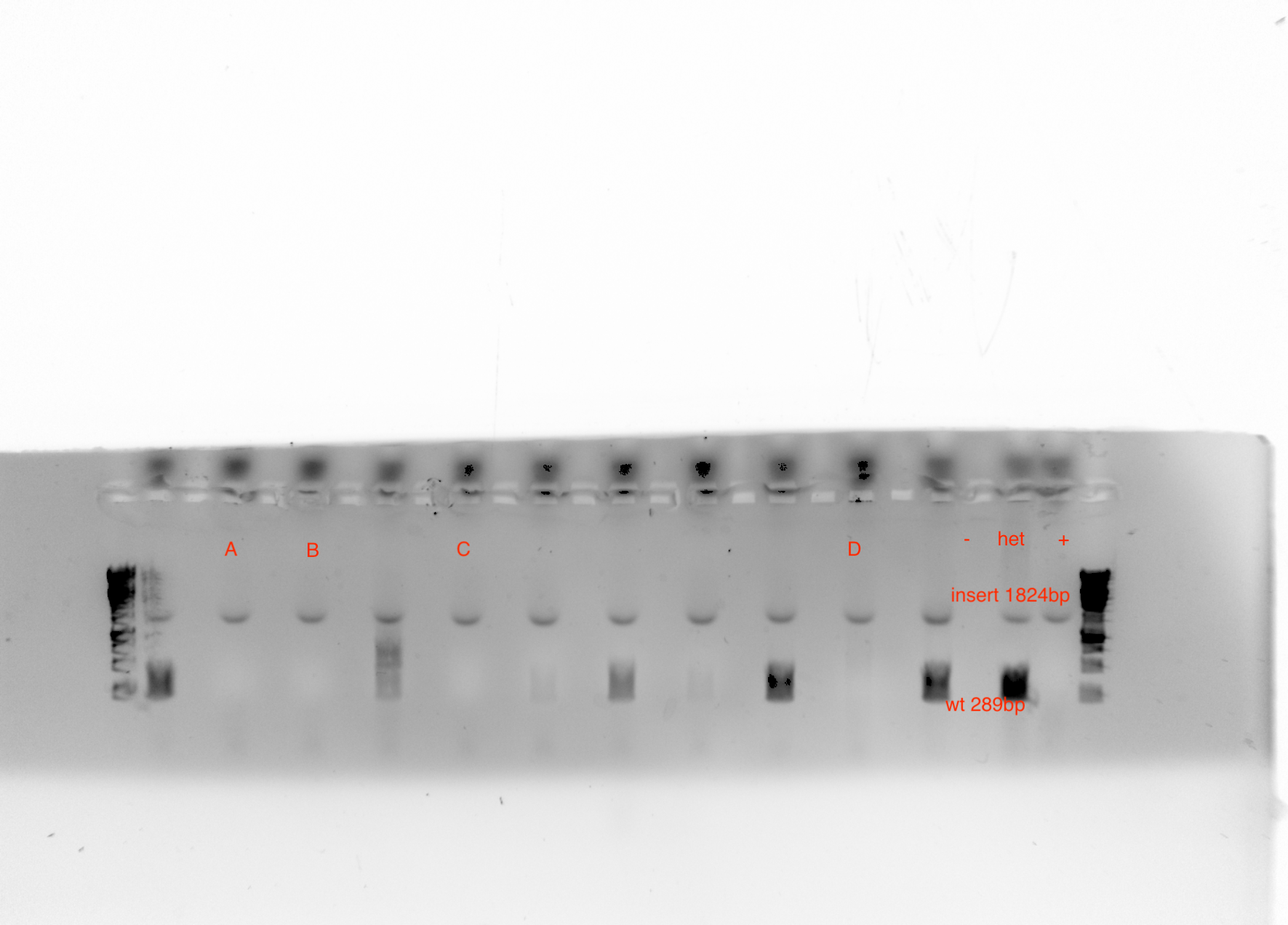

Supplement: Figure 2—figure supplement 1—source data 1. [file elife-83291-fig2-figsupp1-data1.zip › FigS1_source_data/FOXL2_redo_176-177 2021-01-14_12h38m00s annotated.tif]

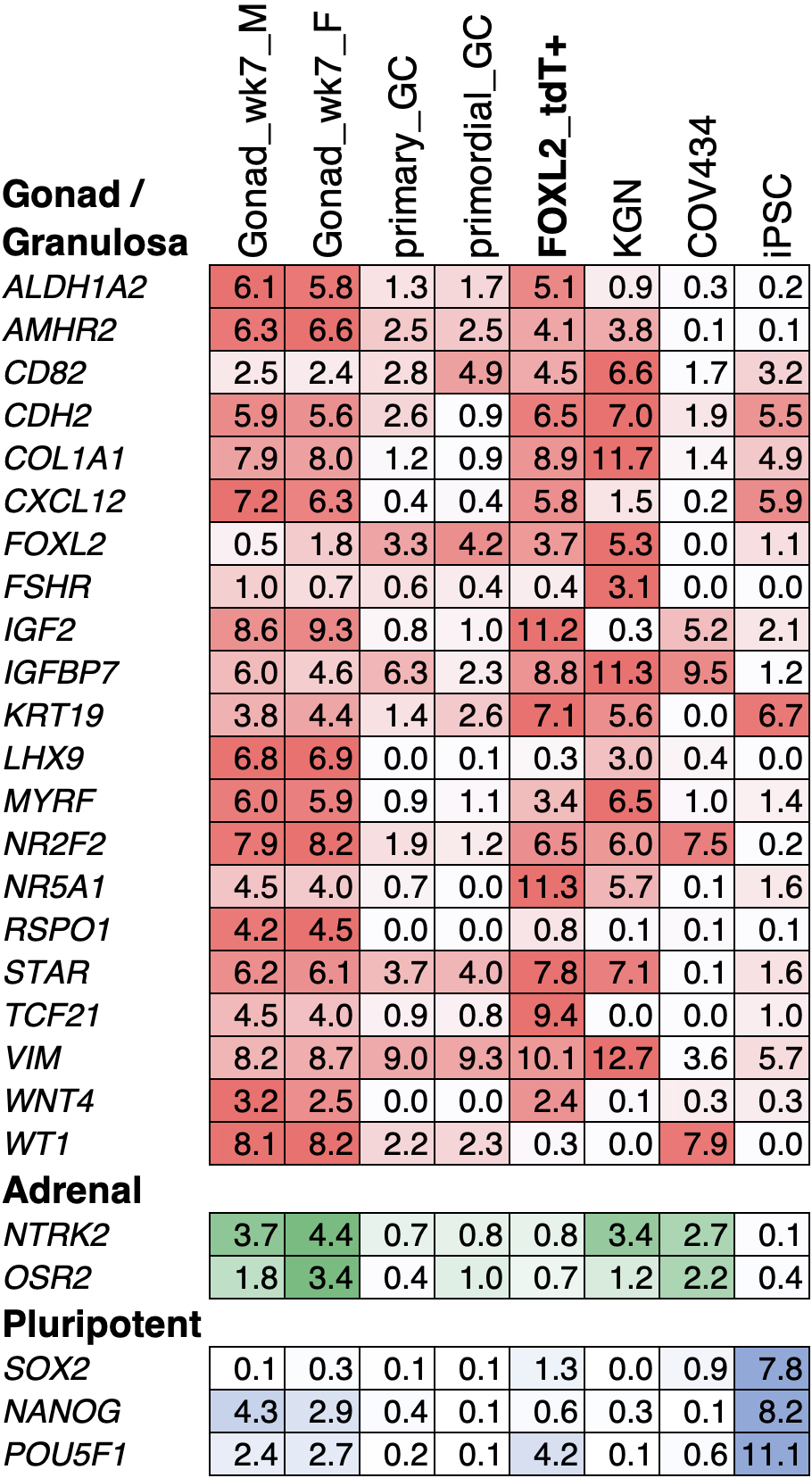

Supplement: Figure 3—source data 1. [file elife-83291-fig3-data1.zip › Figure3_sourcedata/Fig3A_TPM_merge/TPM_markers_2022-10-02.png]

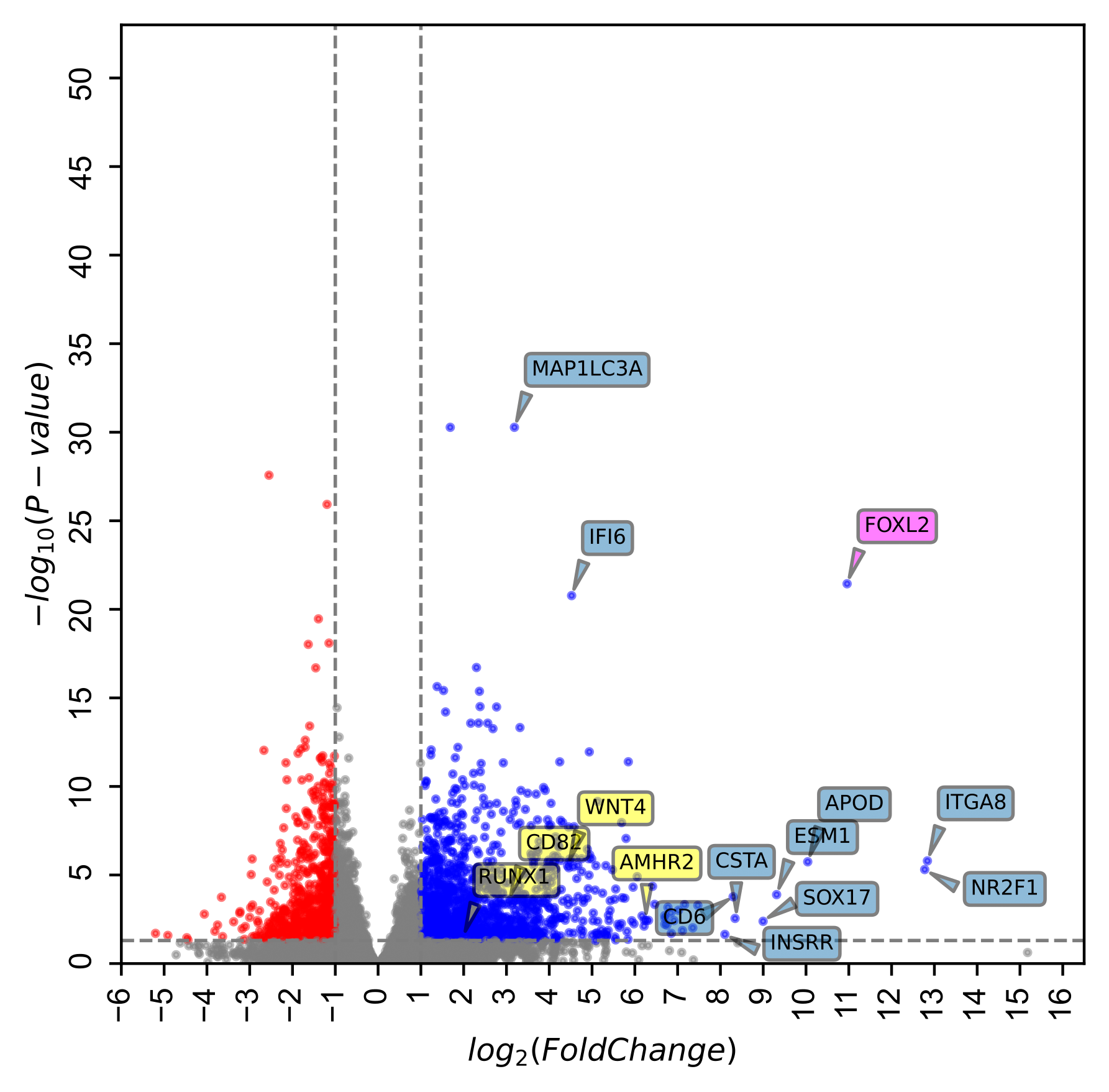

Supplement: Figure 3—source data 1. [file elife-83291-fig3-data1.zip › Figure3_sourcedata/Volcano_plots/volcano_FOXL2.png]

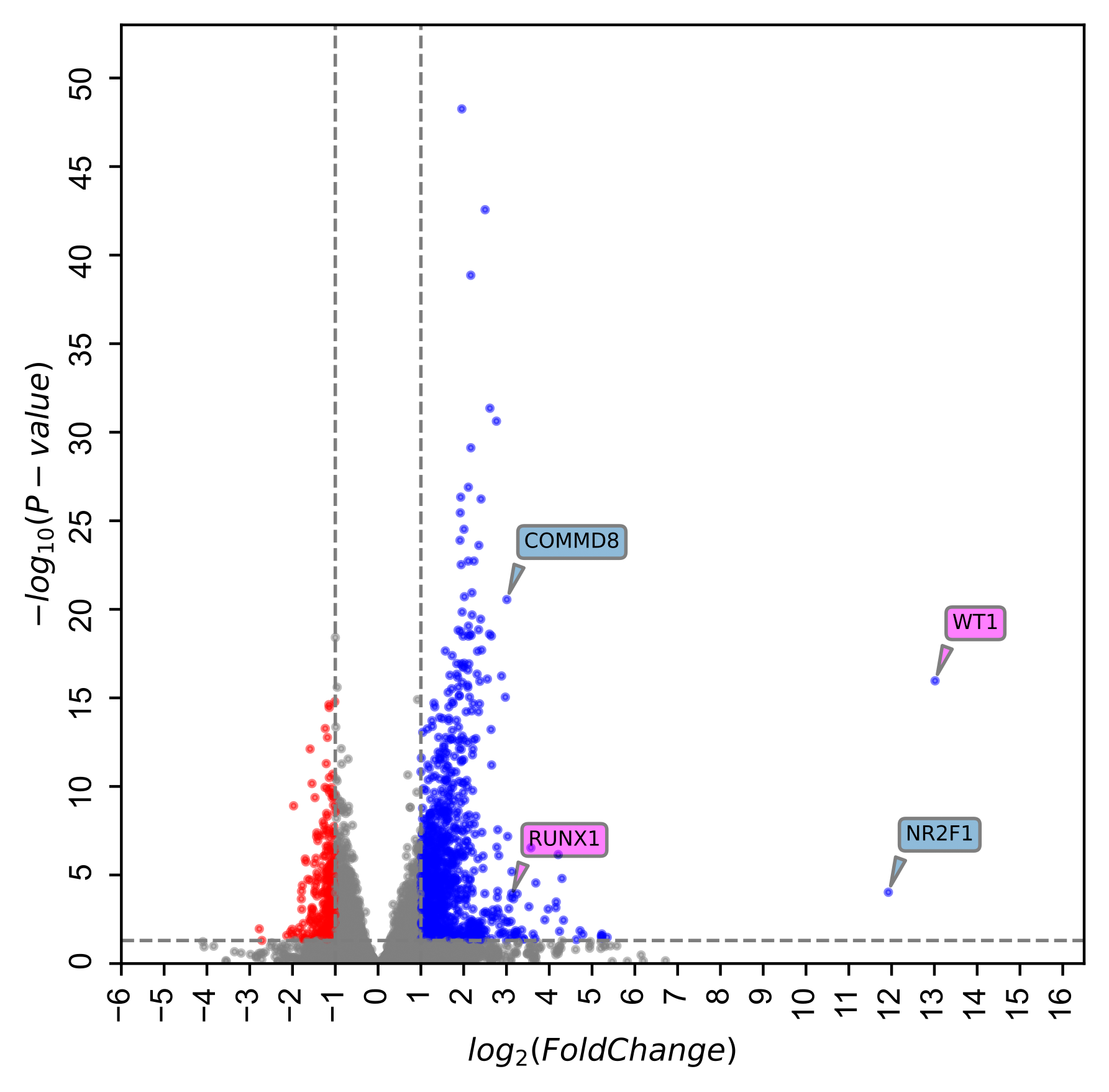

Supplement: Figure 3—source data 1. [file elife-83291-fig3-data1.zip › Figure3_sourcedata/Volcano_plots/volcano_WT1.png]

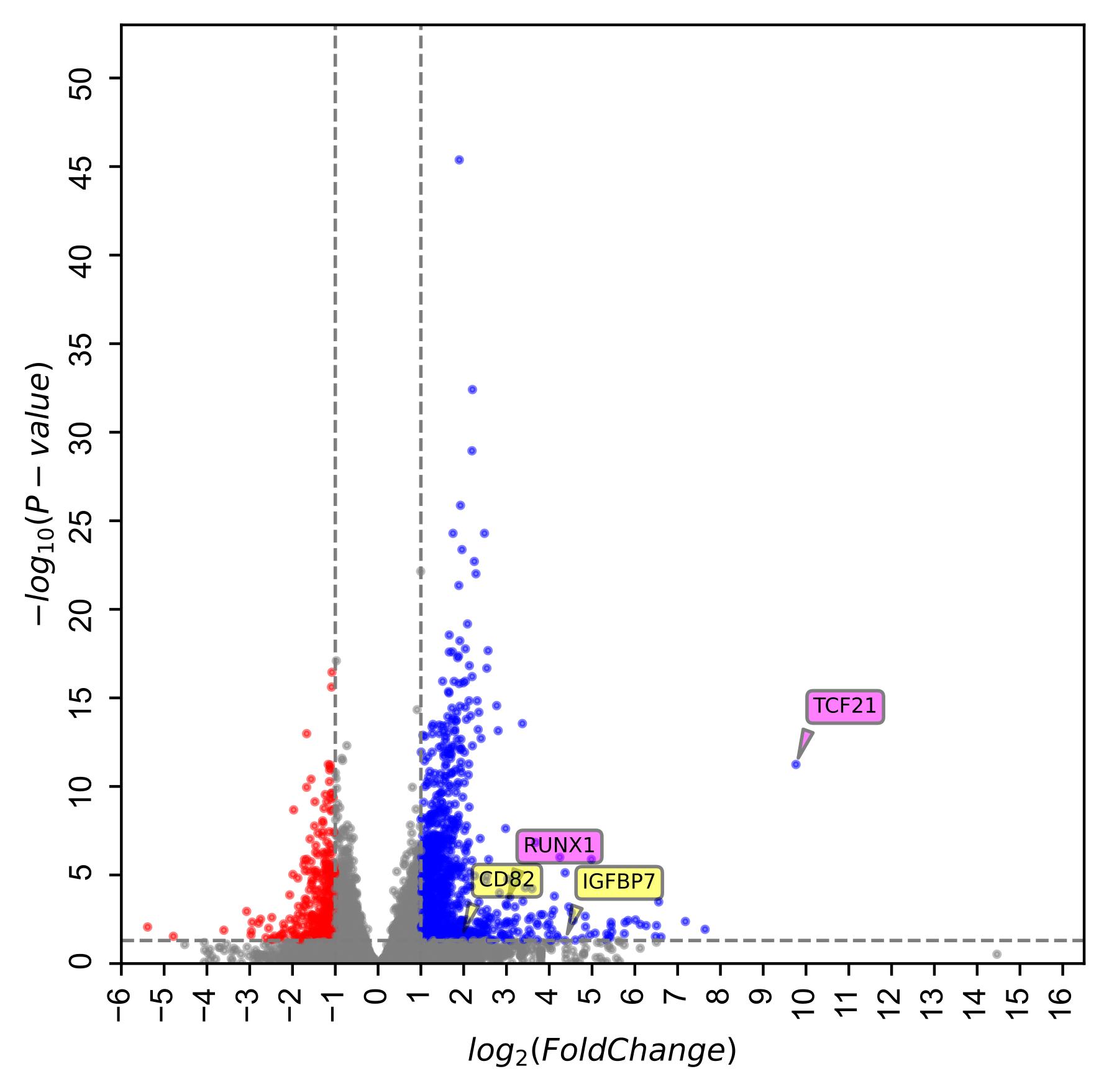

Supplement: Figure 3—source data 1. [file elife-83291-fig3-data1.zip › Figure3_sourcedata/Volcano_plots/volcano_TCF21.png]

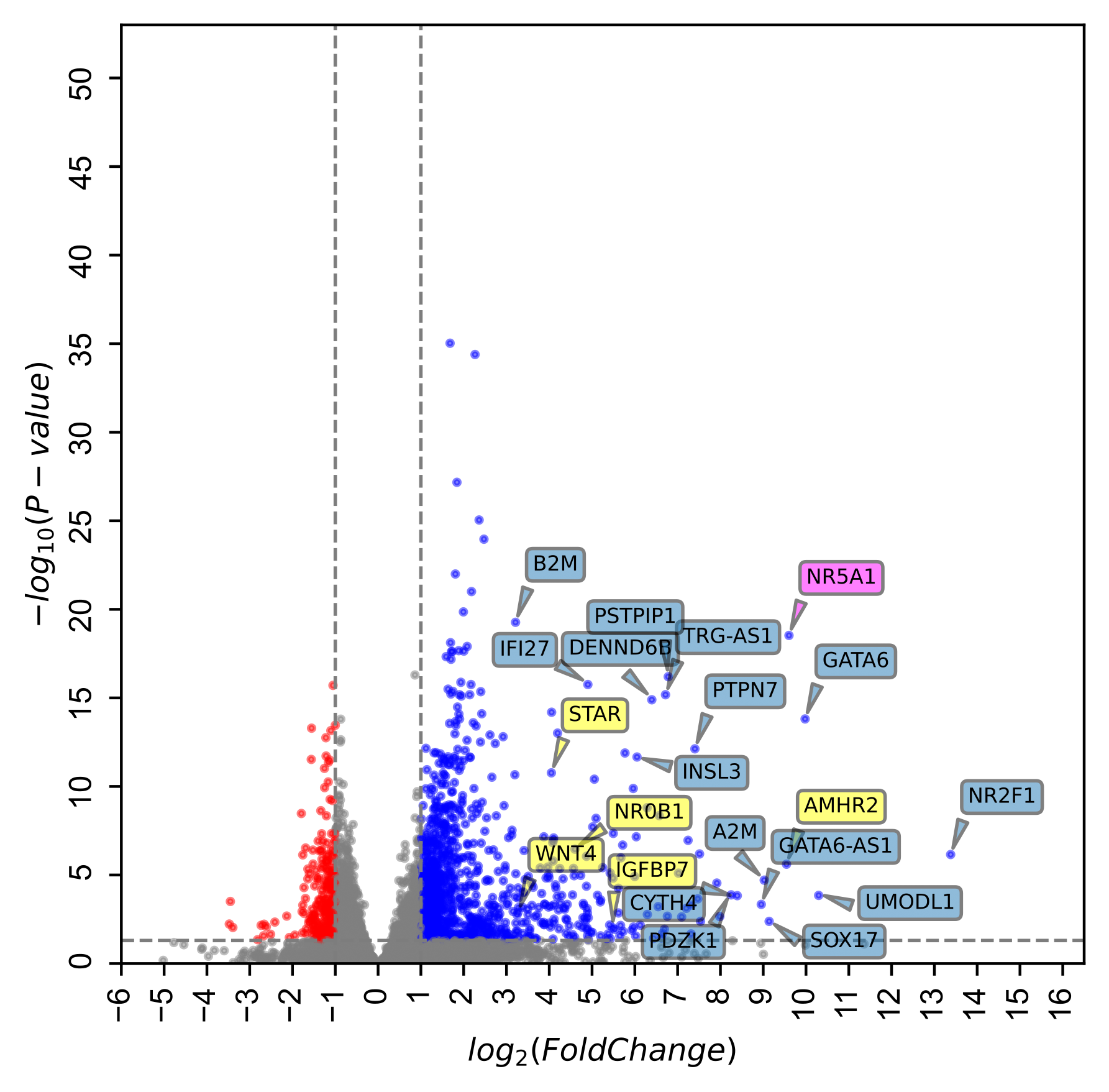

Supplement: Figure 3—source data 1. [file elife-83291-fig3-data1.zip › Figure3_sourcedata/Volcano_plots/volcano_NR5A1.png]

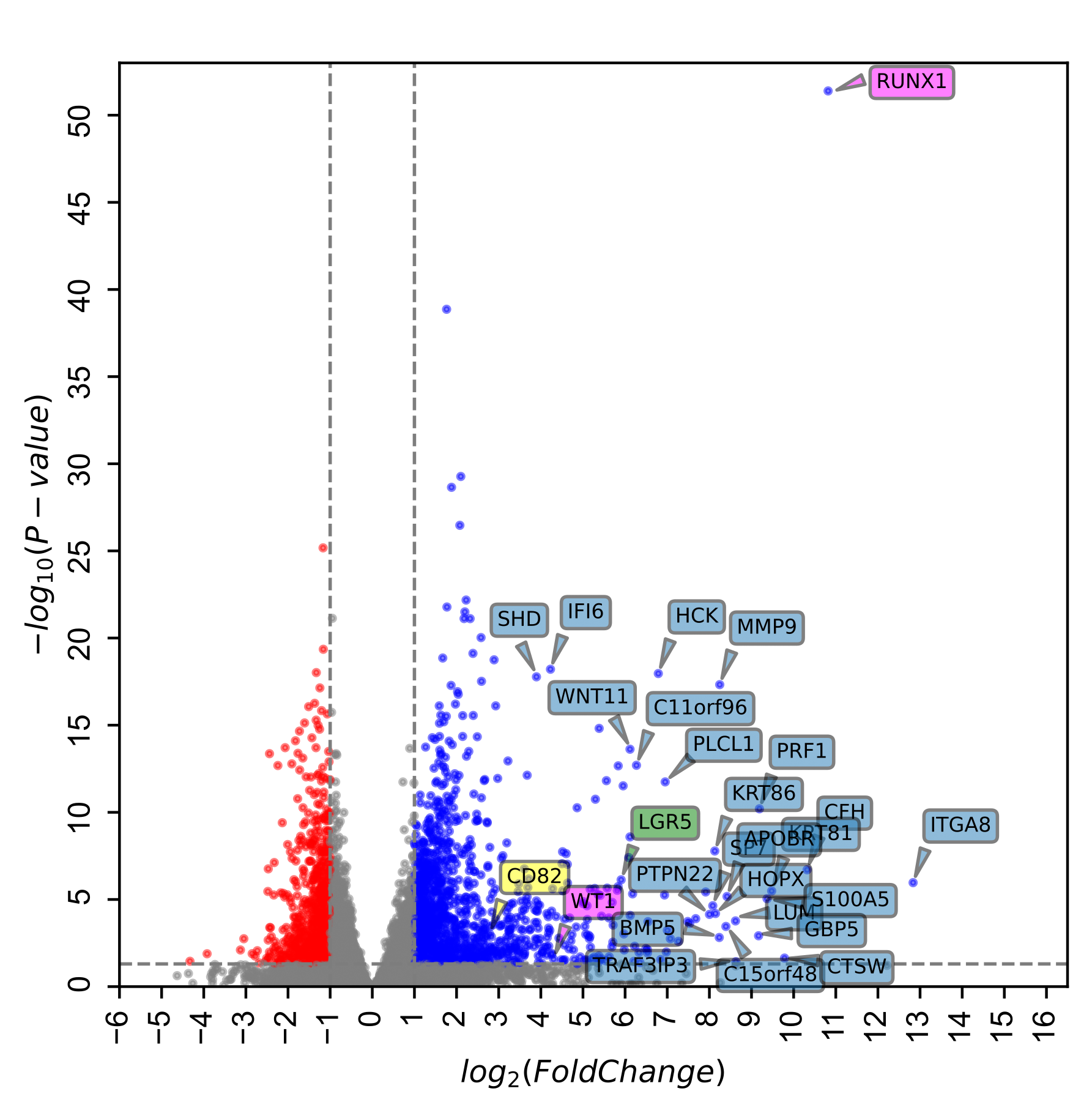

Supplement: Figure 3—source data 1. [file elife-83291-fig3-data1.zip › Figure3_sourcedata/Volcano_plots/volcano_RUNX1.png]

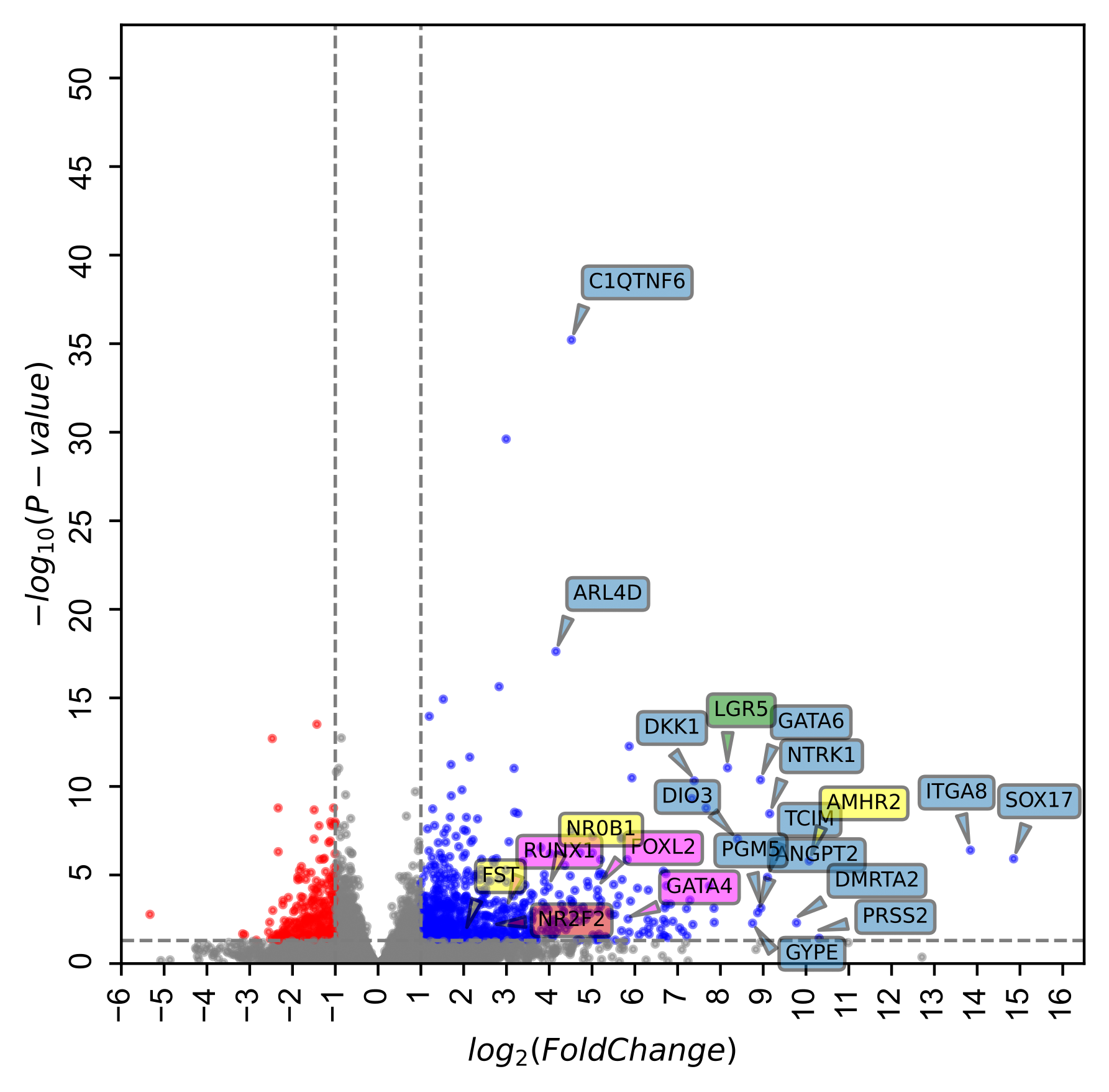

Supplement: Figure 3—source data 1. [file elife-83291-fig3-data1.zip › Figure3_sourcedata/Volcano_plots/volcano_GATA4.png]

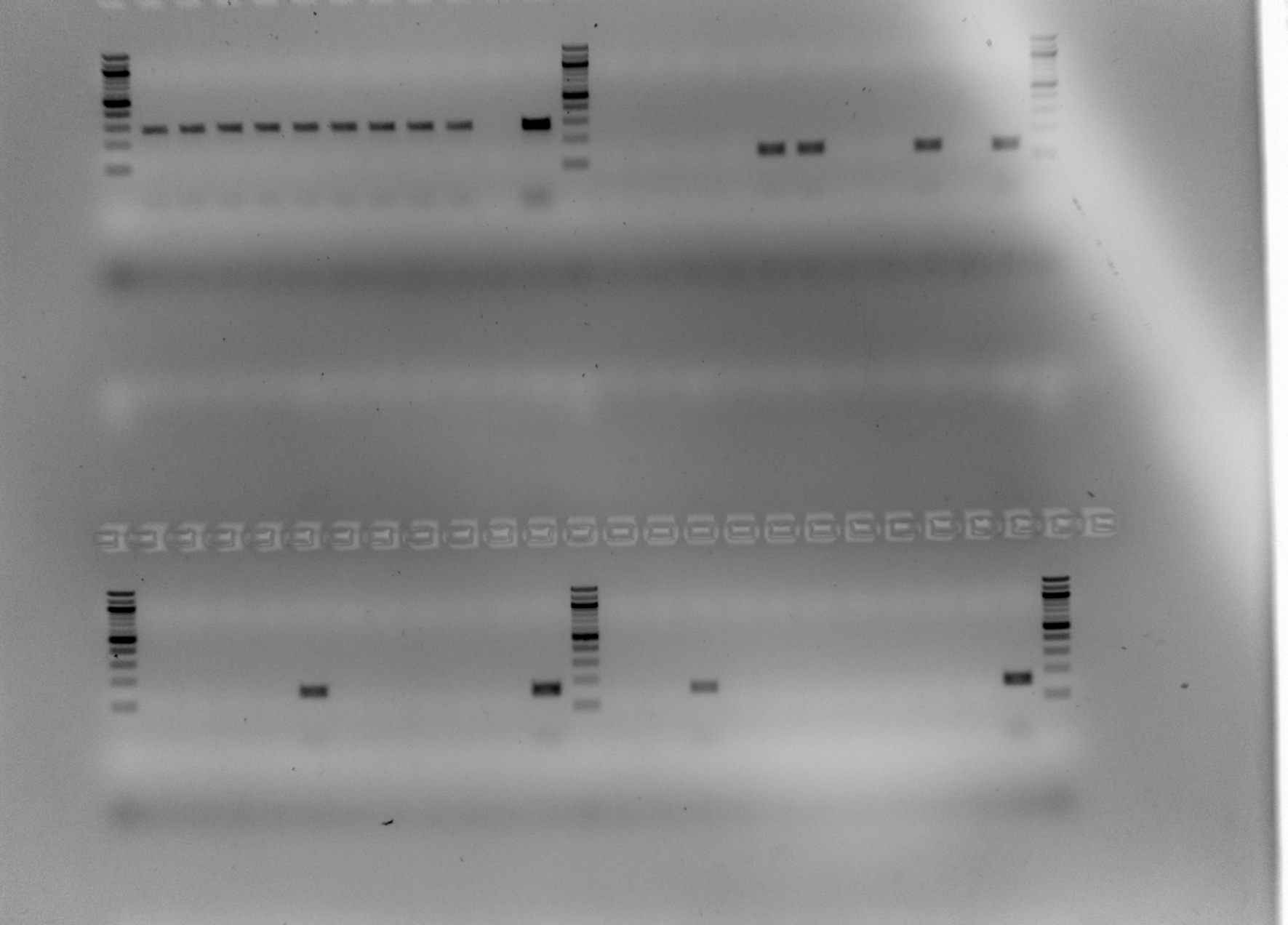

Supplement: Figure 3—figure supplement 1—source data 1. [file elife-83291-fig3-figsupp1-data1.zip › FigureS3_sourcedata/S3C_TF_genotyping_gels_raw/2022-12-09_09h40m11s_TFgeno_NR5A1_GATA4_FOXL2_TCF21.png]

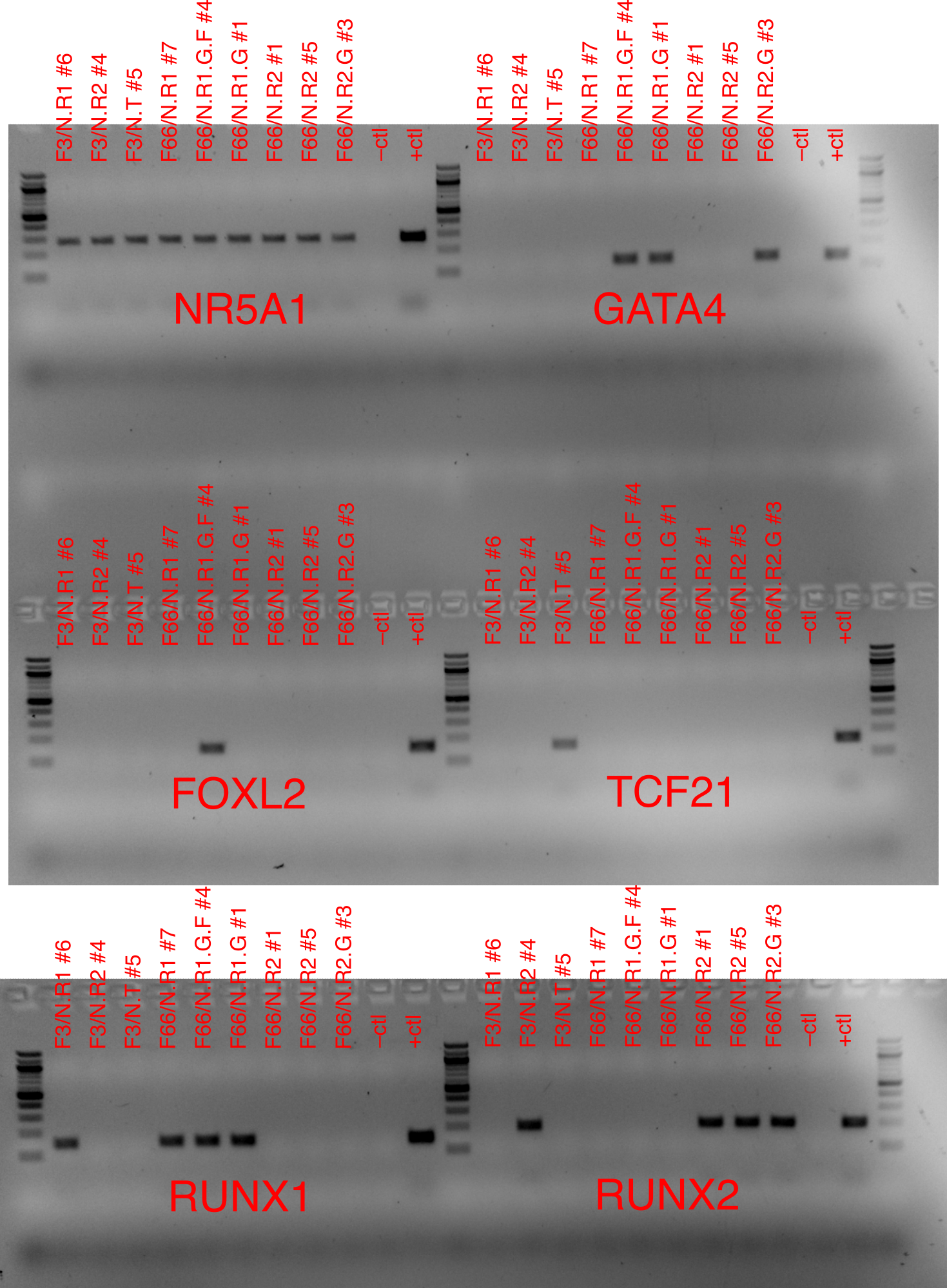

Supplement: Figure 3—figure supplement 1—source data 1. [file elife-83291-fig3-figsupp1-data1.zip › FigureS3_sourcedata/S3C_TF_genotyping_gels_raw/TF_Genotyping_Gel_Annotated.png]

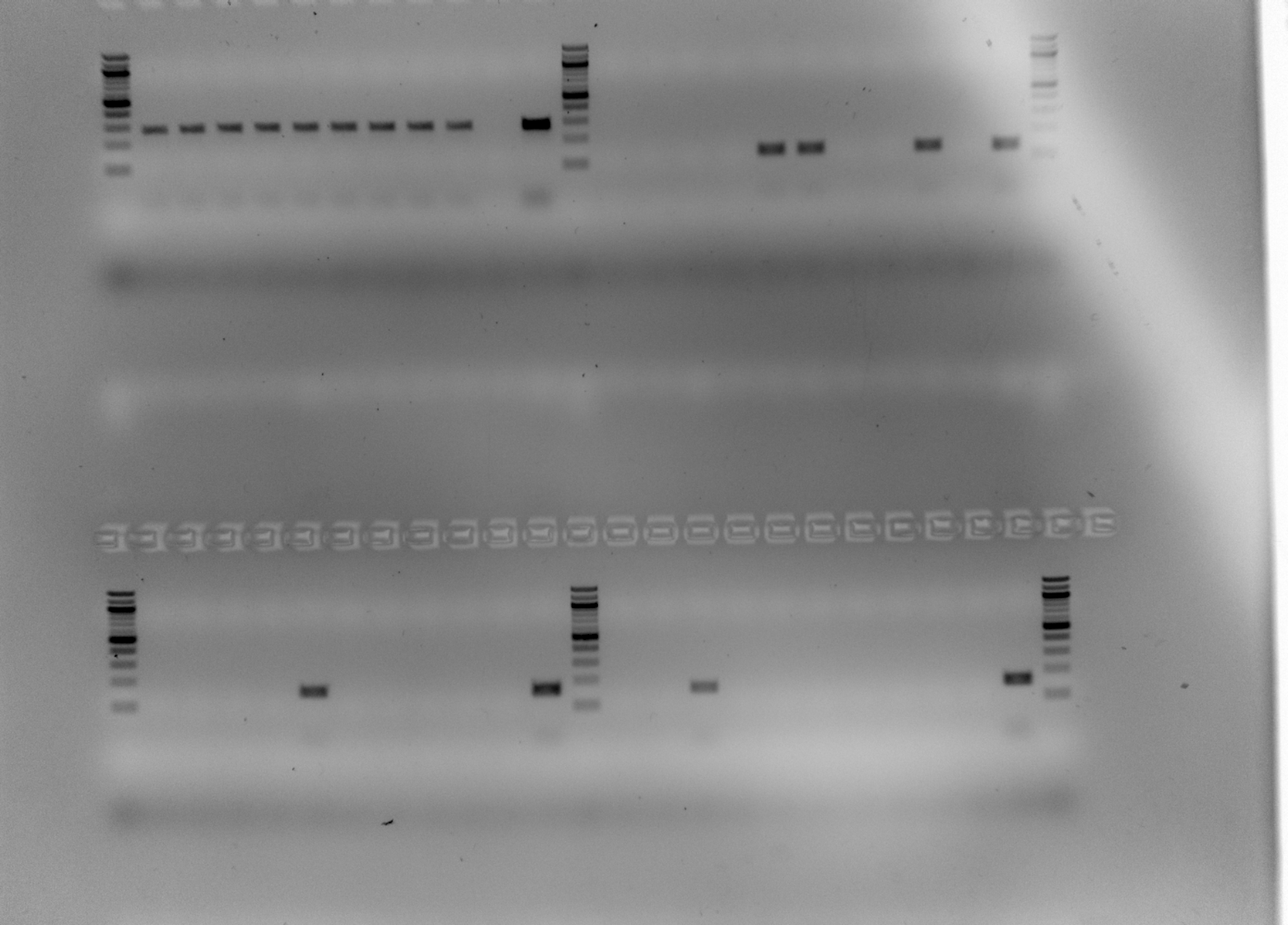

Supplement: Figure 3—figure supplement 1—source data 1. [file elife-83291-fig3-figsupp1-data1.zip › FigureS3_sourcedata/S3C_TF_genotyping_gels_raw/2022-12-09_09h40m11s_TFgeno_NR5A1_GATA4_FOXL2_TCF21.tif]

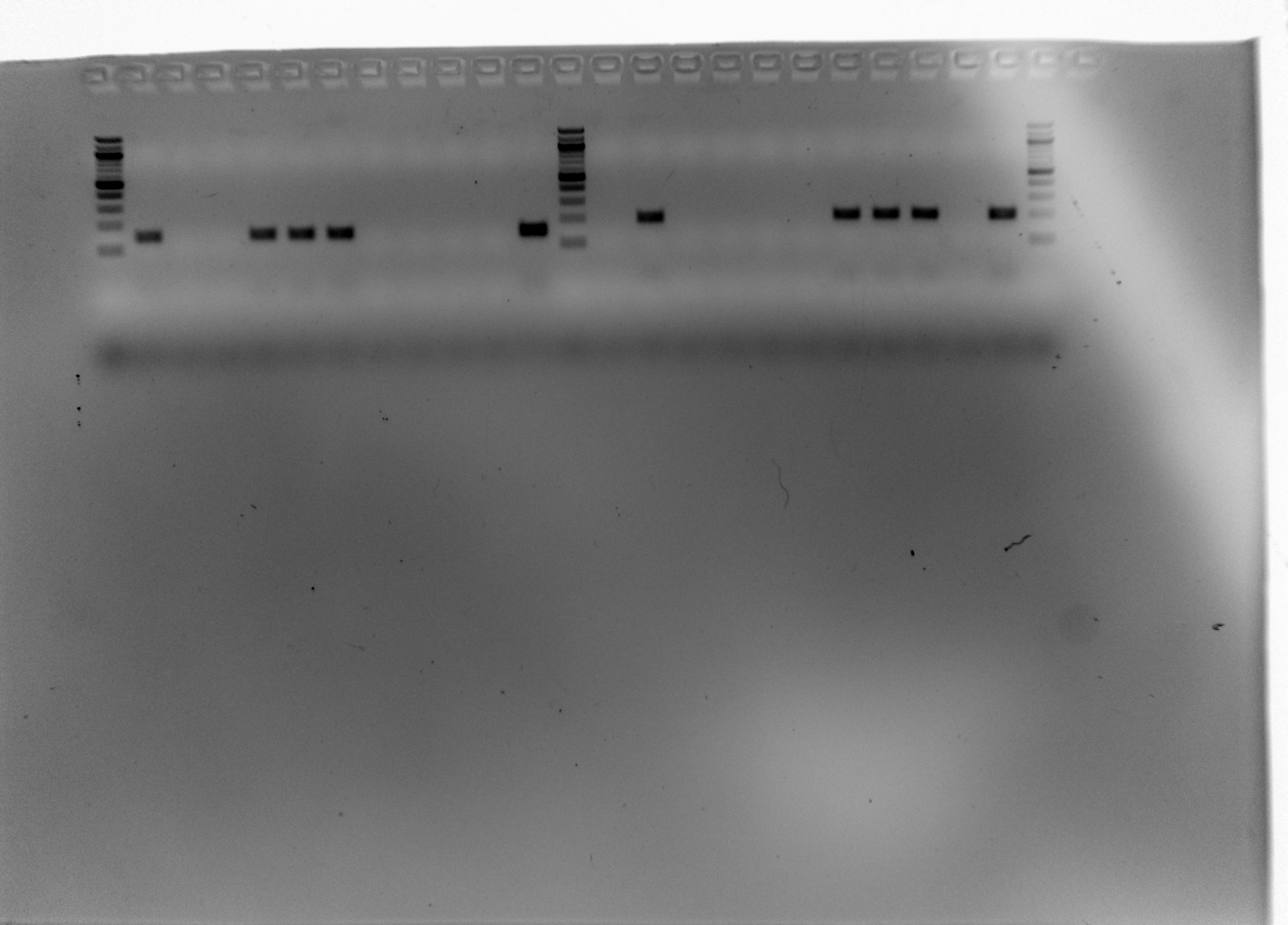

Supplement: Figure 3—figure supplement 1—source data 1. [file elife-83291-fig3-figsupp1-data1.zip › FigureS3_sourcedata/S3C_TF_genotyping_gels_raw/2022-12-09_09h36m43s_TFgeno_RUNX1_RUNX2.png]

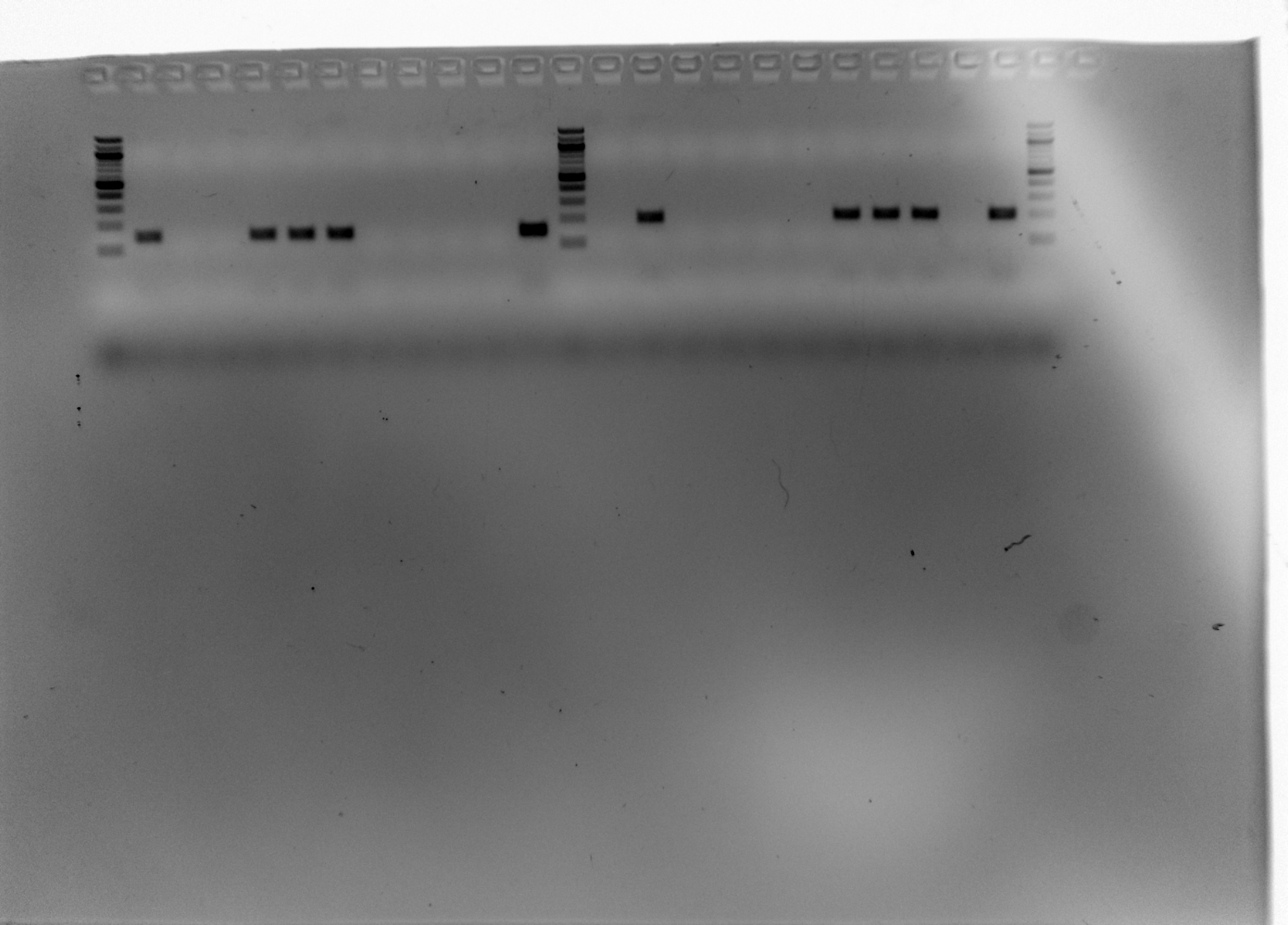

Supplement: Figure 3—figure supplement 1—source data 1. [file elife-83291-fig3-figsupp1-data1.zip › FigureS3_sourcedata/S3C_TF_genotyping_gels_raw/2022-12-09_09h36m43s_TFgeno_RUNX1_RUNX2.tif]

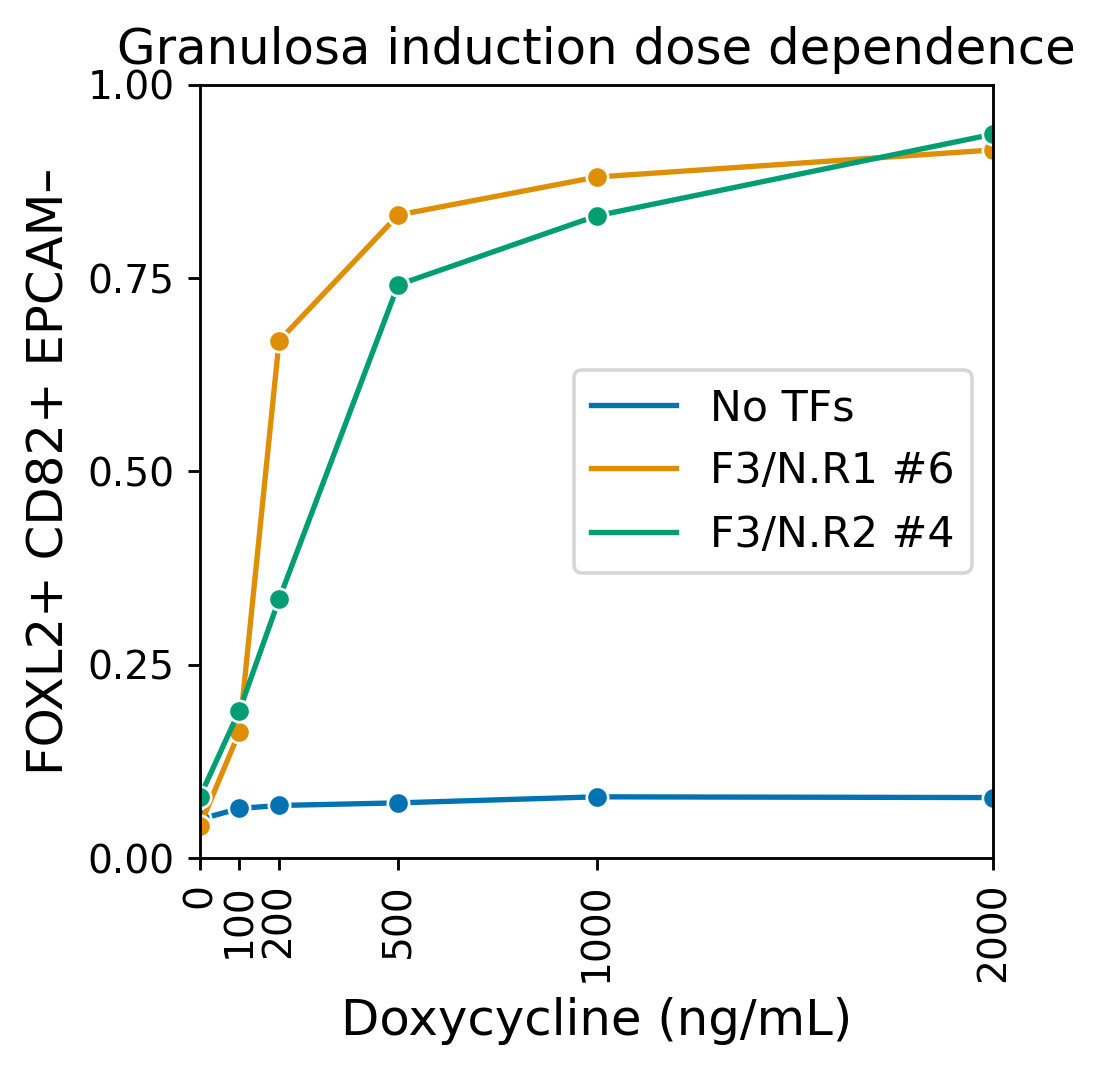

Supplement: Figure 3—figure supplement 2—source data 1. [file elife-83291-fig3-figsupp2-data1.zip › FigureS4_sourcedata/dose-dependence.png]

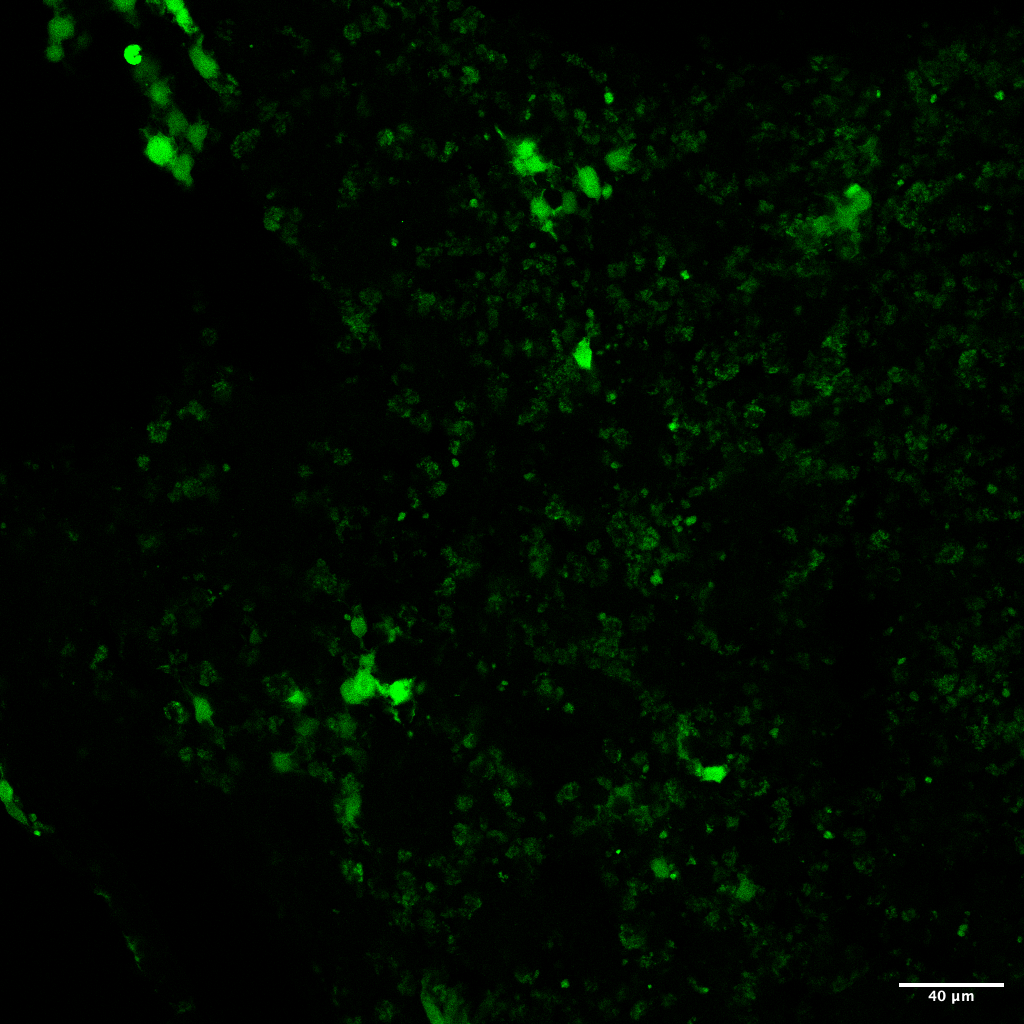

Supplement: Figure 5—source data 2. [file elife-83291-fig5-data2.zip › Figure5_sourcedata_timecourse_F66NR1GF#4/2022-08-25-human_day32_1_DAZL.png]

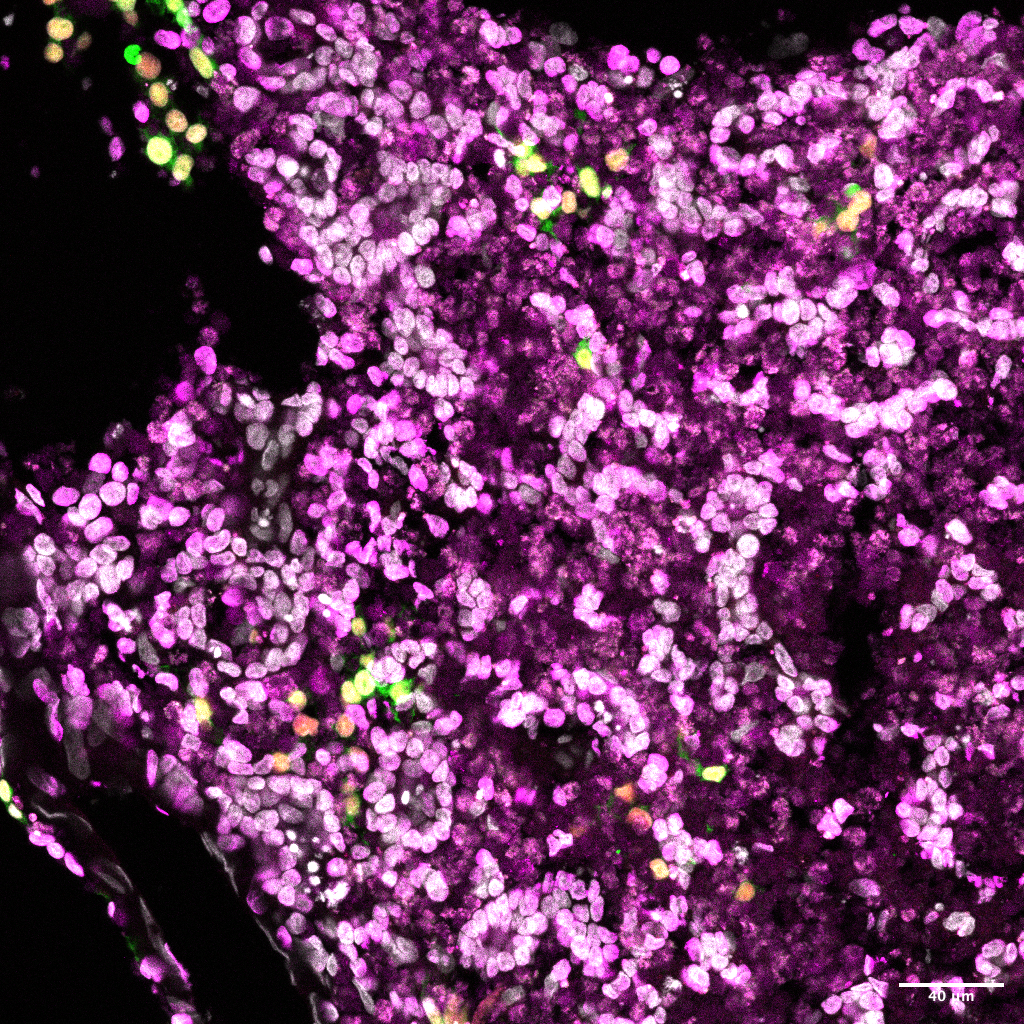

Supplement: Figure 5—source data 2. [file elife-83291-fig5-data2.zip › Figure5_sourcedata_timecourse_F66NR1GF#4/2022-08-25-human_day32_1_composite.png]

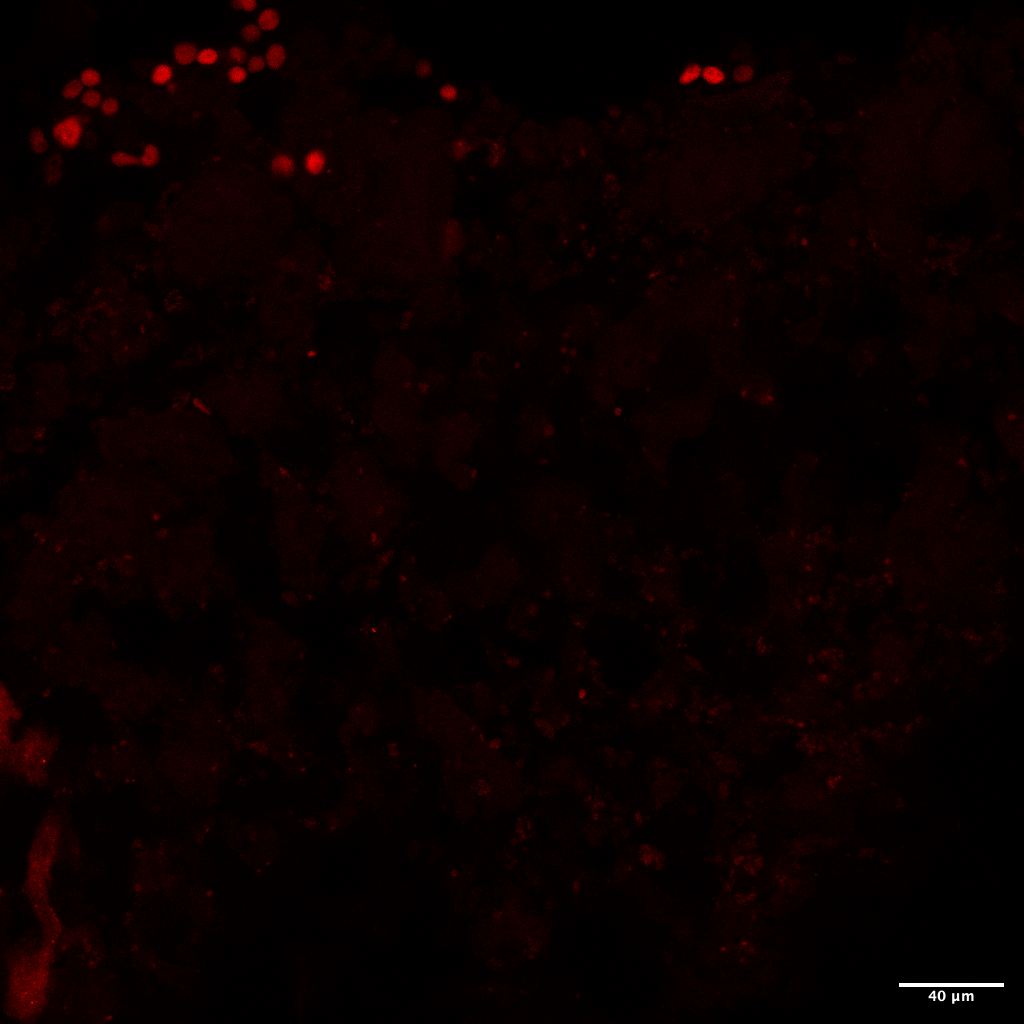

Supplement: Figure 5—source data 2. [file elife-83291-fig5-data2.zip › Figure5_sourcedata_timecourse_F66NR1GF#4/2022-08-24-human_day26_2_OCT4.png]

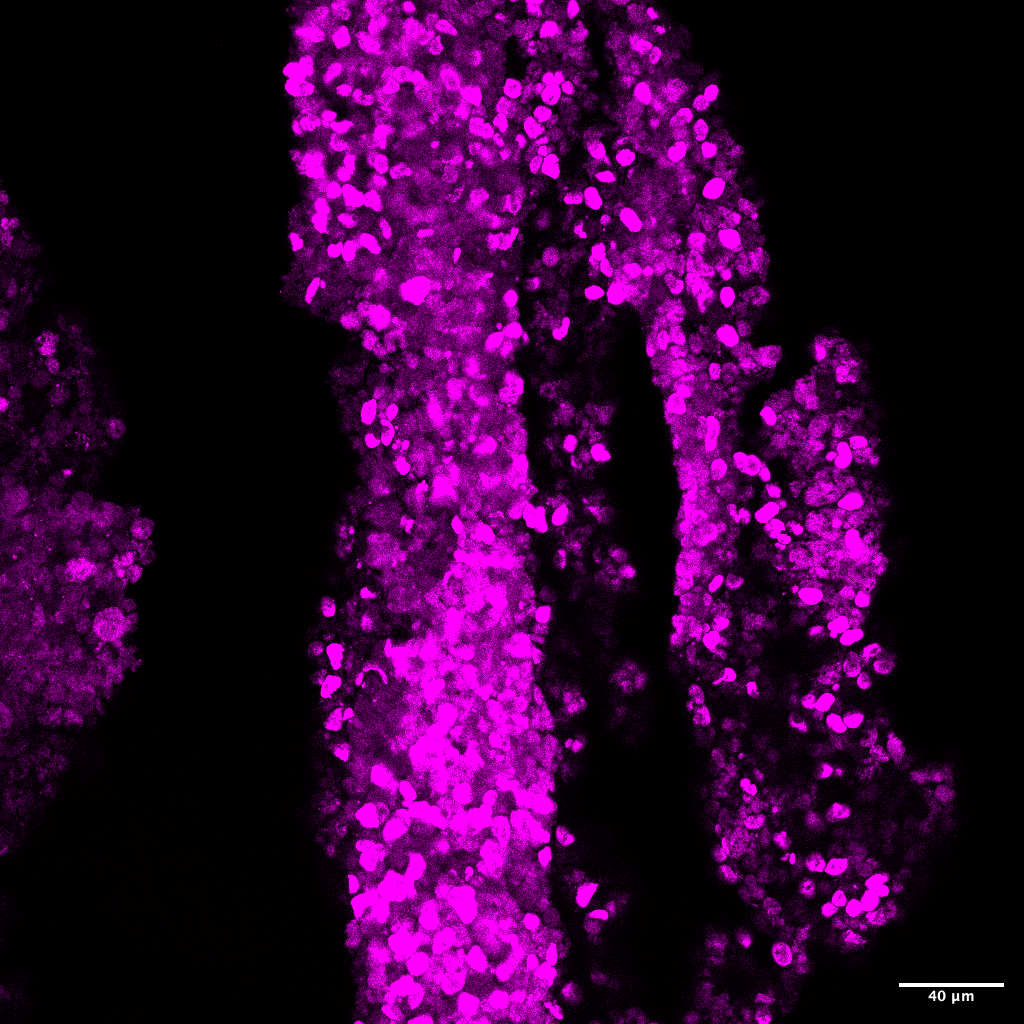

Supplement: Figure 5—source data 2. [file elife-83291-fig5-data2.zip › Figure5_sourcedata_timecourse_F66NR1GF#4/2022-08-23_day8_stainA.lif - FOXL2.png]

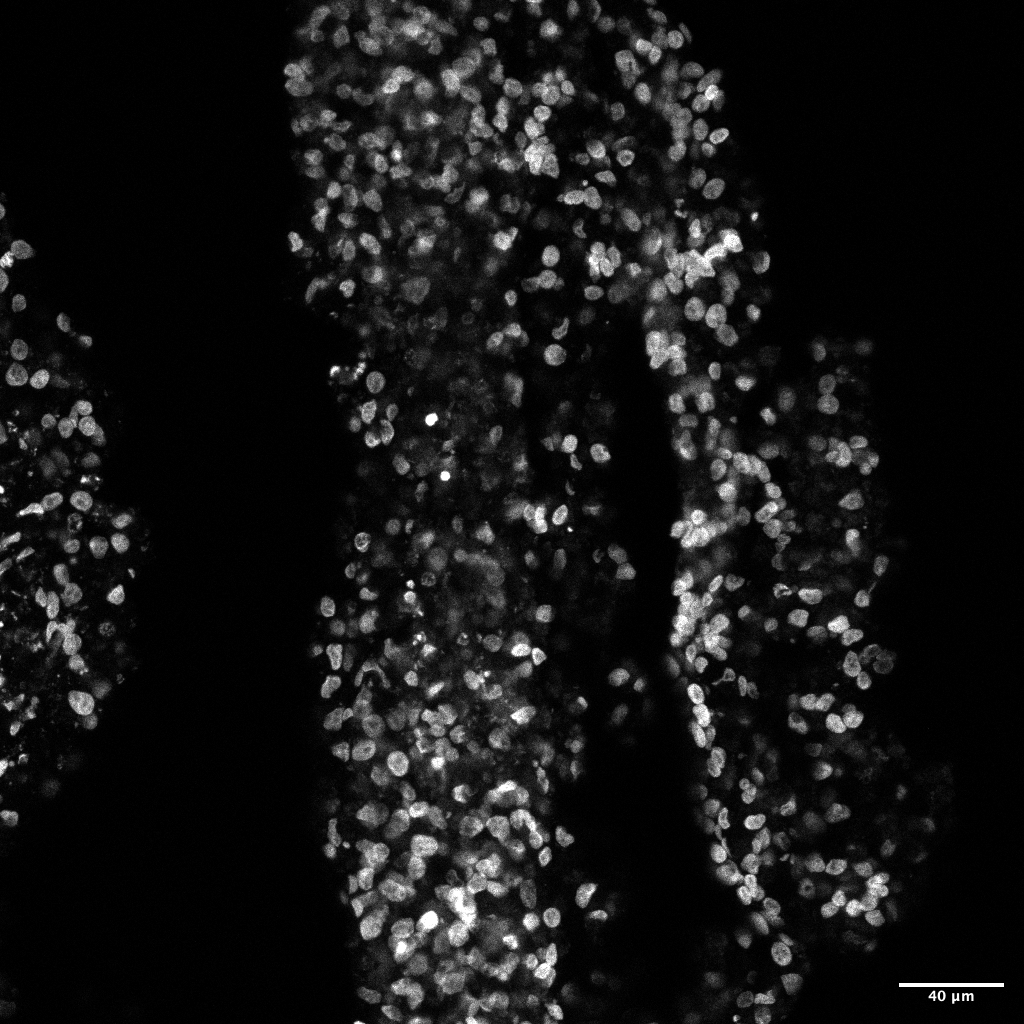

Supplement: Figure 5—source data 2. [file elife-83291-fig5-data2.zip › Figure5_sourcedata_timecourse_F66NR1GF#4/2022-08-23_day8_stainA.lif - D4_1.png]

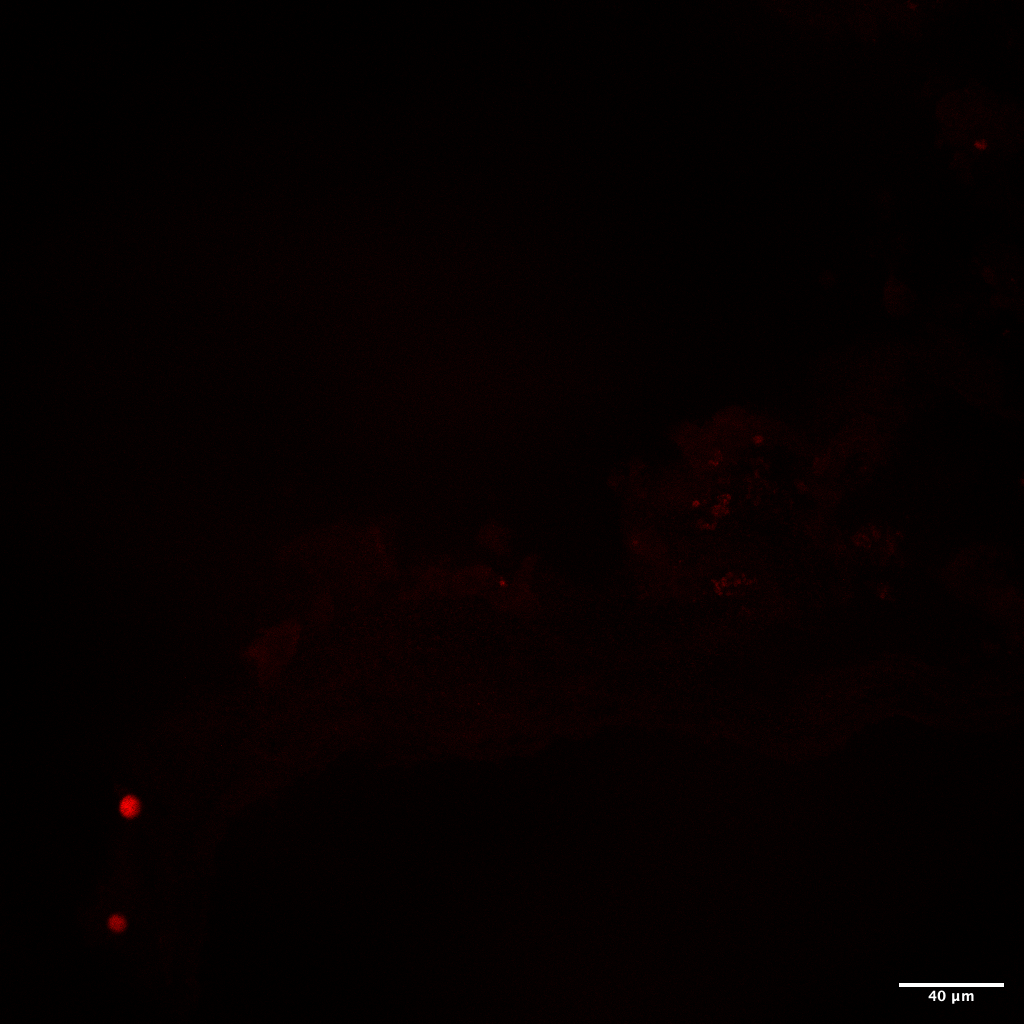

Supplement: Figure 5—source data 2. [file elife-83291-fig5-data2.zip › Figure5_sourcedata_timecourse_F66NR1GF#4/2022-09-12_human_day54_1_OCT4.png]

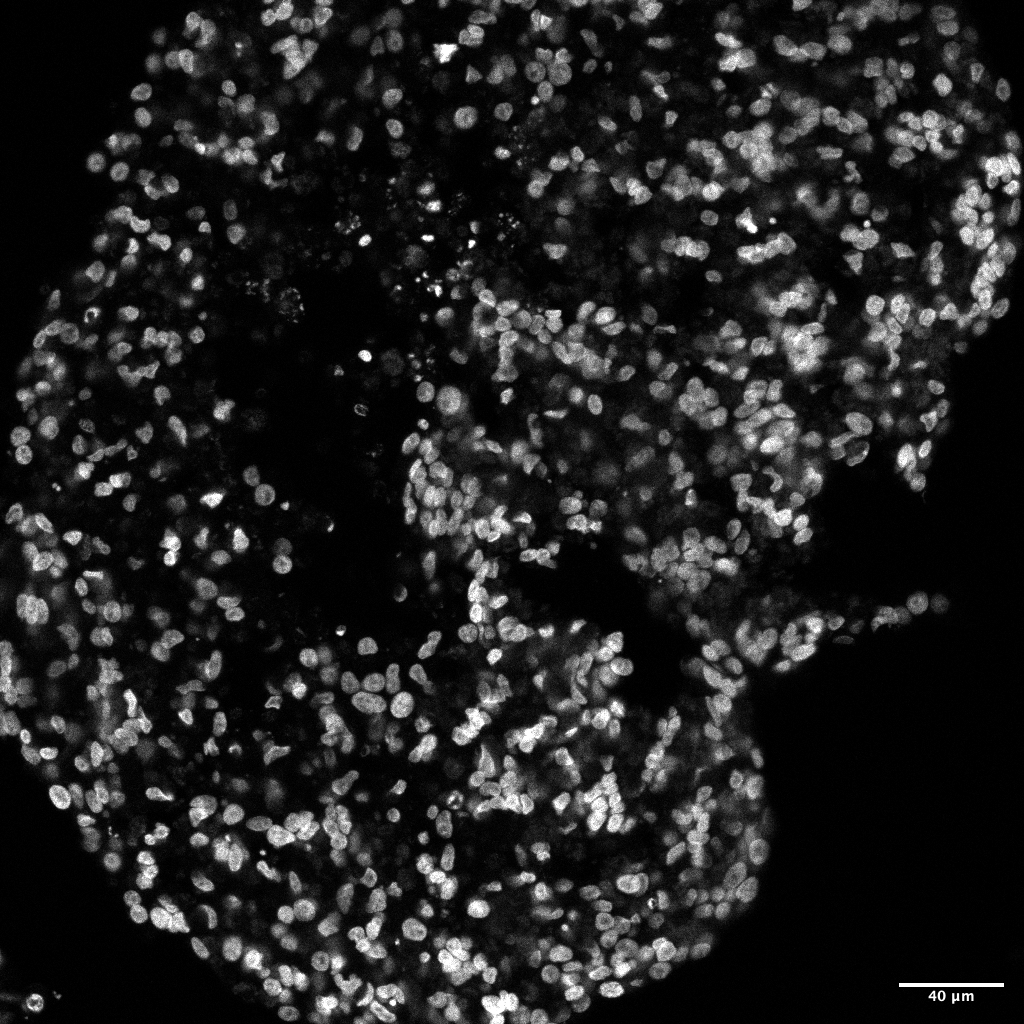

Supplement: Figure 5—source data 2. [file elife-83291-fig5-data2.zip › Figure5_sourcedata_timecourse_F66NR1GF#4/2022-08-23_human_day14_DAPI.png]

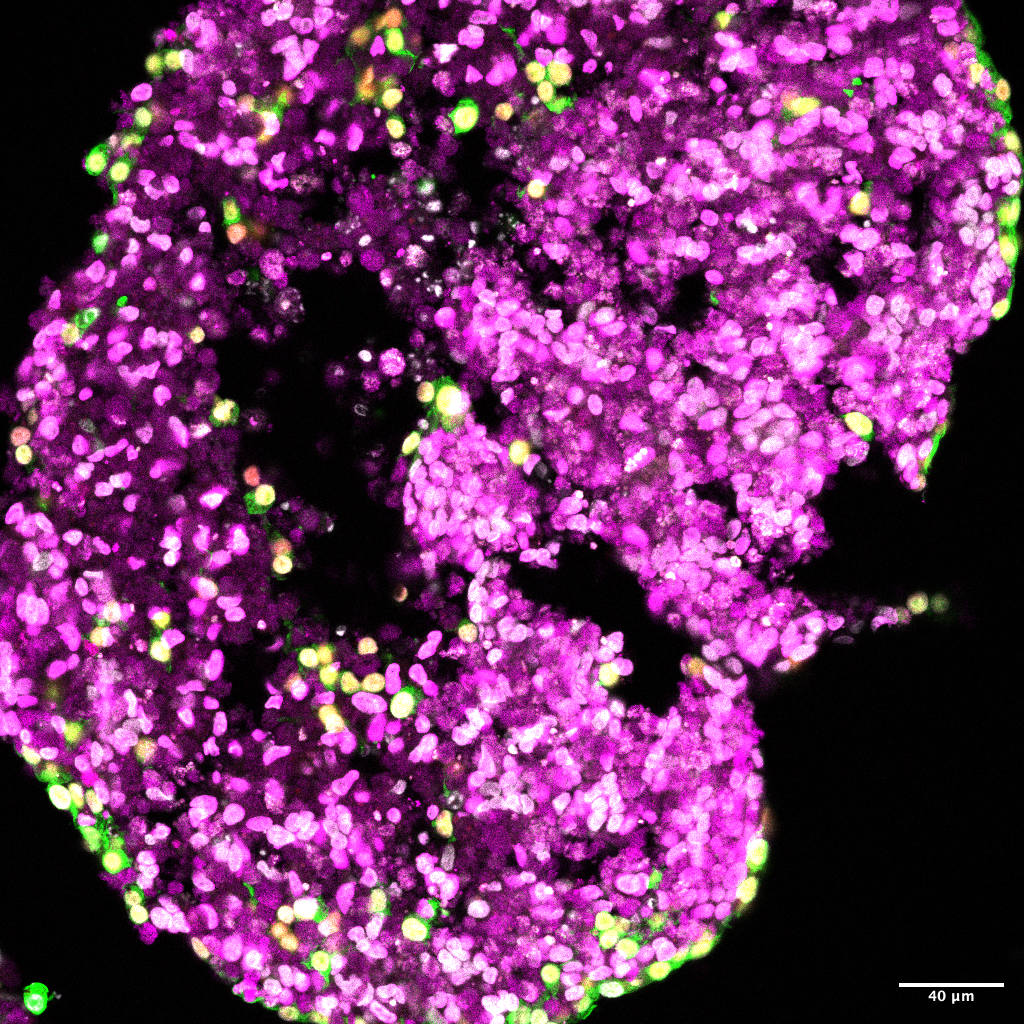

Supplement: Figure 5—source data 2. [file elife-83291-fig5-data2.zip › Figure5_sourcedata_timecourse_F66NR1GF#4/2022-08-23_human_day14_3_composite.png]

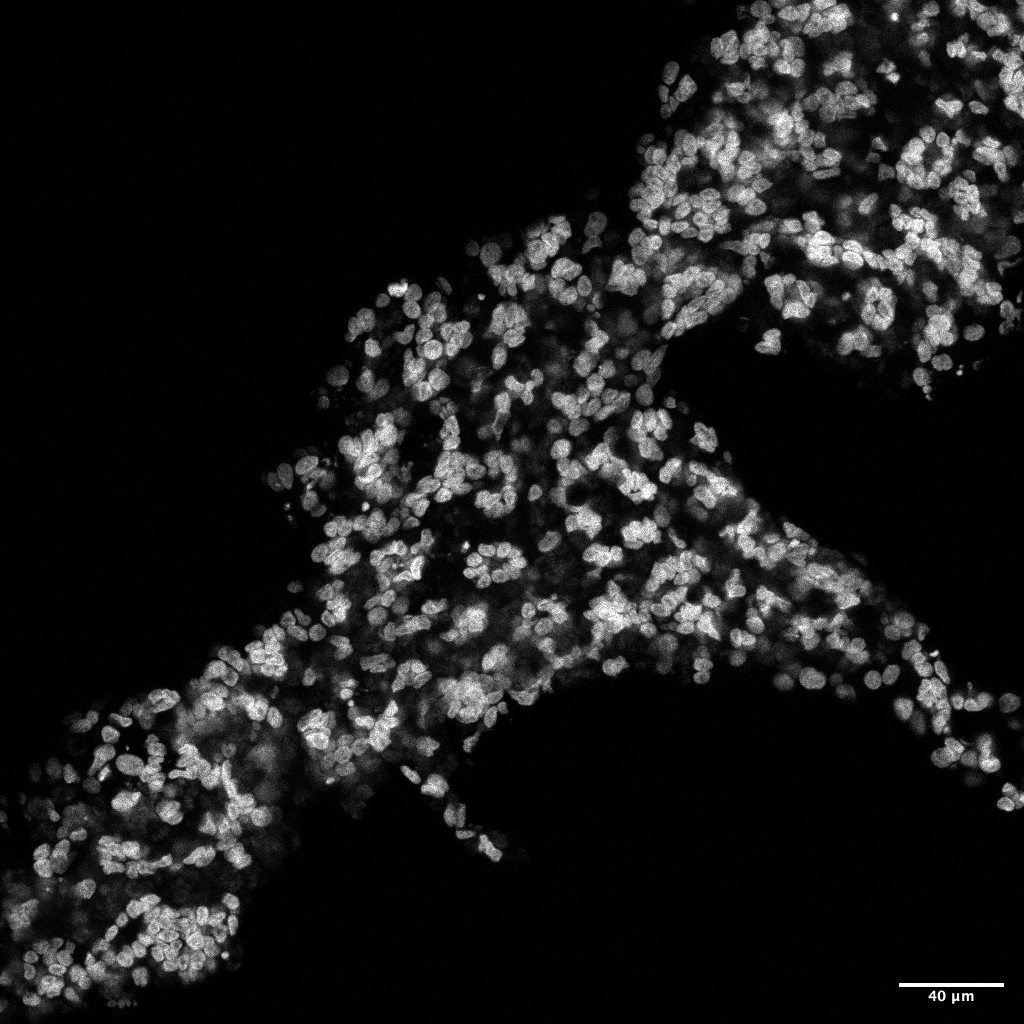

Supplement: Figure 5—source data 2. [file elife-83291-fig5-data2.zip › Figure5_sourcedata_timecourse_F66NR1GF#4/2022-08-25_human_day20_2bDAPI.png]

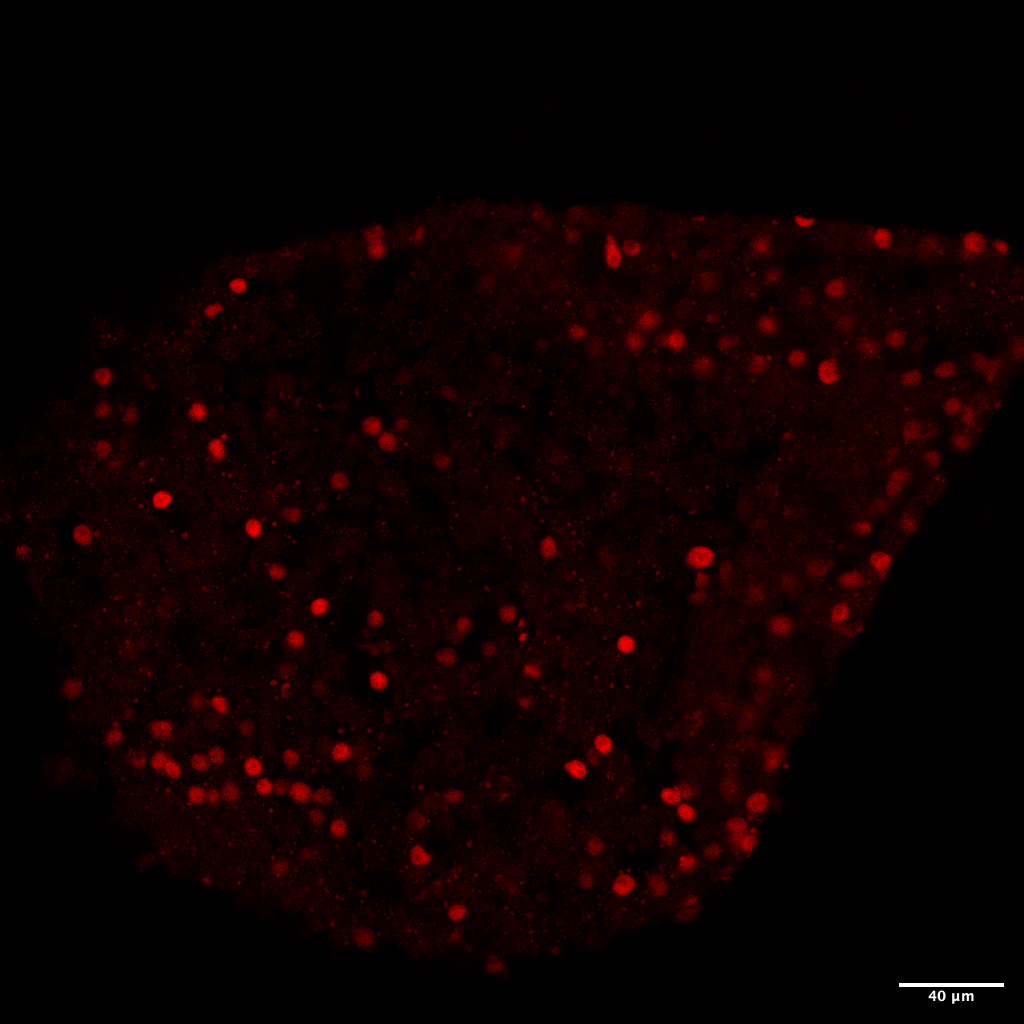

Supplement: Figure 5—source data 2. [file elife-83291-fig5-data2.zip › Figure5_sourcedata_timecourse_F66NR1GF#4/2022-08-24-human_day2_2_OCT4.png]

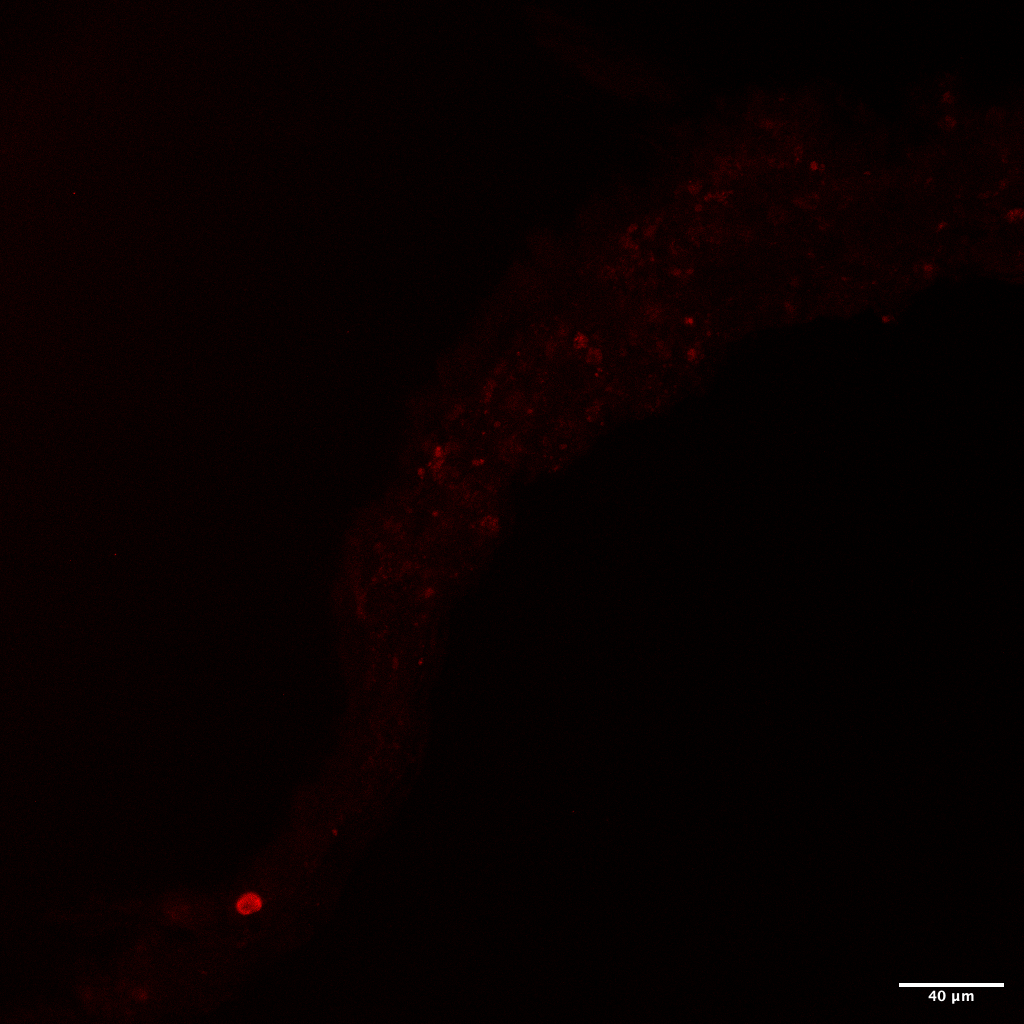

Supplement: Figure 5—source data 2. [file elife-83291-fig5-data2.zip › Figure5_sourcedata_timecourse_F66NR1GF#4/2022-09-12_human_day38_OCT4.png]

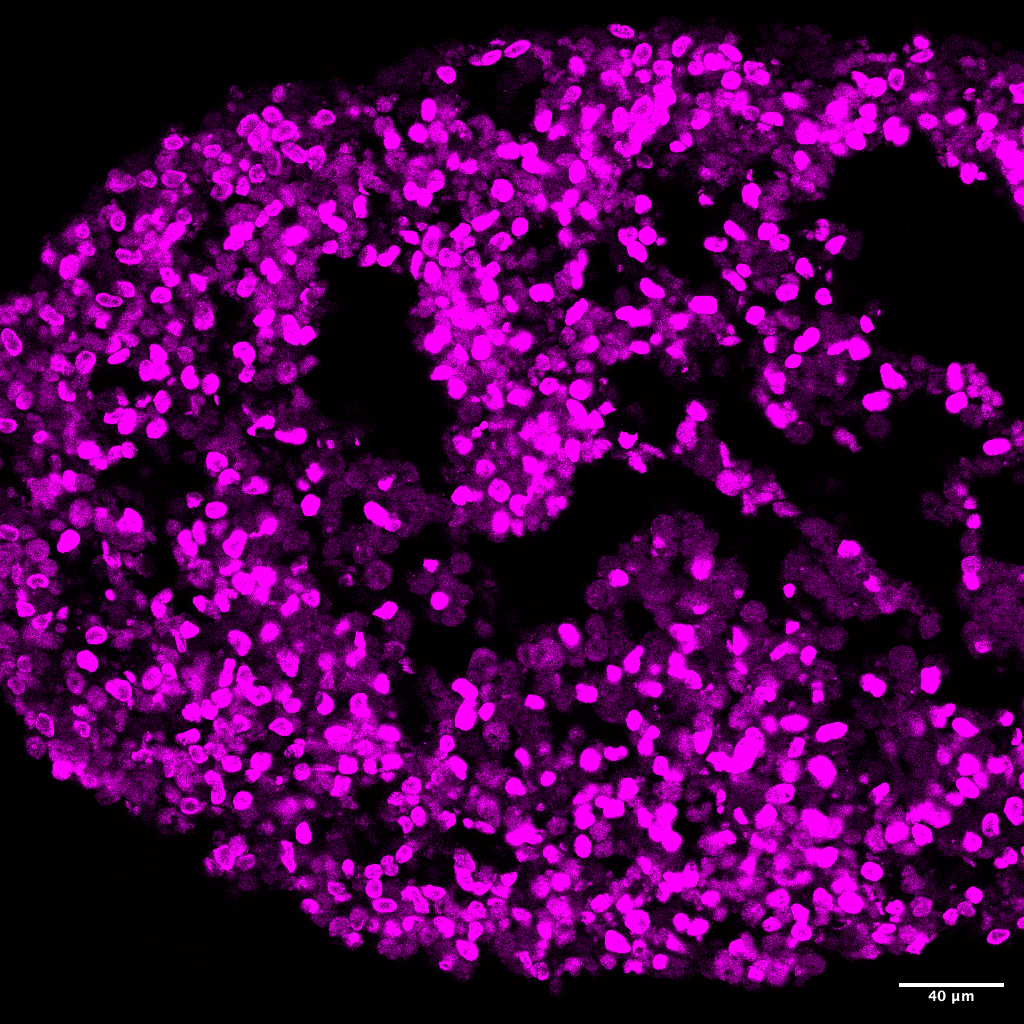

Supplement: Figure 5—source data 2. [file elife-83291-fig5-data2.zip › Figure5_sourcedata_timecourse_F66NR1GF#4/2022-08-24-human_day4_1_FOXL2.png]

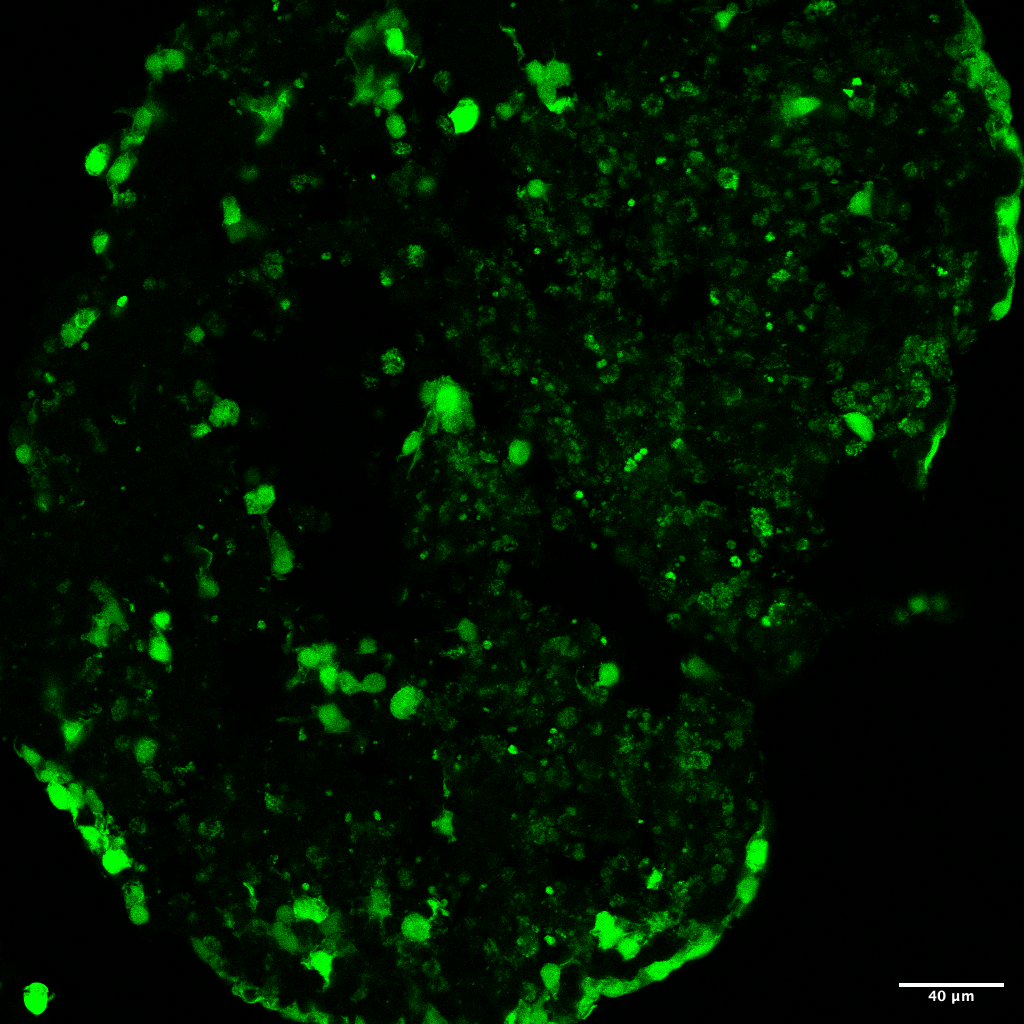

Supplement: Figure 5—source data 2. [file elife-83291-fig5-data2.zip › Figure5_sourcedata_timecourse_F66NR1GF#4/2022-08-23_human_day14_3_DAZL.png]

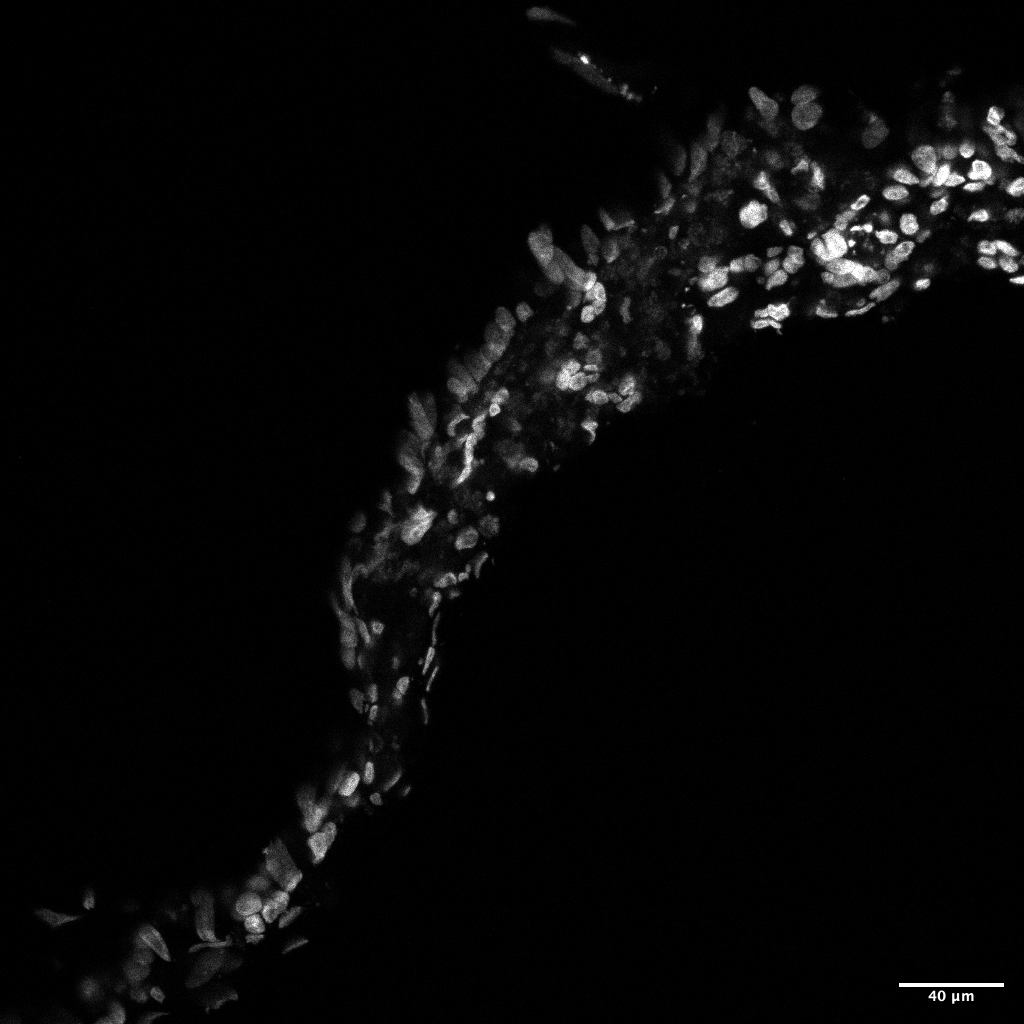

Supplement: Figure 5—source data 2. [file elife-83291-fig5-data2.zip › Figure5_sourcedata_timecourse_F66NR1GF#4/2022-09-12_human_day38DAPI.png]

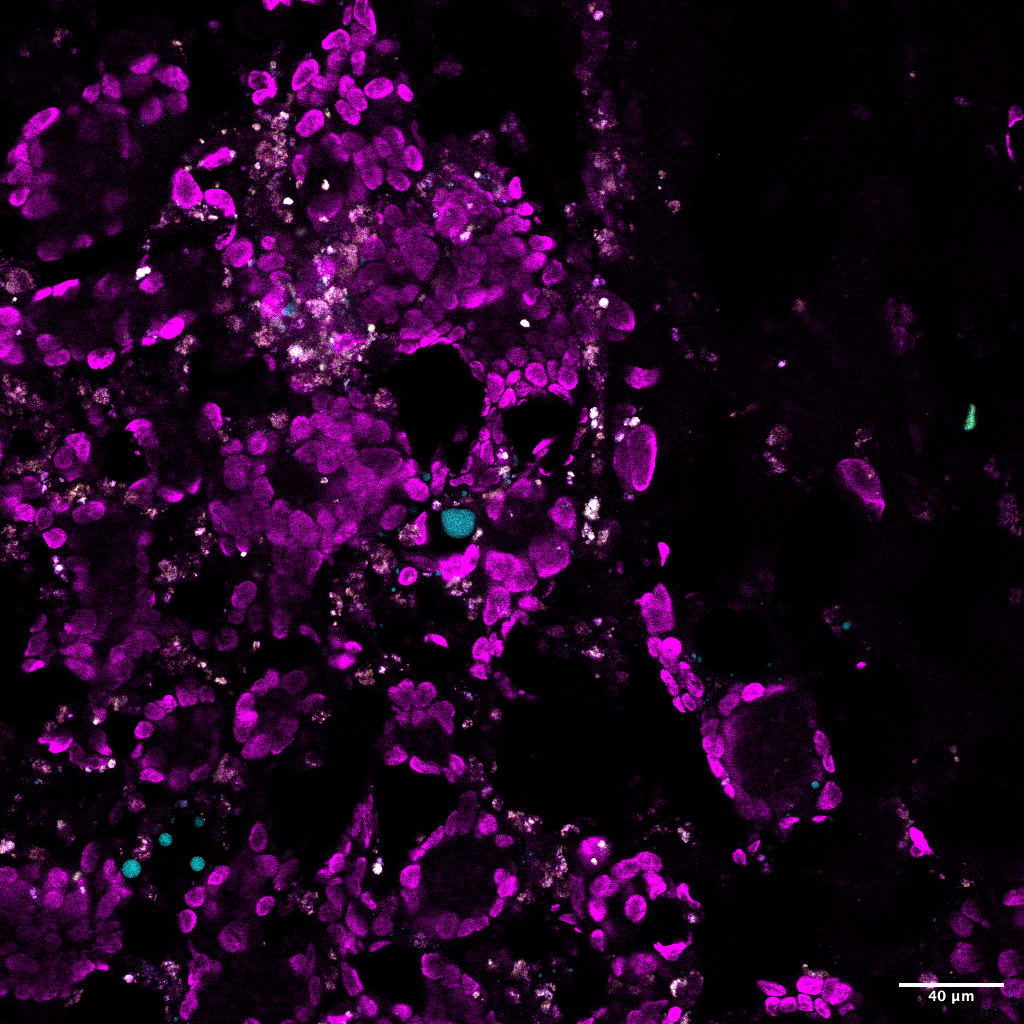

Supplement: Figure 5—source data 2. [file elife-83291-fig5-data2.zip › Figure5_sourcedata_timecourse_F66NR1GF#4/Day70_OCT4_composite.png]

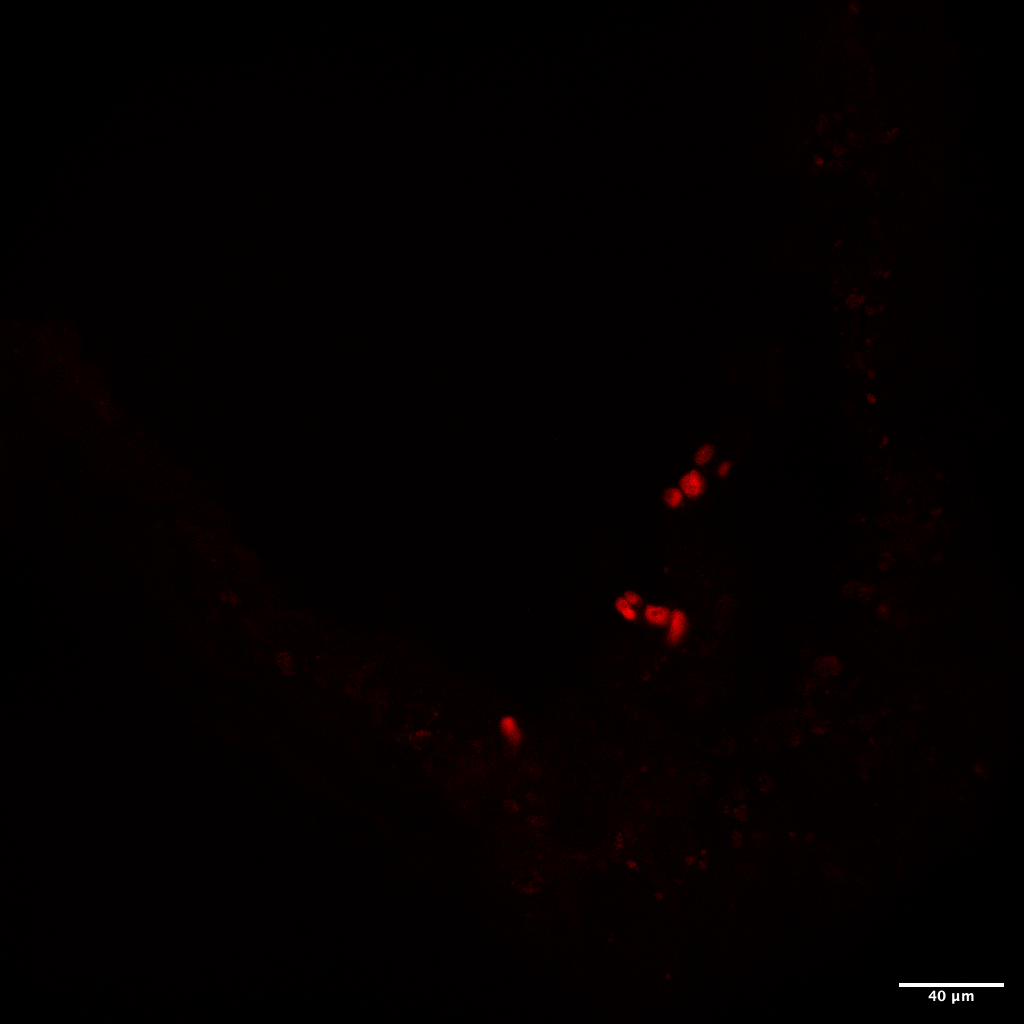

Supplement: Figure 5—source data 2. [file elife-83291-fig5-data2.zip › Figure5_sourcedata_timecourse_F66NR1GF#4/2022-09-12_human_day46_A_OCT4.png]

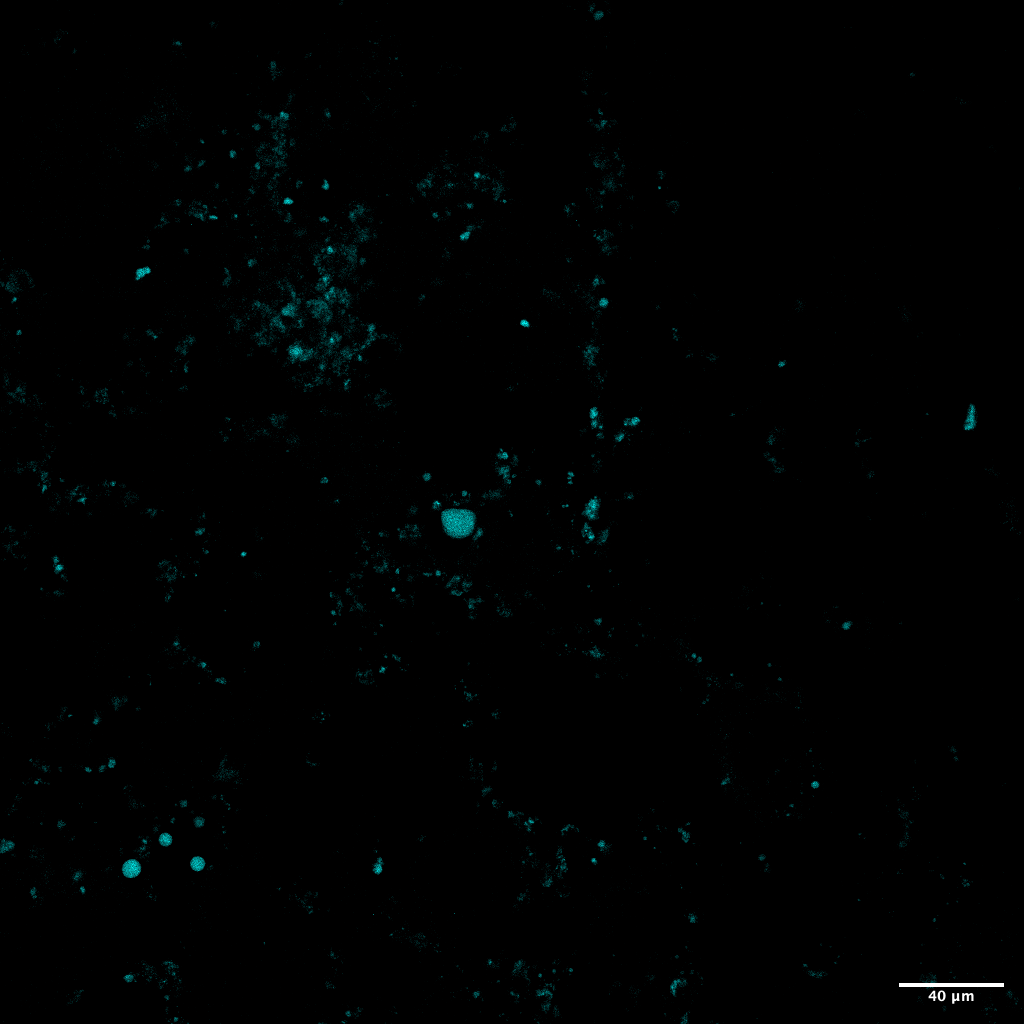

Supplement: Figure 5—source data 2. [file elife-83291-fig5-data2.zip › Figure5_sourcedata_timecourse_F66NR1GF#4/Day70_OCT4.png]

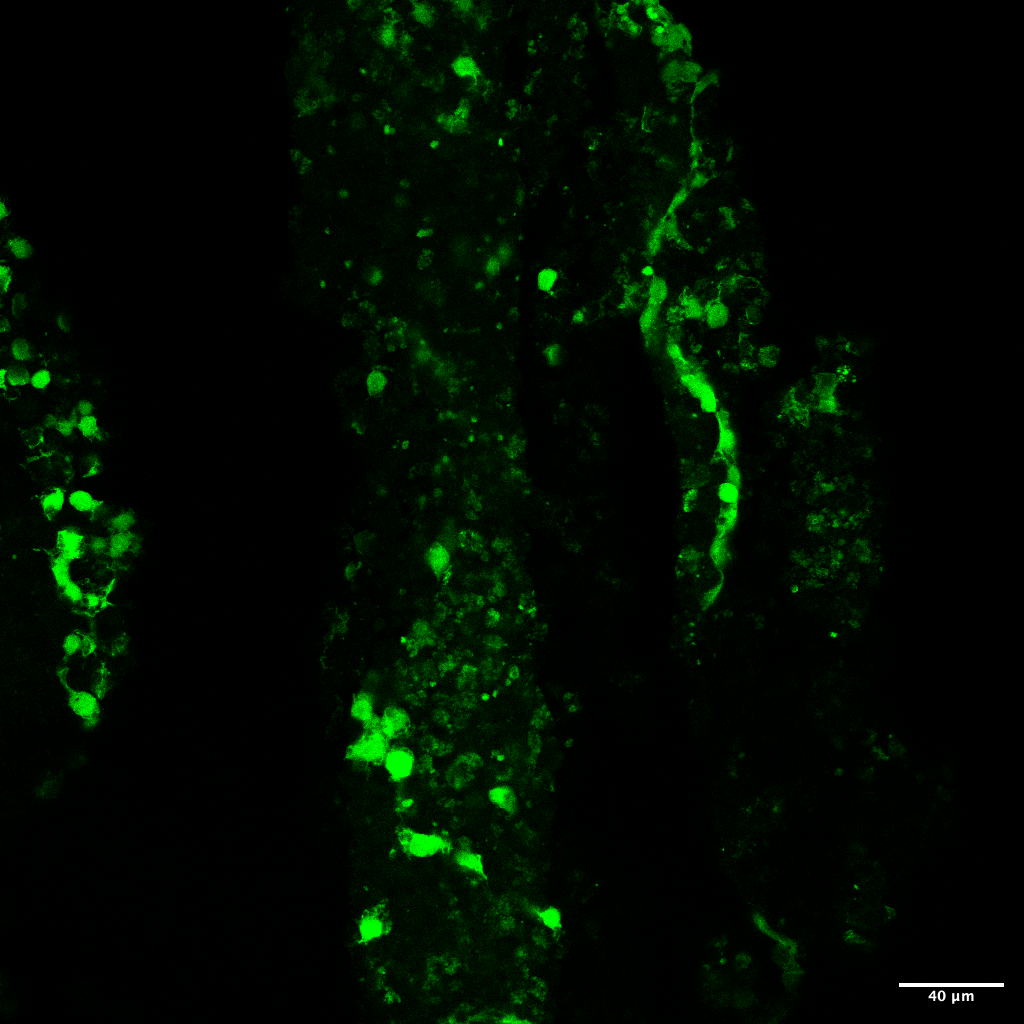

Supplement: Figure 5—source data 2. [file elife-83291-fig5-data2.zip › Figure5_sourcedata_timecourse_F66NR1GF#4/2022-08-23_day8_stainA.lif - DAZL.png]

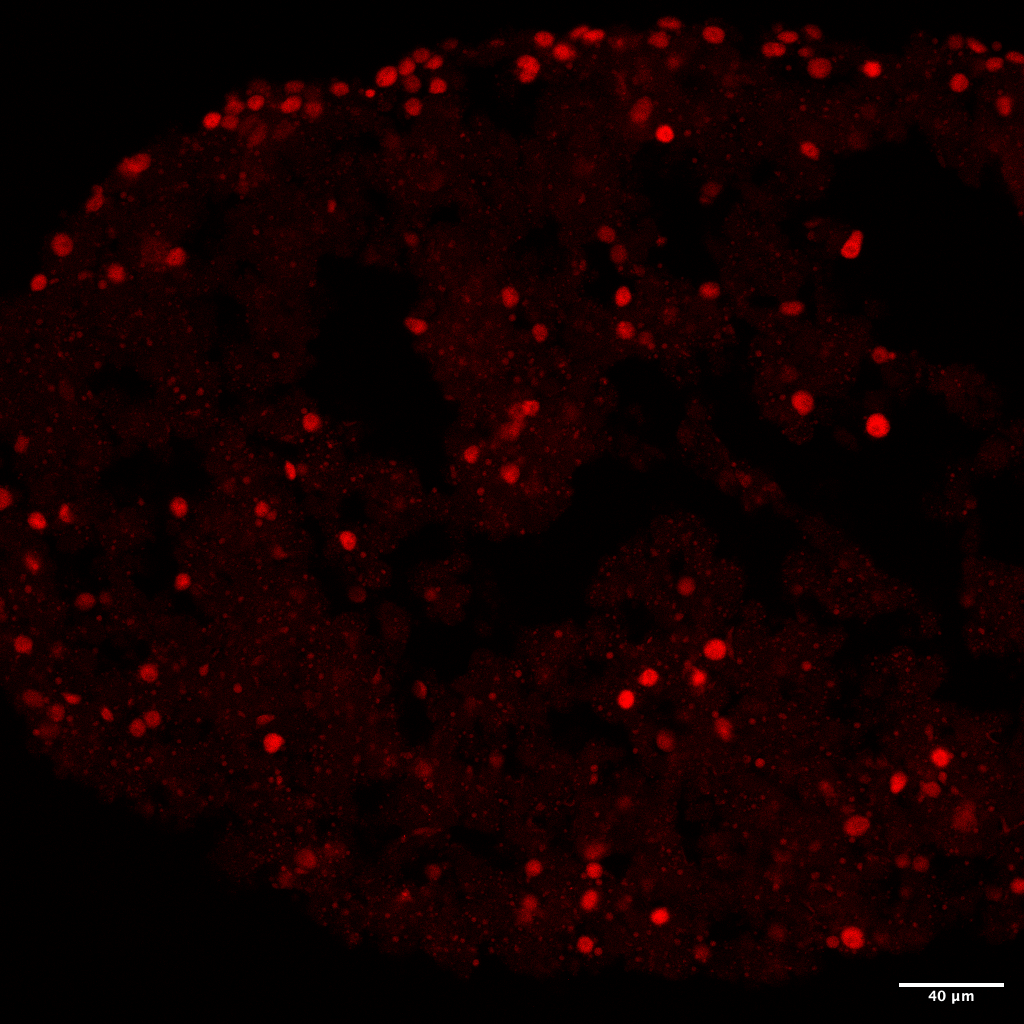

Supplement: Figure 5—source data 2. [file elife-83291-fig5-data2.zip › Figure5_sourcedata_timecourse_F66NR1GF#4/2022-08-24-human_day4_1_OCT4.png]

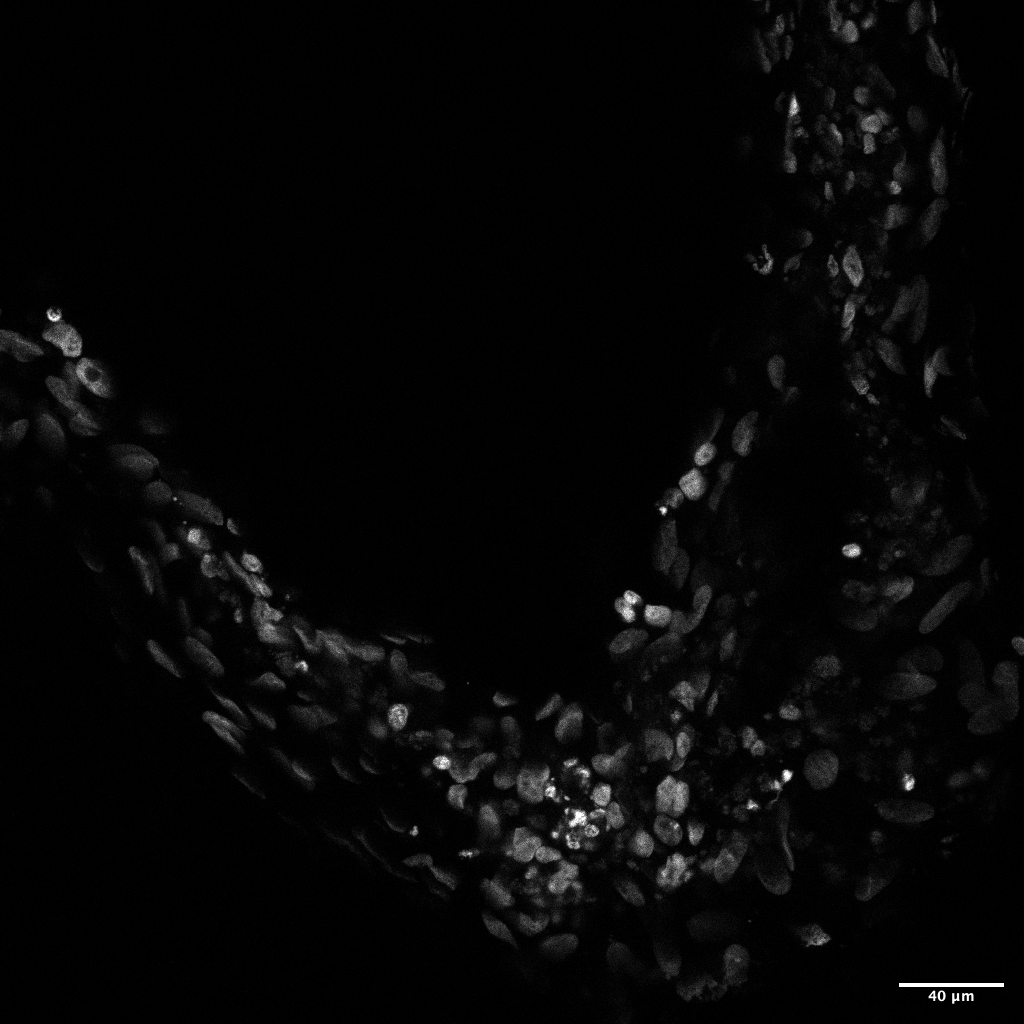

Supplement: Figure 5—source data 2. [file elife-83291-fig5-data2.zip › Figure5_sourcedata_timecourse_F66NR1GF#4/2022-09-12_human_day46_ADAPI.png]

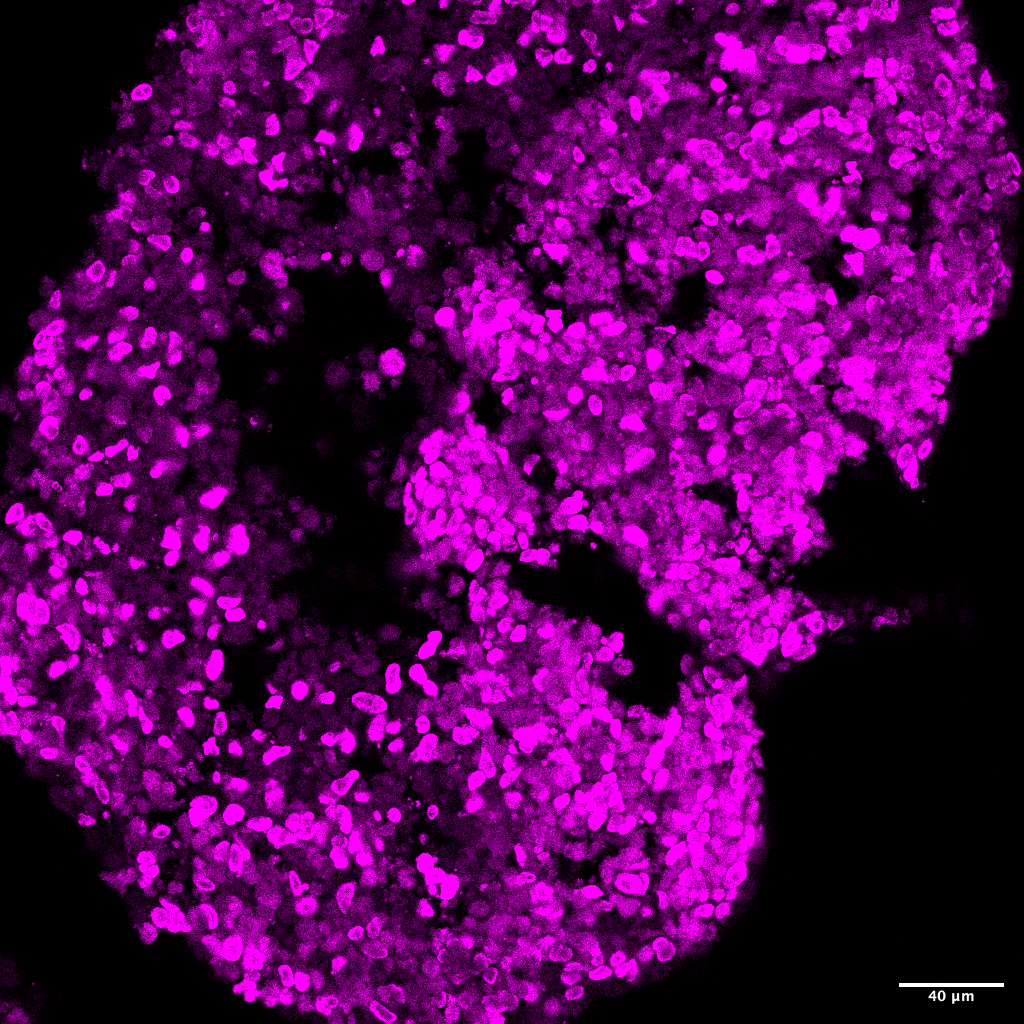

Supplement: Figure 5—source data 2. [file elife-83291-fig5-data2.zip › Figure5_sourcedata_timecourse_F66NR1GF#4/2022-08-23_human_day14_3_FOXL2.png]

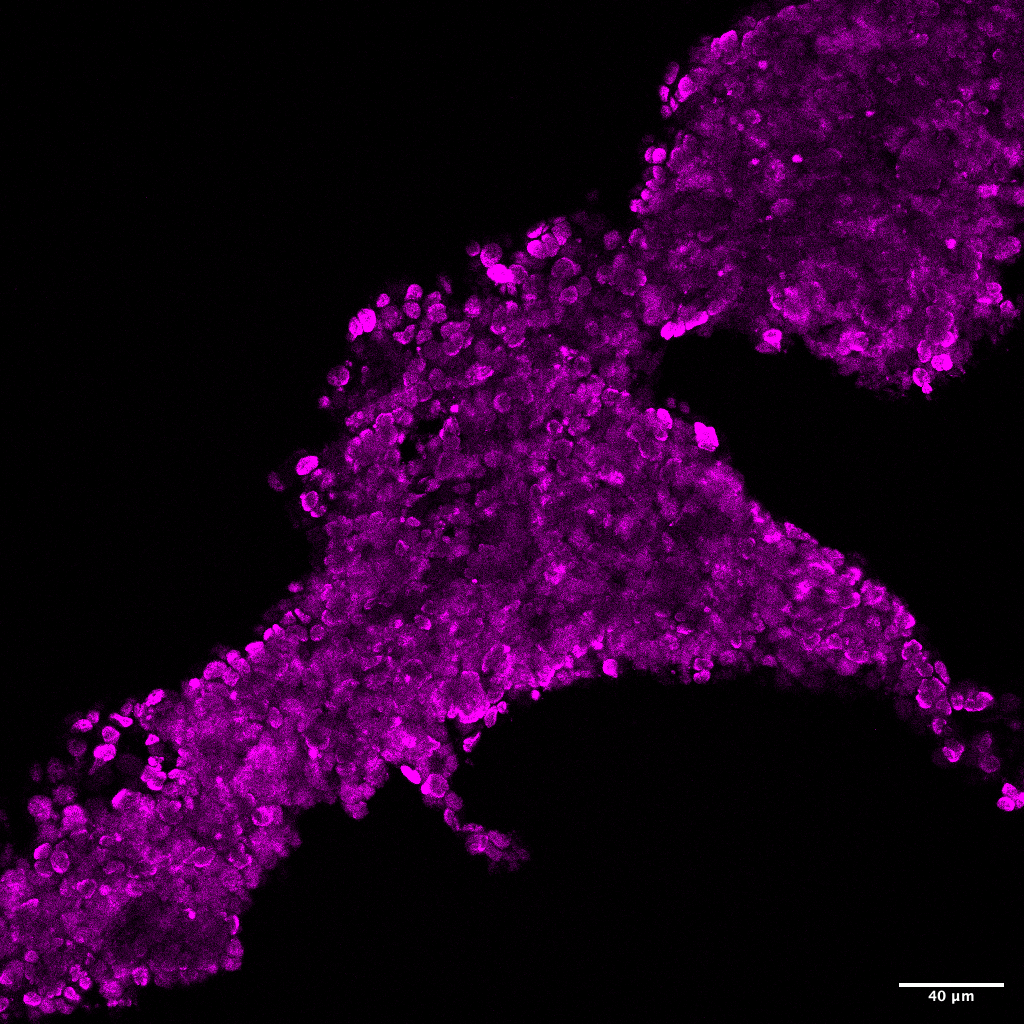

Supplement: Figure 5—source data 2. [file elife-83291-fig5-data2.zip › Figure5_sourcedata_timecourse_F66NR1GF#4/2022-08-25_human_day20_2b_FOXL2.png]

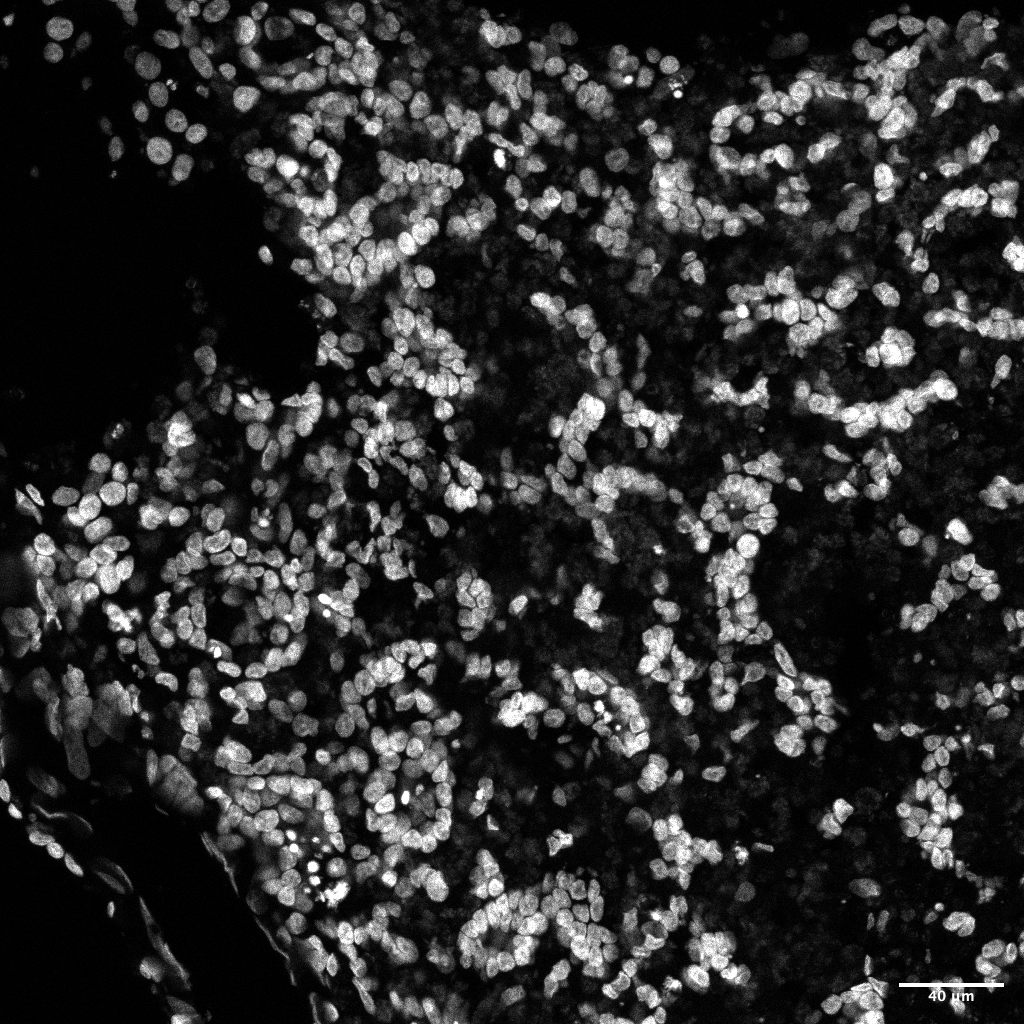

Supplement: Figure 5—source data 2. [file elife-83291-fig5-data2.zip › Figure5_sourcedata_timecourse_F66NR1GF#4/2022-08-25-human_day32_DAPI.png]

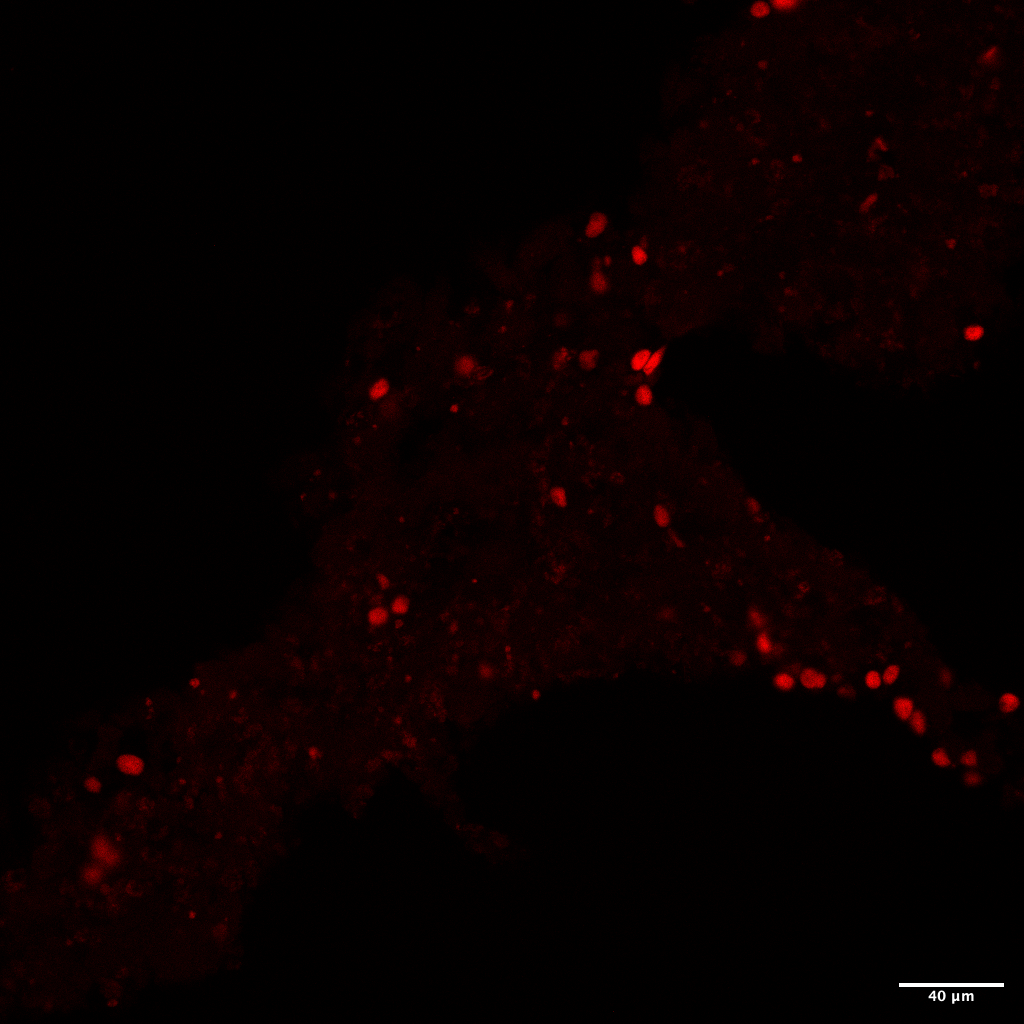

Supplement: Figure 5—source data 2. [file elife-83291-fig5-data2.zip › Figure5_sourcedata_timecourse_F66NR1GF#4/2022-08-25_human_day20_2b_OCT4.png]

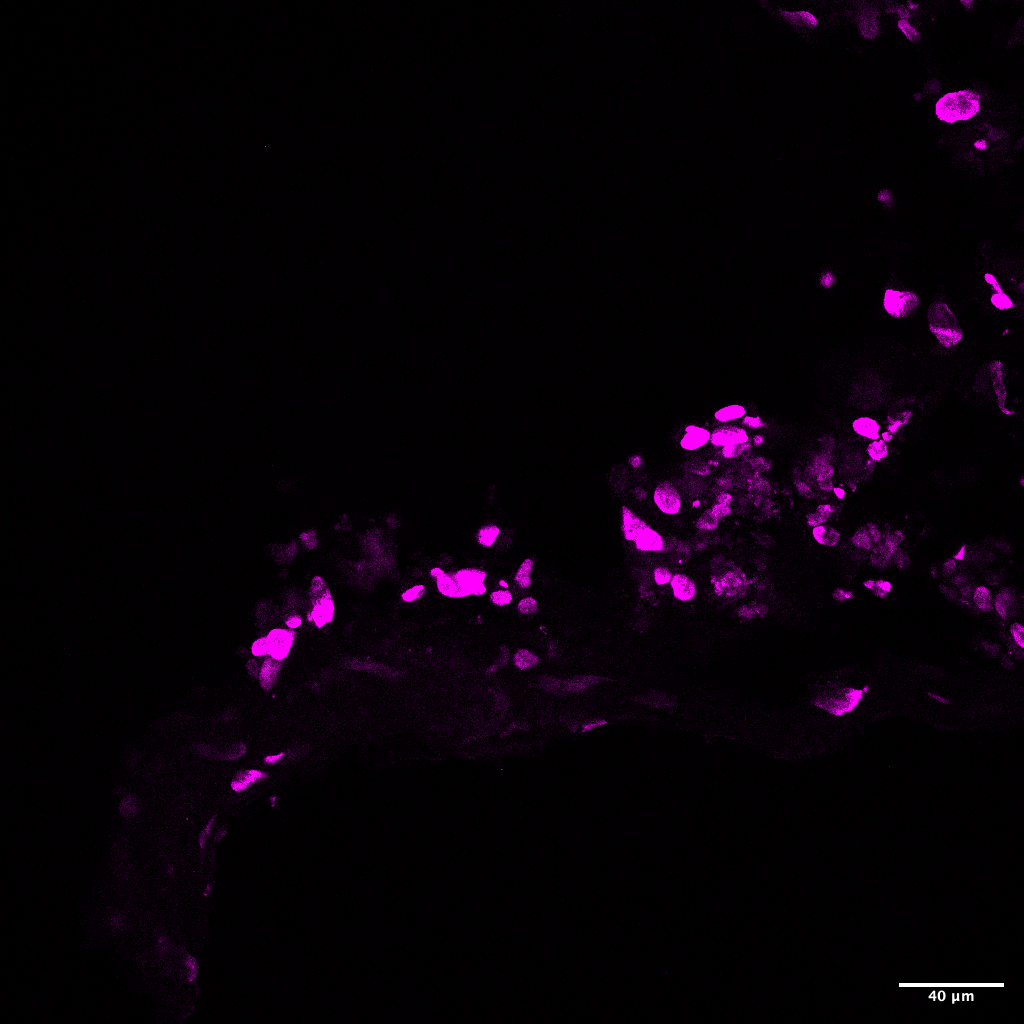

Supplement: Figure 5—source data 2. [file elife-83291-fig5-data2.zip › Figure5_sourcedata_timecourse_F66NR1GF#4/2022-09-12_human_day54_1_FOXL2.png]

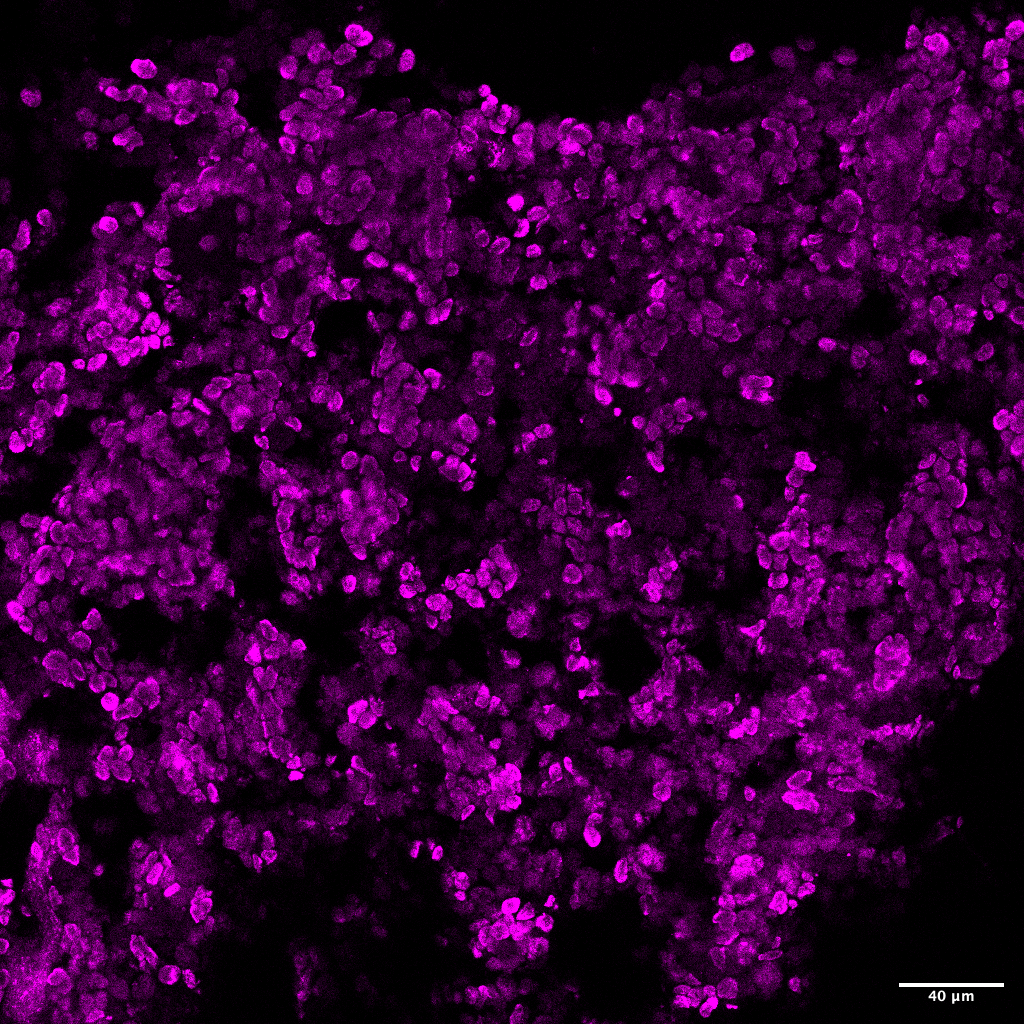

Supplement: Figure 5—source data 2. [file elife-83291-fig5-data2.zip › Figure5_sourcedata_timecourse_F66NR1GF#4/2022-08-24-human_day26_2_FOXL2.png]

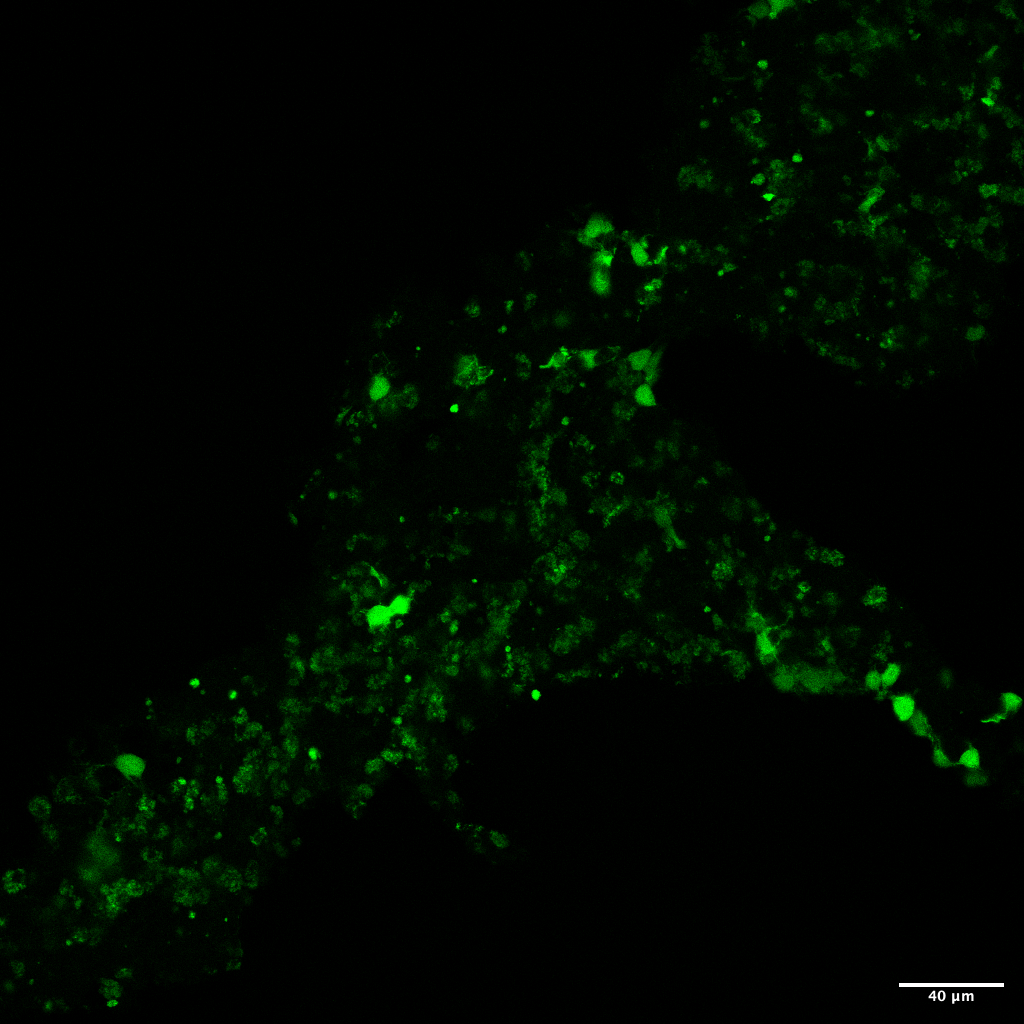

Supplement: Figure 5—source data 2. [file elife-83291-fig5-data2.zip › Figure5_sourcedata_timecourse_F66NR1GF#4/2022-08-25_human_day20_2b_DAZL.png]

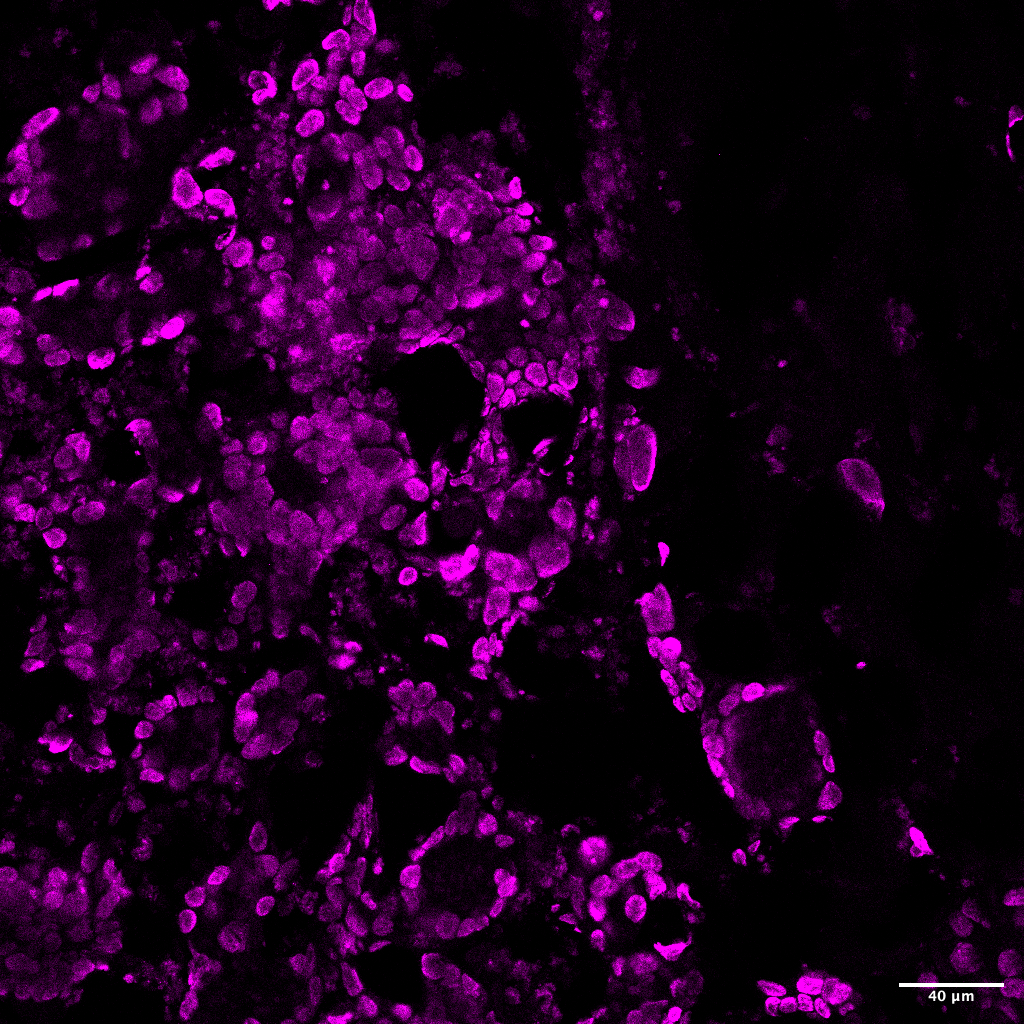

Supplement: Figure 5—source data 2. [file elife-83291-fig5-data2.zip › Figure5_sourcedata_timecourse_F66NR1GF#4/Day70_FOXL2.png]

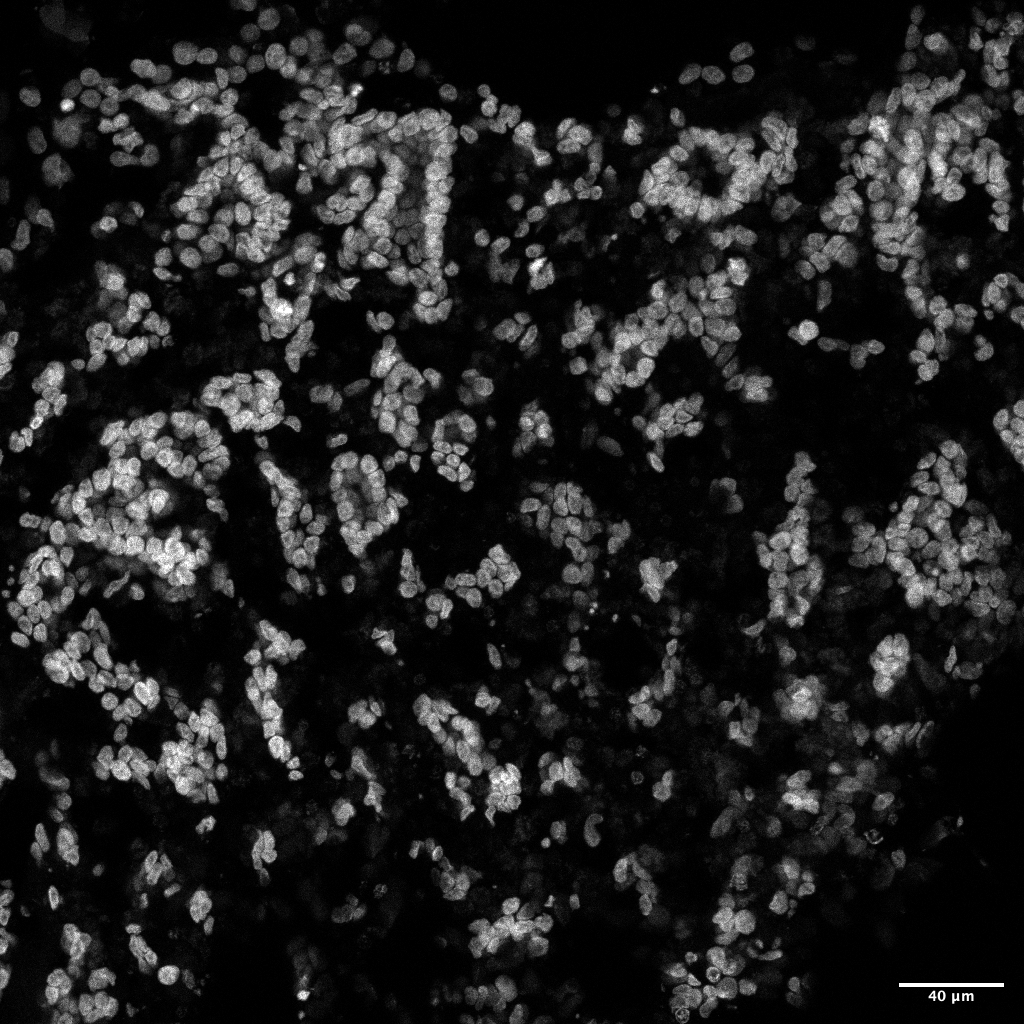

Supplement: Figure 5—source data 2. [file elife-83291-fig5-data2.zip › Figure5_sourcedata_timecourse_F66NR1GF#4/2022-08-24-human_day26_DAPI.png]

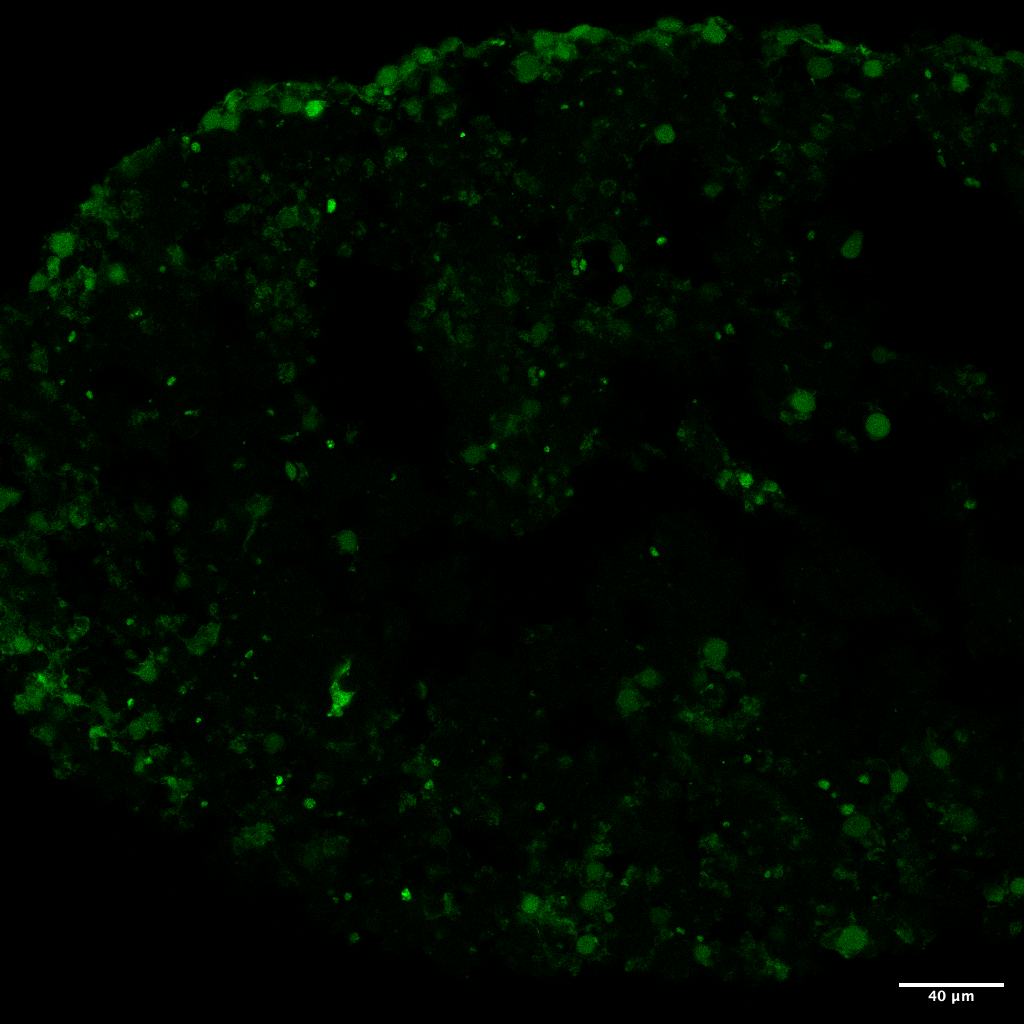

Supplement: Figure 5—source data 2. [file elife-83291-fig5-data2.zip › Figure5_sourcedata_timecourse_F66NR1GF#4/2022-08-24-human_day4_1_DAZL.png]

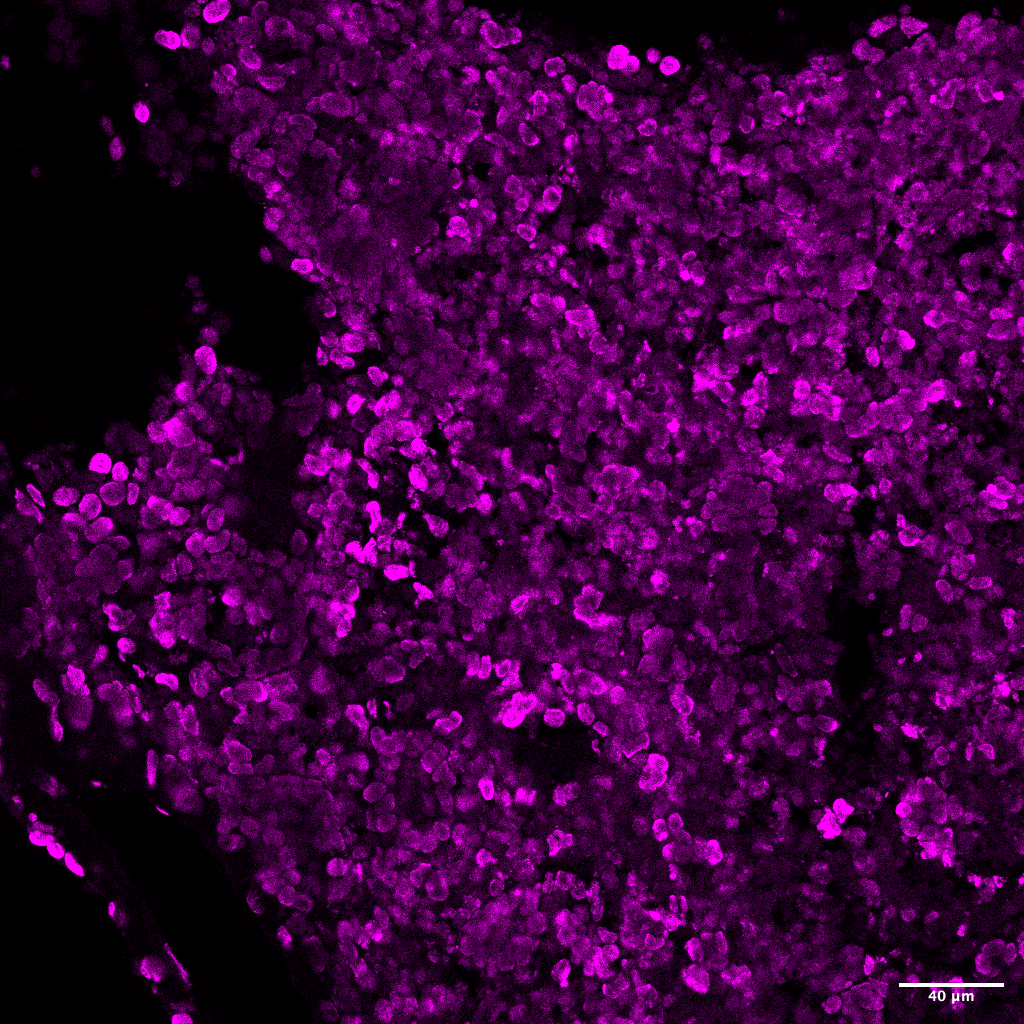

Supplement: Figure 5—source data 2. [file elife-83291-fig5-data2.zip › Figure5_sourcedata_timecourse_F66NR1GF#4/2022-08-25-human_day32_1_FOXL2.png]

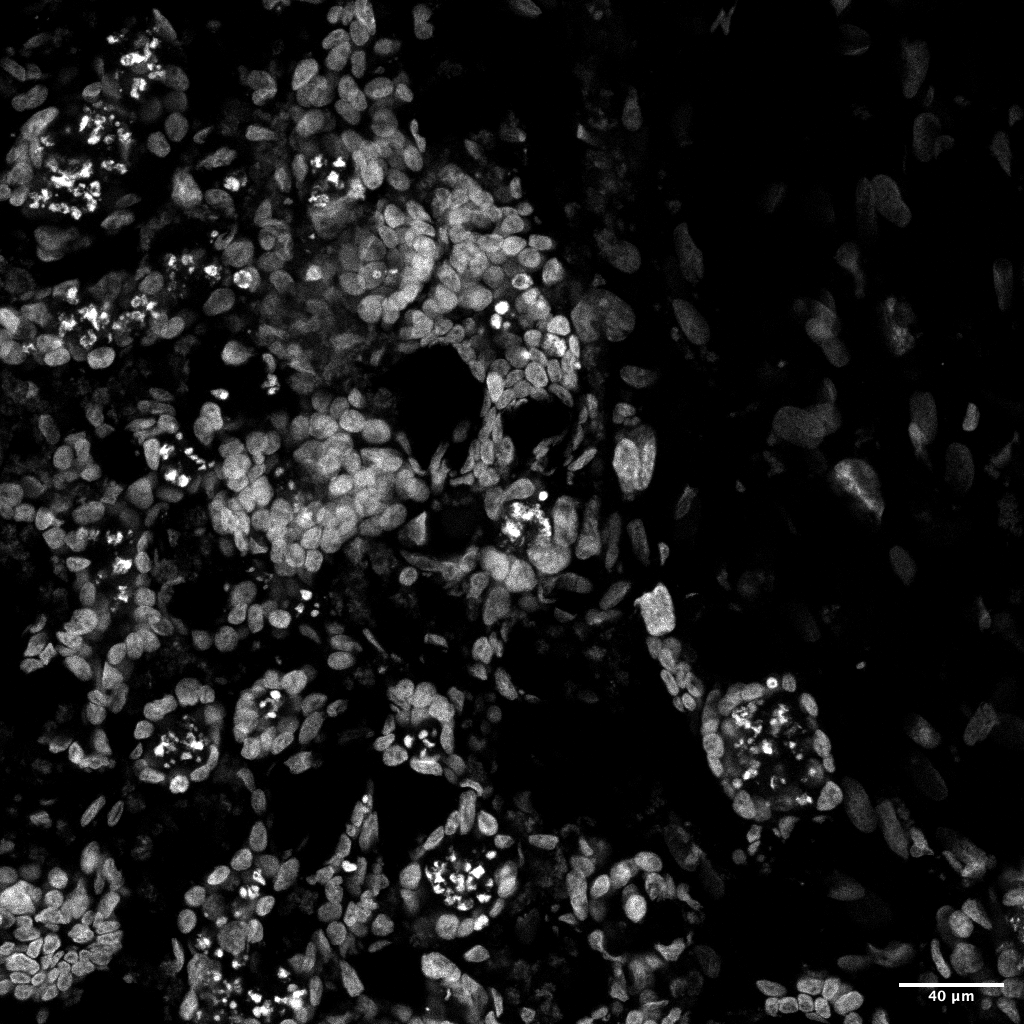

Supplement: Figure 5—source data 2. [file elife-83291-fig5-data2.zip › Figure5_sourcedata_timecourse_F66NR1GF#4/Day70_DAPI.png]

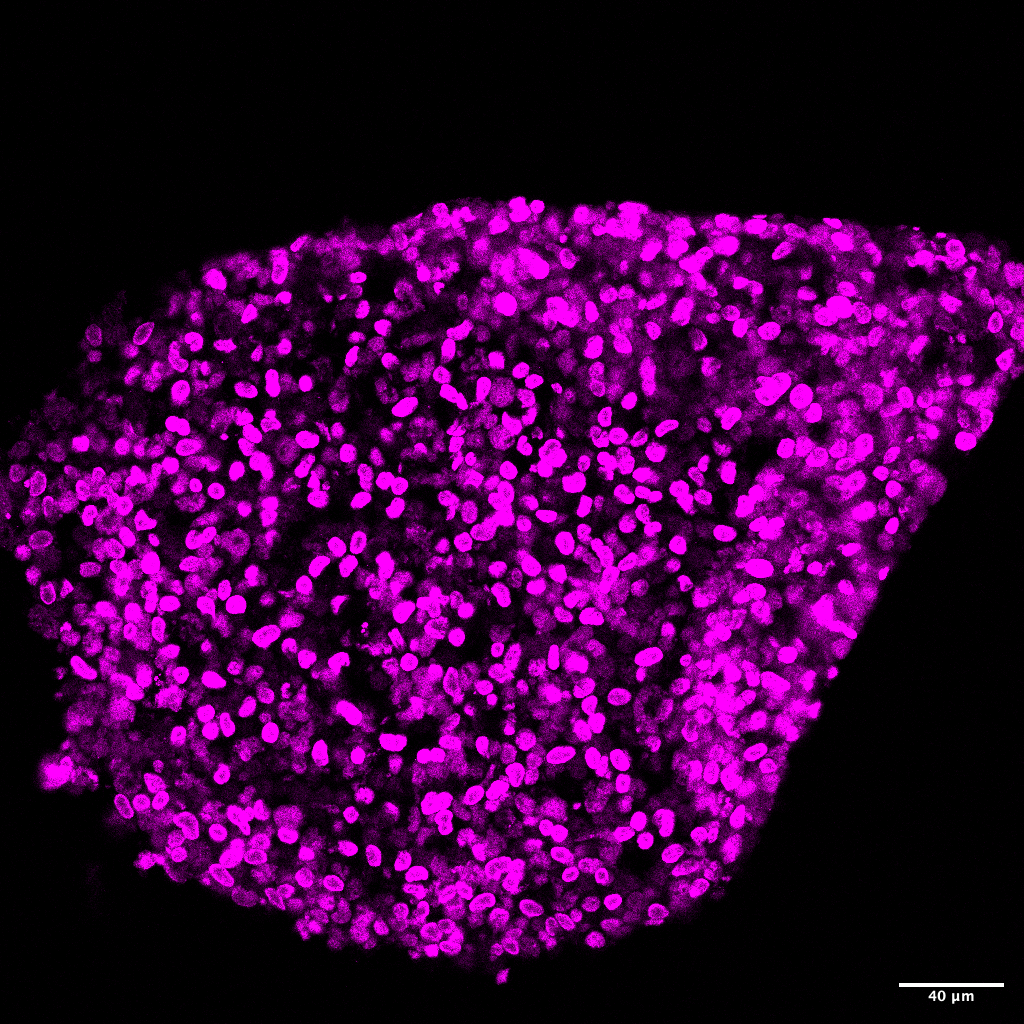

Supplement: Figure 5—source data 2. [file elife-83291-fig5-data2.zip › Figure5_sourcedata_timecourse_F66NR1GF#4/2022-08-24-human_day2_2_FOXL2.png]

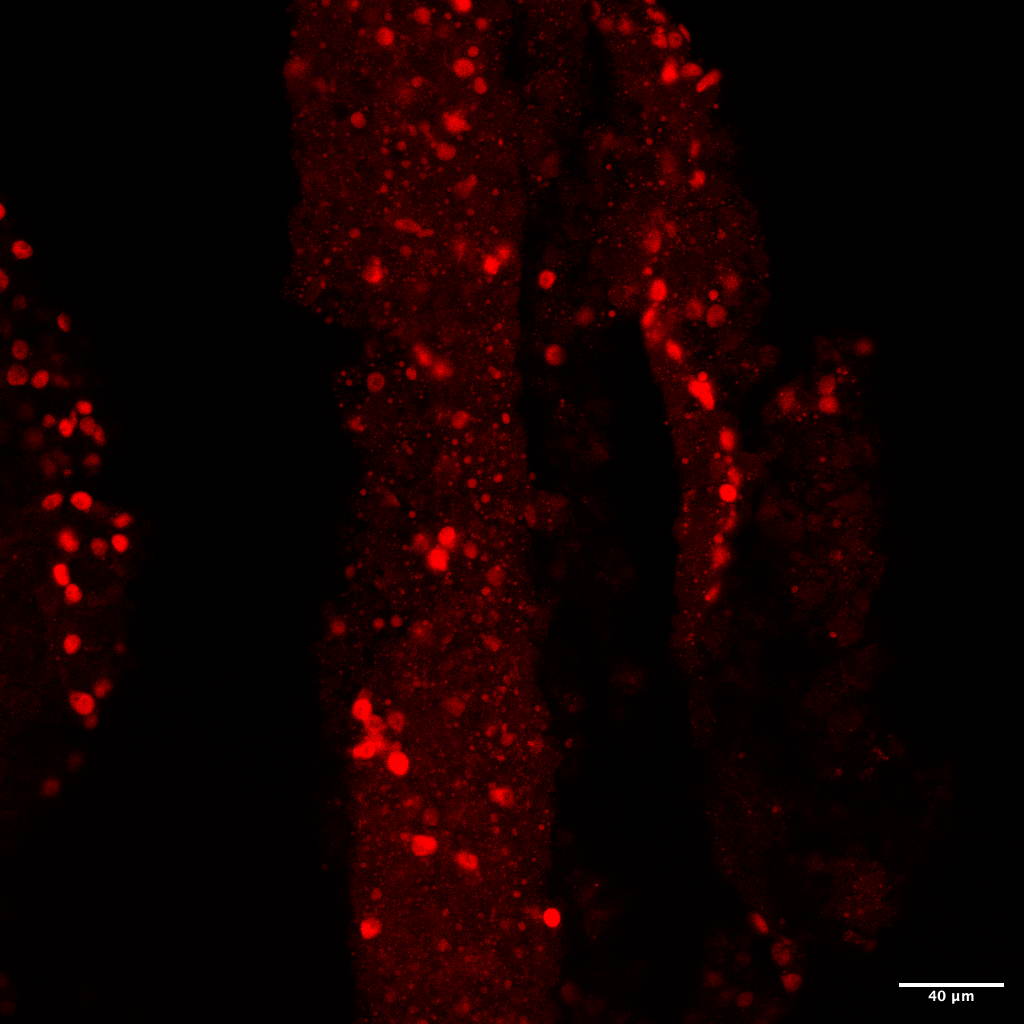

Supplement: Figure 5—source data 2. [file elife-83291-fig5-data2.zip › Figure5_sourcedata_timecourse_F66NR1GF#4/2022-08-23_day8_stainA.lif - OCT4.png]

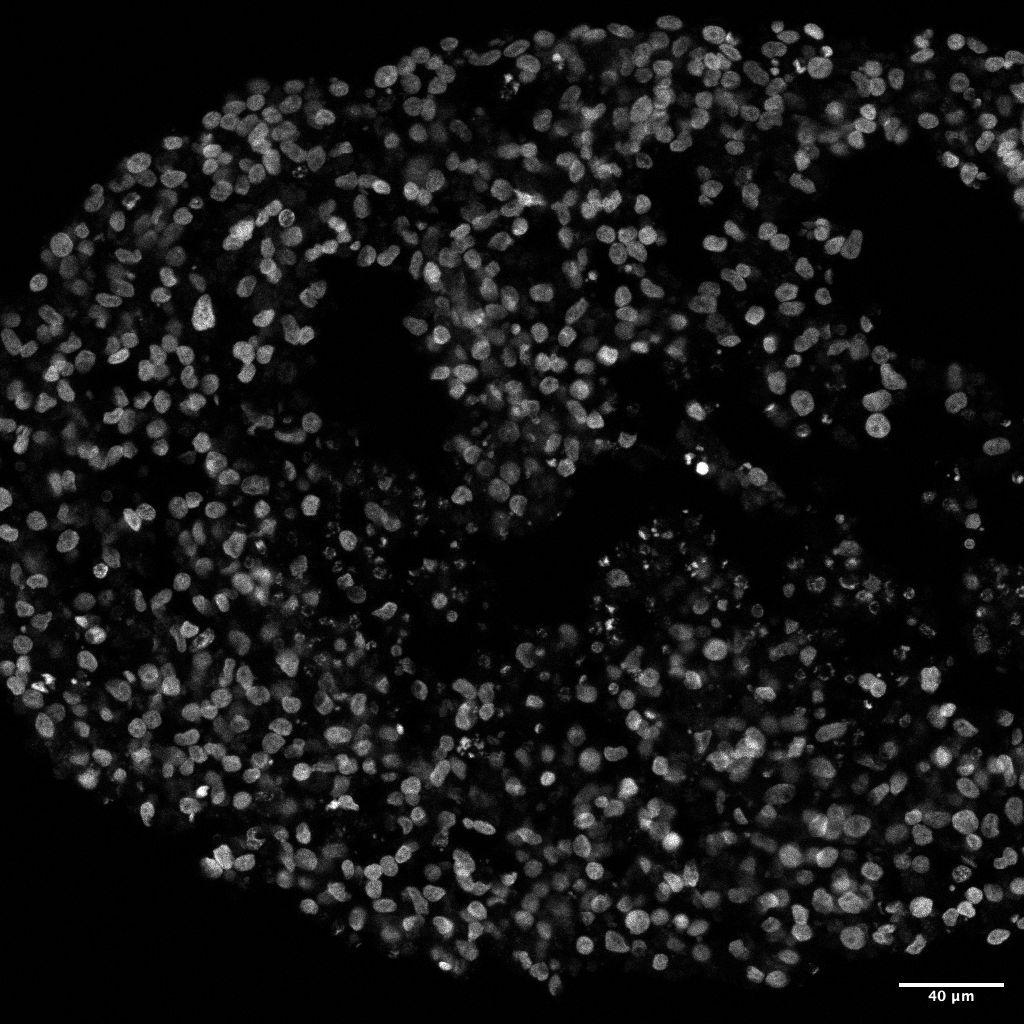

Supplement: Figure 5—source data 2. [file elife-83291-fig5-data2.zip › Figure5_sourcedata_timecourse_F66NR1GF#4/2022-08-24-human_day4_DAPI.png]

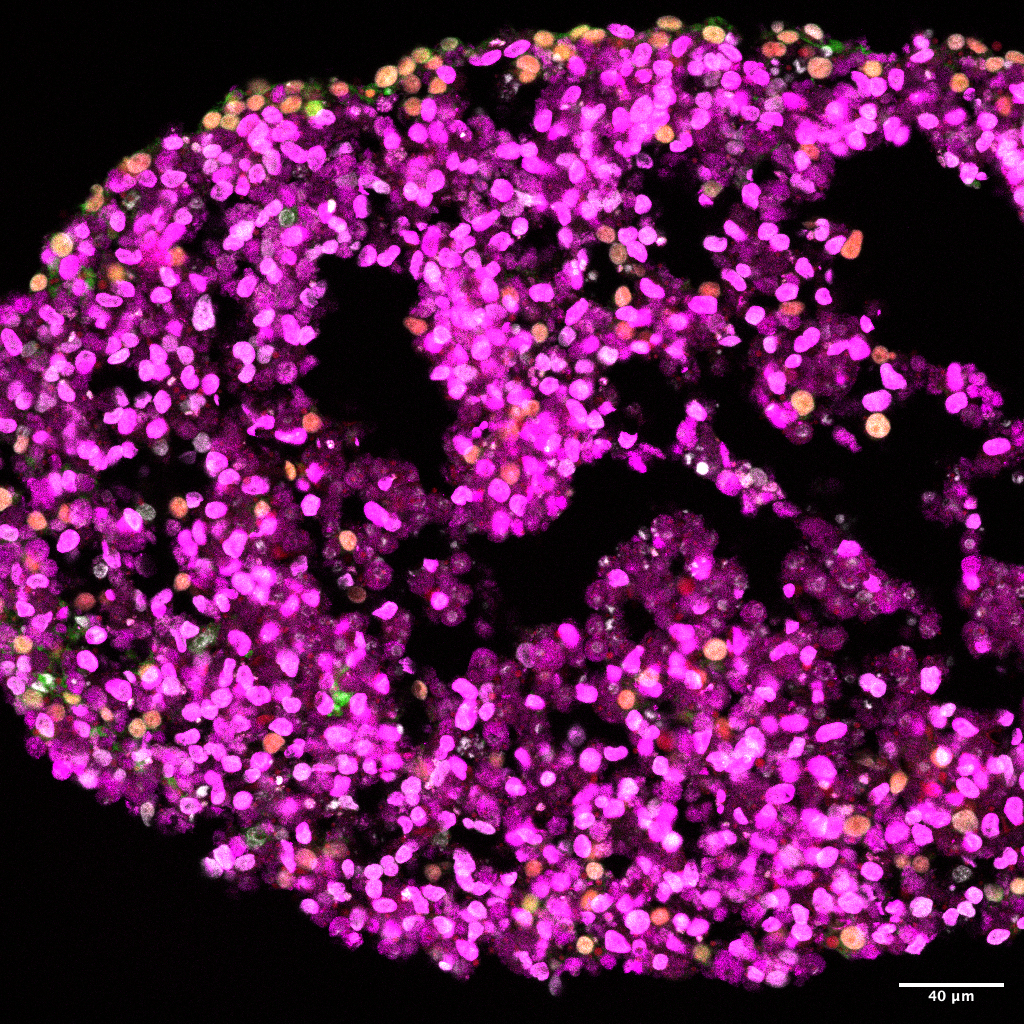

Supplement: Figure 5—source data 2. [file elife-83291-fig5-data2.zip › Figure5_sourcedata_timecourse_F66NR1GF#4/2022-08-24-human_day4_1_composite.png]

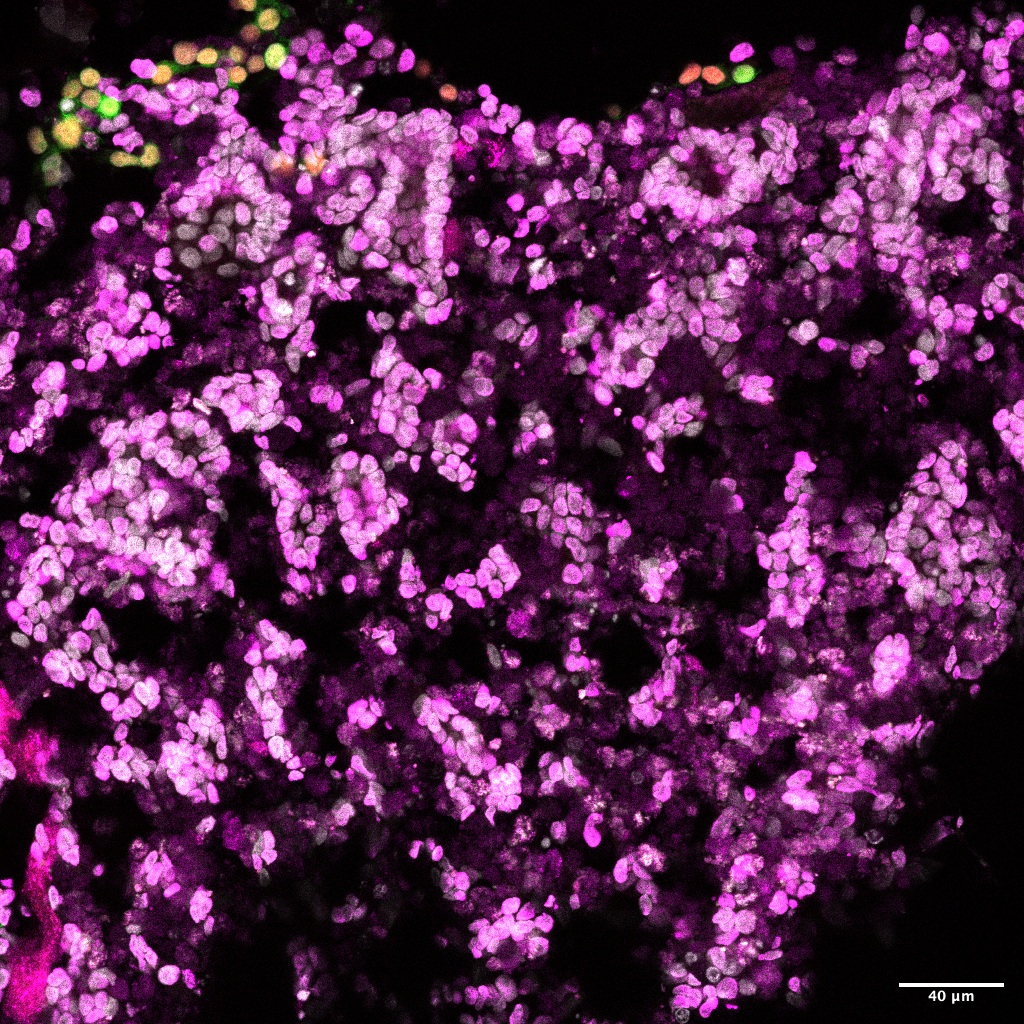

Supplement: Figure 5—source data 2. [file elife-83291-fig5-data2.zip › Figure5_sourcedata_timecourse_F66NR1GF#4/2022-08-24-human_day26_2_composite.png]

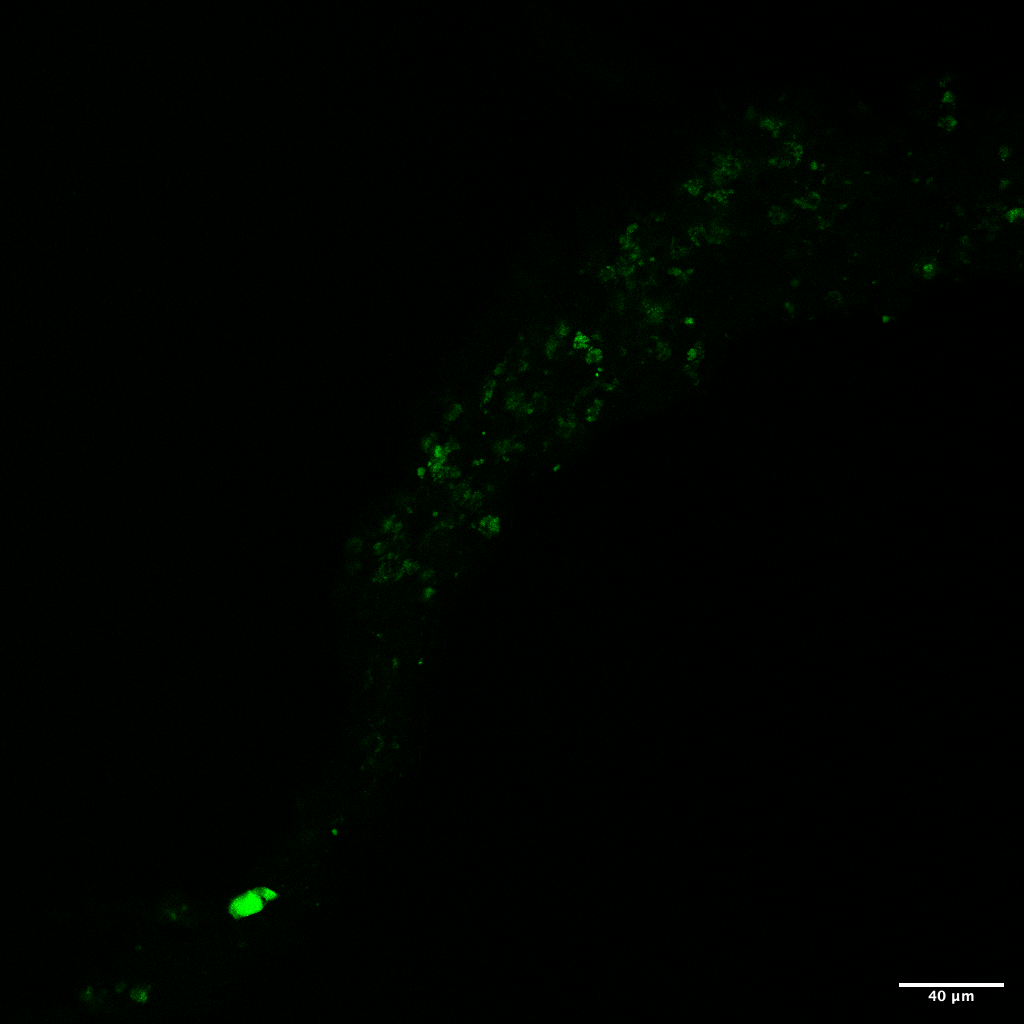

Supplement: Figure 5—source data 2. [file elife-83291-fig5-data2.zip › Figure5_sourcedata_timecourse_F66NR1GF#4/2022-09-12_human_day38_DAZL.png]

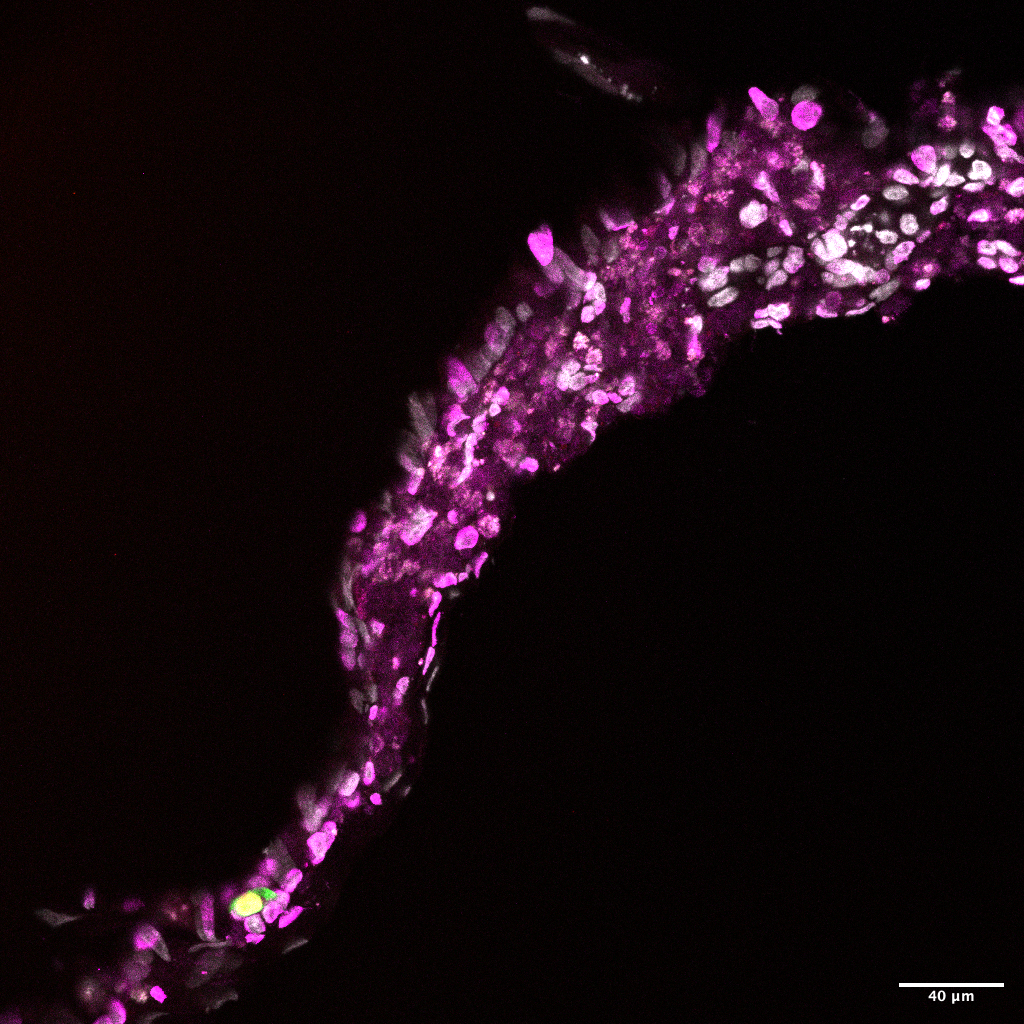

Supplement: Figure 5—source data 2. [file elife-83291-fig5-data2.zip › Figure5_sourcedata_timecourse_F66NR1GF#4/2022-09-12_human_day38_composite.png]

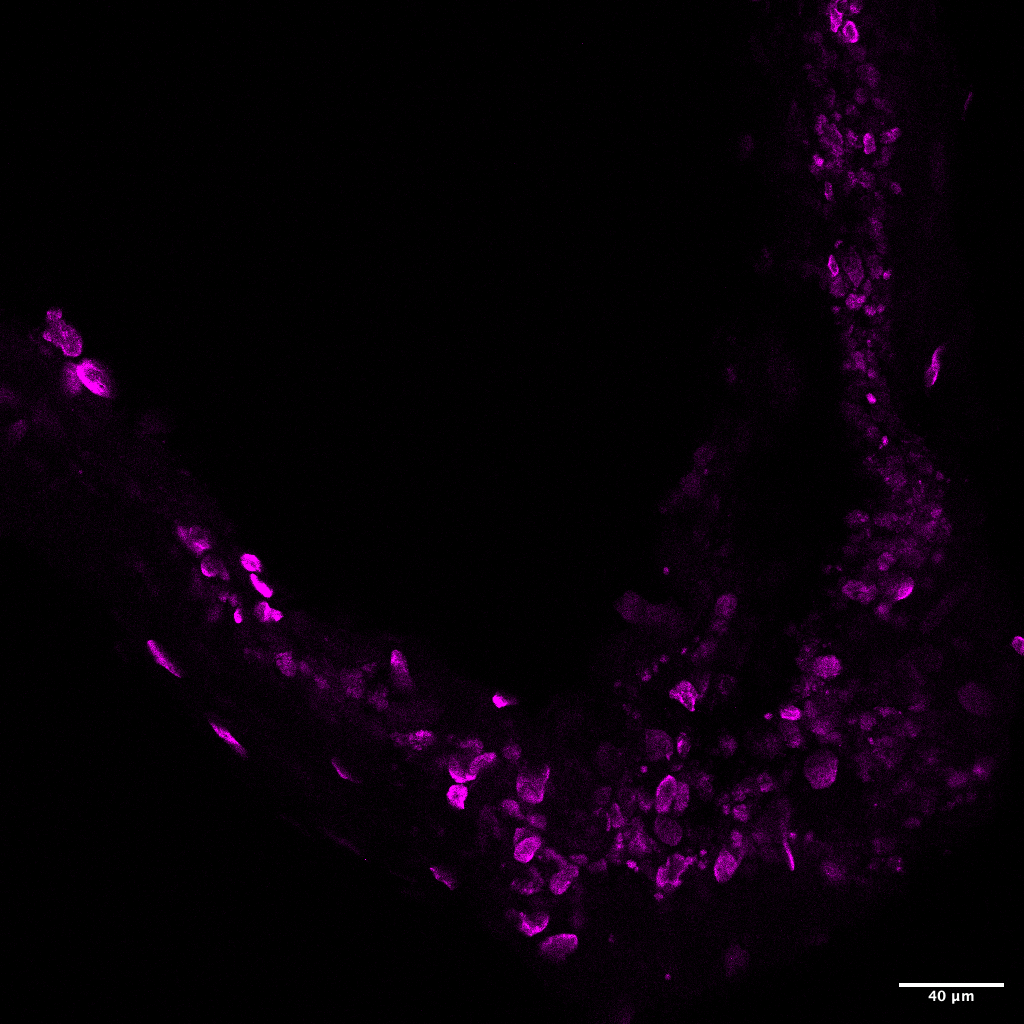

Supplement: Figure 5—source data 2. [file elife-83291-fig5-data2.zip › Figure5_sourcedata_timecourse_F66NR1GF#4/2022-09-12_human_day46_A_FOXL2.png]

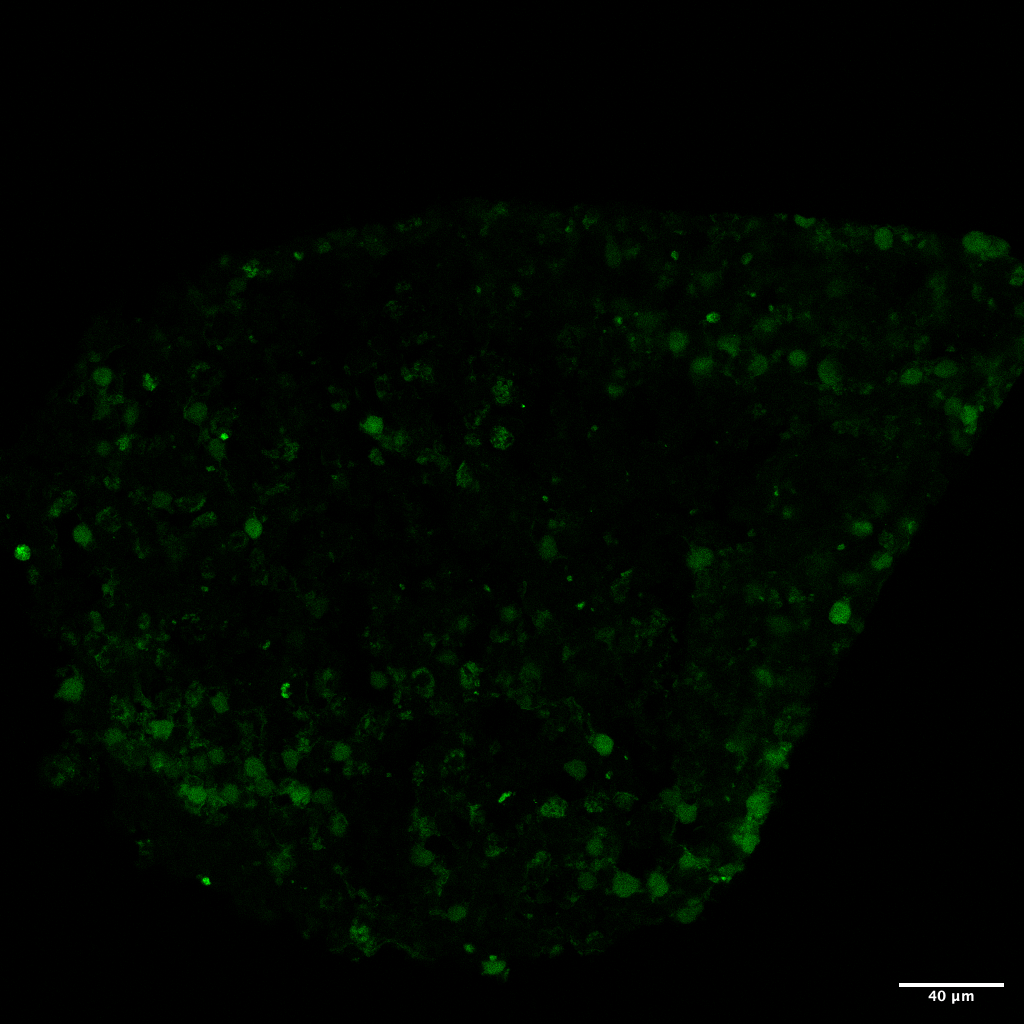

Supplement: Figure 5—source data 2. [file elife-83291-fig5-data2.zip › Figure5_sourcedata_timecourse_F66NR1GF#4/2022-08-24-human_day2_2_DAZL.png]

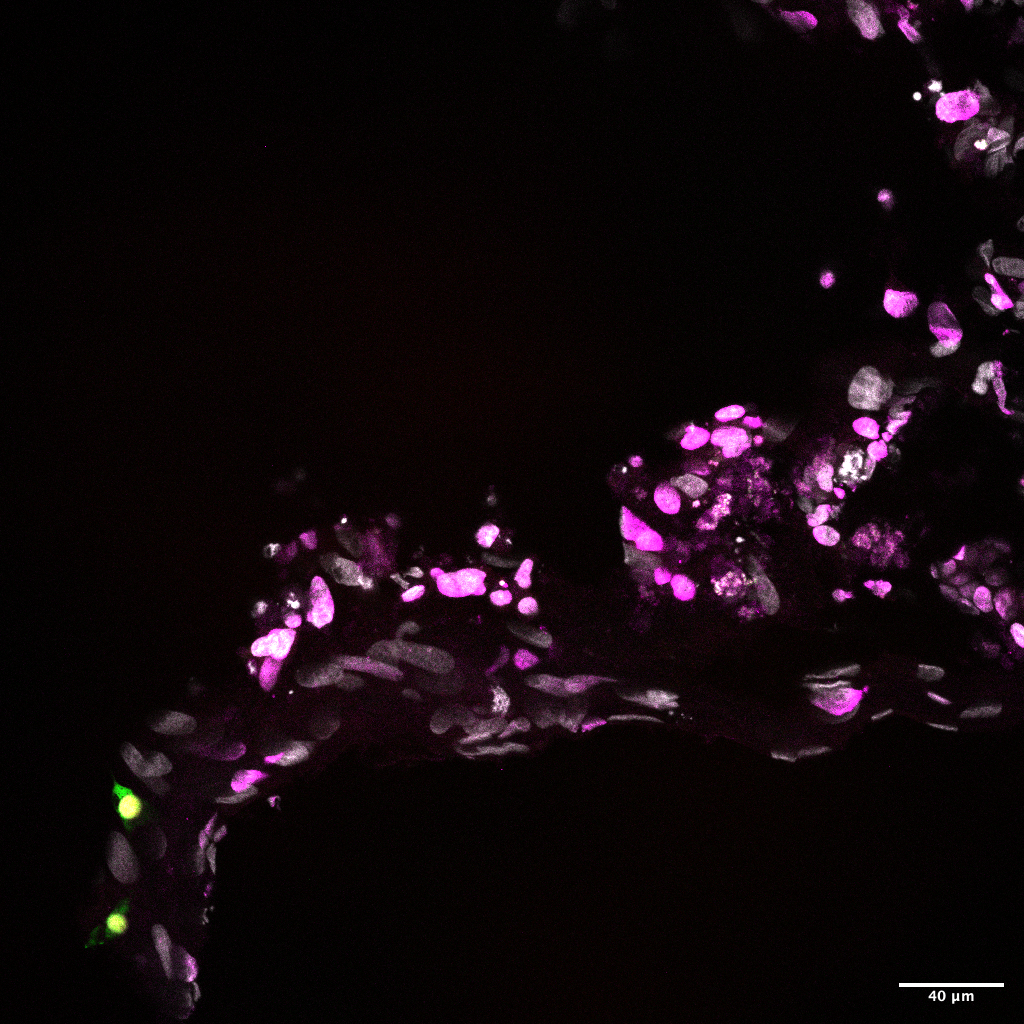

Supplement: Figure 5—source data 2. [file elife-83291-fig5-data2.zip › Figure5_sourcedata_timecourse_F66NR1GF#4/2022-09-12_human_day54_1_composite.png]

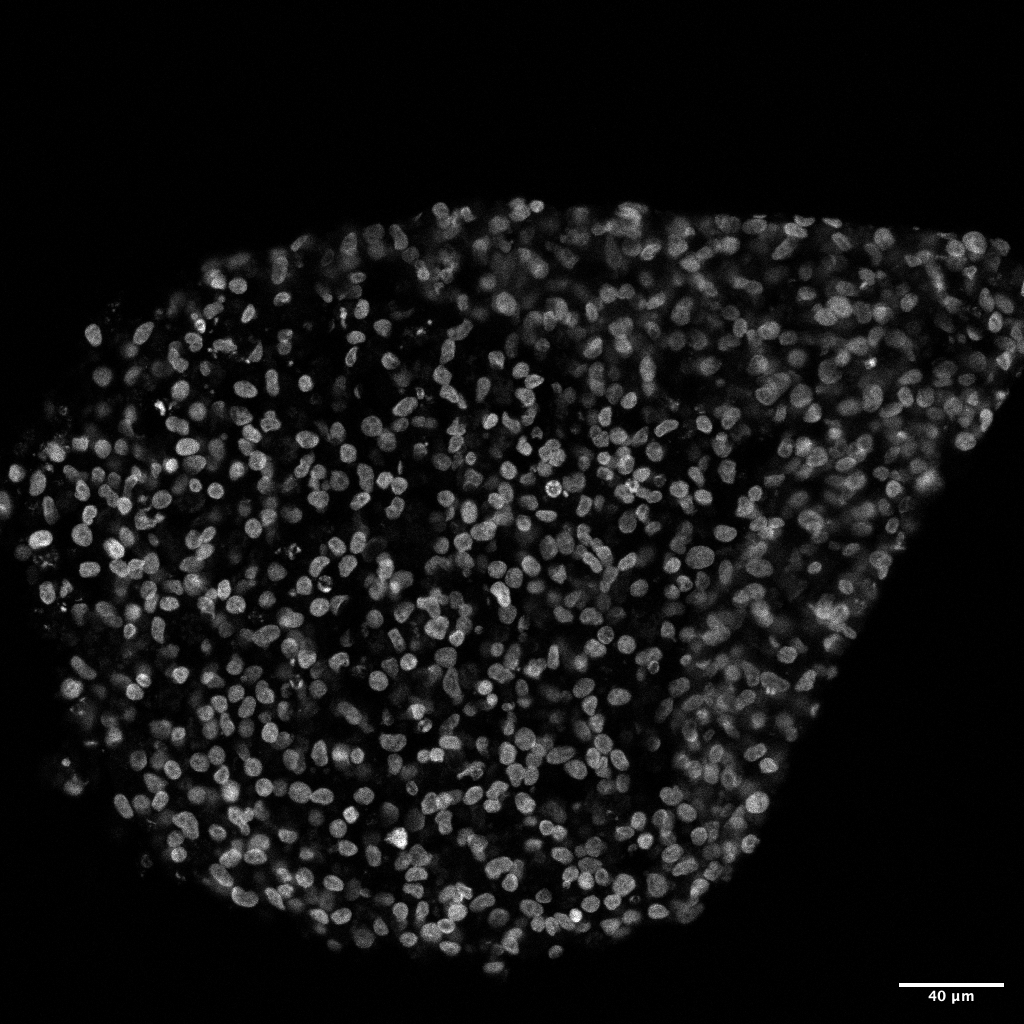

Supplement: Figure 5—source data 2. [file elife-83291-fig5-data2.zip › Figure5_sourcedata_timecourse_F66NR1GF#4/2022-08-24-human_day2_DAPI.png]

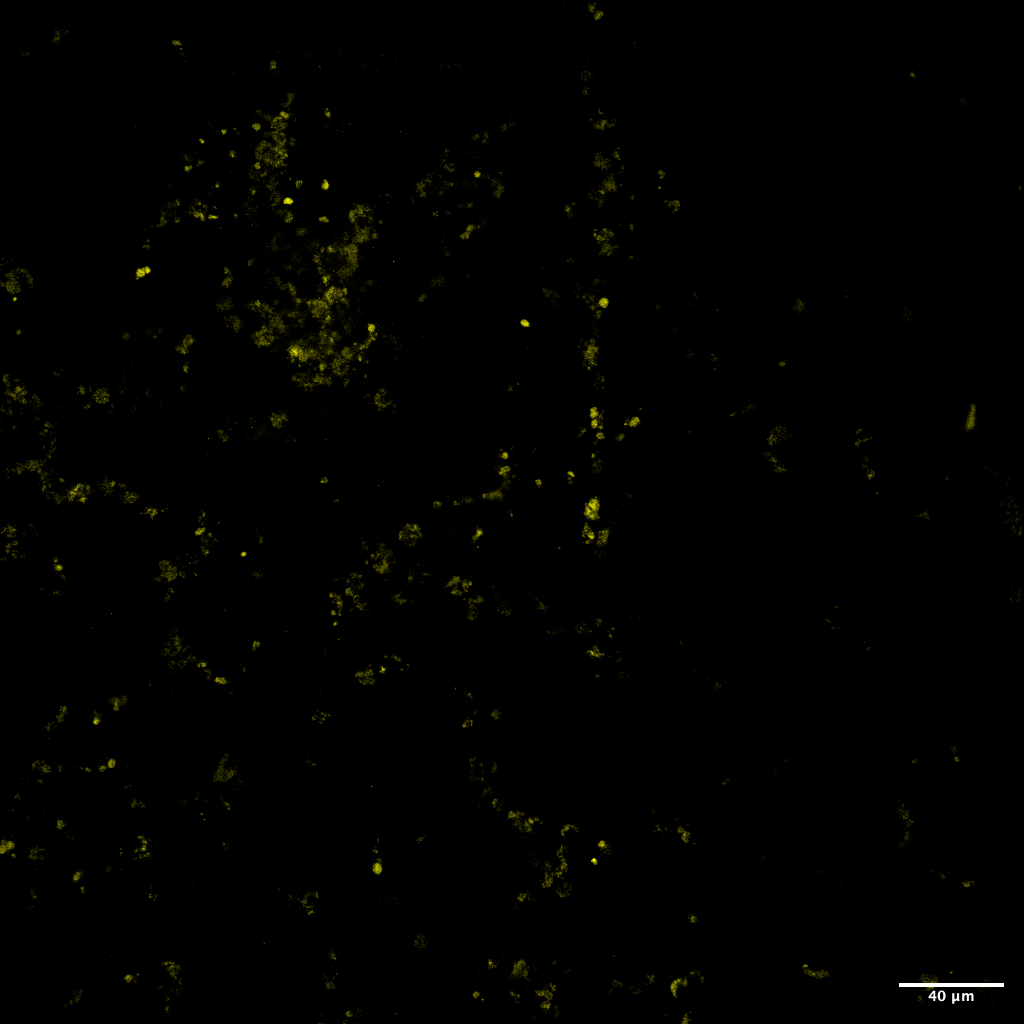

Supplement: Figure 5—source data 2. [file elife-83291-fig5-data2.zip › Figure5_sourcedata_timecourse_F66NR1GF#4/Day70_DAZL.png]

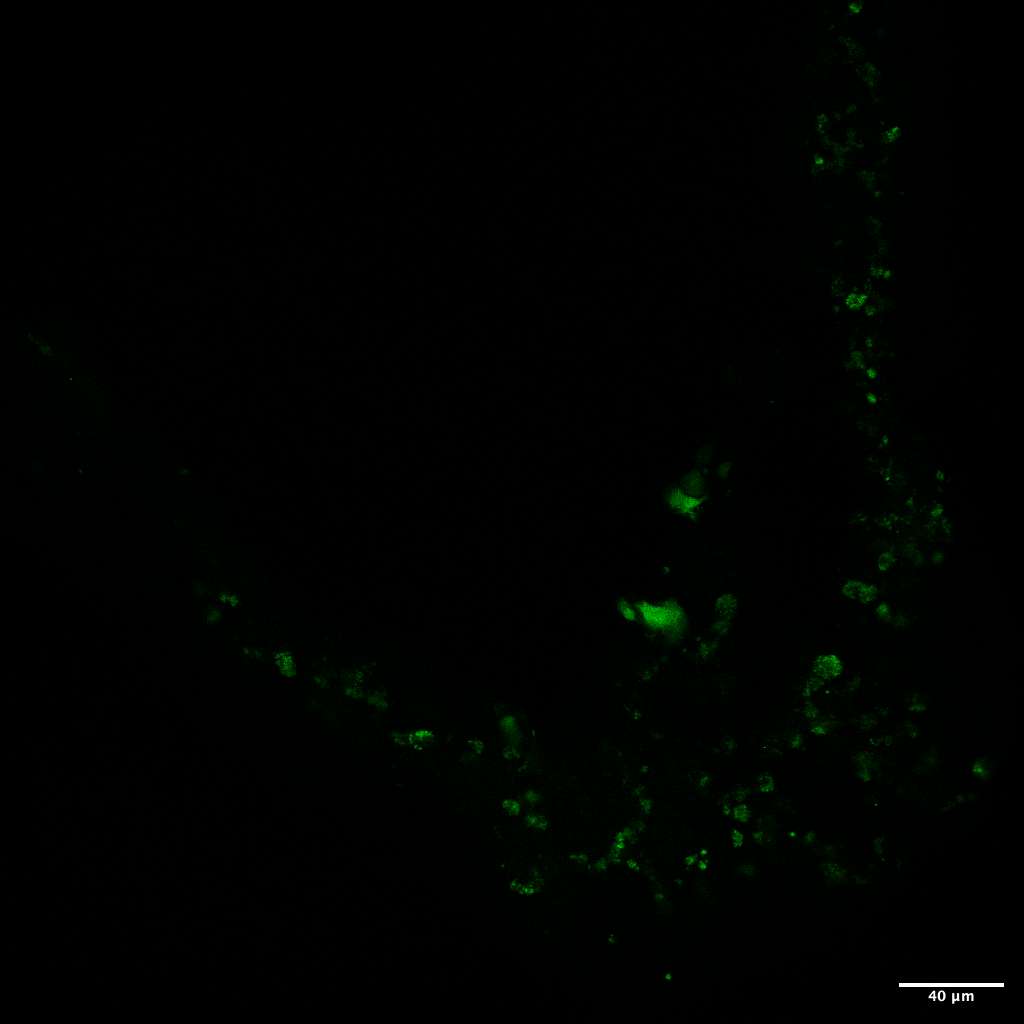

Supplement: Figure 5—source data 2. [file elife-83291-fig5-data2.zip › Figure5_sourcedata_timecourse_F66NR1GF#4/2022-09-12_human_day46_A_DAZL.png]

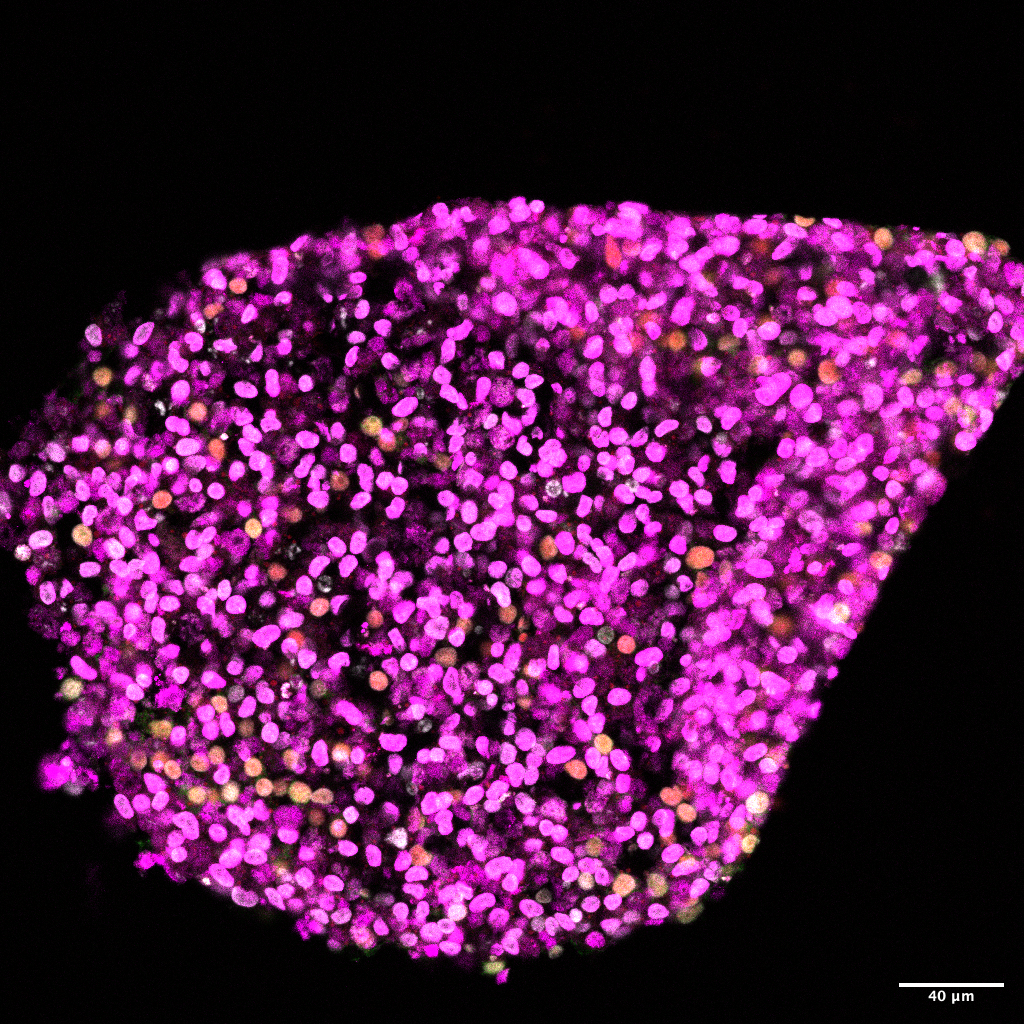

Supplement: Figure 5—source data 2. [file elife-83291-fig5-data2.zip › Figure5_sourcedata_timecourse_F66NR1GF#4/2022-08-24-human_day2_2_composite.png]

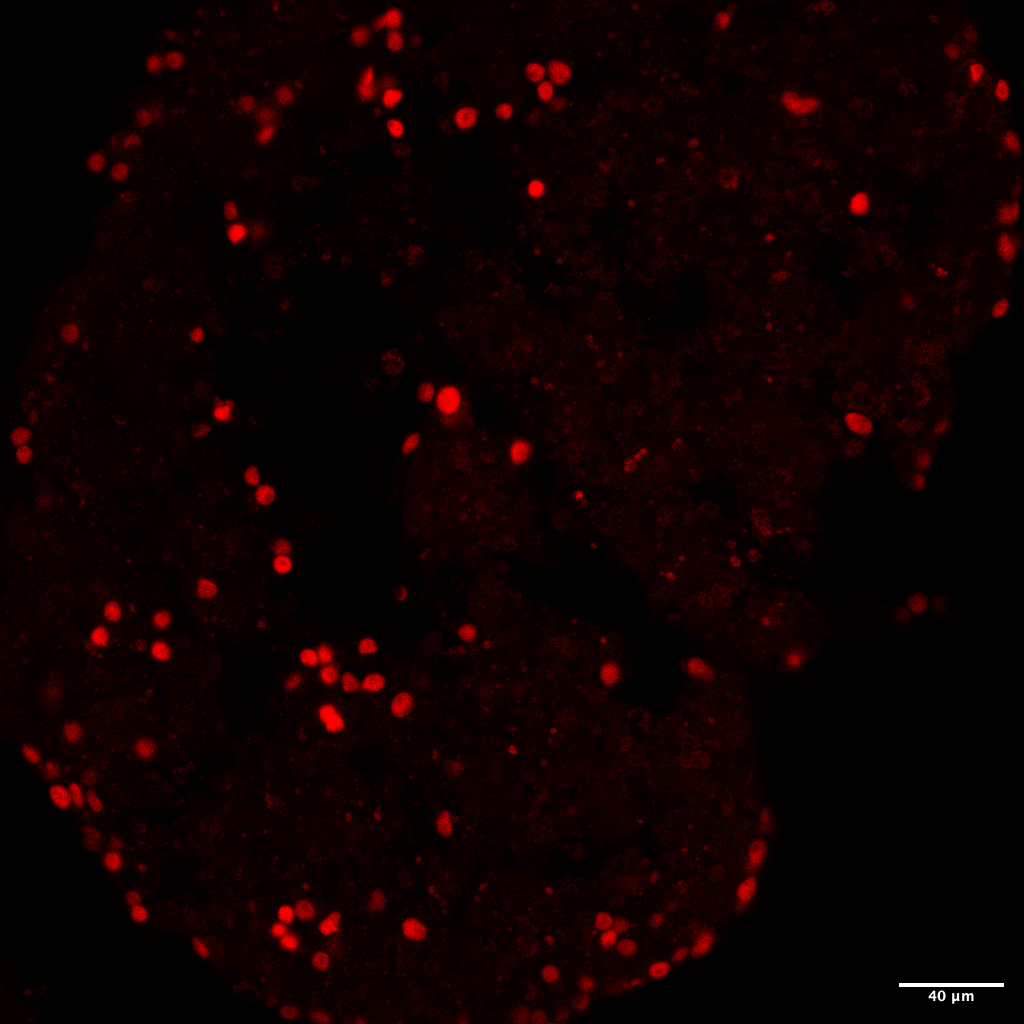

Supplement: Figure 5—source data 2. [file elife-83291-fig5-data2.zip › Figure5_sourcedata_timecourse_F66NR1GF#4/2022-08-23_human_day14_3_OCT4.png]

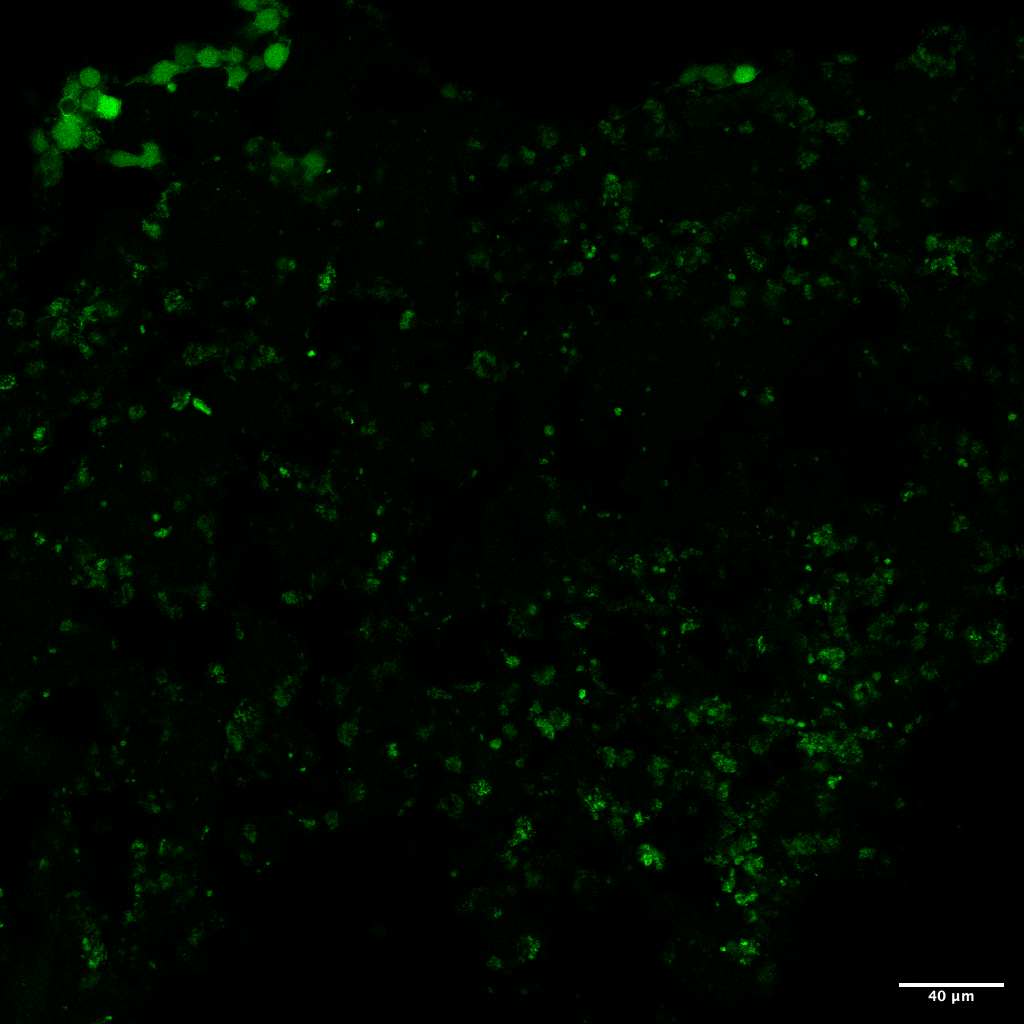

Supplement: Figure 5—source data 2. [file elife-83291-fig5-data2.zip › Figure5_sourcedata_timecourse_F66NR1GF#4/2022-08-24-human_day26_2_DAZL.png]

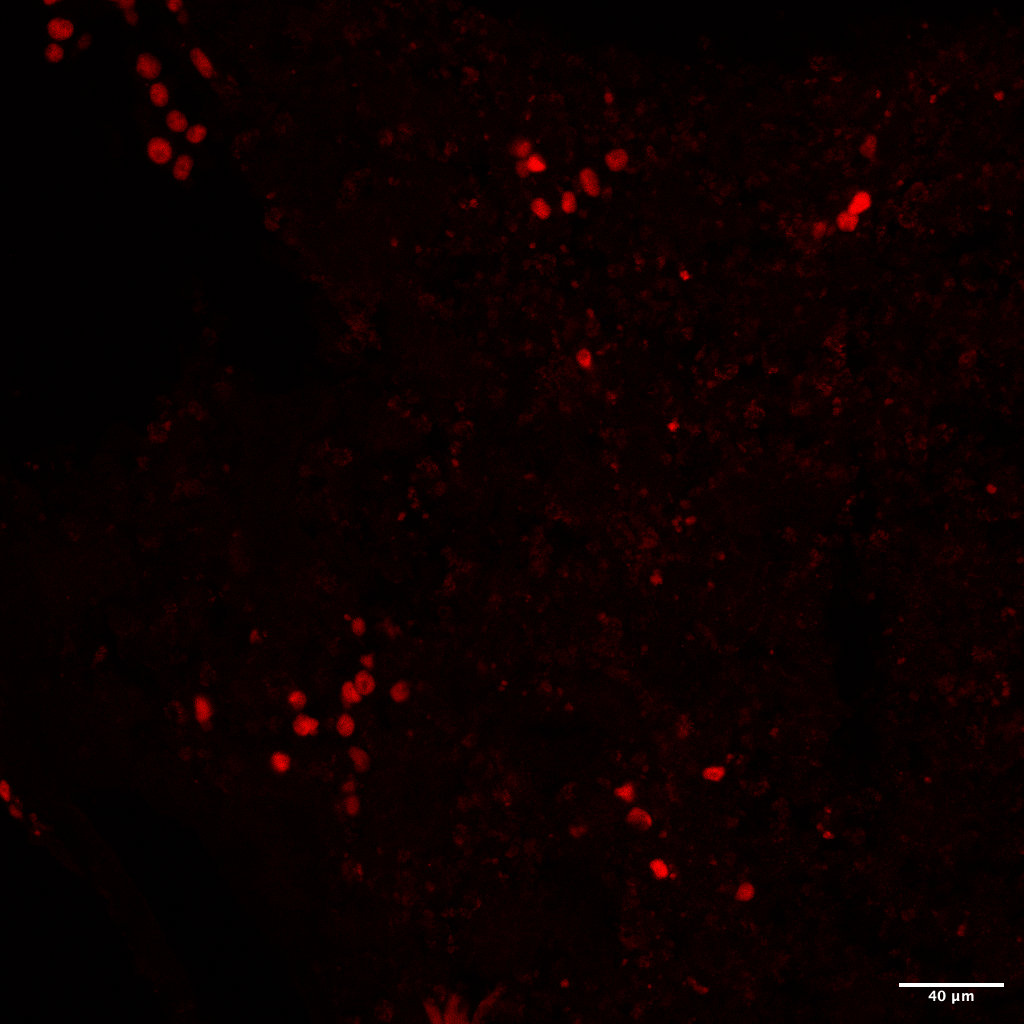

Supplement: Figure 5—source data 2. [file elife-83291-fig5-data2.zip › Figure5_sourcedata_timecourse_F66NR1GF#4/2022-08-25-human_day32_1_OCT4.png]

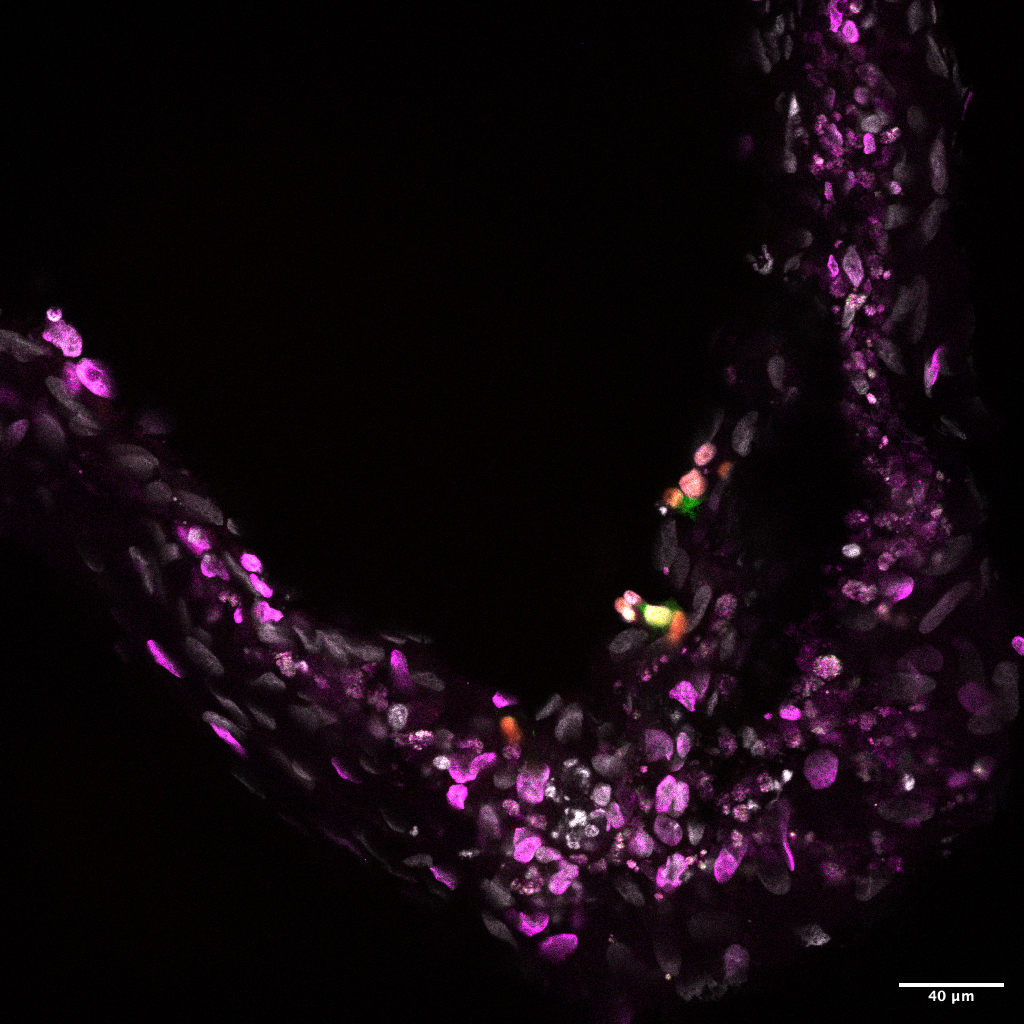

Supplement: Figure 5—source data 2. [file elife-83291-fig5-data2.zip › Figure5_sourcedata_timecourse_F66NR1GF#4/2022-09-12_human_day46_A_composite.png]

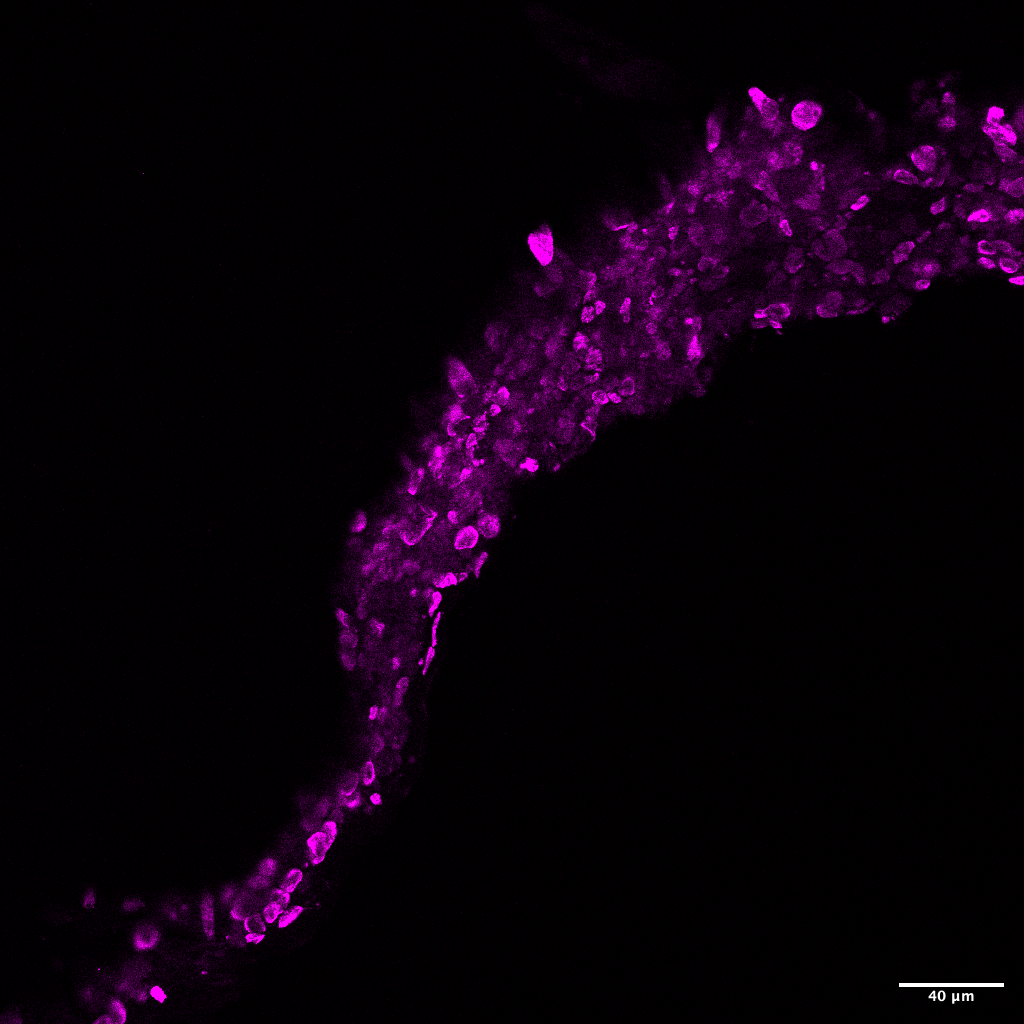

Supplement: Figure 5—source data 2. [file elife-83291-fig5-data2.zip › Figure5_sourcedata_timecourse_F66NR1GF#4/2022-09-12_human_day38_FOXL2.png]

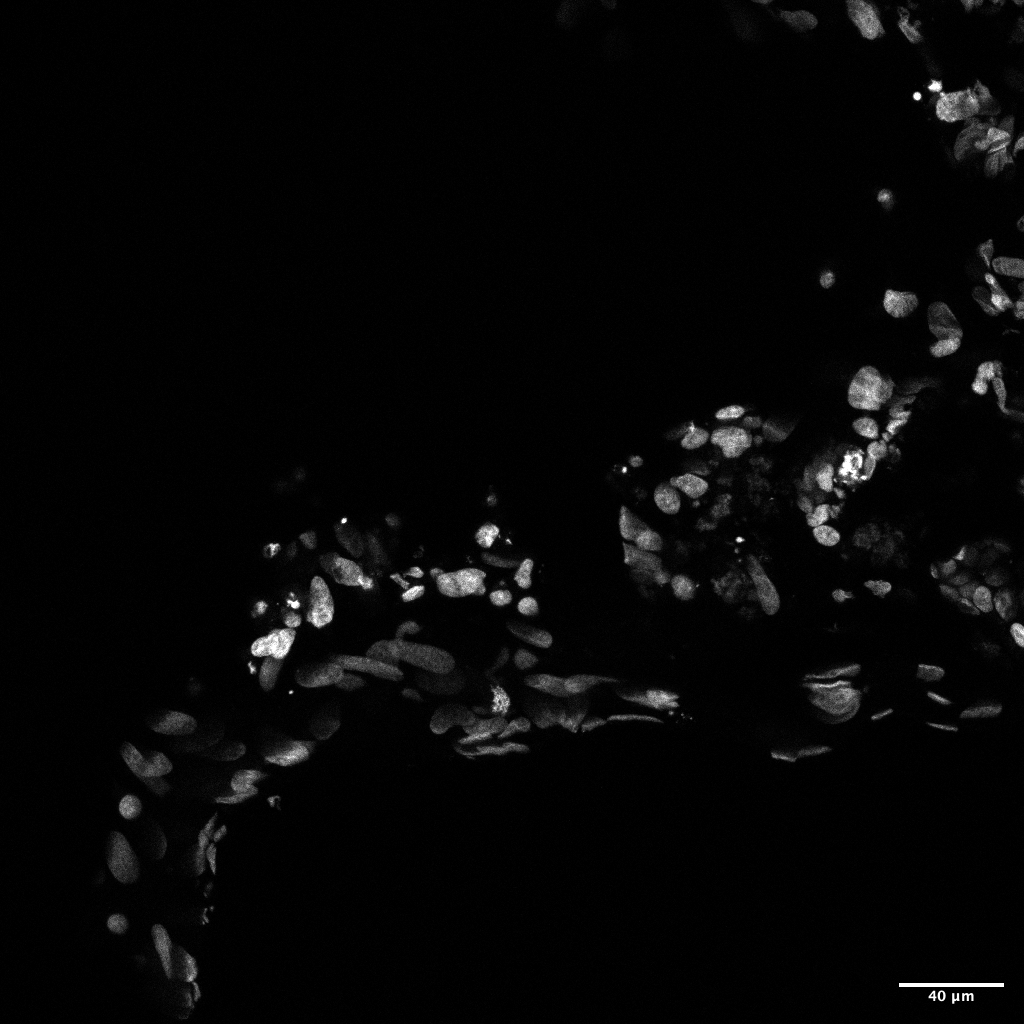

Supplement: Figure 5—source data 2. [file elife-83291-fig5-data2.zip › Figure5_sourcedata_timecourse_F66NR1GF#4/2022-09-12_human_day54_DAPI.png]

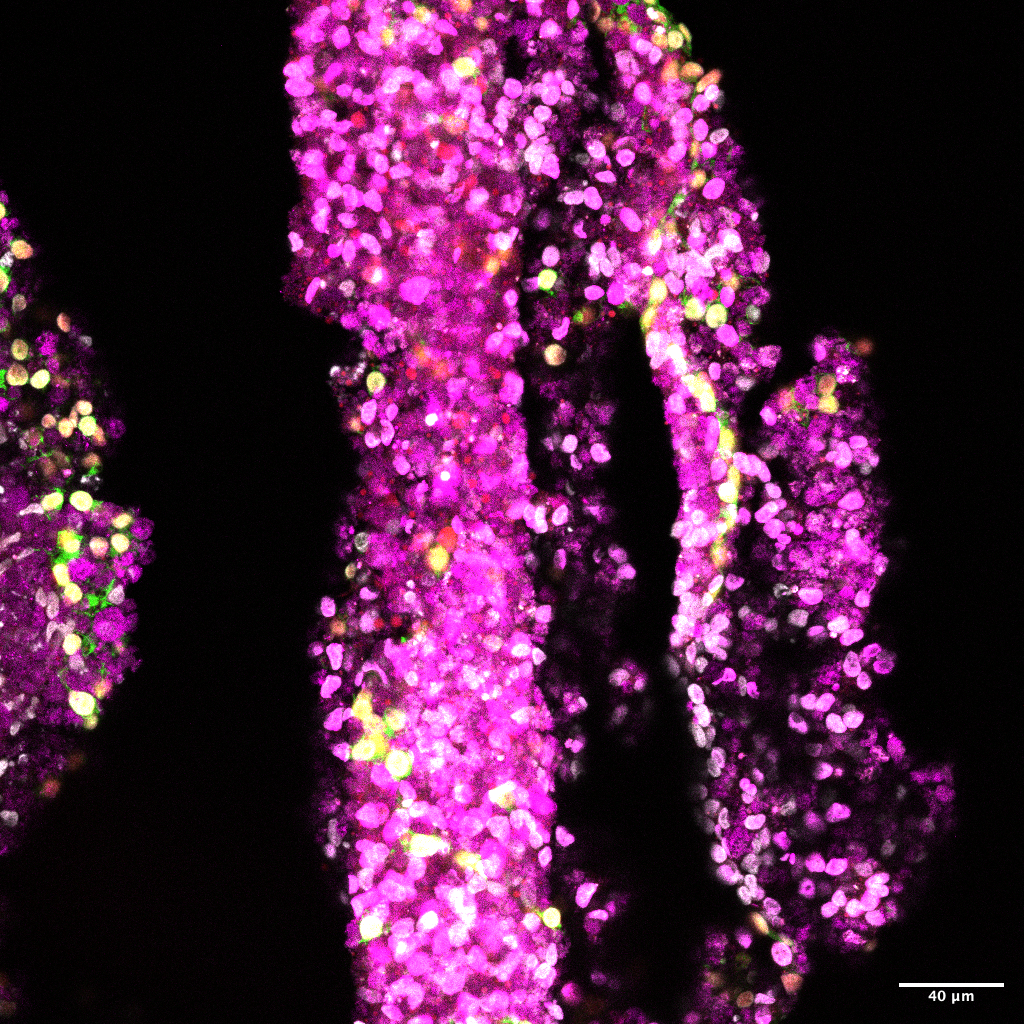

Supplement: Figure 5—source data 2. [file elife-83291-fig5-data2.zip › Figure5_sourcedata_timecourse_F66NR1GF#4/2022-08-23_day8_stainA.lif - overlay.png]

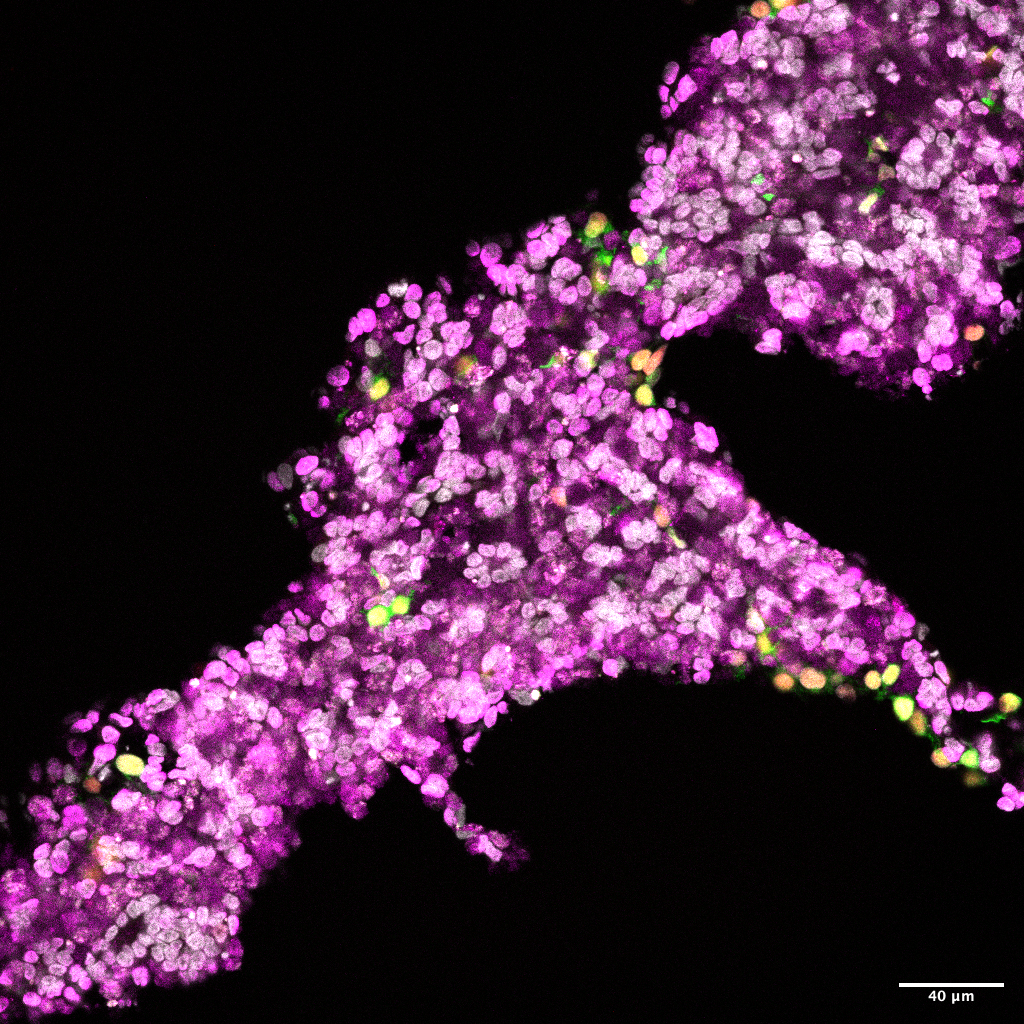

Supplement: Figure 5—source data 2. [file elife-83291-fig5-data2.zip › Figure5_sourcedata_timecourse_F66NR1GF#4/2022-08-25_human_day20_2b_composite.png]

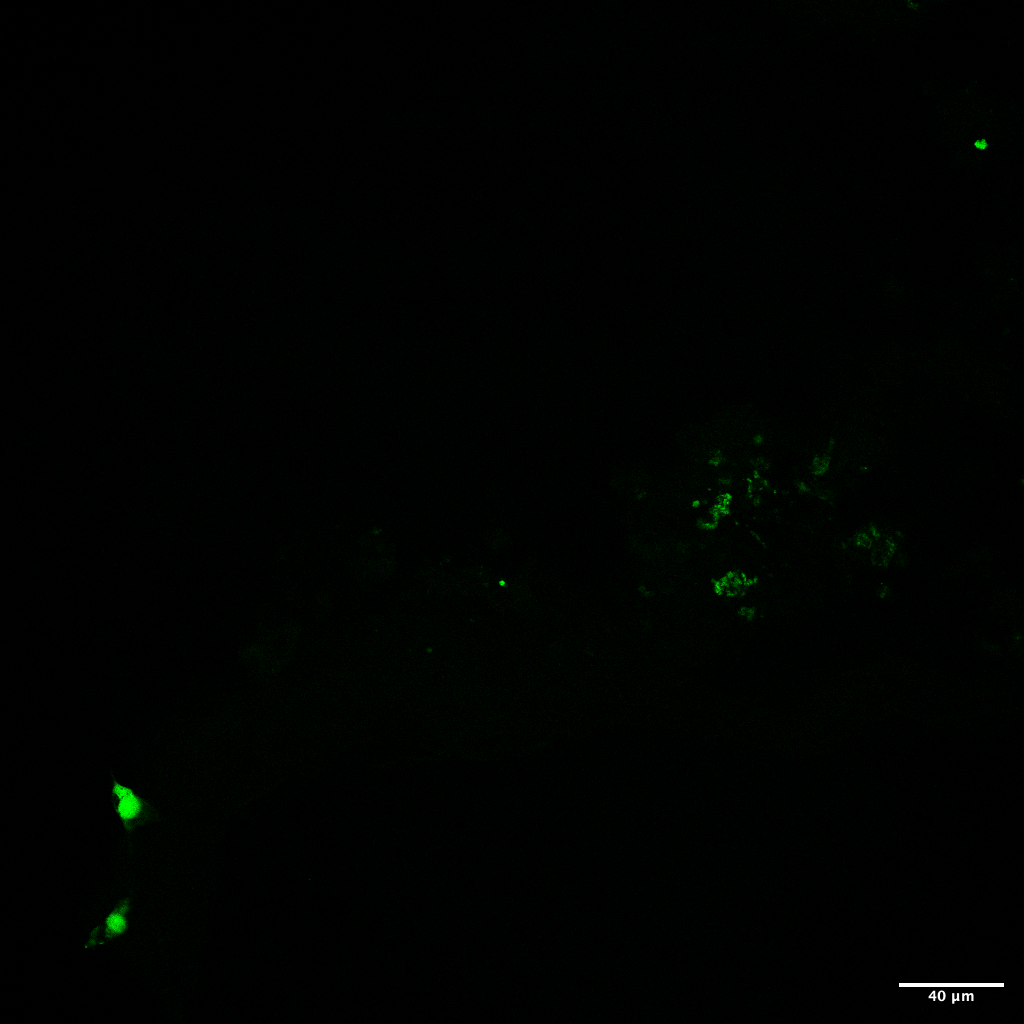

Supplement: Figure 5—source data 2. [file elife-83291-fig5-data2.zip › Figure5_sourcedata_timecourse_F66NR1GF#4/2022-09-12_human_day54_1_DAZL.png]

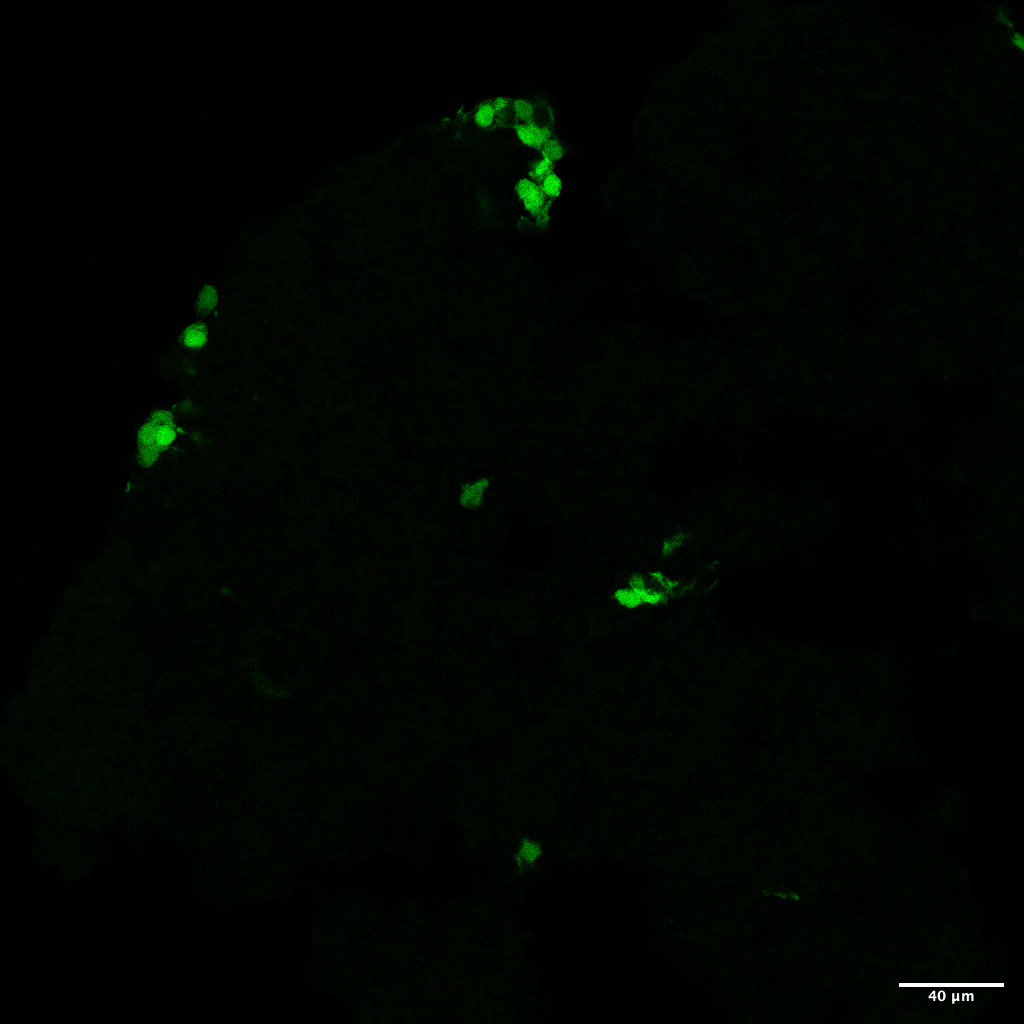

Supplement: Figure 5—source data 3. [file elife-83291-fig5-data3.zip › Figure5_sourcedata_timecourse_F66NR2#1/Day8DAZL.png]

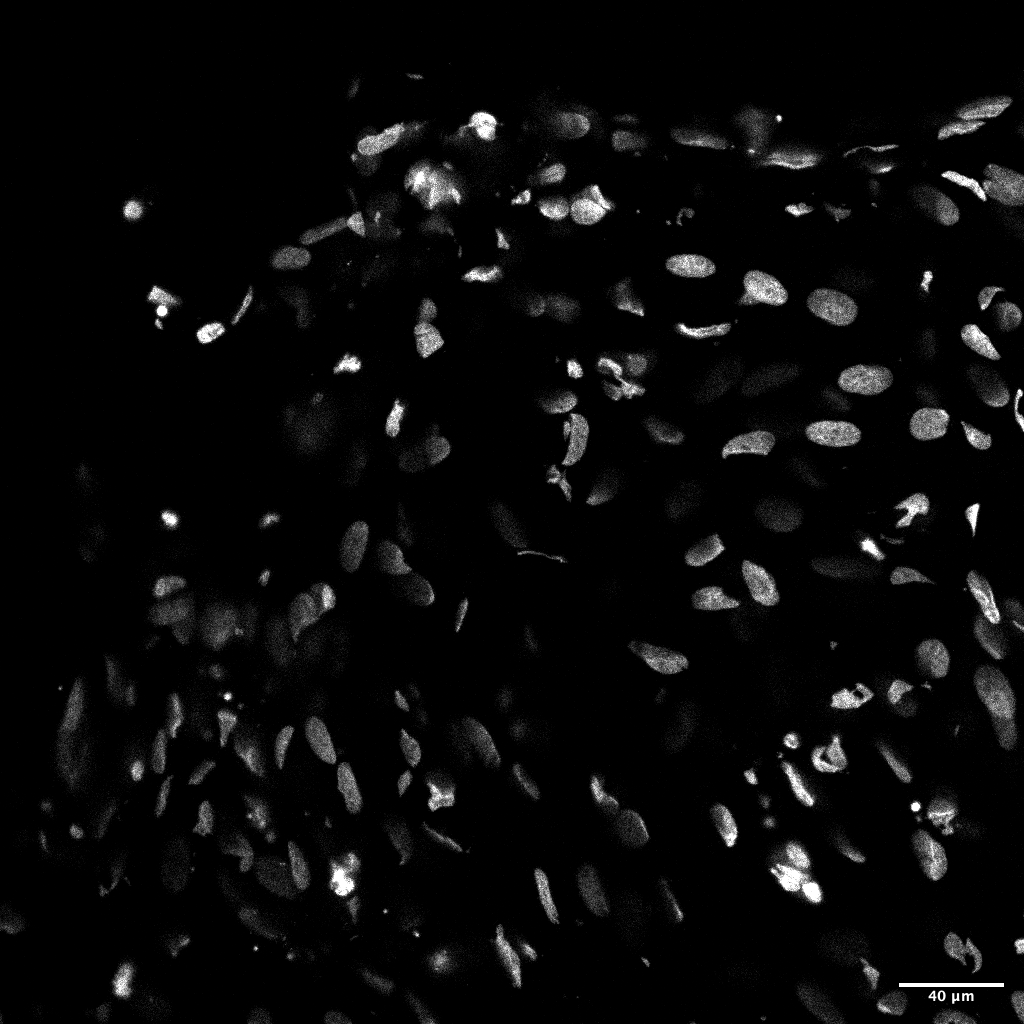

Supplement: Figure 5—source data 3. [file elife-83291-fig5-data3.zip › Figure5_sourcedata_timecourse_F66NR2#1/Day46_DAPI.png]

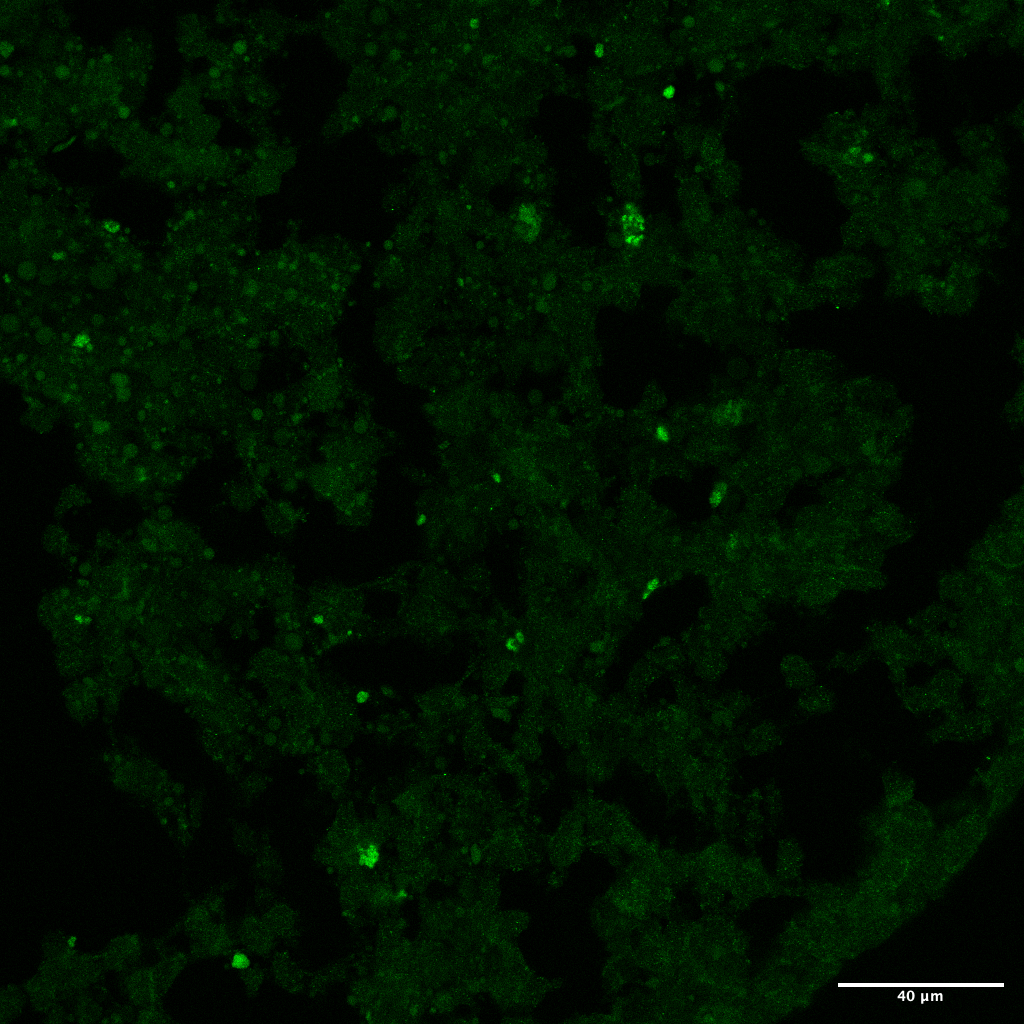

Supplement: Figure 5—source data 3. [file elife-83291-fig5-data3.zip › Figure5_sourcedata_timecourse_F66NR2#1/Day2-_A1_DAZL.png]

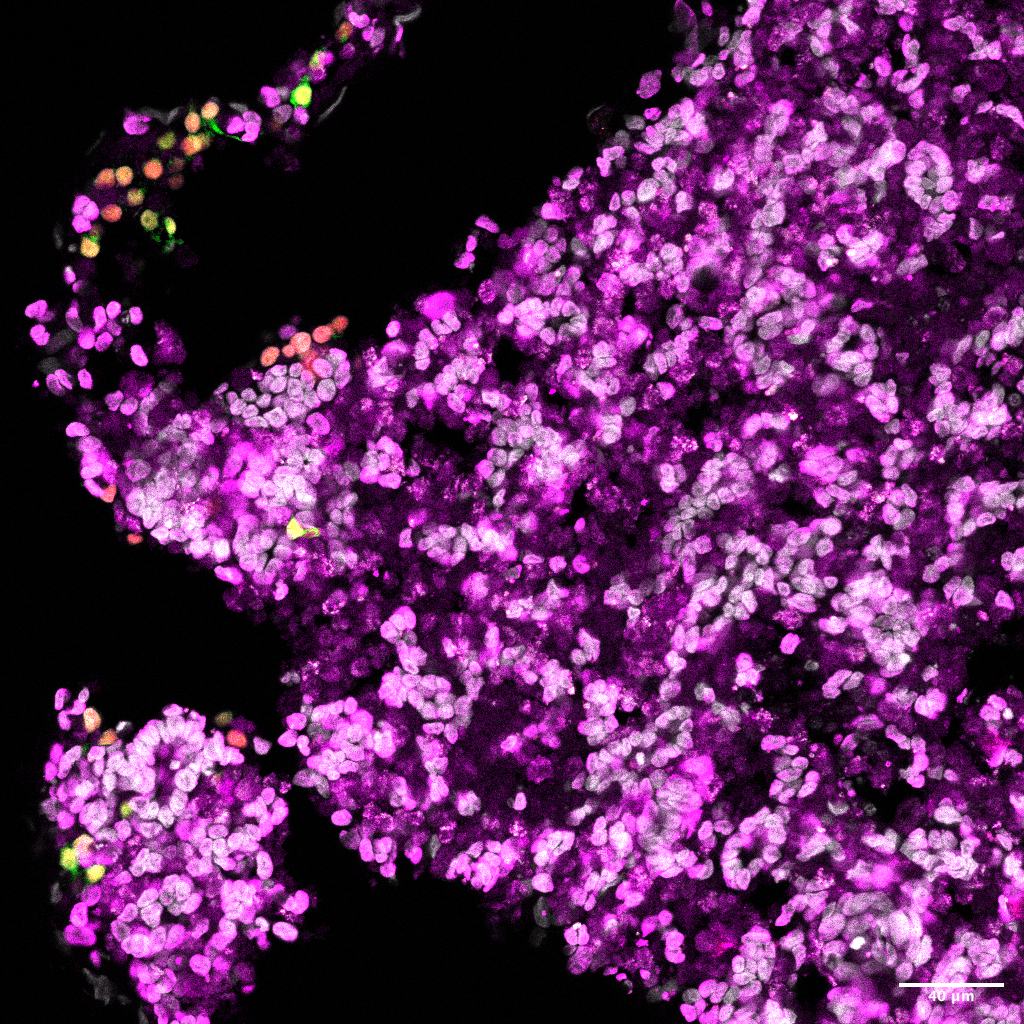

Supplement: Figure 5—source data 3. [file elife-83291-fig5-data3.zip › Figure5_sourcedata_timecourse_F66NR2#1/Day26_overlay.png]

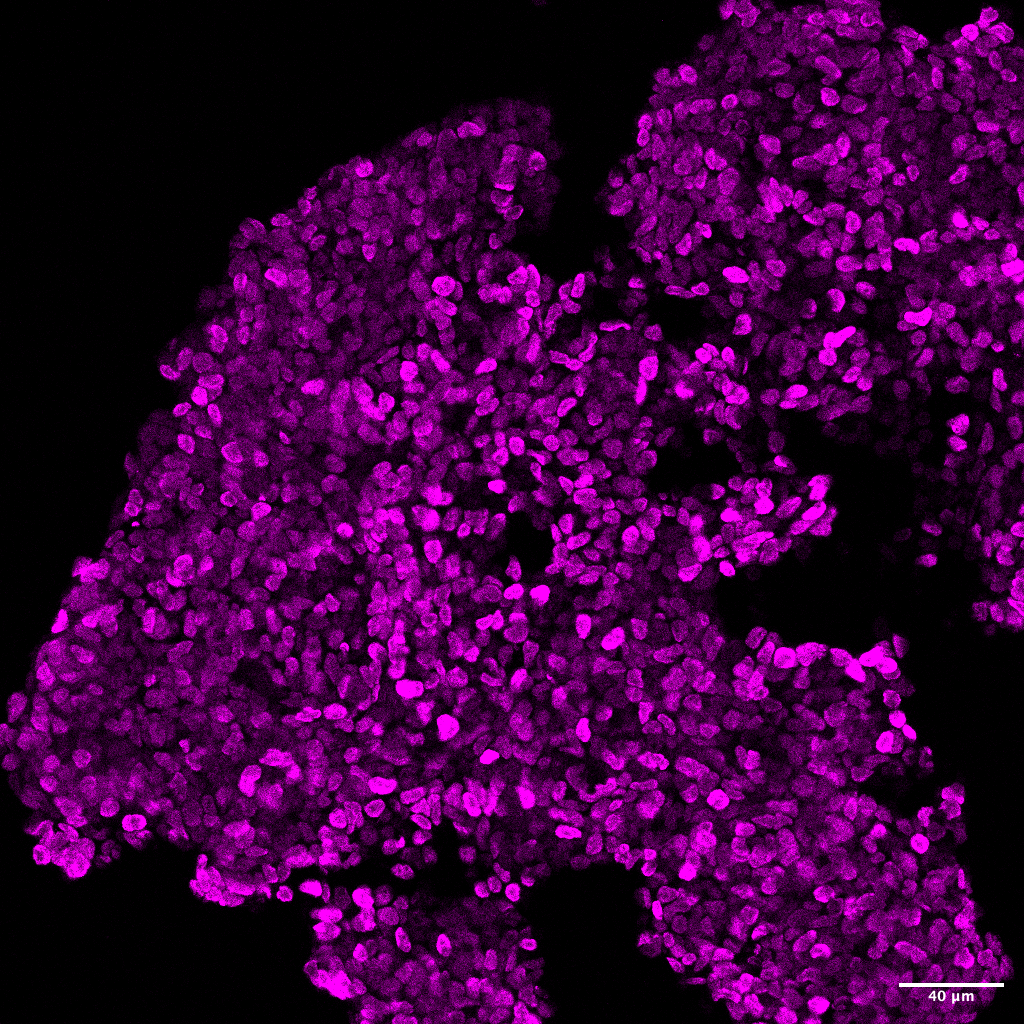

Supplement: Figure 5—source data 3. [file elife-83291-fig5-data3.zip › Figure5_sourcedata_timecourse_F66NR2#1/Day8FOXL2.png]

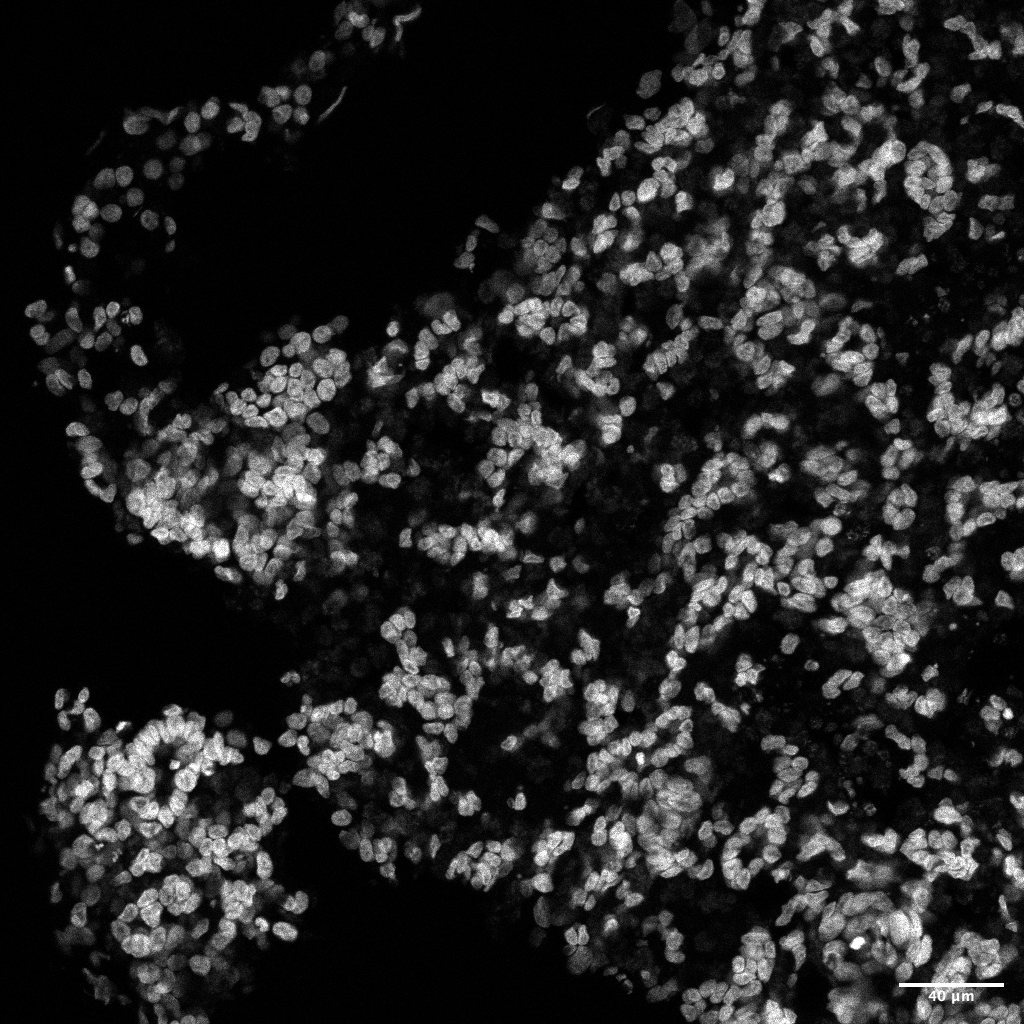

Supplement: Figure 5—source data 3. [file elife-83291-fig5-data3.zip › Figure5_sourcedata_timecourse_F66NR2#1/Day26_DAPI.png]

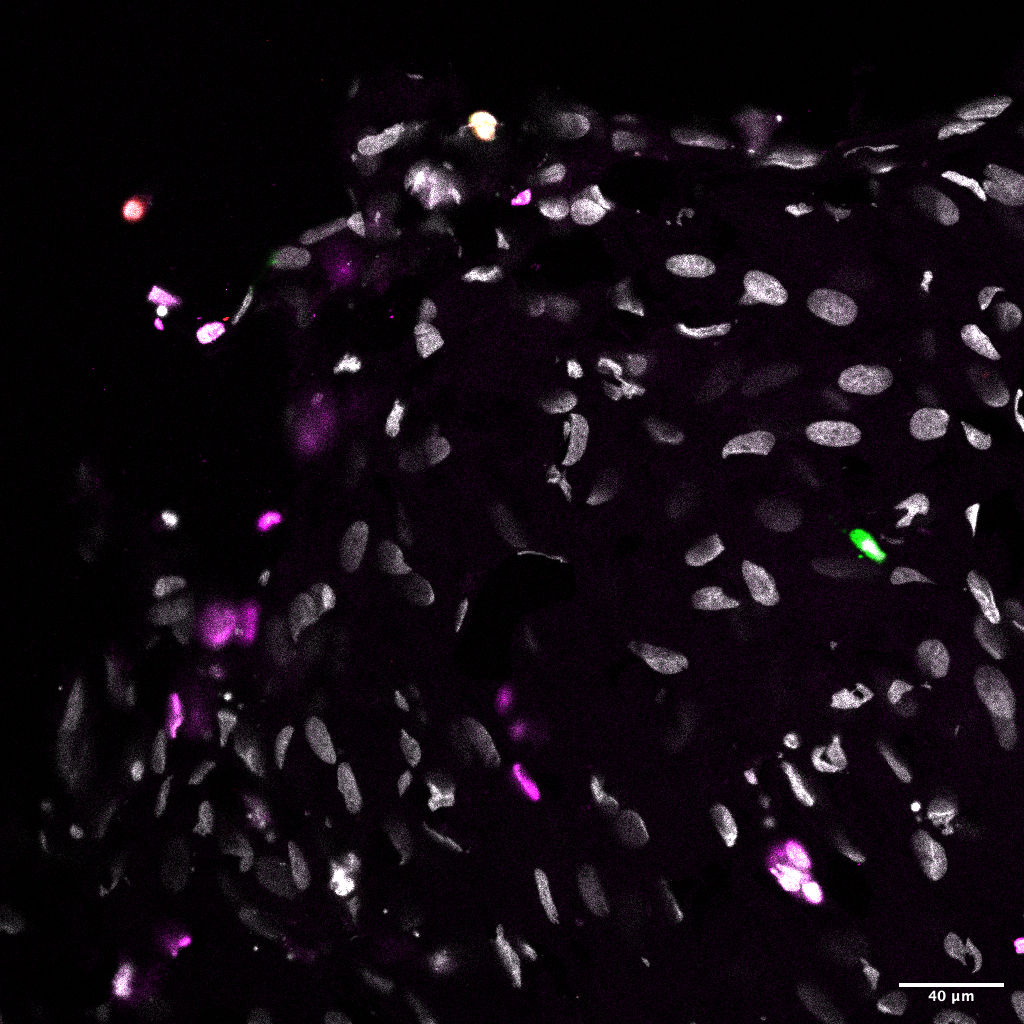

Supplement: Figure 5—source data 3. [file elife-83291-fig5-data3.zip › Figure5_sourcedata_timecourse_F66NR2#1/Day46_overlay.png]

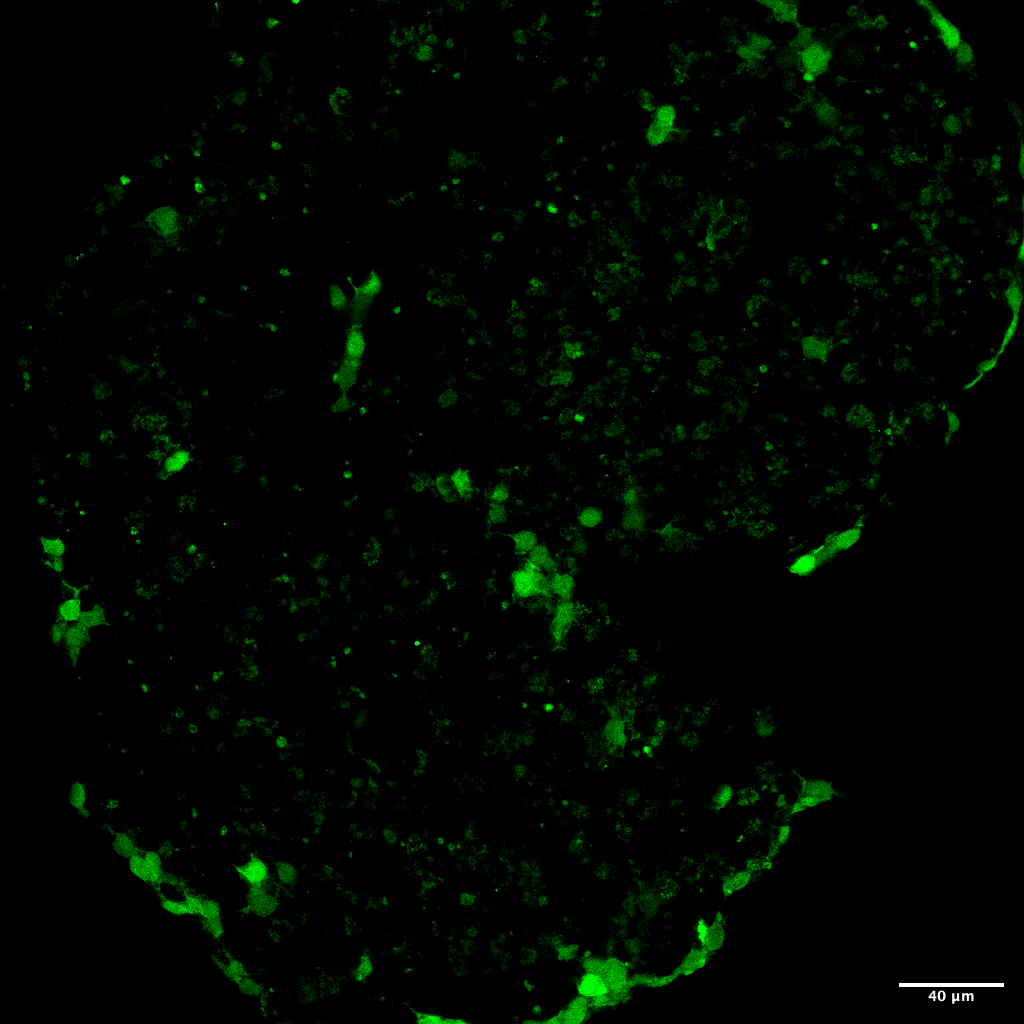

Supplement: Figure 5—source data 3. [file elife-83291-fig5-data3.zip › Figure5_sourcedata_timecourse_F66NR2#1/Day14_DAZL.png]

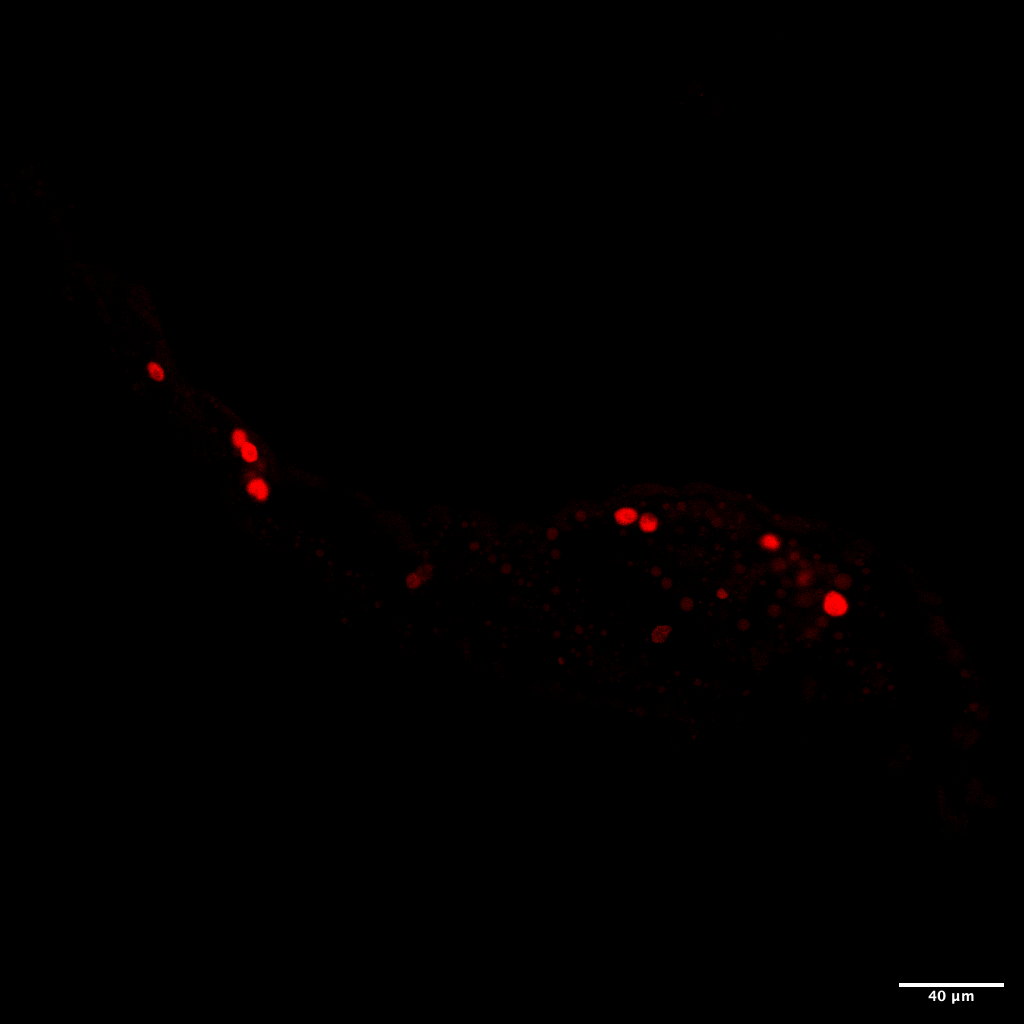

Supplement: Figure 5—source data 3. [file elife-83291-fig5-data3.zip › Figure5_sourcedata_timecourse_F66NR2#1/Day32_OCT4.png]

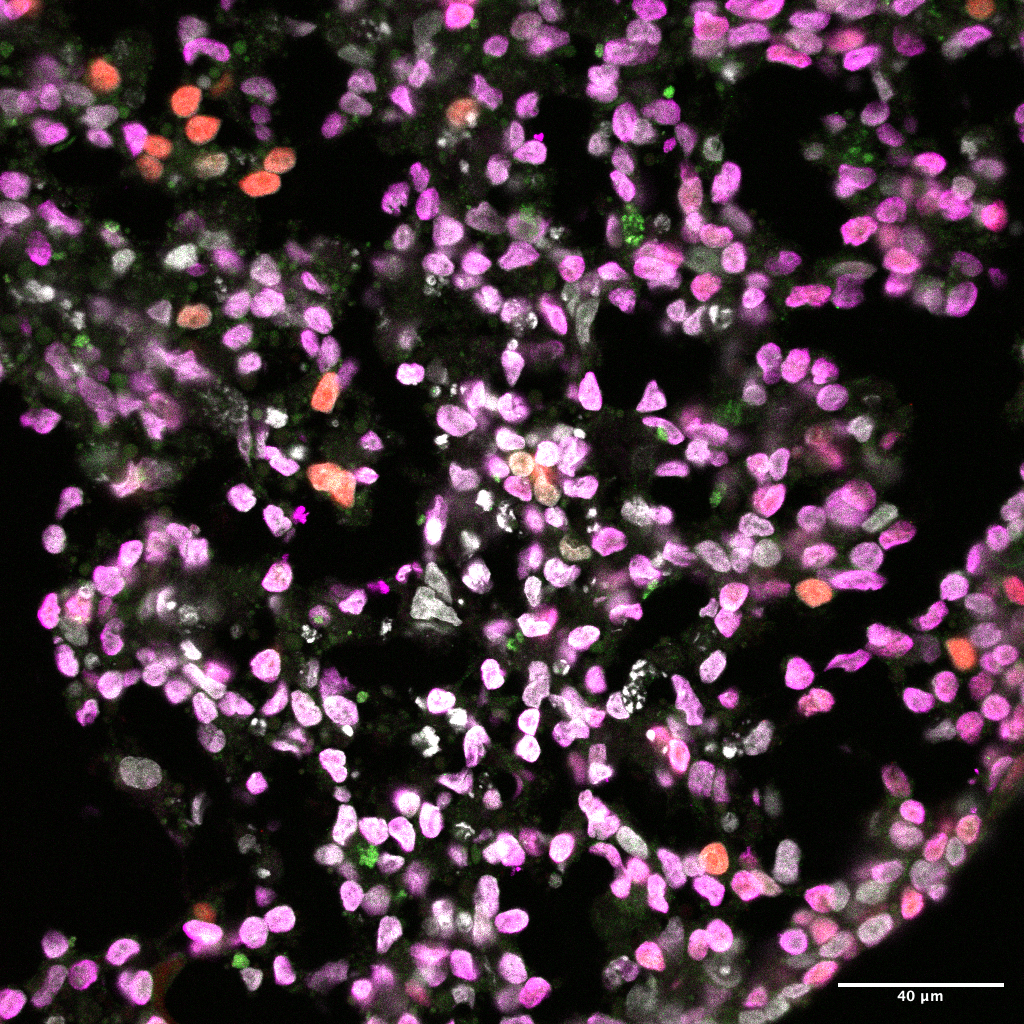

Supplement: Figure 5—source data 3. [file elife-83291-fig5-data3.zip › Figure5_sourcedata_timecourse_F66NR2#1/Day2-_A1_comp.png]

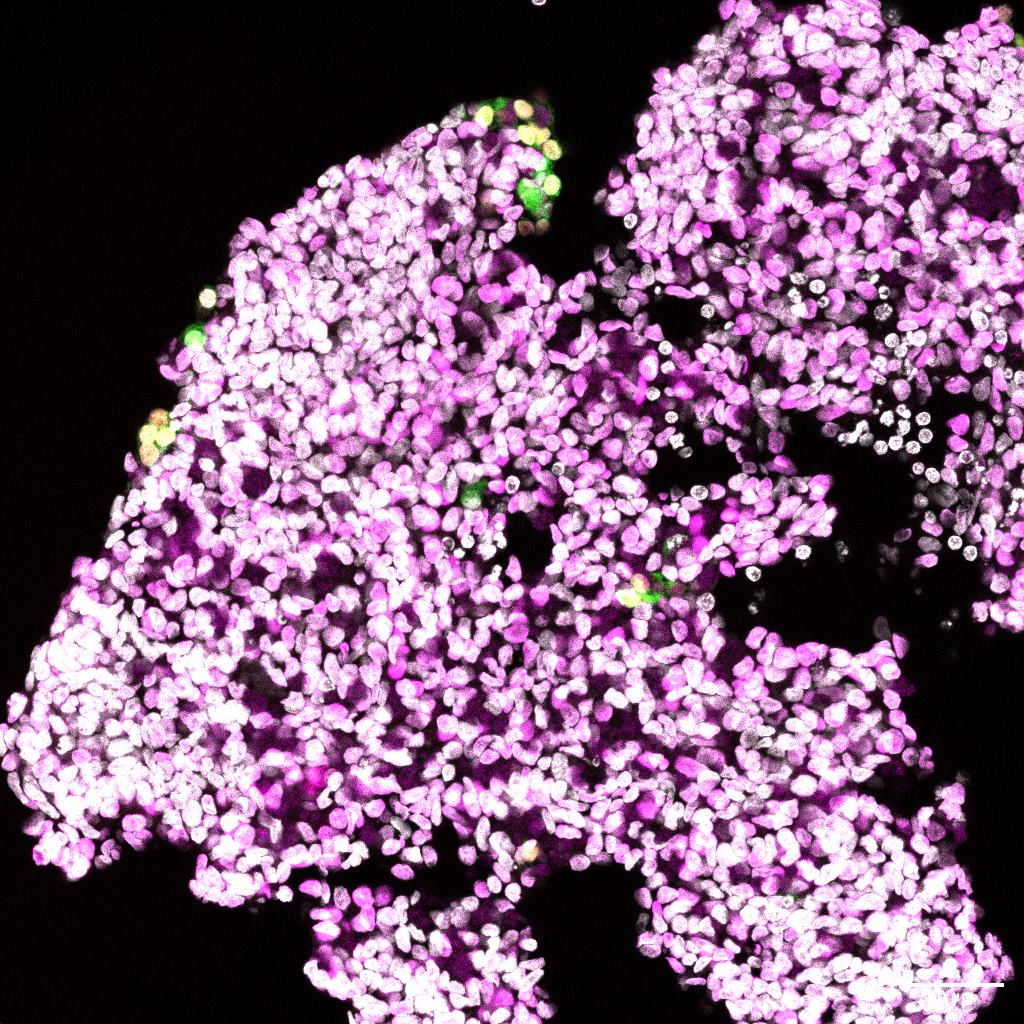

Supplement: Figure 5—source data 3. [file elife-83291-fig5-data3.zip › Figure5_sourcedata_timecourse_F66NR2#1/Day8comp.png]

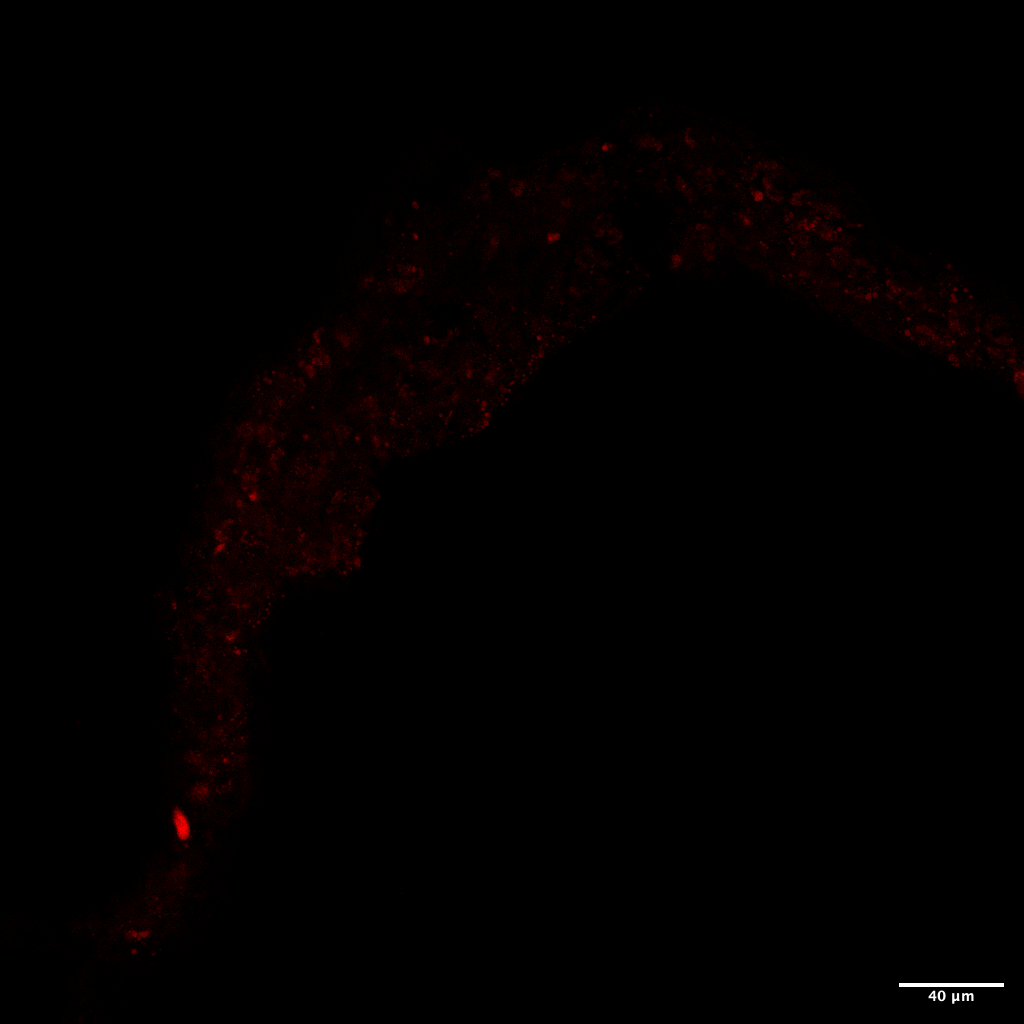

Supplement: Figure 5—source data 3. [file elife-83291-fig5-data3.zip › Figure5_sourcedata_timecourse_F66NR2#1/Day38_OCT4.png]

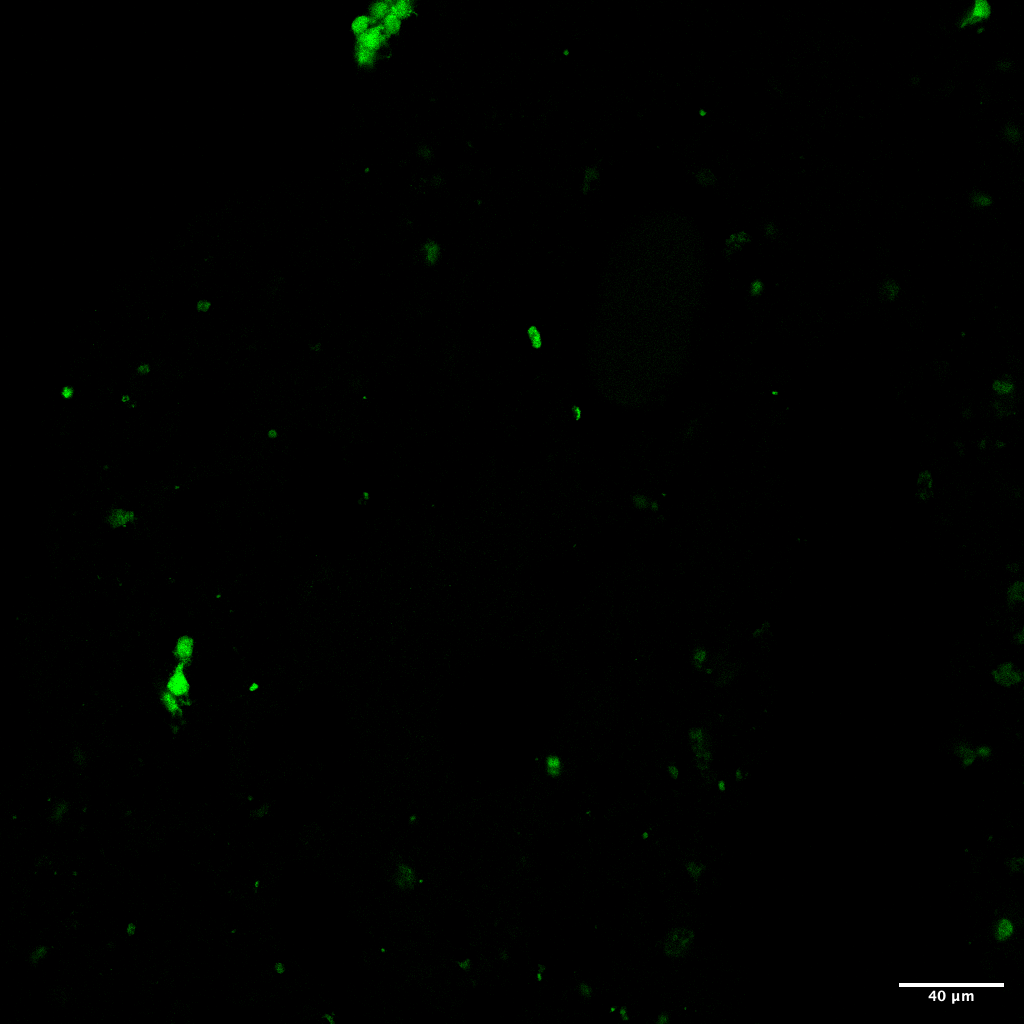

Supplement: Figure 5—source data 3. [file elife-83291-fig5-data3.zip › Figure5_sourcedata_timecourse_F66NR2#1/Day20_3_DAZL.png]

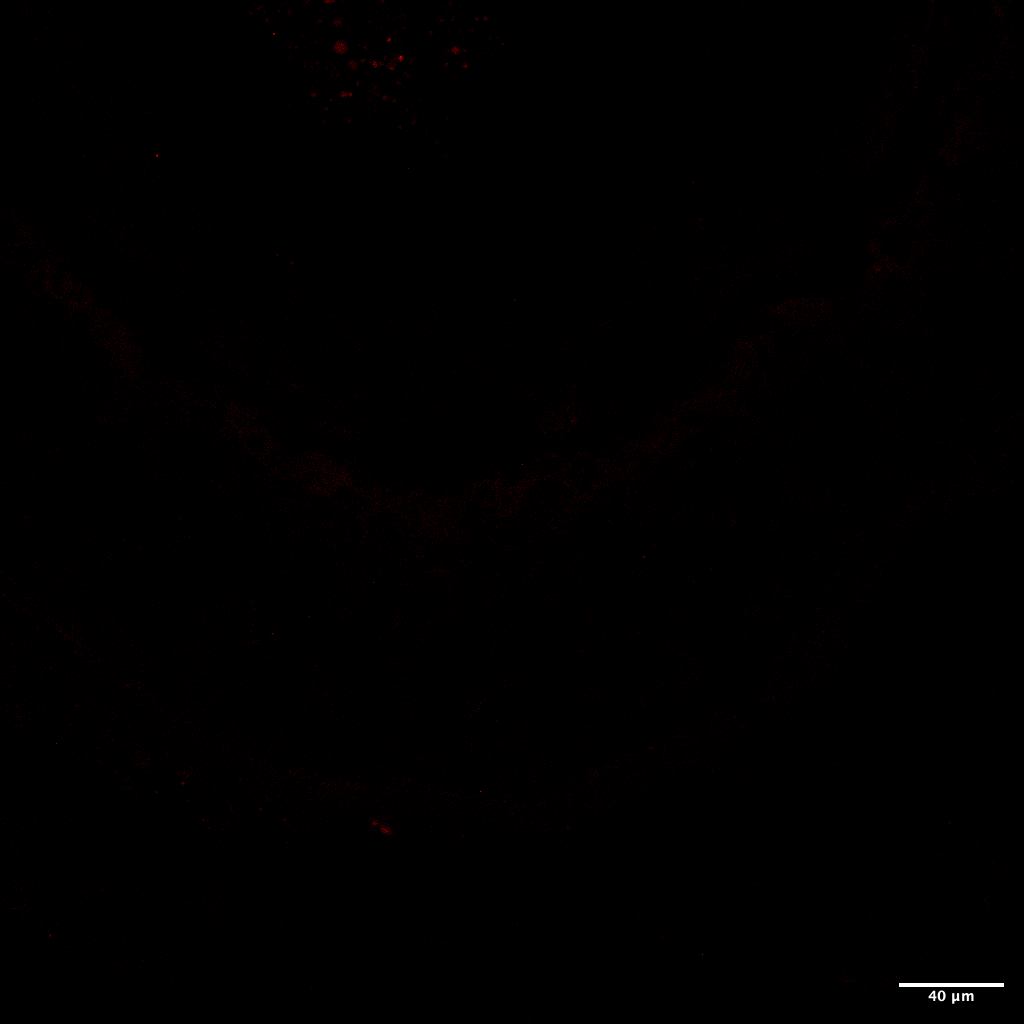

Supplement: Figure 5—source data 3. [file elife-83291-fig5-data3.zip › Figure5_sourcedata_timecourse_F66NR2#1/Day70_OCT4.png]

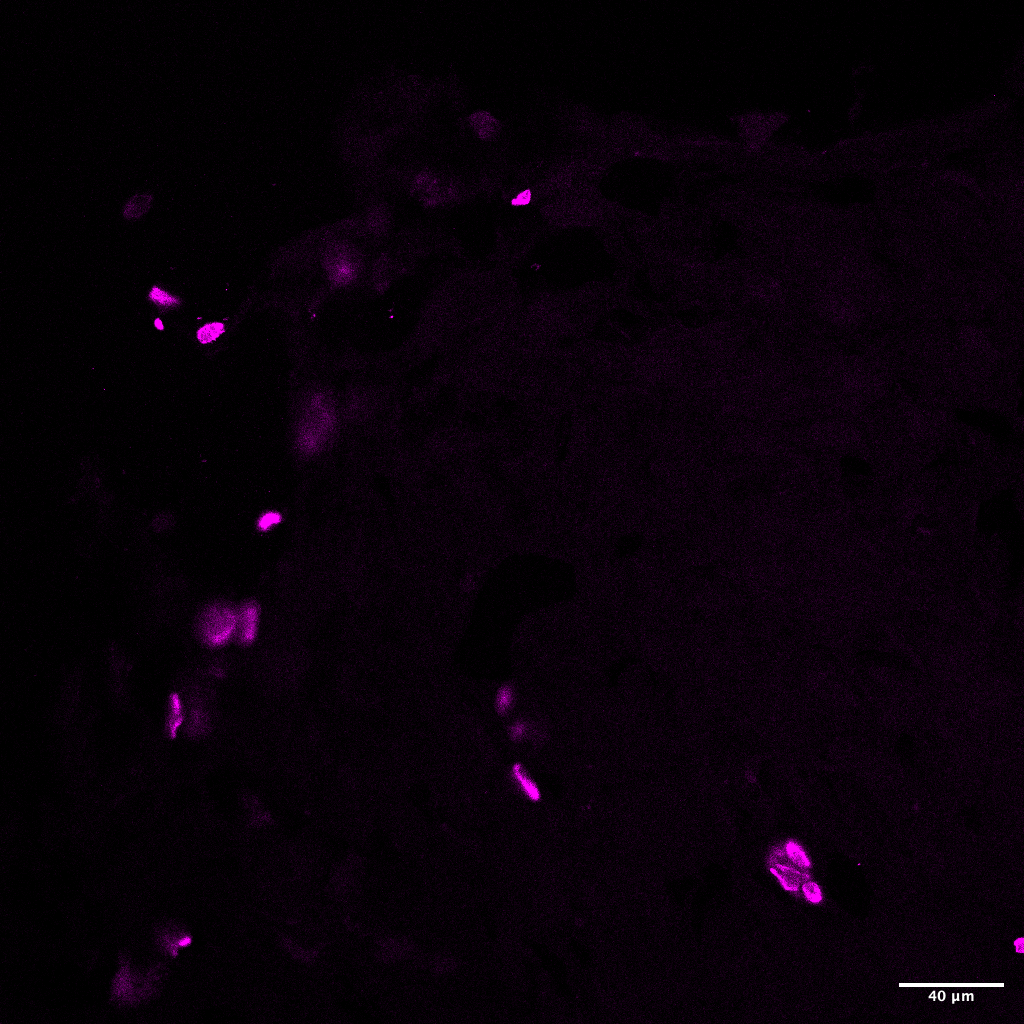

Supplement: Figure 5—source data 3. [file elife-83291-fig5-data3.zip › Figure5_sourcedata_timecourse_F66NR2#1/Day46_FOXL2.png]

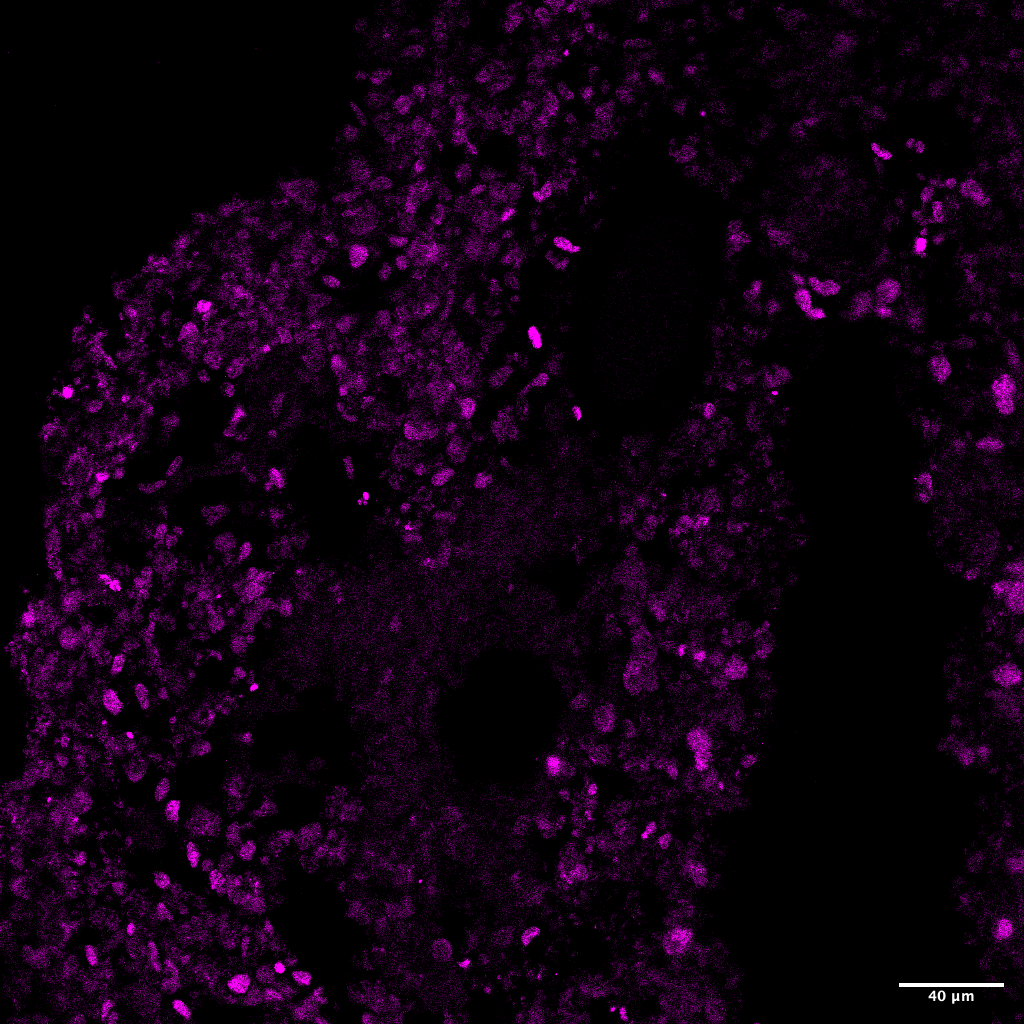

Supplement: Figure 5—source data 3. [file elife-83291-fig5-data3.zip › Figure5_sourcedata_timecourse_F66NR2#1/Day20_3_FOXL2.png]

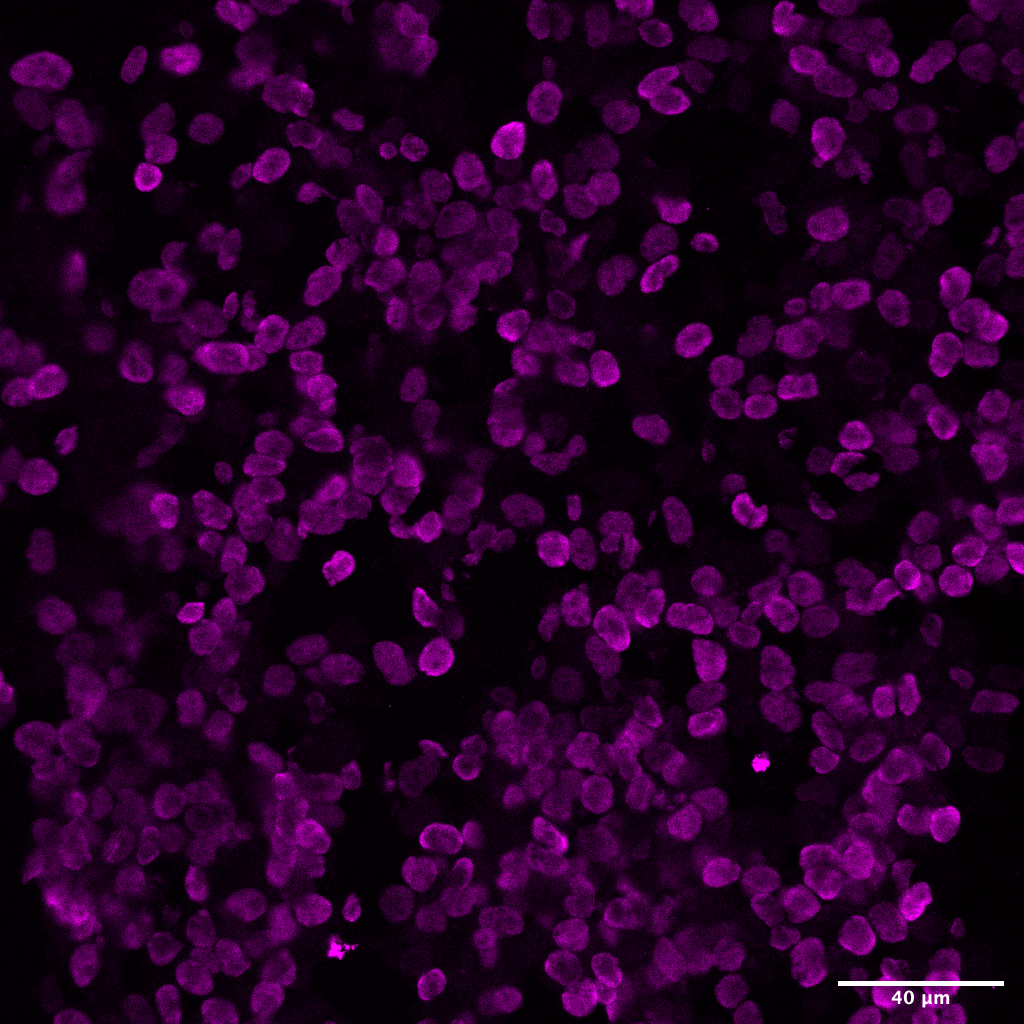

Supplement: Figure 5—source data 3. [file elife-83291-fig5-data3.zip › Figure5_sourcedata_timecourse_F66NR2#1/Day4_FOXL2.png]

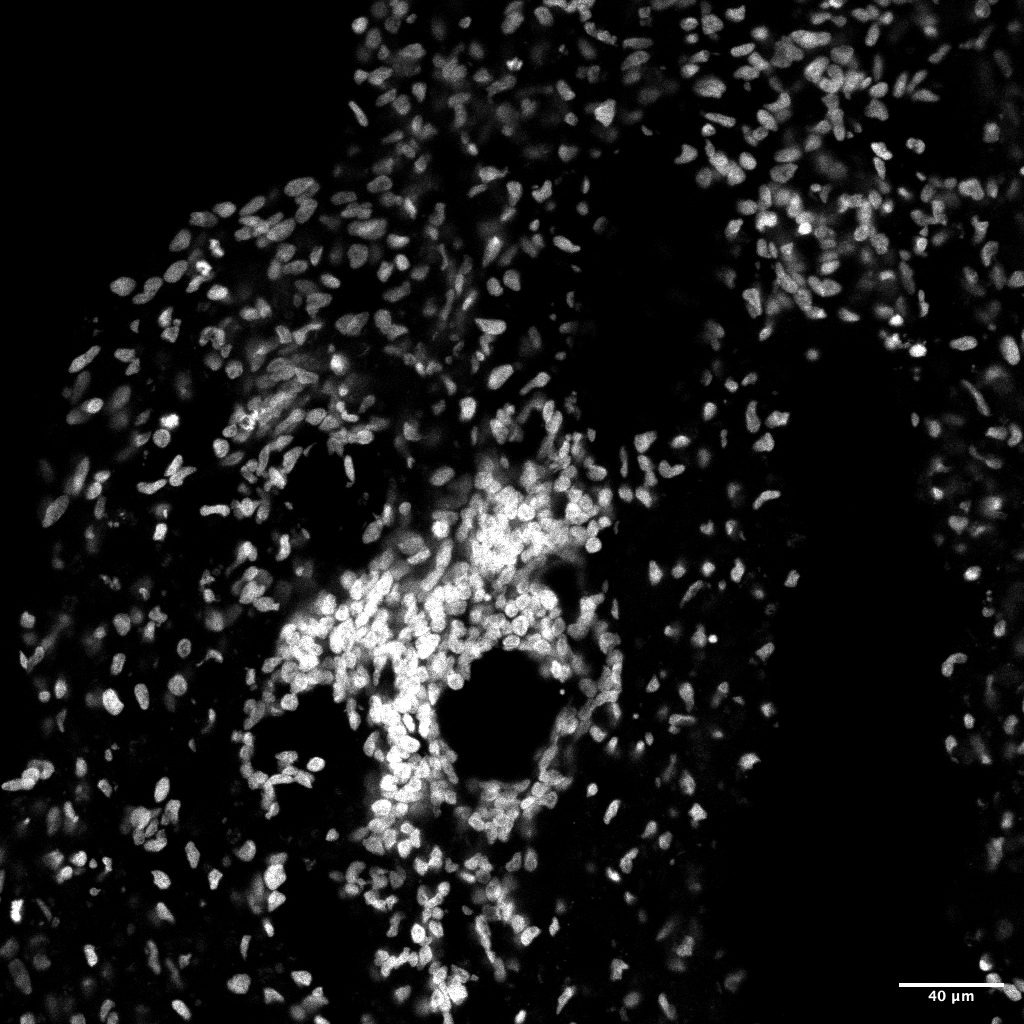

Supplement: Figure 5—source data 3. [file elife-83291-fig5-data3.zip › Figure5_sourcedata_timecourse_F66NR2#1/Day20_3_DAPI.png]

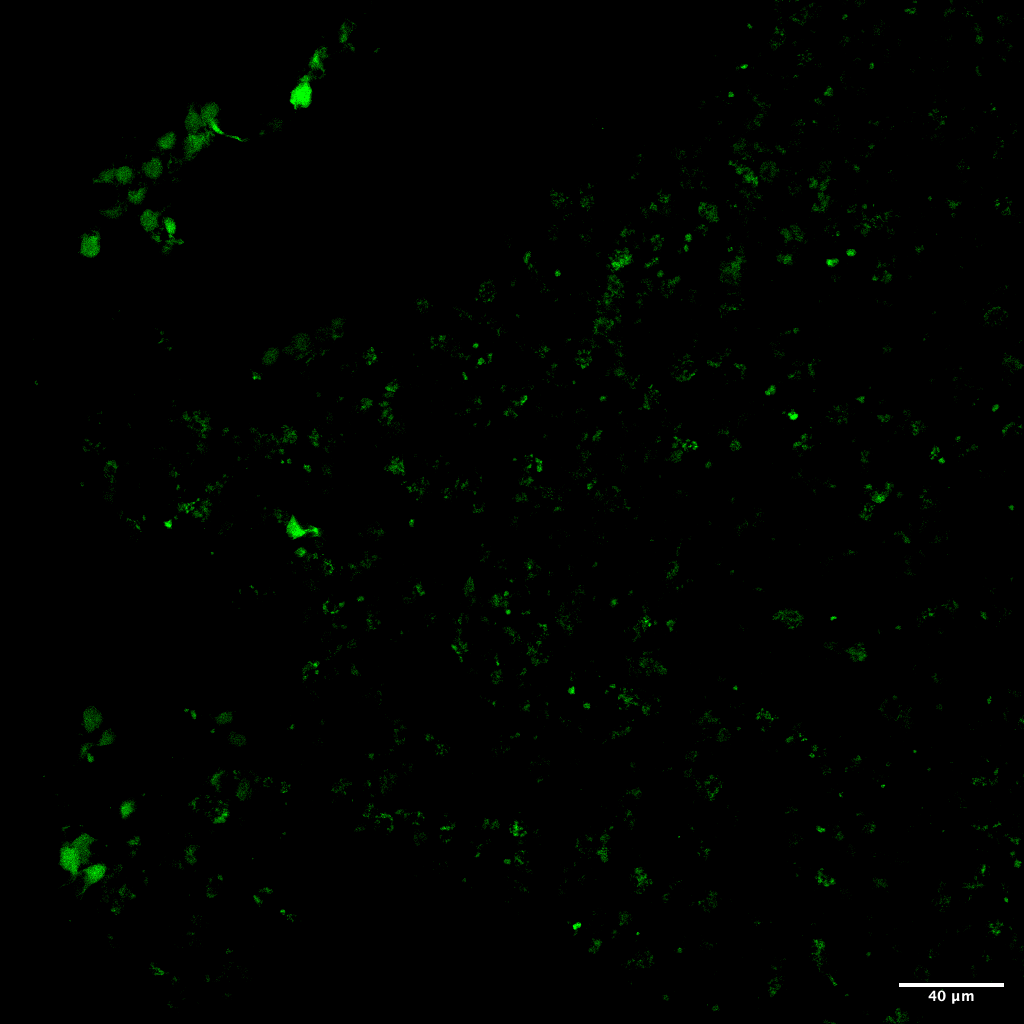

Supplement: Figure 5—source data 3. [file elife-83291-fig5-data3.zip › Figure5_sourcedata_timecourse_F66NR2#1/Day26_DAZL.png]

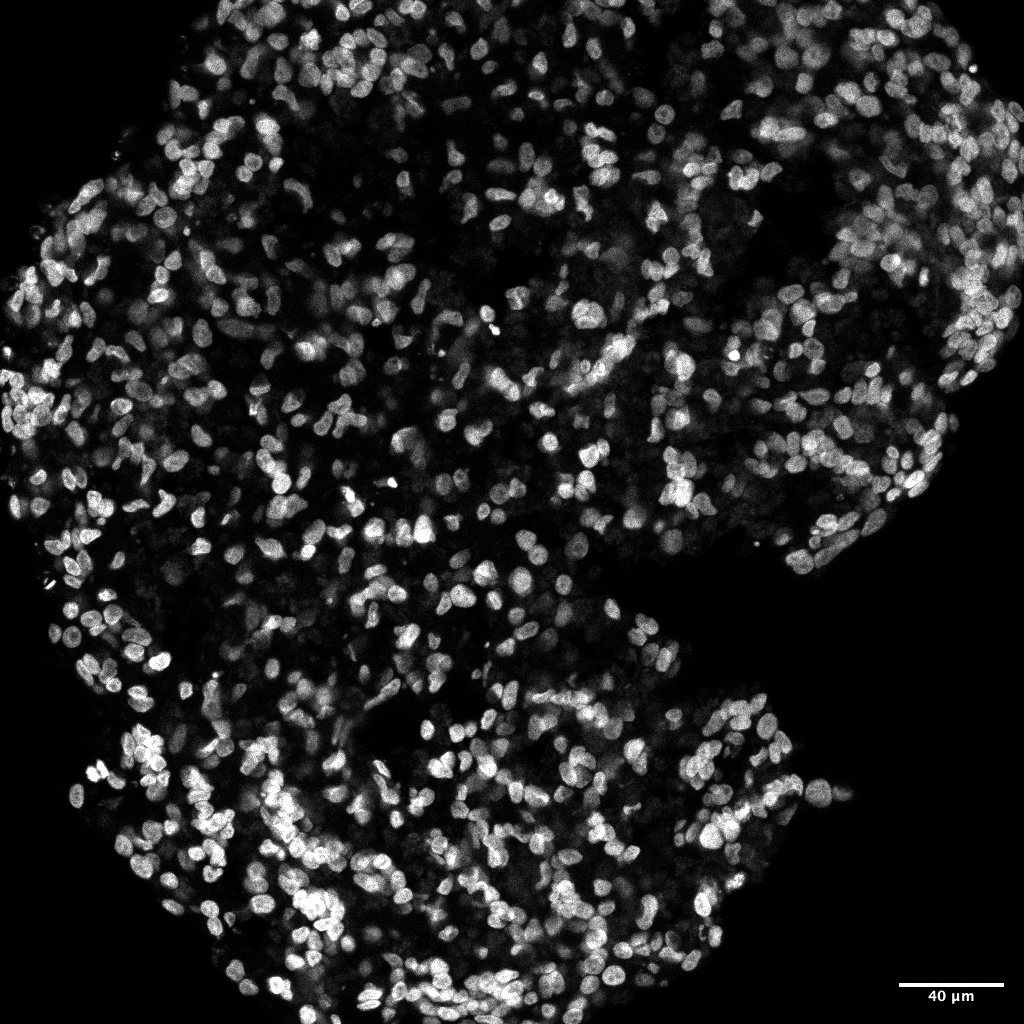

Supplement: Figure 5—source data 3. [file elife-83291-fig5-data3.zip › Figure5_sourcedata_timecourse_F66NR2#1/Day14_DAPI.png]

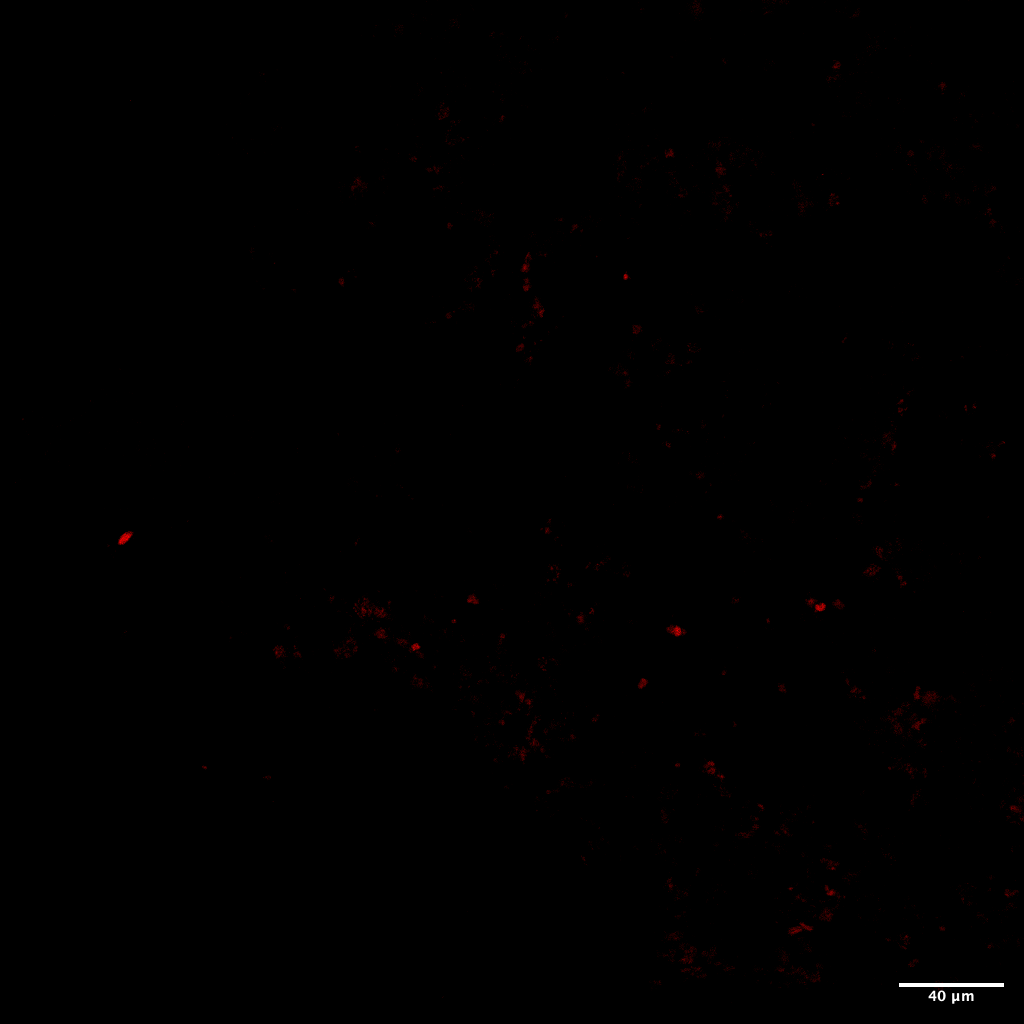

Supplement: Figure 5—source data 3. [file elife-83291-fig5-data3.zip › Figure5_sourcedata_timecourse_F66NR2#1/Day54_OCT4.png]

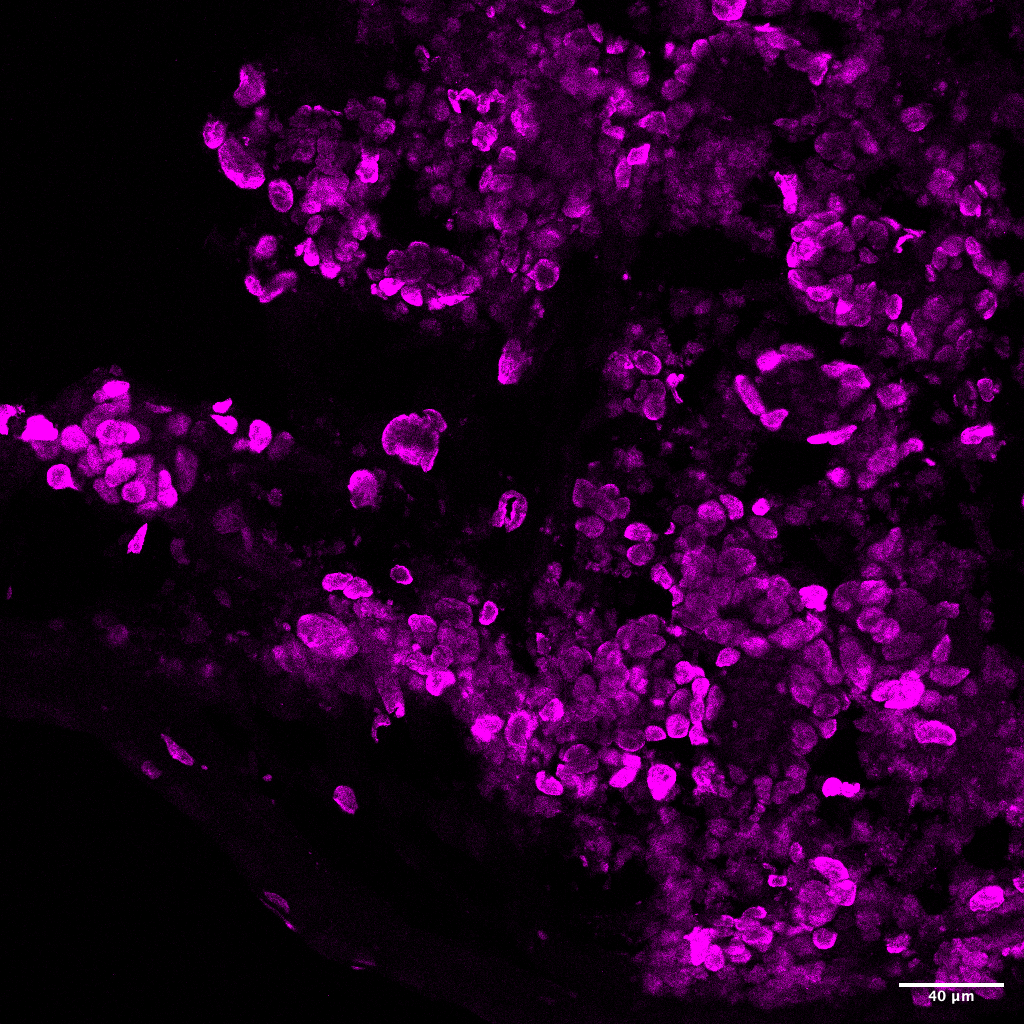

Supplement: Figure 5—source data 3. [file elife-83291-fig5-data3.zip › Figure5_sourcedata_timecourse_F66NR2#1/Day54_FOXL2.png]

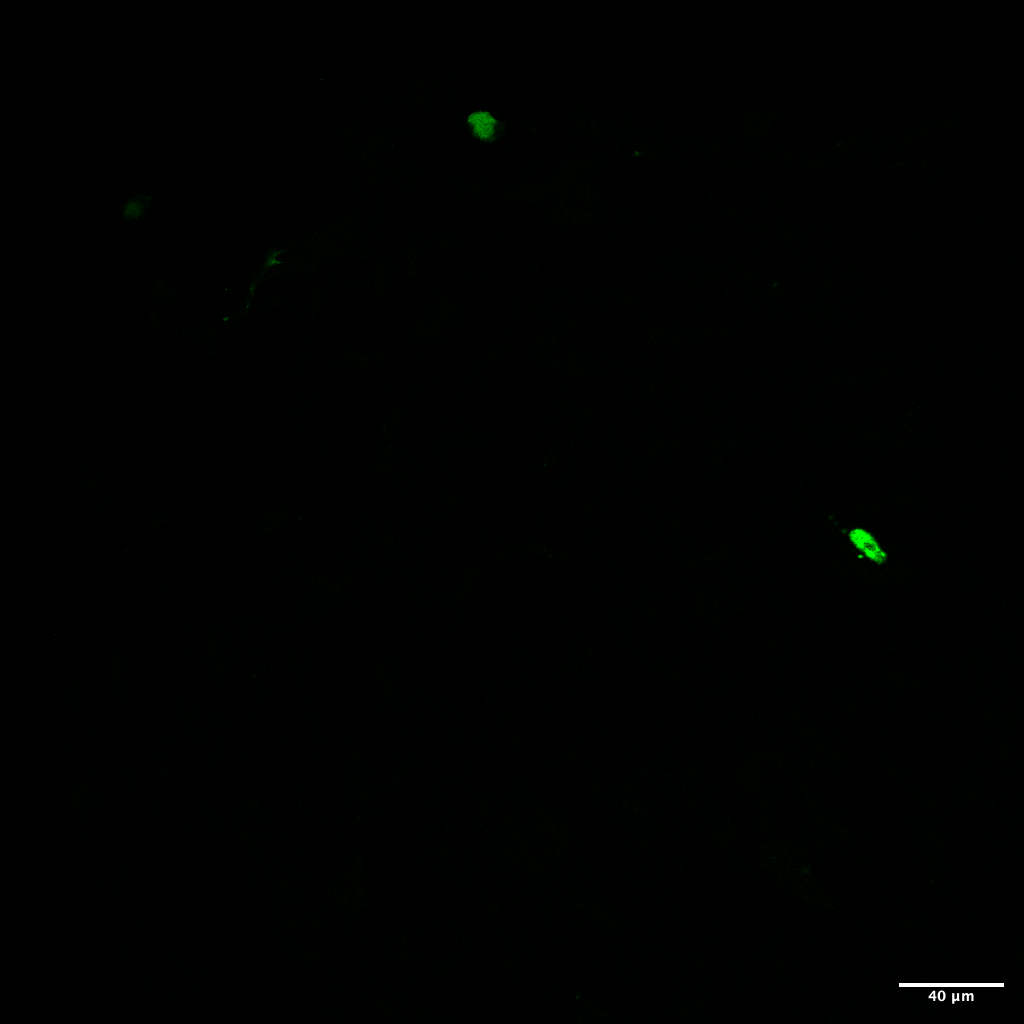

Supplement: Figure 5—source data 3. [file elife-83291-fig5-data3.zip › Figure5_sourcedata_timecourse_F66NR2#1/Day46_DAZL.png]

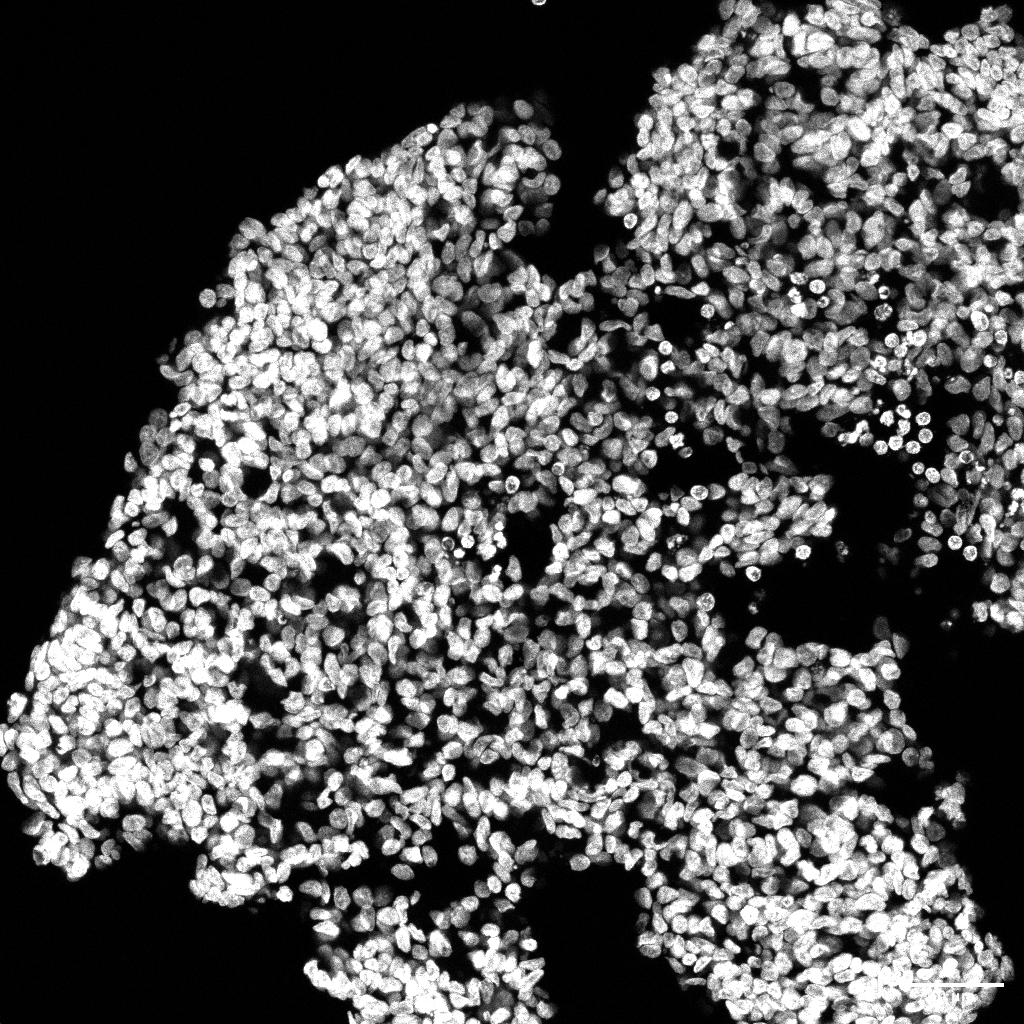

Supplement: Figure 5—source data 3. [file elife-83291-fig5-data3.zip › Figure5_sourcedata_timecourse_F66NR2#1/Day8DAPI.png]

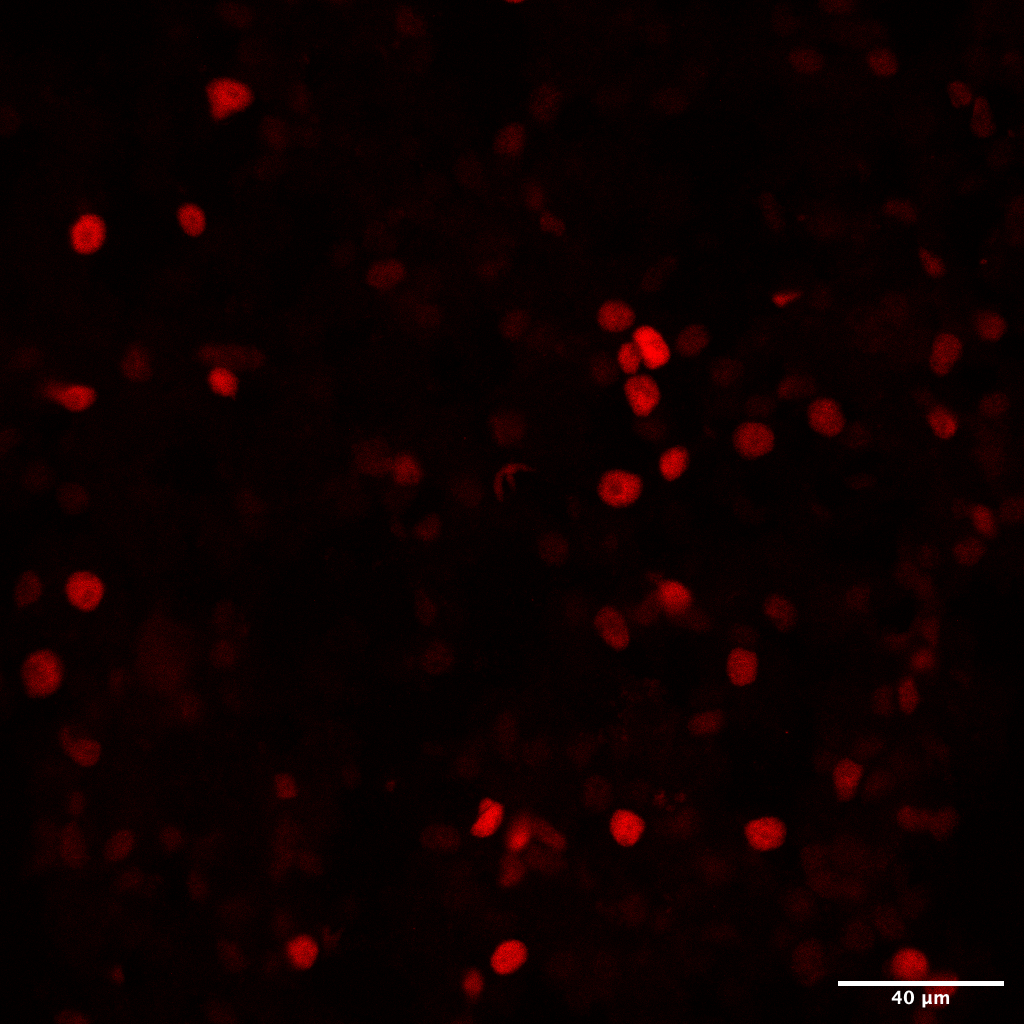

Supplement: Figure 5—source data 3. [file elife-83291-fig5-data3.zip › Figure5_sourcedata_timecourse_F66NR2#1/Day4_OCT4.png]

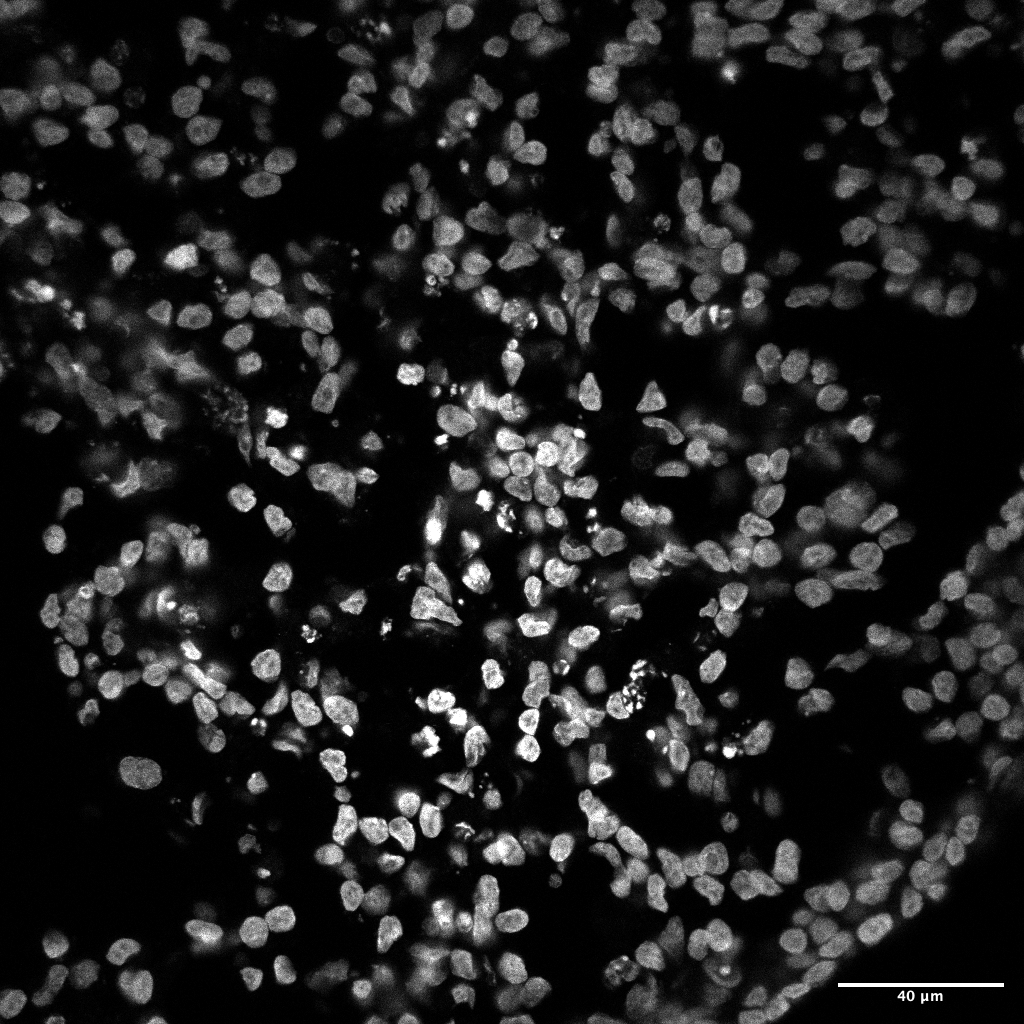

Supplement: Figure 5—source data 3. [file elife-83291-fig5-data3.zip › Figure5_sourcedata_timecourse_F66NR2#1/Day2-_A1_DAPI.png]

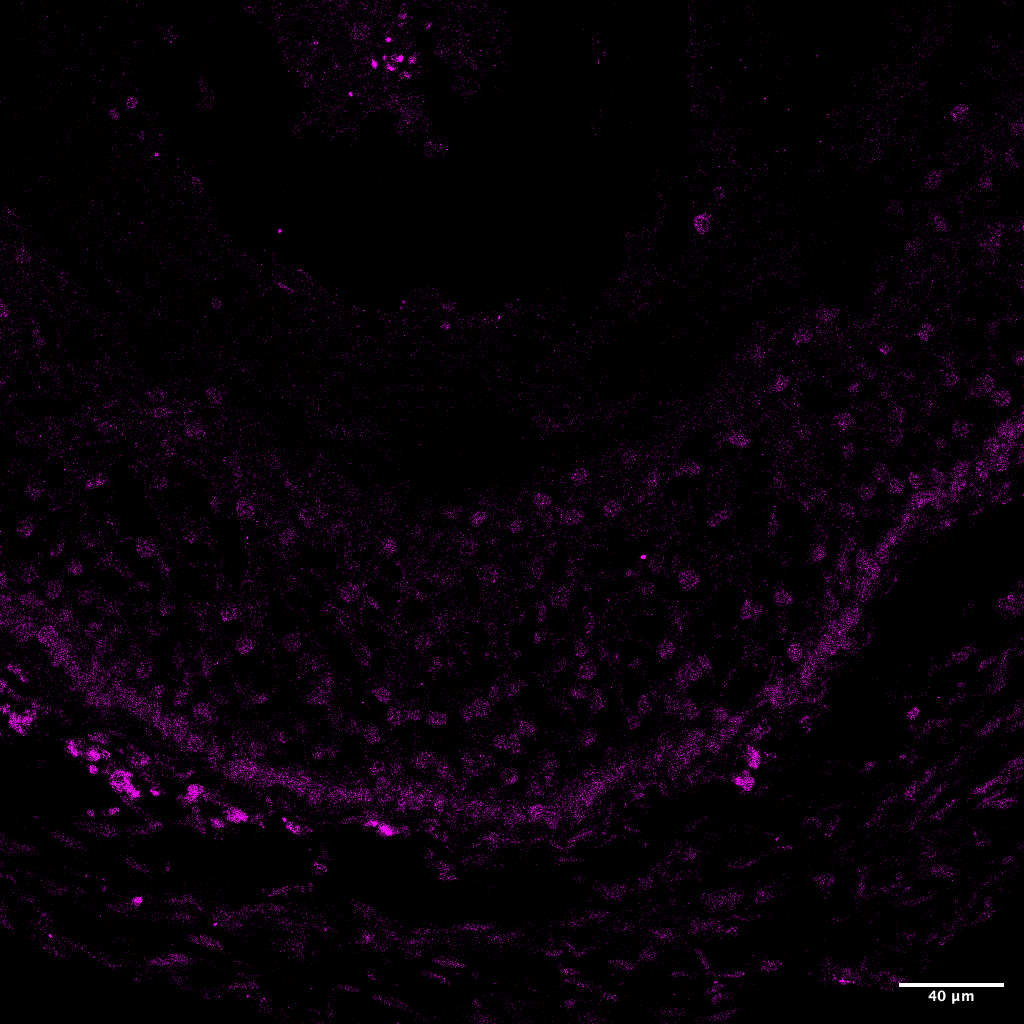

Supplement: Figure 5—source data 3. [file elife-83291-fig5-data3.zip › Figure5_sourcedata_timecourse_F66NR2#1/Day70_FOXL2.png]
