# Supplementary material for: Directed differentiation of human iPSCs to functional ovarian granulosa-like cells via transcription factor overexpression
Source: eLife. 2023 Feb 21;12:e83291. doi: 10.7554/eLife.83291 (PMC9943069; doi:10.7554/eLife.83291)
Supplement: Figure 2—source data 2. [file elife-83291-fig2-data2.docx]

**List of screened TFs**

| **TF** | **Library** | **Reason for inclusion** |
| --- | --- | --- |
| ATF4 | 1 | DEG analysis |
| EMX2 | 1 | DEG analysis |
| FOSB | 1 | DEG analysis |
| HOPX | 1 | DEG analysis |
| HOXC9 | 1 | DEG analysis |
| JUNB | 1 | DEG analysis |
| MAFF | 1 | DEG analysis |
| TSC22D3 | 1 | DEG analysis |
| WT1 | 1 | DEG analysis |
| ZBTB16 | 1 | DEG analysis |
| LHX1 | 1 | Literature |
| LHX9 | 1 | Literature |
| TAF4B | 1 | Literature |
| KLF6 | 1 | Network analysis |
| MYC | 1 | Network analysis |
| NR1H2 | 1 | Network analysis |
| TOX2 | 1 | Network analysis |
| FOS | 1 and 2 | DEG analysis |
| CEBPD | 1 and 2 | DEG analysis |
| ELK1 | 1 and 2 | DEG analysis |
| FOXL2 | 1 and 2 | DEG analysis |
| GATA4 | 1 and 2 | DEG analysis |
| JUN | 1 and 2 | DEG analysis |
| KLF2 | 1 and 2 | DEG analysis |
| NR2F2 | 1 and 2 | DEG analysis |
| NR4A1 | 1 and 2 | DEG analysis |
| NR5A1 | 1 and 2 | DEG analysis |
| TCF21 | 1 and 2 | DEG analysis |
| RUNX1 | 1 and 2 | Literature |
| ZFPM2 | 1 and 2 | Literature |
| EGR1 | 1 and 2 | Network analysis |
| KLF4 | 1 and 2 | Network analysis |
| PPARG | 1 and 2 | Network analysis |
| RUNX2 | 1 and 2 | Network analysis |
| YBX1 | 1 and 2 | Network analysis |
