# Supplementary material for: Directed differentiation of human iPSCs to functional ovarian granulosa-like cells via transcription factor overexpression
Source: eLife. 2023 Feb 21;12:e83291. doi: 10.7554/eLife.83291 (PMC9943069; doi:10.7554/eLife.83291)
Supplement: Figure 3—source data 2. [file elife-83291-fig3-data2.docx]

**List of monoclonal hiPSC lines used for granulosa-like cell production**

| **Line Name** | **Parental Line** | **TFs** |
| --- | --- | --- |
| F3/N.R1 #6 | F3/FOXL2-T2A-tdTomato | NR5A1, RUNX1 |
| F3/N.R2 #4 | F3/FOXL2-T2A-tdTomato | NR5A1, RUNX2 |
| F3/N.T #5 | F3/FOXL2-T2A-tdTomato | NR5A1, TCF21 |
| F66/N.R1 #7 | F66 | NR5A1, RUNX1 |
| F66/N.R1.G.F #4 | F66 | NR5A1, RUNX1, GATA4, FOXL2 |
| F66/N.R1.G #1 | F66 | NR5A1, RUNX1, GATA4 |
| F66/N.R2 #1 | F66 | NR5A1, RUNX2 |
| F66/N.R2 #5 | F66 | NR5A1, RUNX2 |
| F66/N.R2.G #3 | F66 | NR5A1, RUNX2, GATA4 |

Presence of TF integration was confirmed by PCR, see Figure 3-Figure Supplement 1.
