# Supplementary figures and images for: Directed differentiation of human iPSCs to functional ovarian granulosa-like cells via transcription factor overexpression (part 2 of 3)
Source: eLife. 2023 Feb 21;12:e83291. doi: 10.7554/eLife.83291 (PMC9943069; doi:10.7554/eLife.83291)

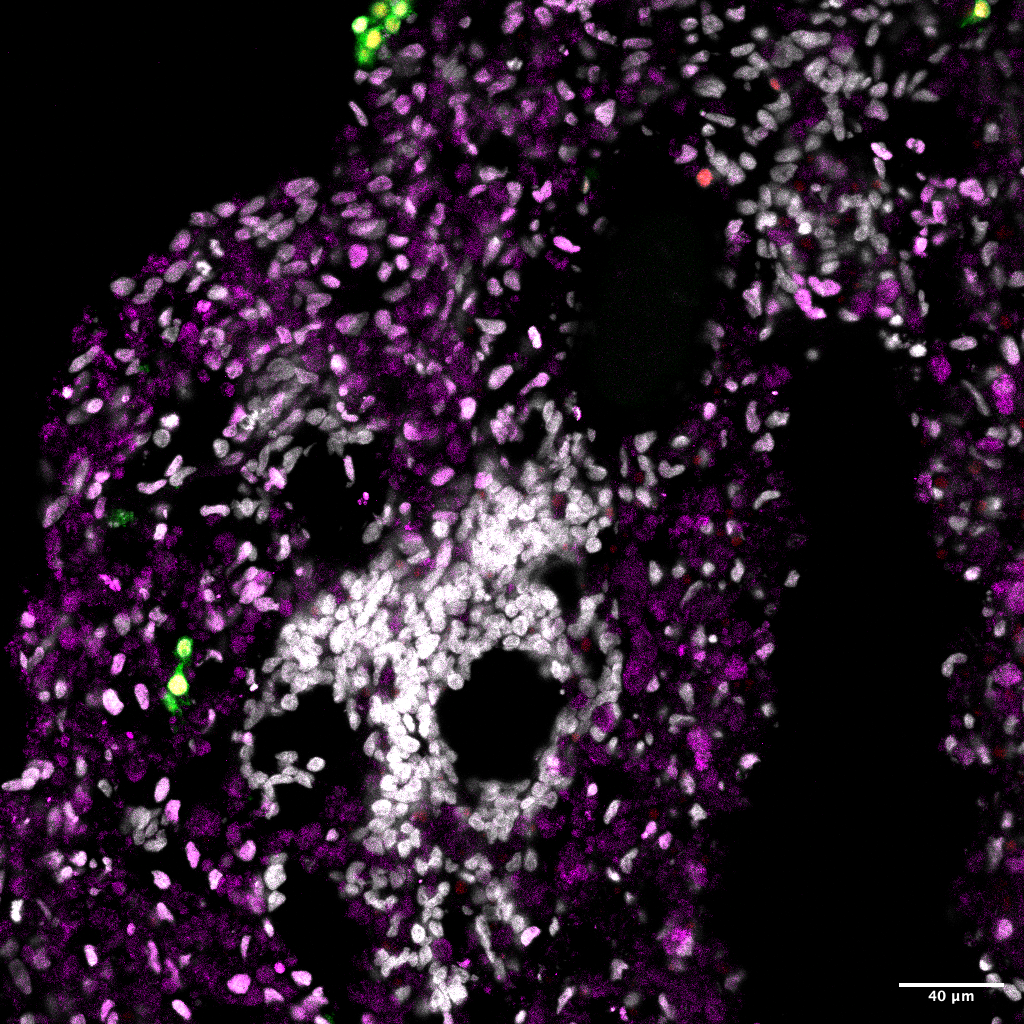

Supplement: Figure 5—source data 3. [file elife-83291-fig5-data3.zip › Figure5_sourcedata_timecourse_F66NR2#1/Day20_3_overlay.png]

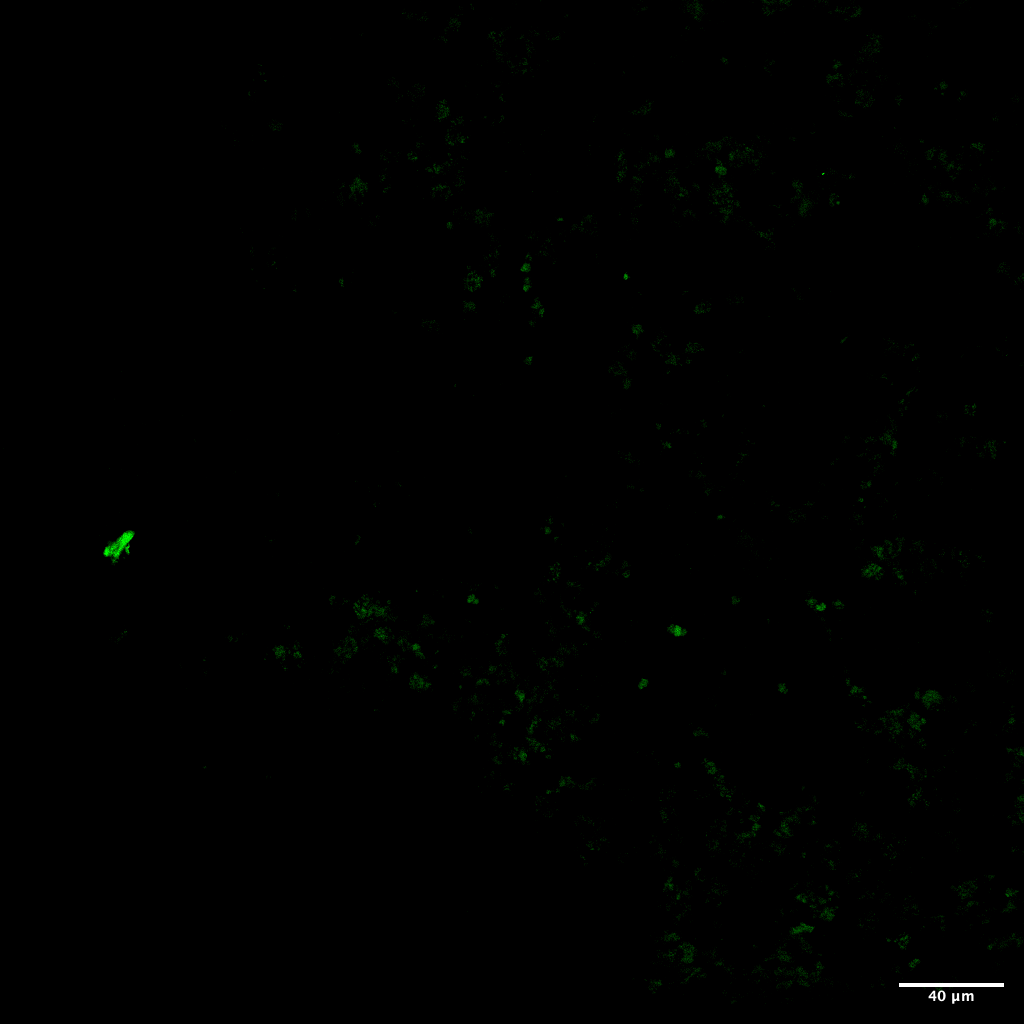

Supplement: Figure 5—source data 3. [file elife-83291-fig5-data3.zip › Figure5_sourcedata_timecourse_F66NR2#1/Day54_DAZL.png]

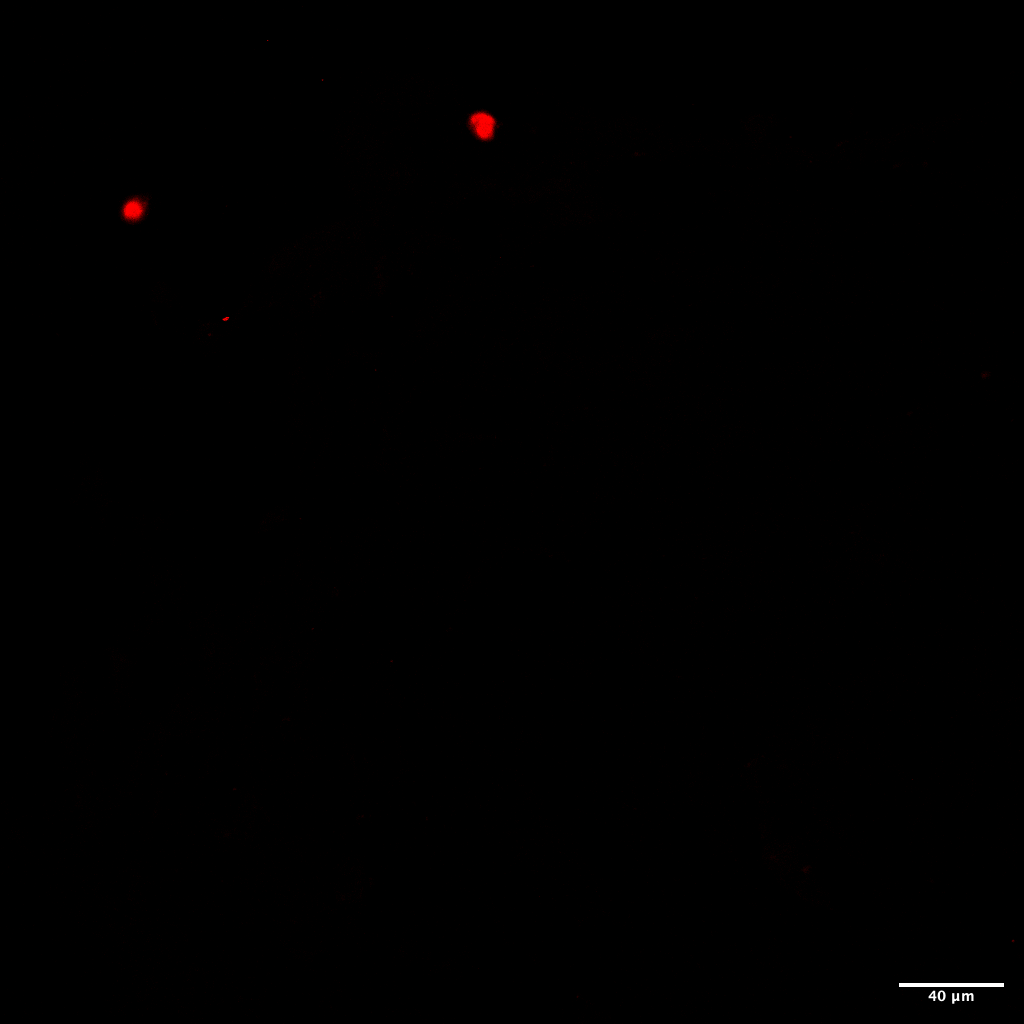

Supplement: Figure 5—source data 3. [file elife-83291-fig5-data3.zip › Figure5_sourcedata_timecourse_F66NR2#1/Day46_OCT4.png]

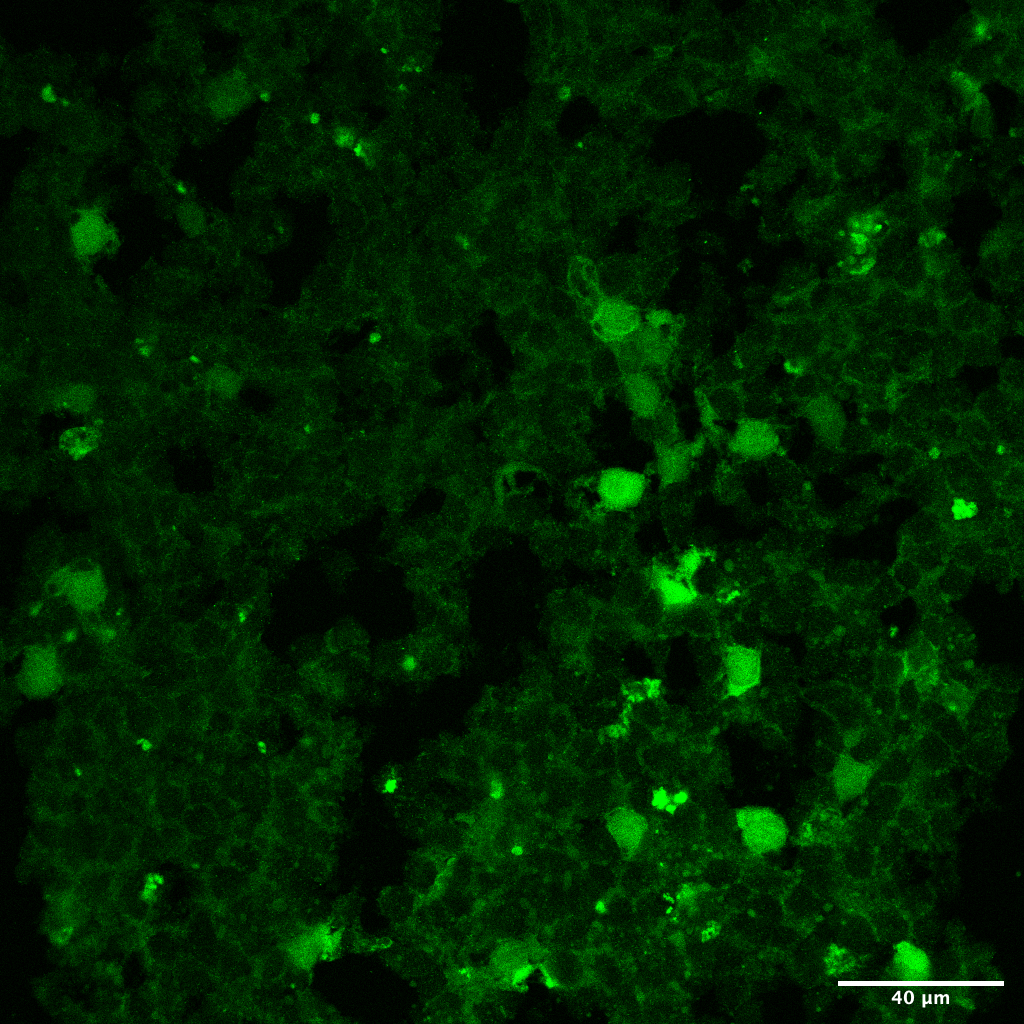

Supplement: Figure 5—source data 3. [file elife-83291-fig5-data3.zip › Figure5_sourcedata_timecourse_F66NR2#1/Day4_DAZL.png]

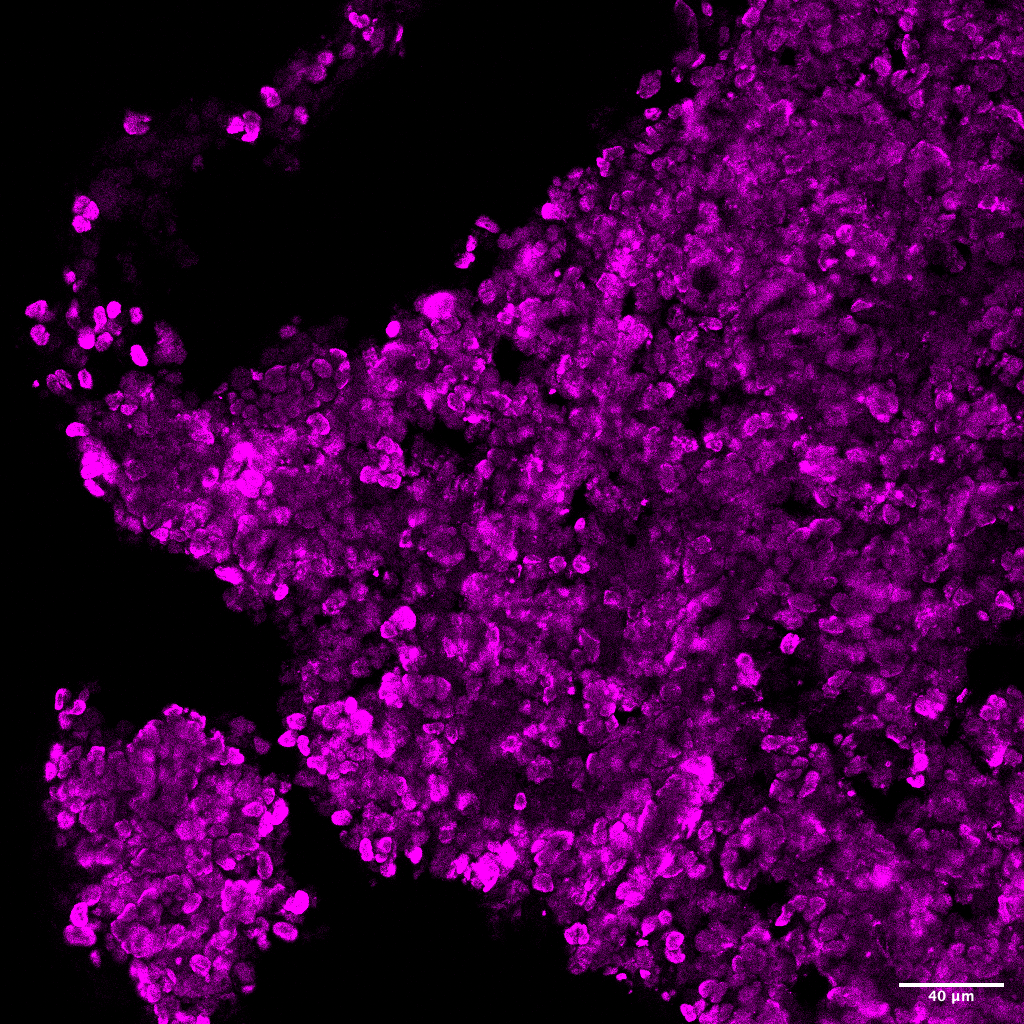

Supplement: Figure 5—source data 3. [file elife-83291-fig5-data3.zip › Figure5_sourcedata_timecourse_F66NR2#1/Day26_FOXL2.png]

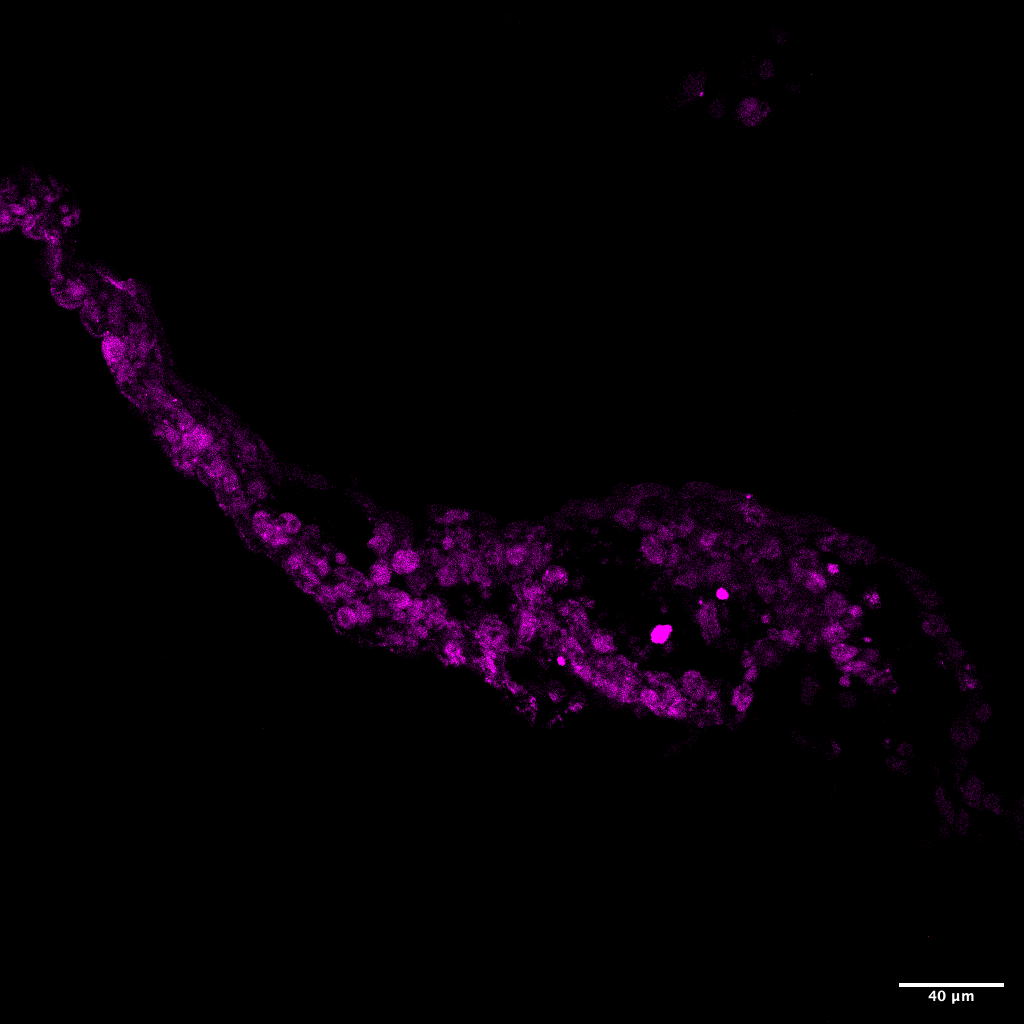

Supplement: Figure 5—source data 3. [file elife-83291-fig5-data3.zip › Figure5_sourcedata_timecourse_F66NR2#1/Day32_FOXL2.png]

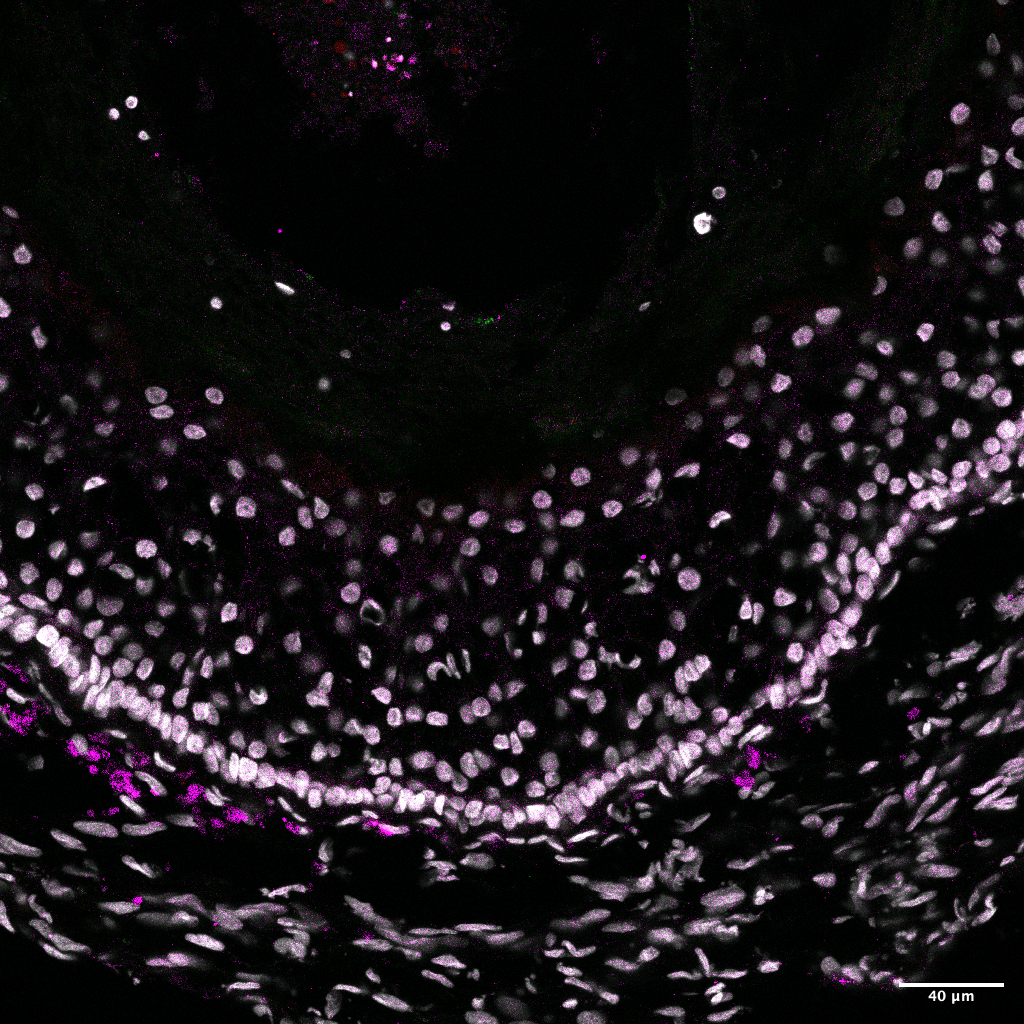

Supplement: Figure 5—source data 3. [file elife-83291-fig5-data3.zip › Figure5_sourcedata_timecourse_F66NR2#1/Day70_overlay.png]

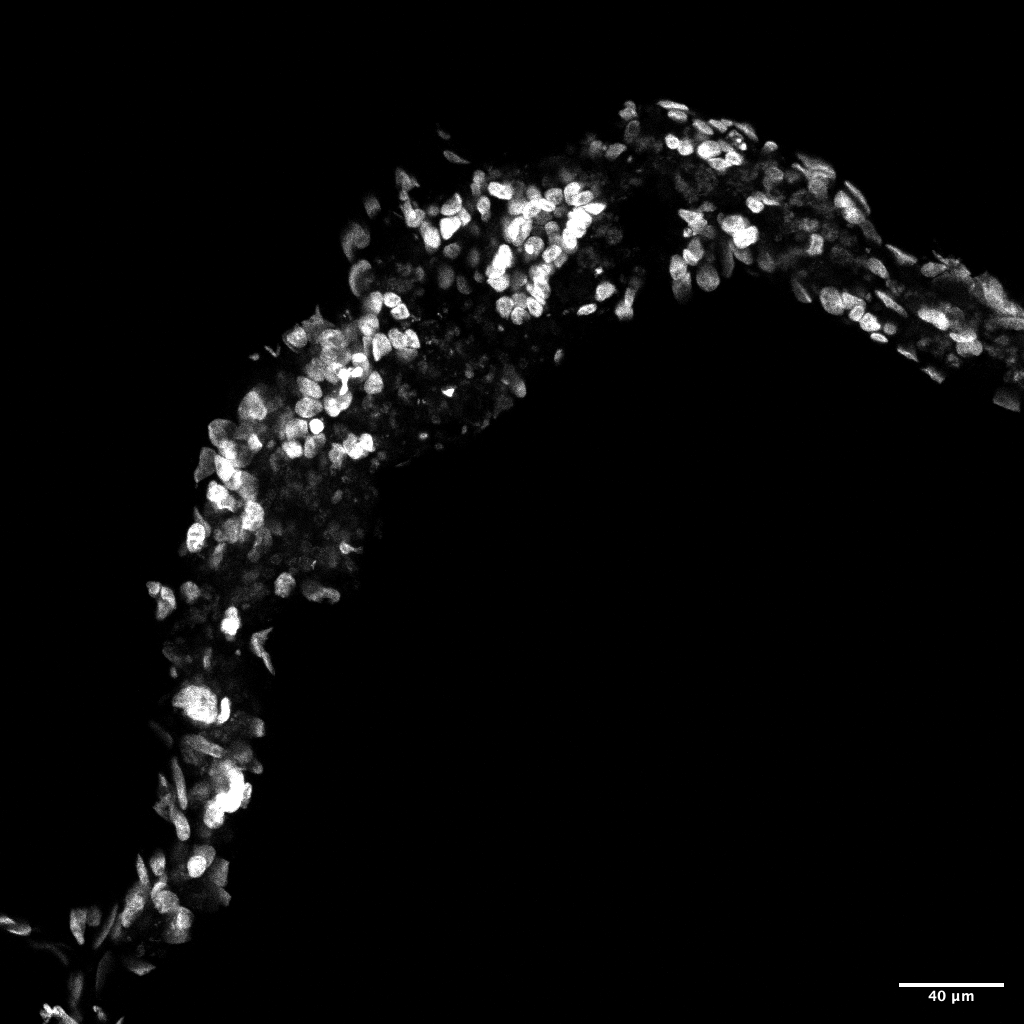

Supplement: Figure 5—source data 3. [file elife-83291-fig5-data3.zip › Figure5_sourcedata_timecourse_F66NR2#1/Day38_DAPI.png]

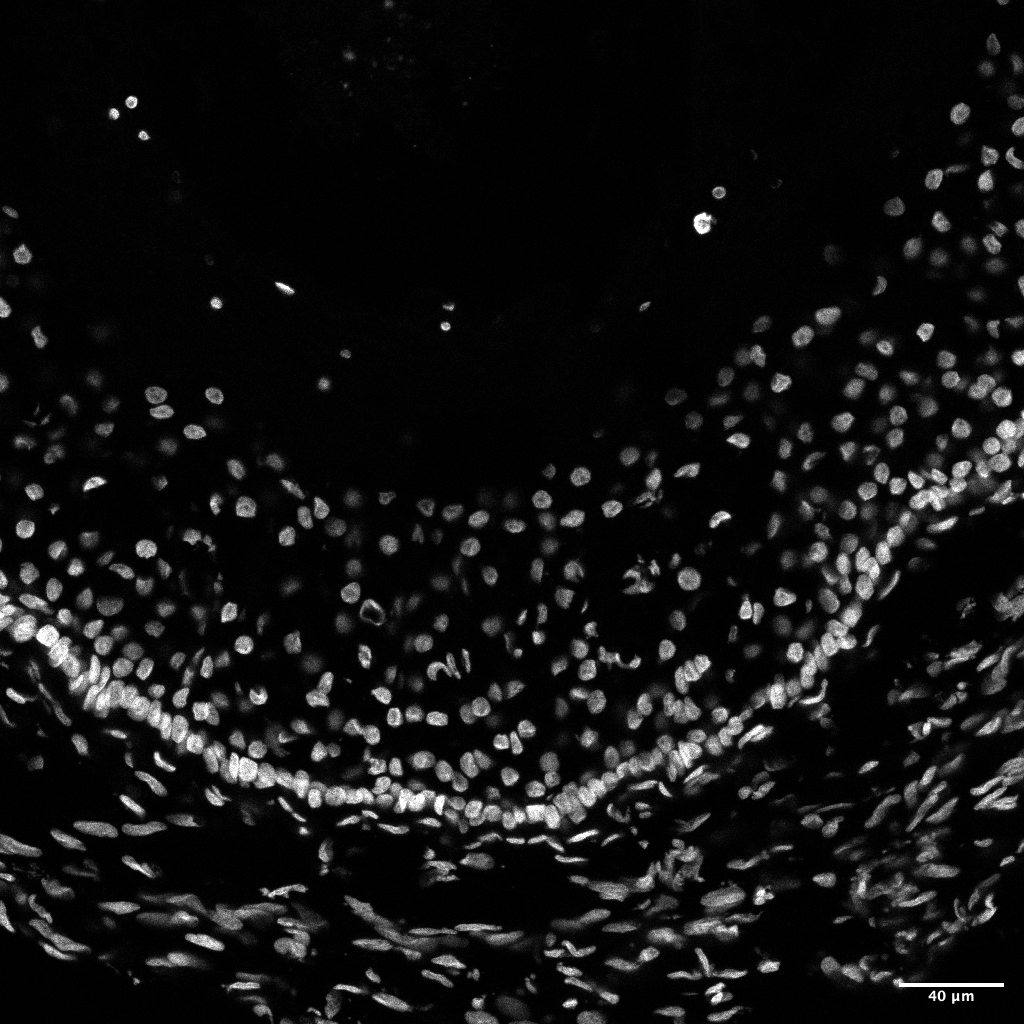

Supplement: Figure 5—source data 3. [file elife-83291-fig5-data3.zip › Figure5_sourcedata_timecourse_F66NR2#1/Day70_DAPI.png]

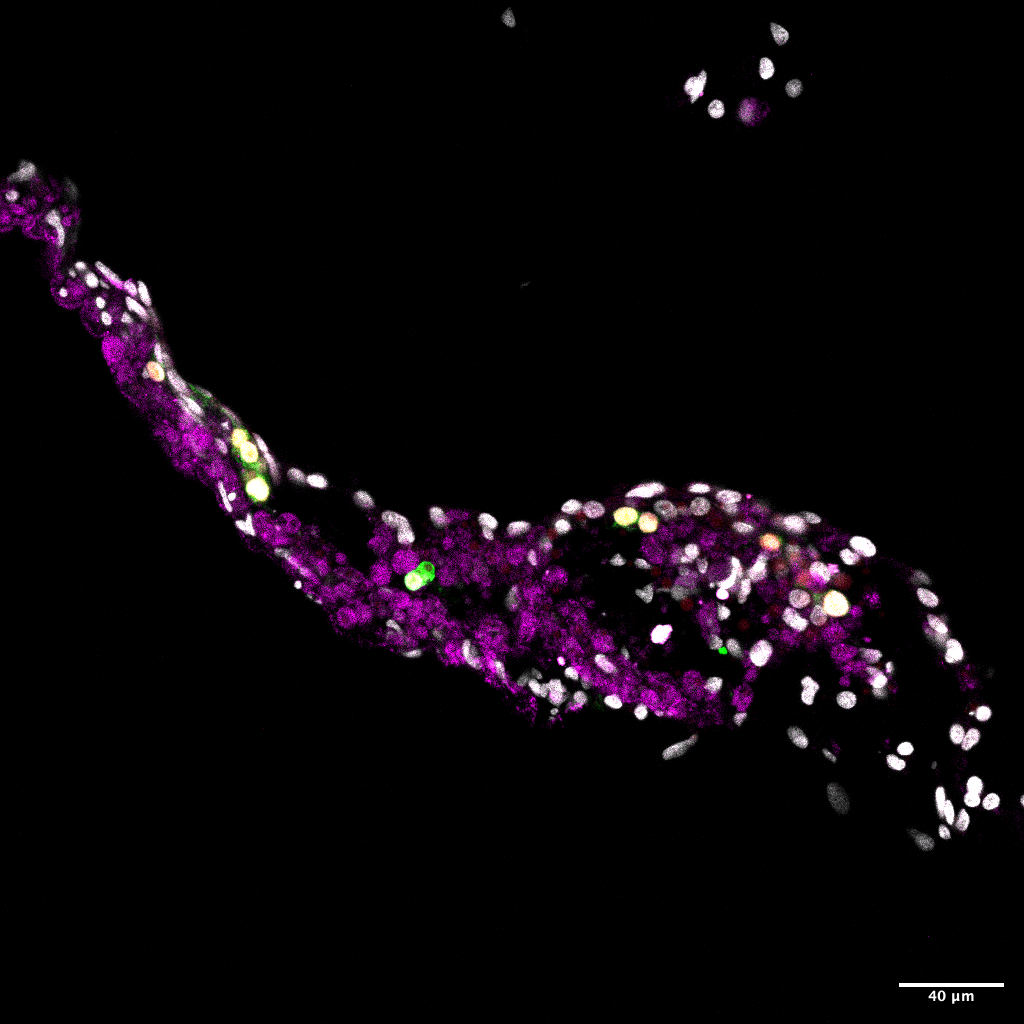

Supplement: Figure 5—source data 3. [file elife-83291-fig5-data3.zip › Figure5_sourcedata_timecourse_F66NR2#1/Day32_overlay.png]

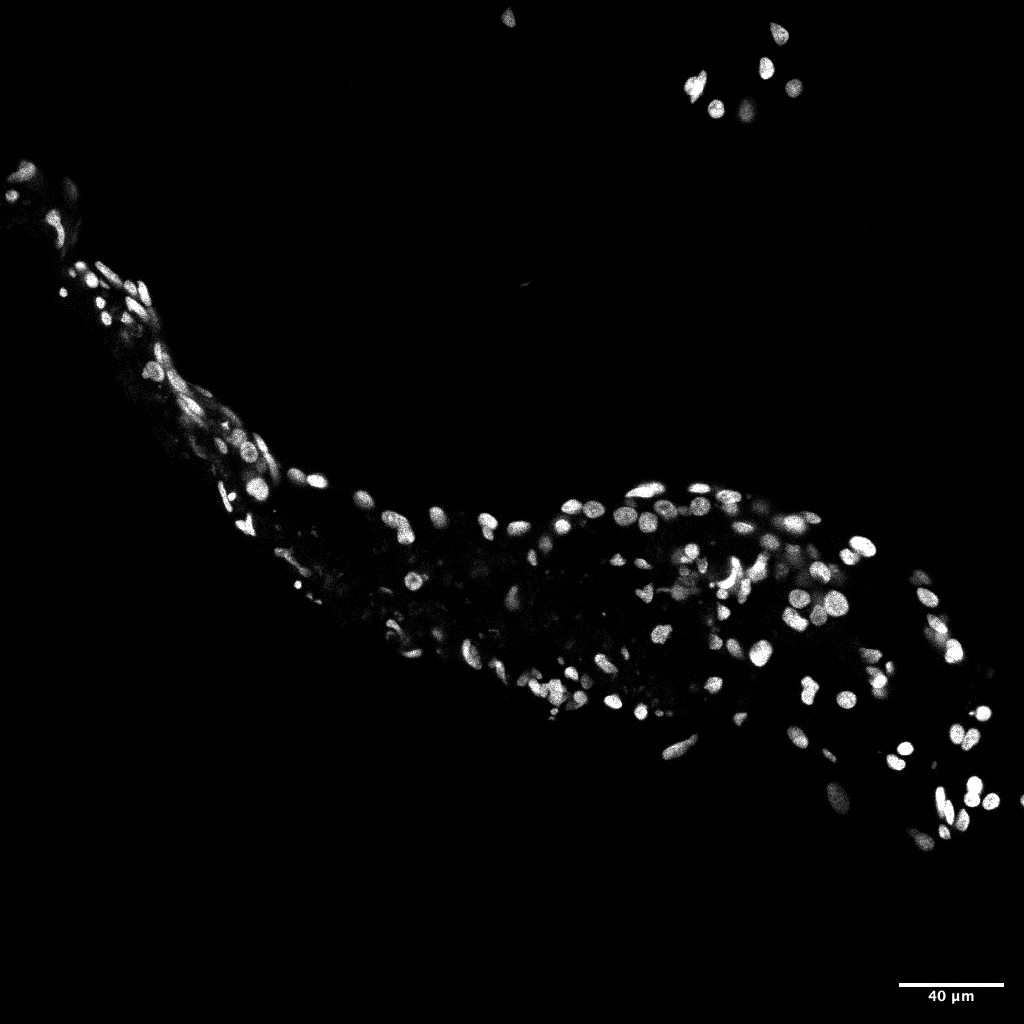

Supplement: Figure 5—source data 3. [file elife-83291-fig5-data3.zip › Figure5_sourcedata_timecourse_F66NR2#1/Day32_DAPI.png]

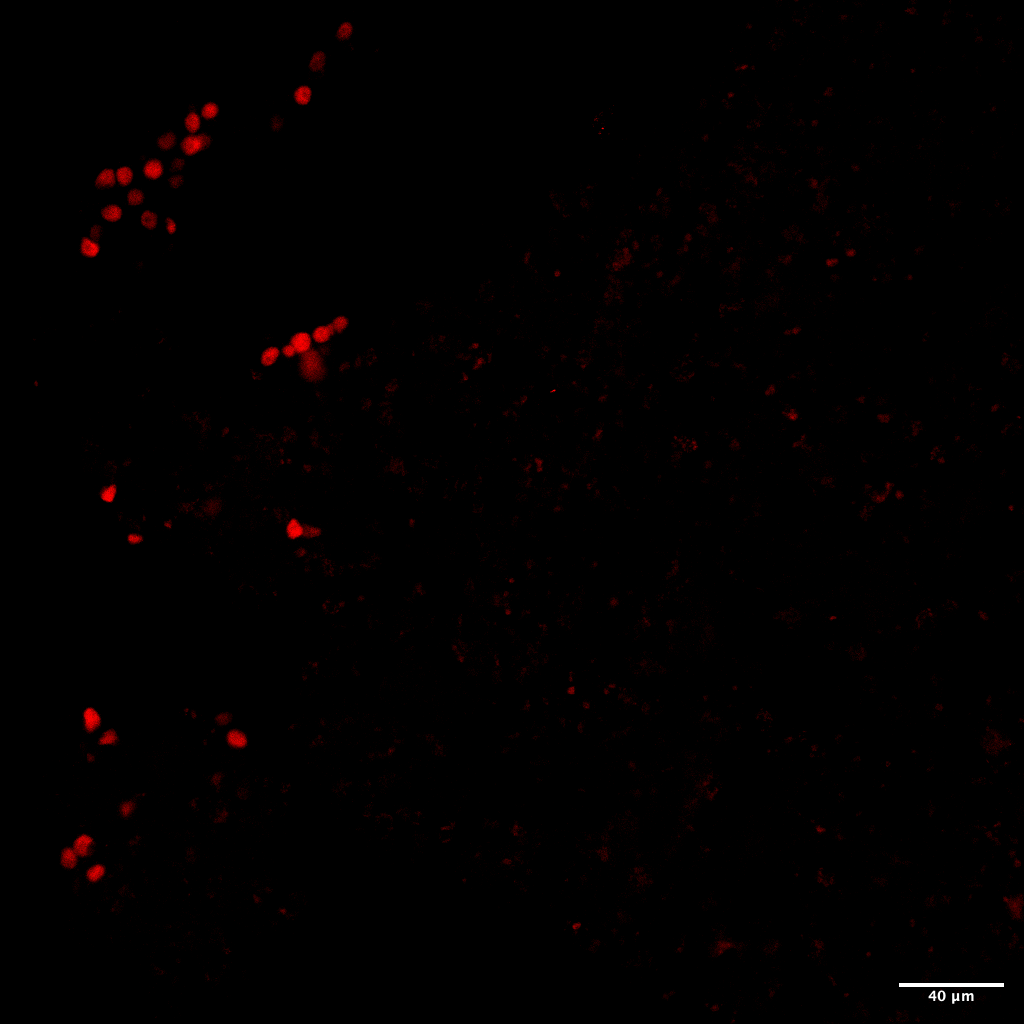

Supplement: Figure 5—source data 3. [file elife-83291-fig5-data3.zip › Figure5_sourcedata_timecourse_F66NR2#1/Day26_OCT4.png]

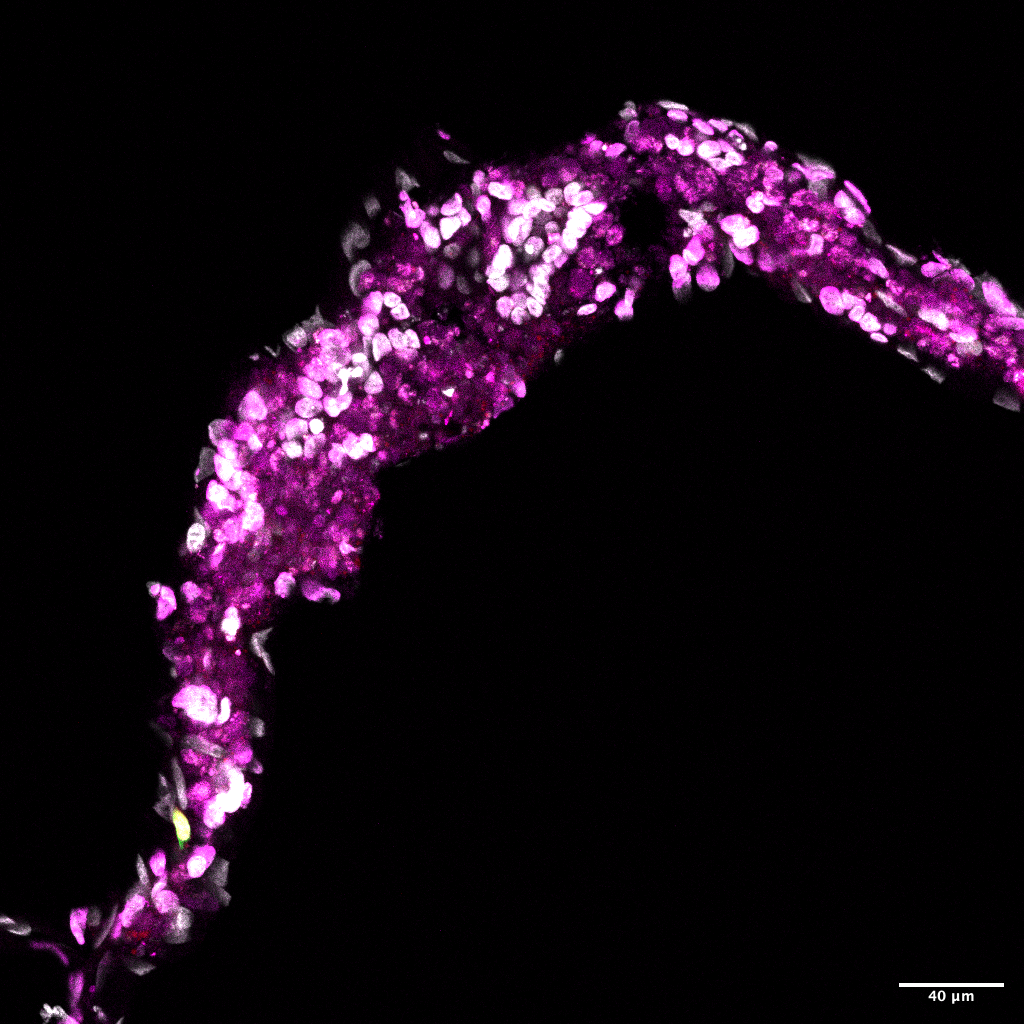

Supplement: Figure 5—source data 3. [file elife-83291-fig5-data3.zip › Figure5_sourcedata_timecourse_F66NR2#1/Day38_overlay.png]

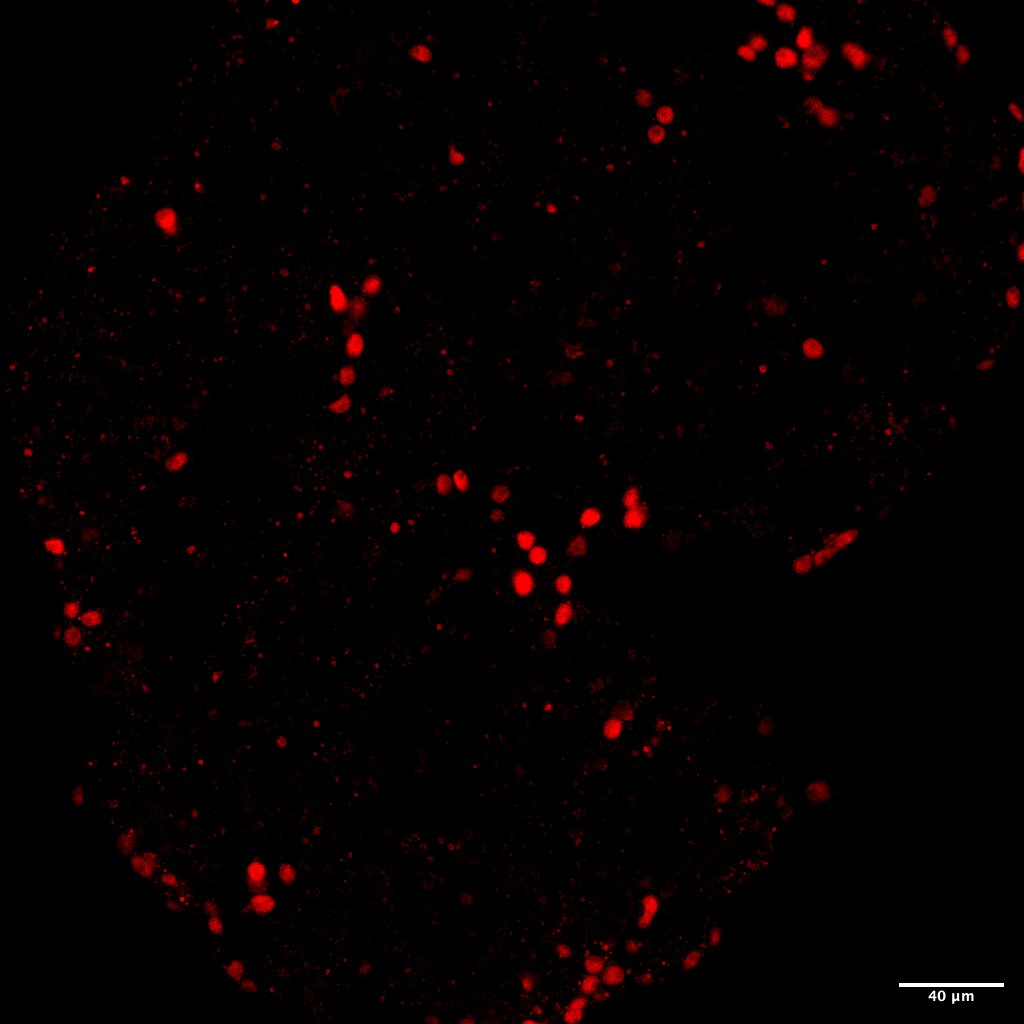

Supplement: Figure 5—source data 3. [file elife-83291-fig5-data3.zip › Figure5_sourcedata_timecourse_F66NR2#1/Day14_OCT4.png]

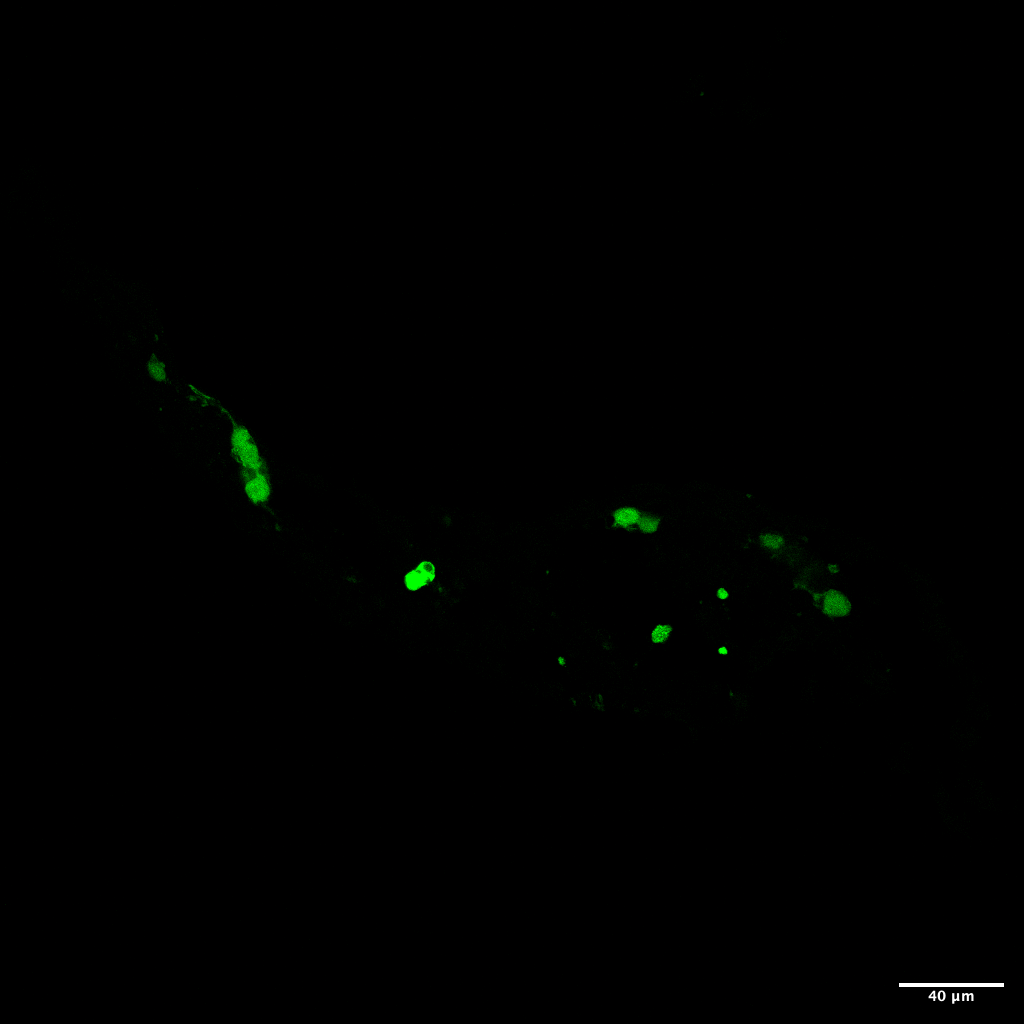

Supplement: Figure 5—source data 3. [file elife-83291-fig5-data3.zip › Figure5_sourcedata_timecourse_F66NR2#1/Day32_DAZL.png]

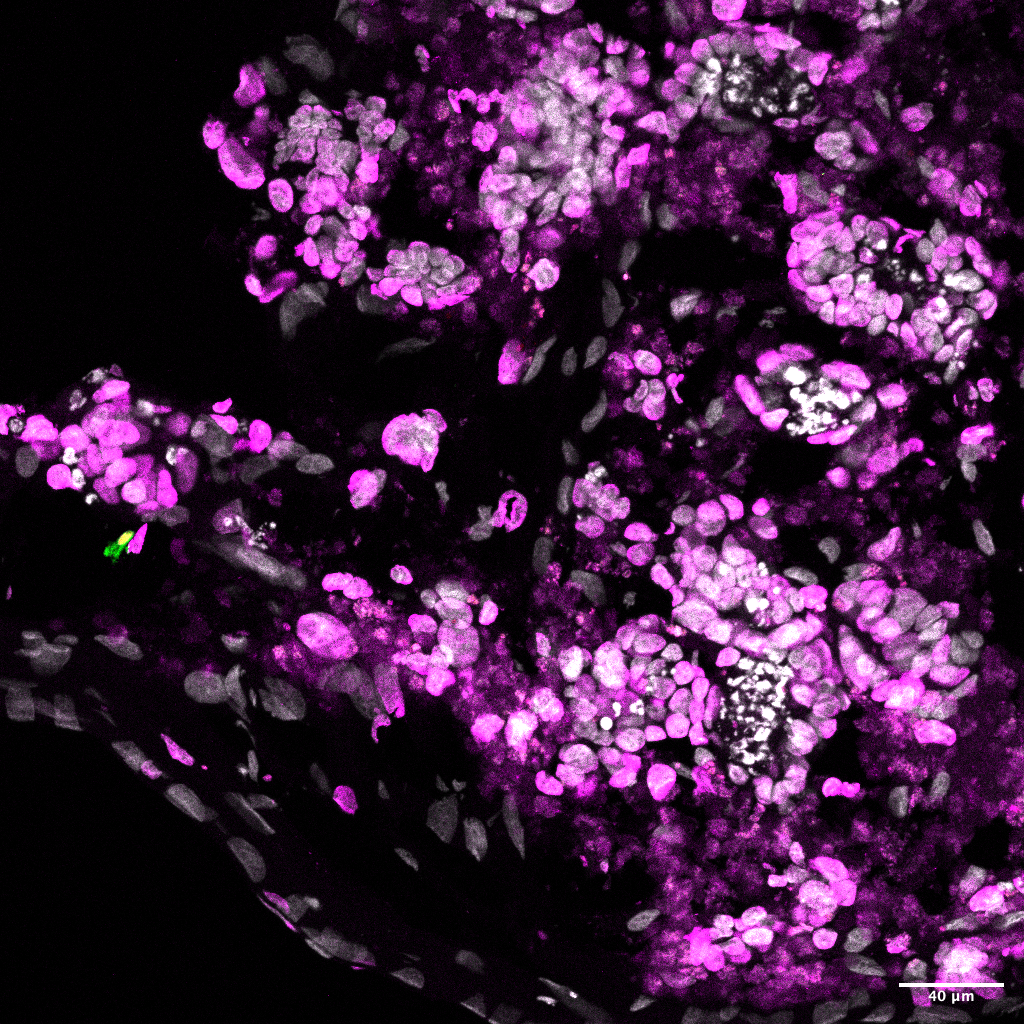

Supplement: Figure 5—source data 3. [file elife-83291-fig5-data3.zip › Figure5_sourcedata_timecourse_F66NR2#1/Day54_overlay.png]

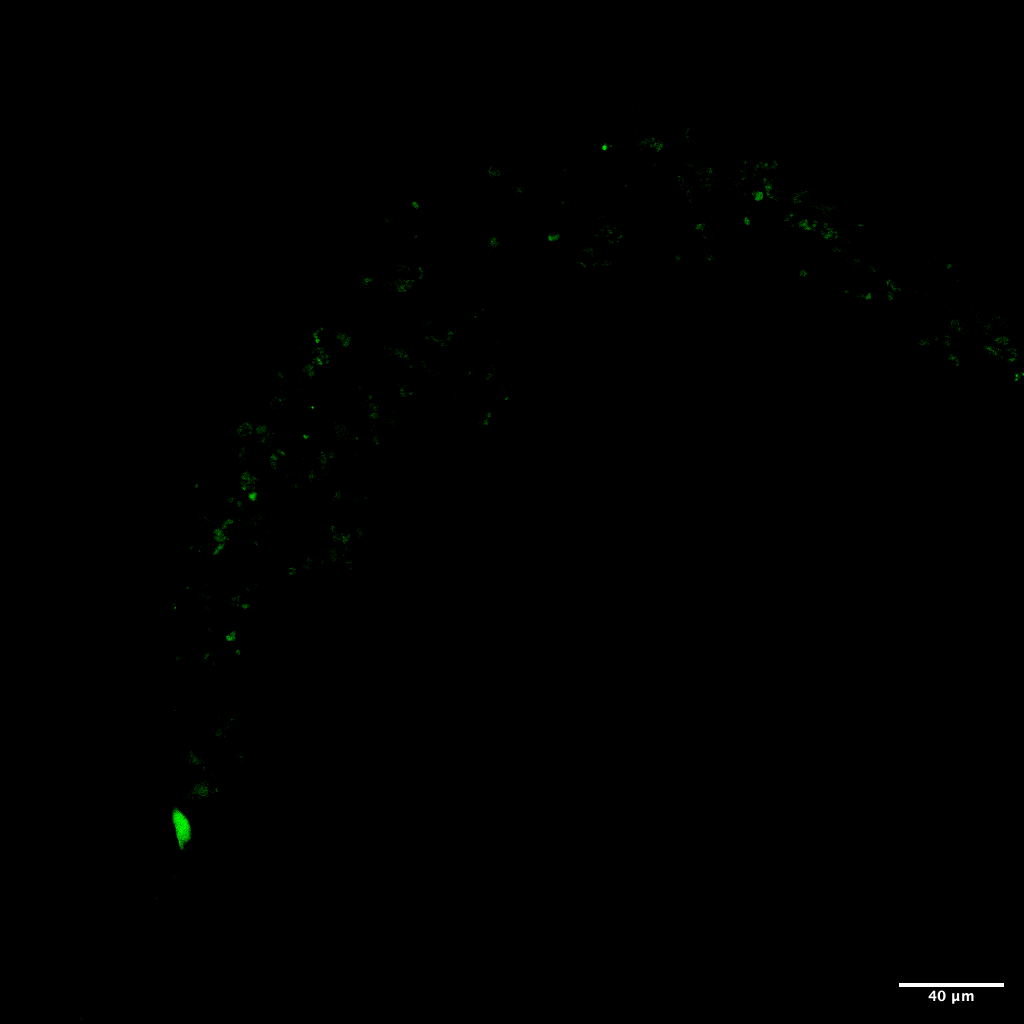

Supplement: Figure 5—source data 3. [file elife-83291-fig5-data3.zip › Figure5_sourcedata_timecourse_F66NR2#1/Day38_DAZL.png]

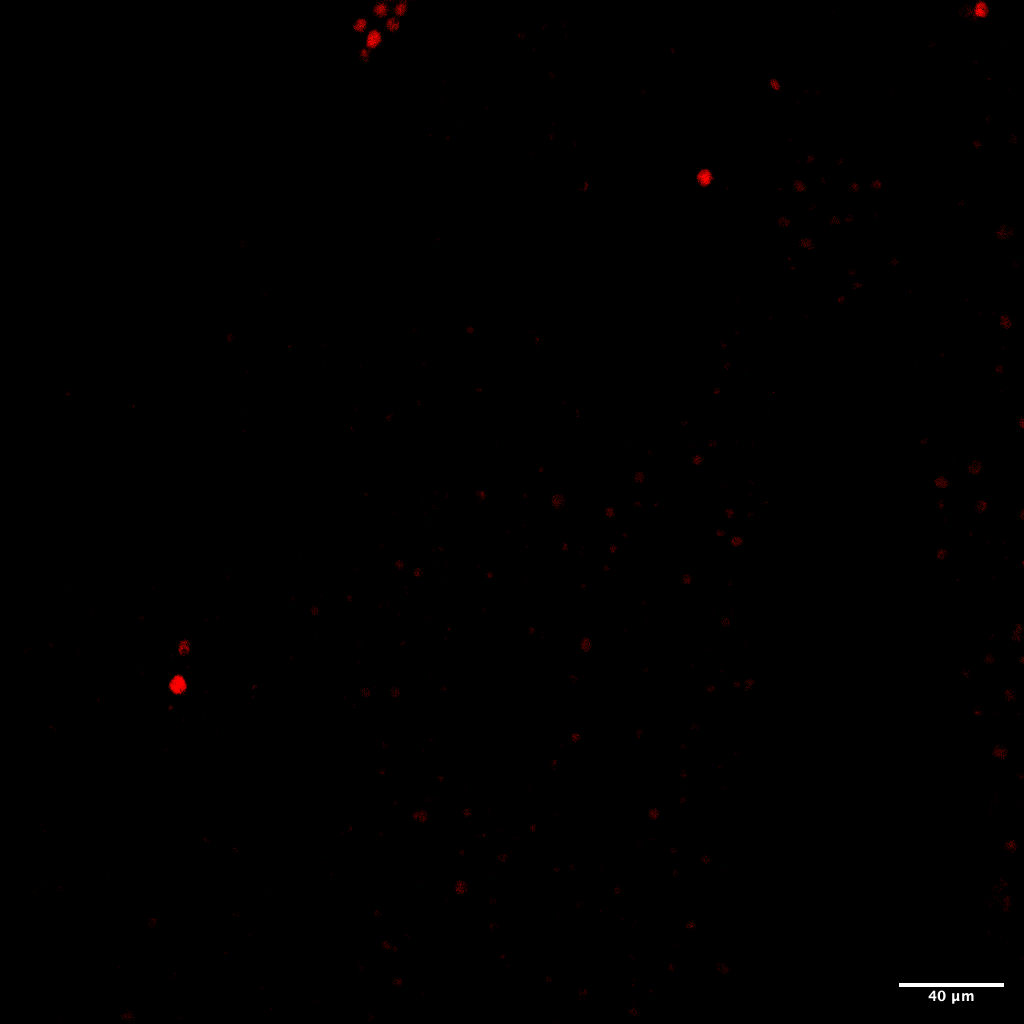

Supplement: Figure 5—source data 3. [file elife-83291-fig5-data3.zip › Figure5_sourcedata_timecourse_F66NR2#1/Day20_3_OCT4.png]

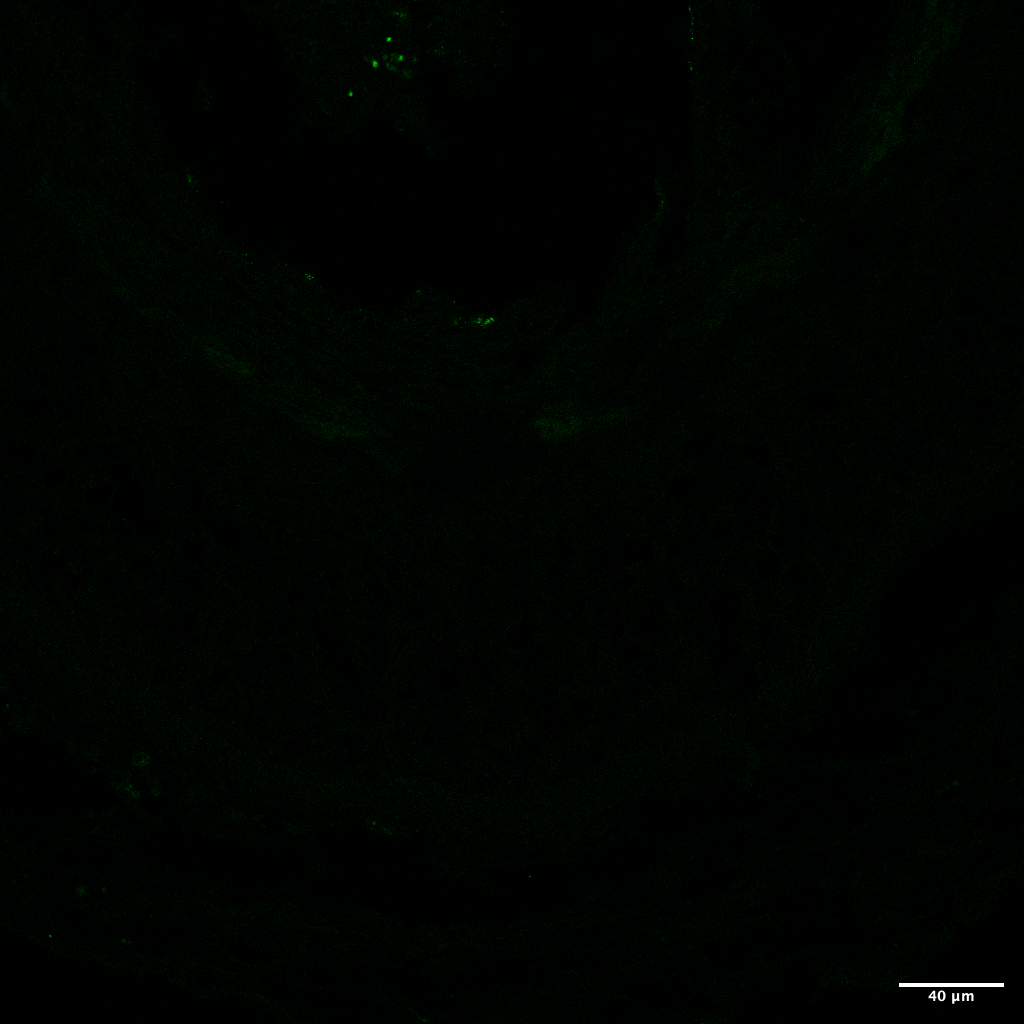

Supplement: Figure 5—source data 3. [file elife-83291-fig5-data3.zip › Figure5_sourcedata_timecourse_F66NR2#1/Day70_DAZL.png]

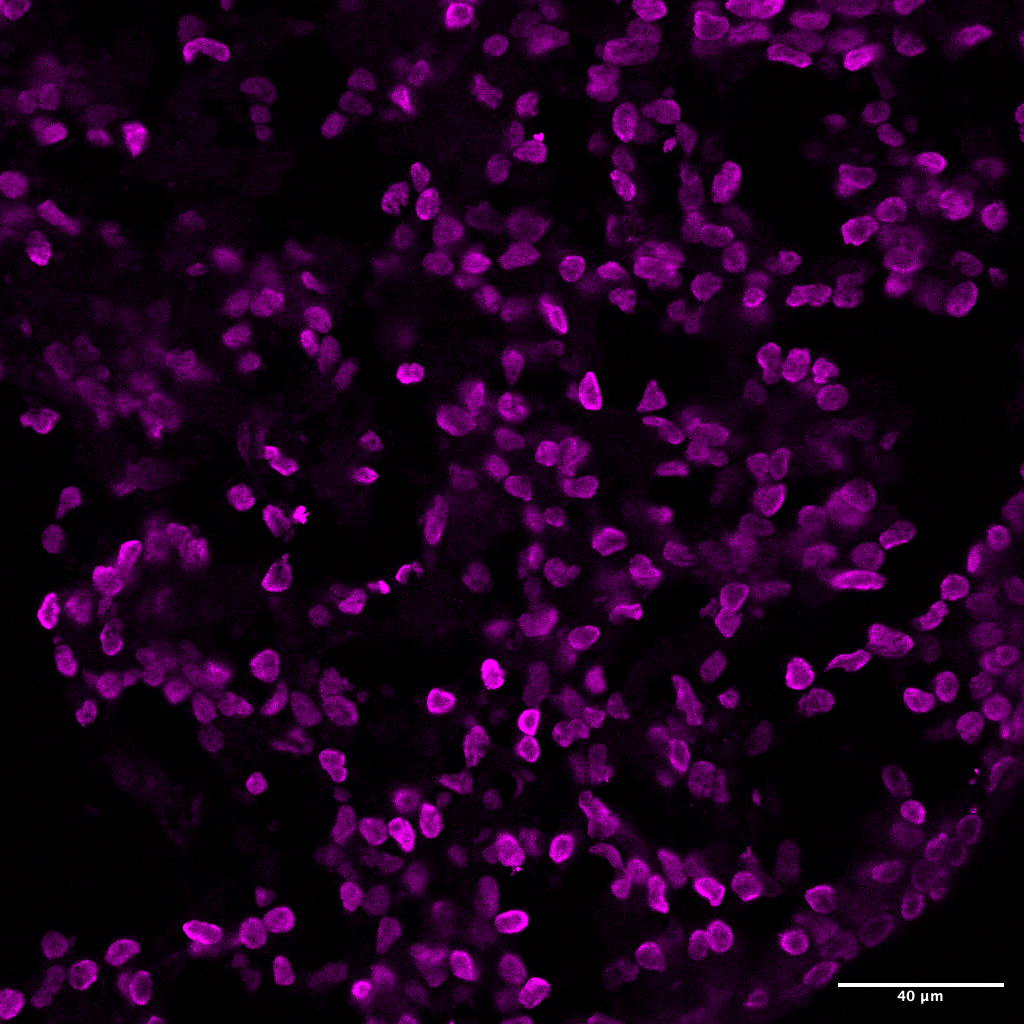

Supplement: Figure 5—source data 3. [file elife-83291-fig5-data3.zip › Figure5_sourcedata_timecourse_F66NR2#1/Day2-_A1_FOXL2.png]

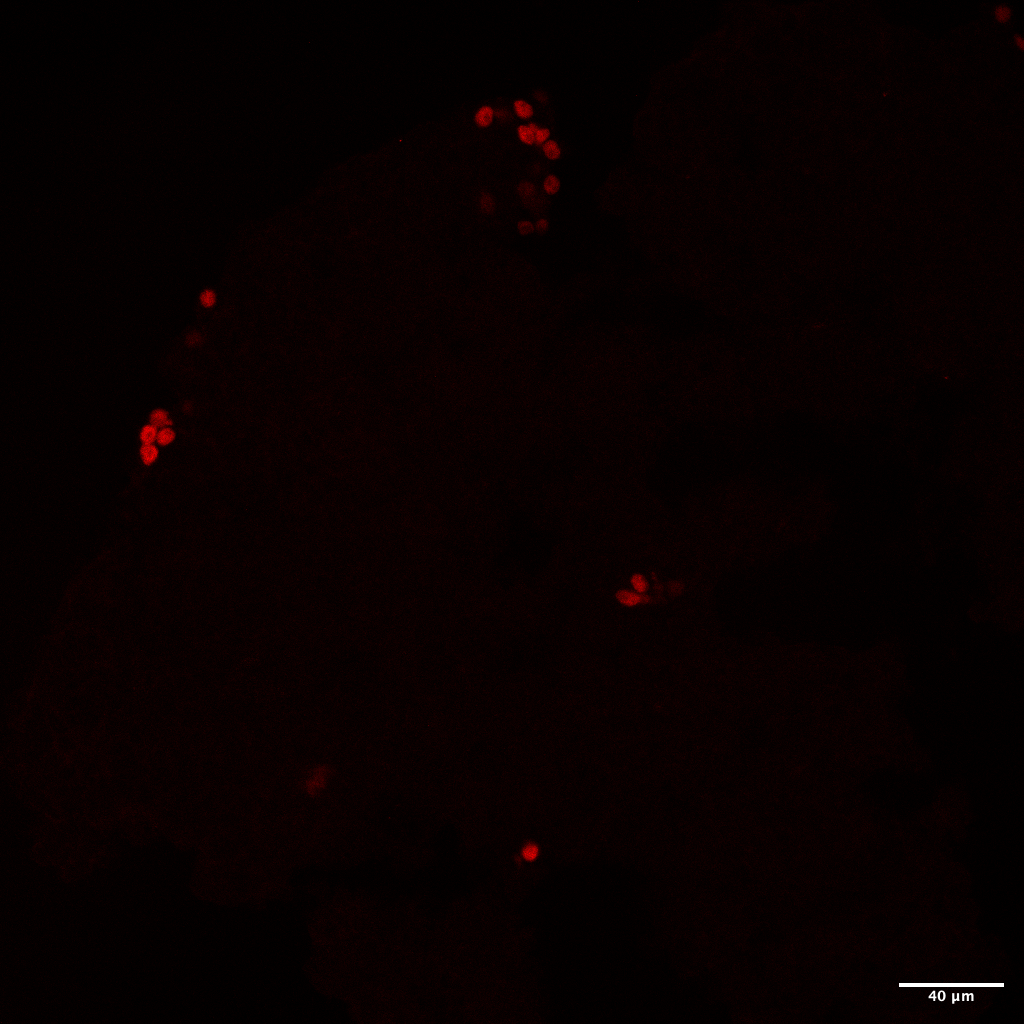

Supplement: Figure 5—source data 3. [file elife-83291-fig5-data3.zip › Figure5_sourcedata_timecourse_F66NR2#1/Day8OCT4.png]

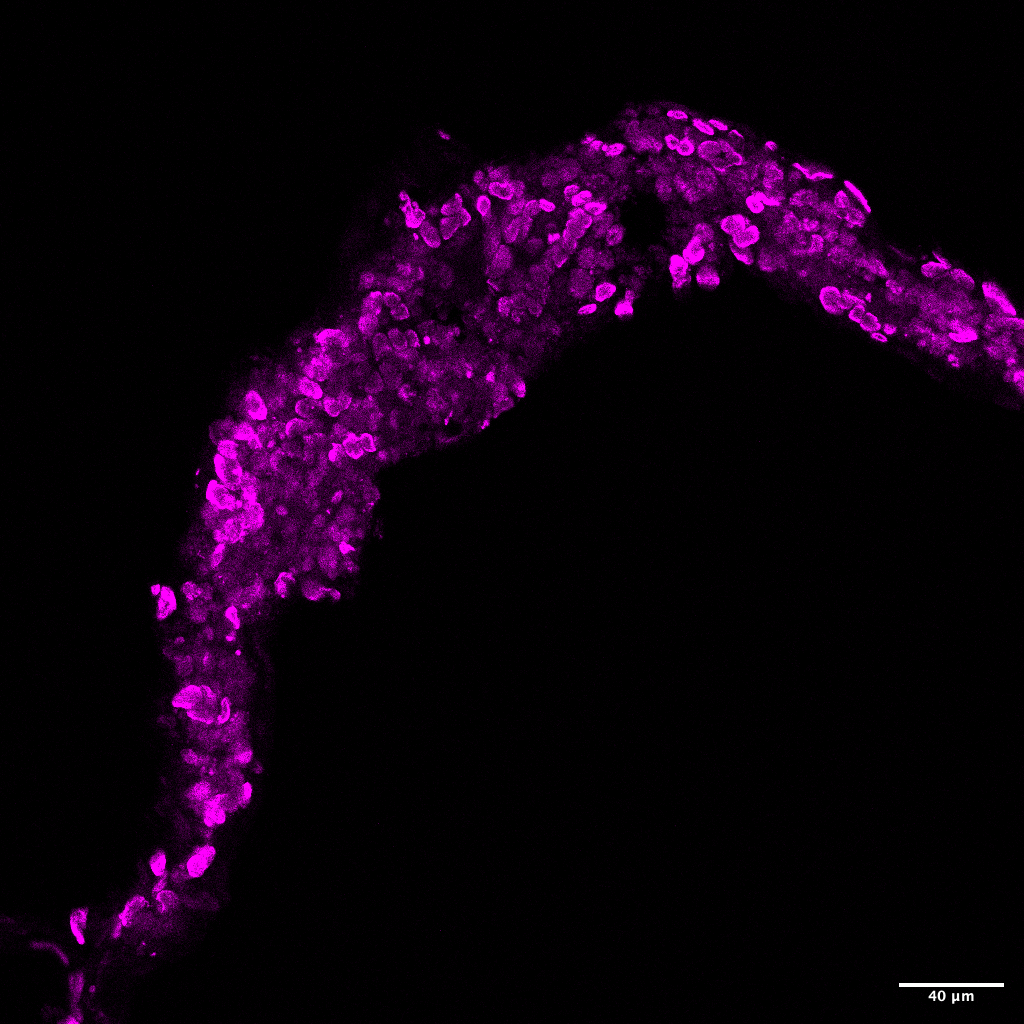

Supplement: Figure 5—source data 3. [file elife-83291-fig5-data3.zip › Figure5_sourcedata_timecourse_F66NR2#1/Day38_FOXL2.png]

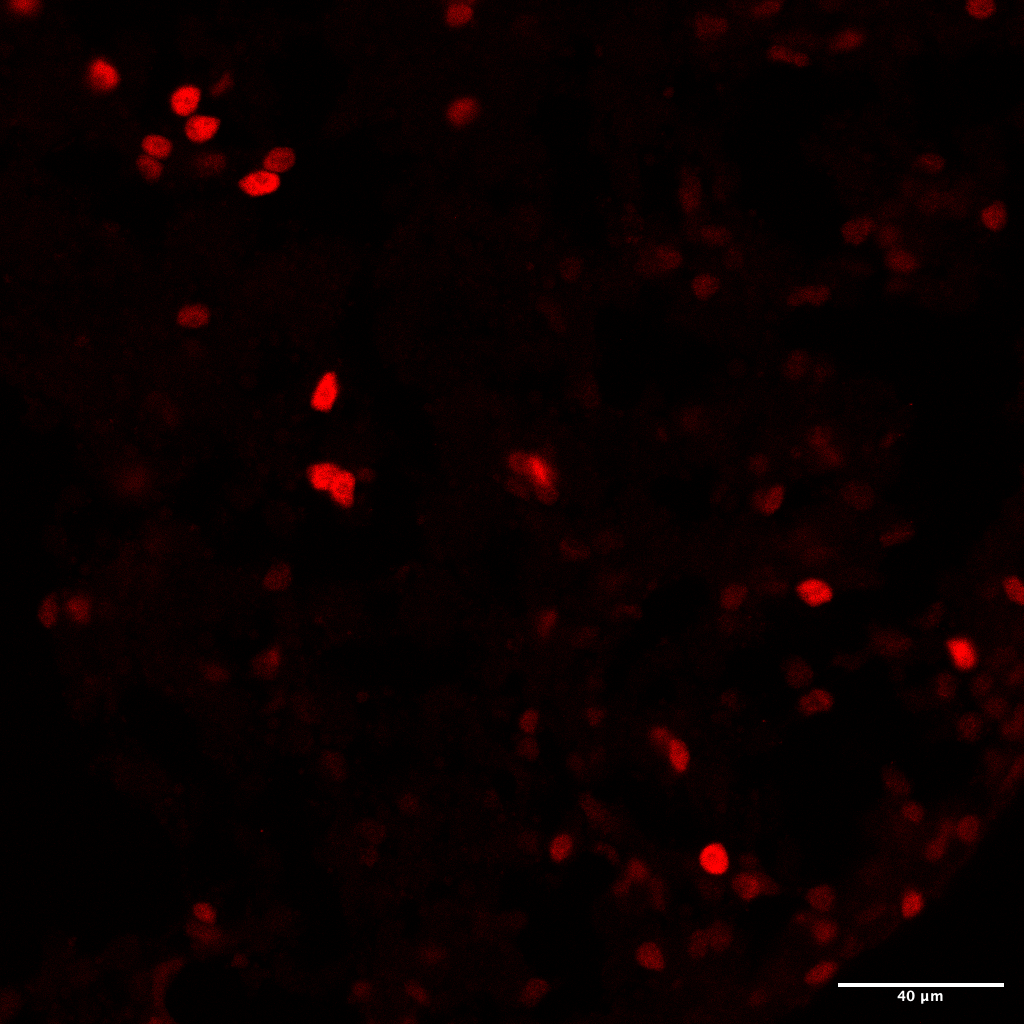

Supplement: Figure 5—source data 3. [file elife-83291-fig5-data3.zip › Figure5_sourcedata_timecourse_F66NR2#1/Day2-_A1_OCT4.png]

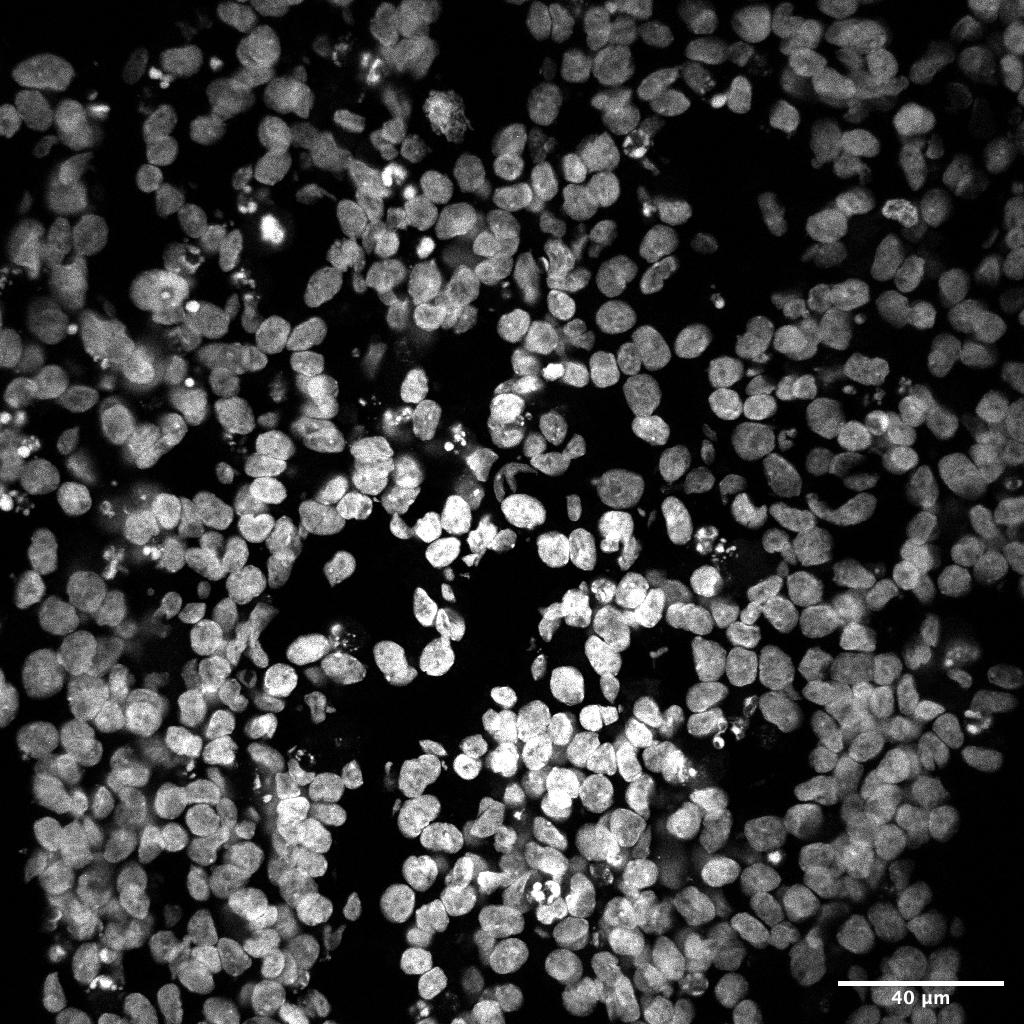

Supplement: Figure 5—source data 3. [file elife-83291-fig5-data3.zip › Figure5_sourcedata_timecourse_F66NR2#1/Day4_DAPI.png]

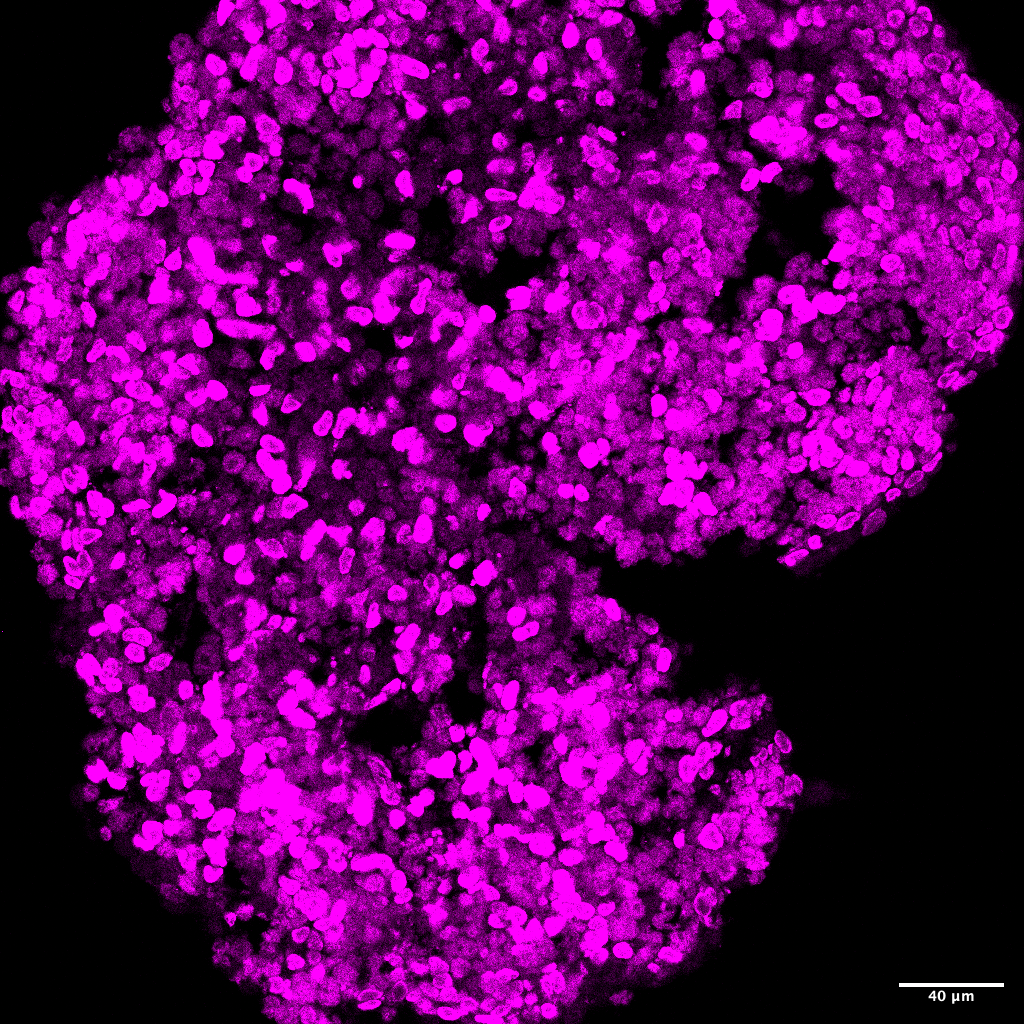

Supplement: Figure 5—source data 3. [file elife-83291-fig5-data3.zip › Figure5_sourcedata_timecourse_F66NR2#1/Day14_FOXL2.png]

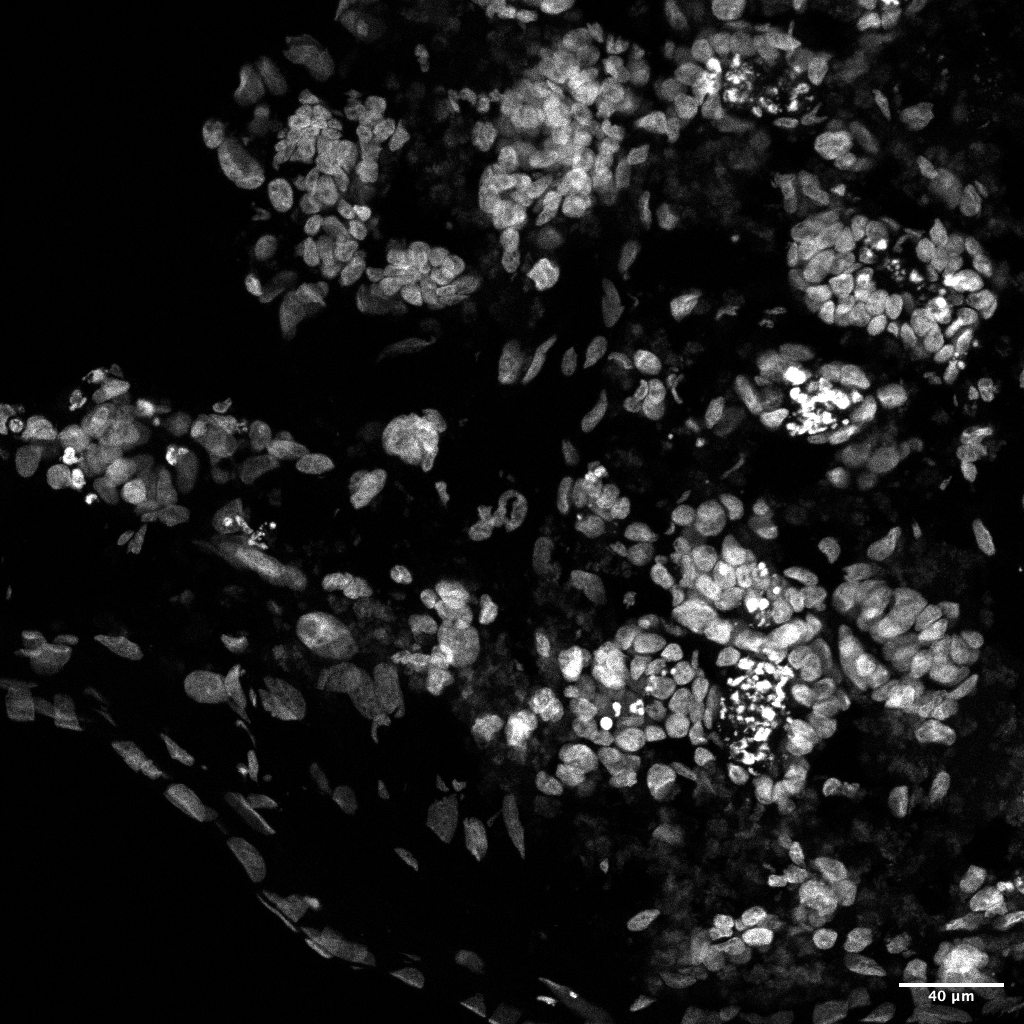

Supplement: Figure 5—source data 3. [file elife-83291-fig5-data3.zip › Figure5_sourcedata_timecourse_F66NR2#1/Day54_DAPI.png]

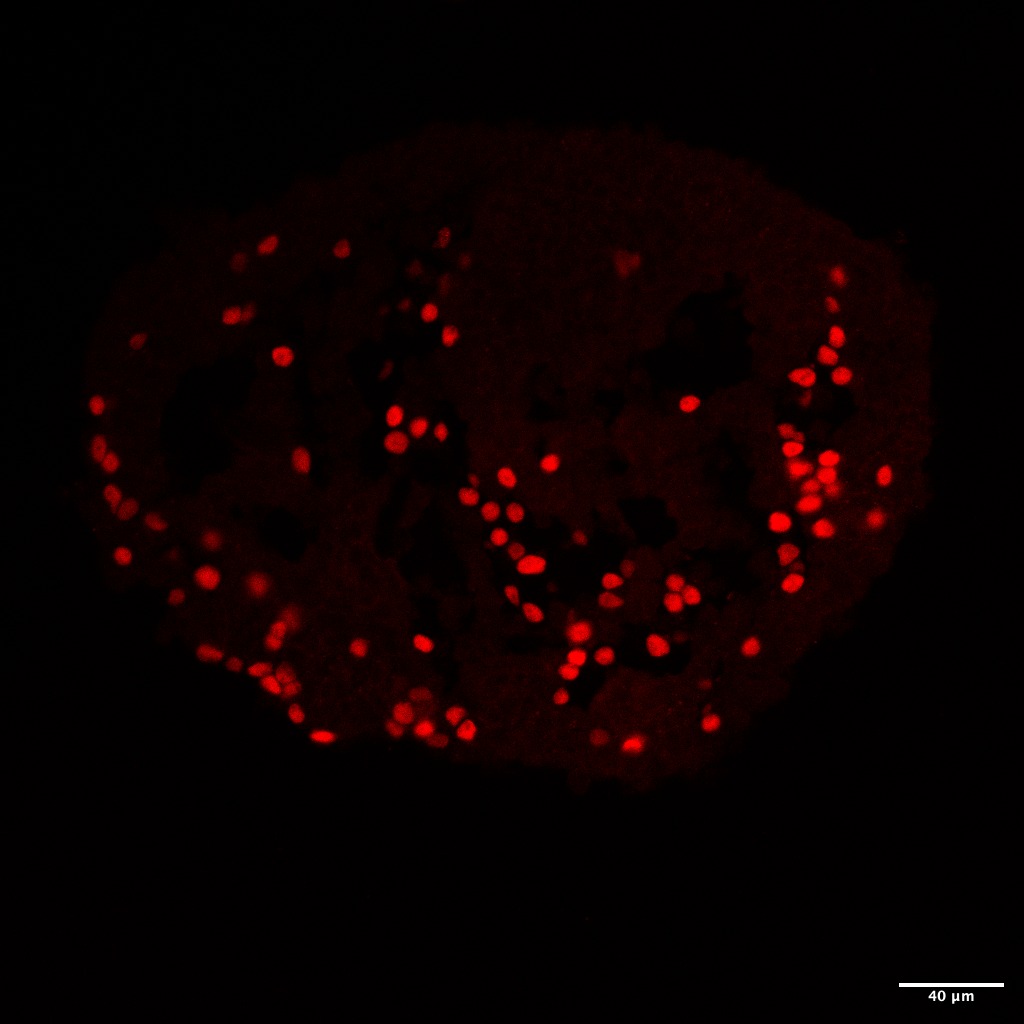

Supplement: Figure 5—source data 4. [file elife-83291-fig5-data4.zip › Figure5_sourcedata_timecourse_mouse/mouse_day2_OCT4.png]

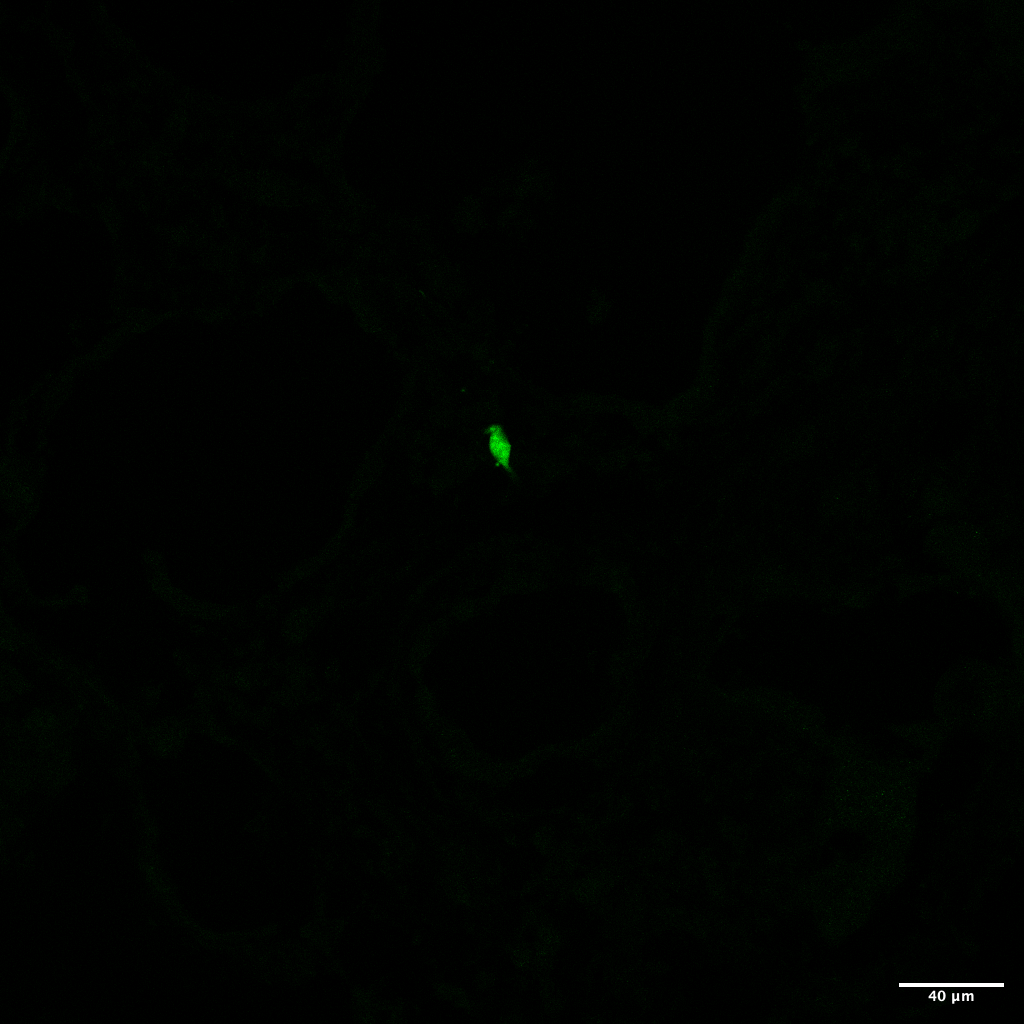

Supplement: Figure 5—source data 4. [file elife-83291-fig5-data4.zip › Figure5_sourcedata_timecourse_mouse/mouse_day46_DAZL.png]

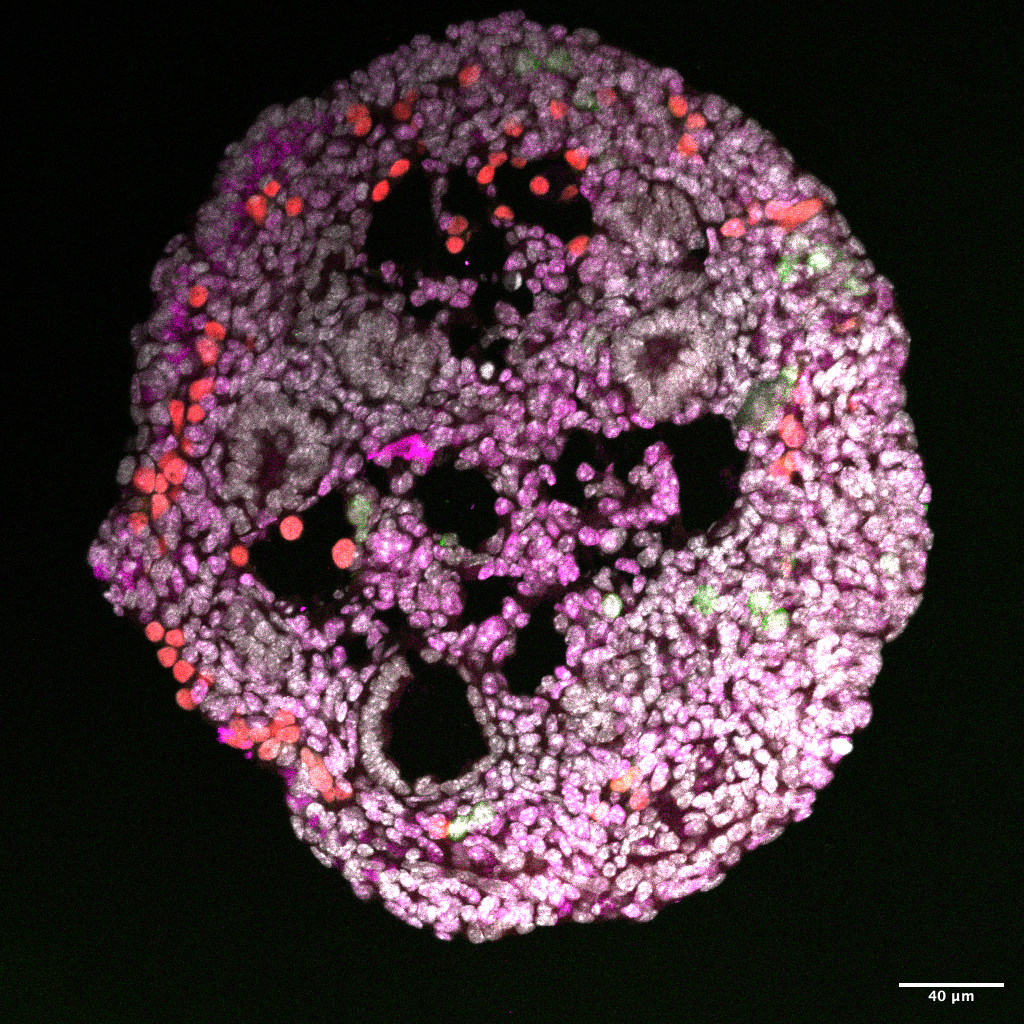

Supplement: Figure 5—source data 4. [file elife-83291-fig5-data4.zip › Figure5_sourcedata_timecourse_mouse/mouse_day4_composite.png]

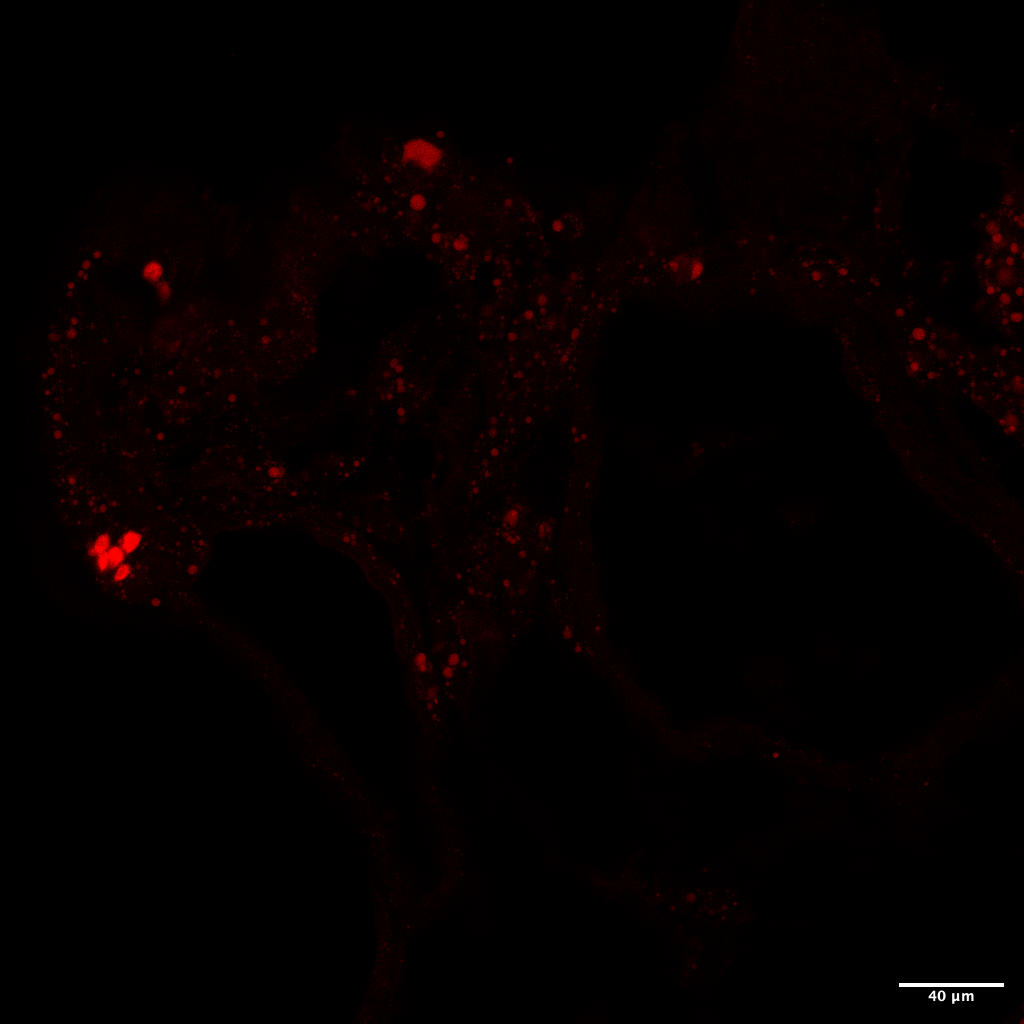

Supplement: Figure 5—source data 4. [file elife-83291-fig5-data4.zip › Figure5_sourcedata_timecourse_mouse/mouse_day54_OCT4.png]

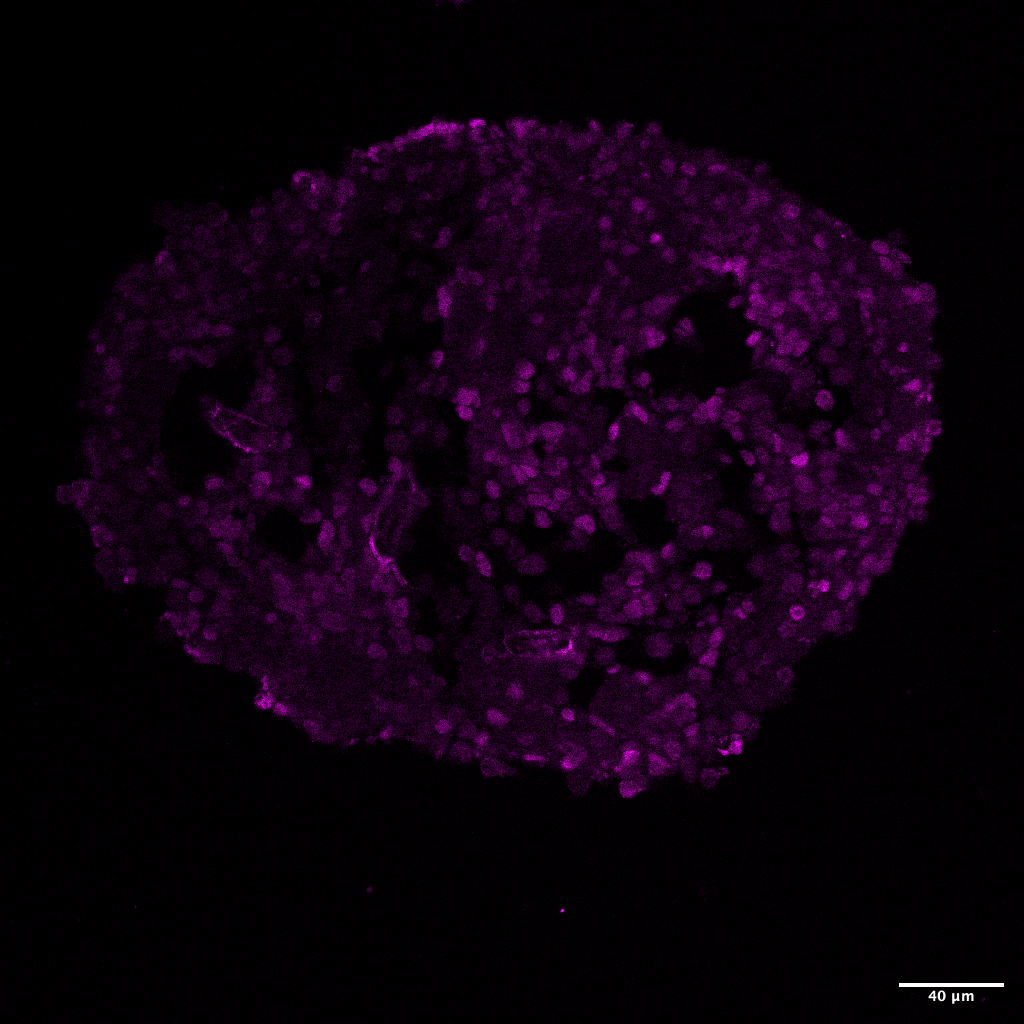

Supplement: Figure 5—source data 4. [file elife-83291-fig5-data4.zip › Figure5_sourcedata_timecourse_mouse/mouse_day2_FOXL2.png]

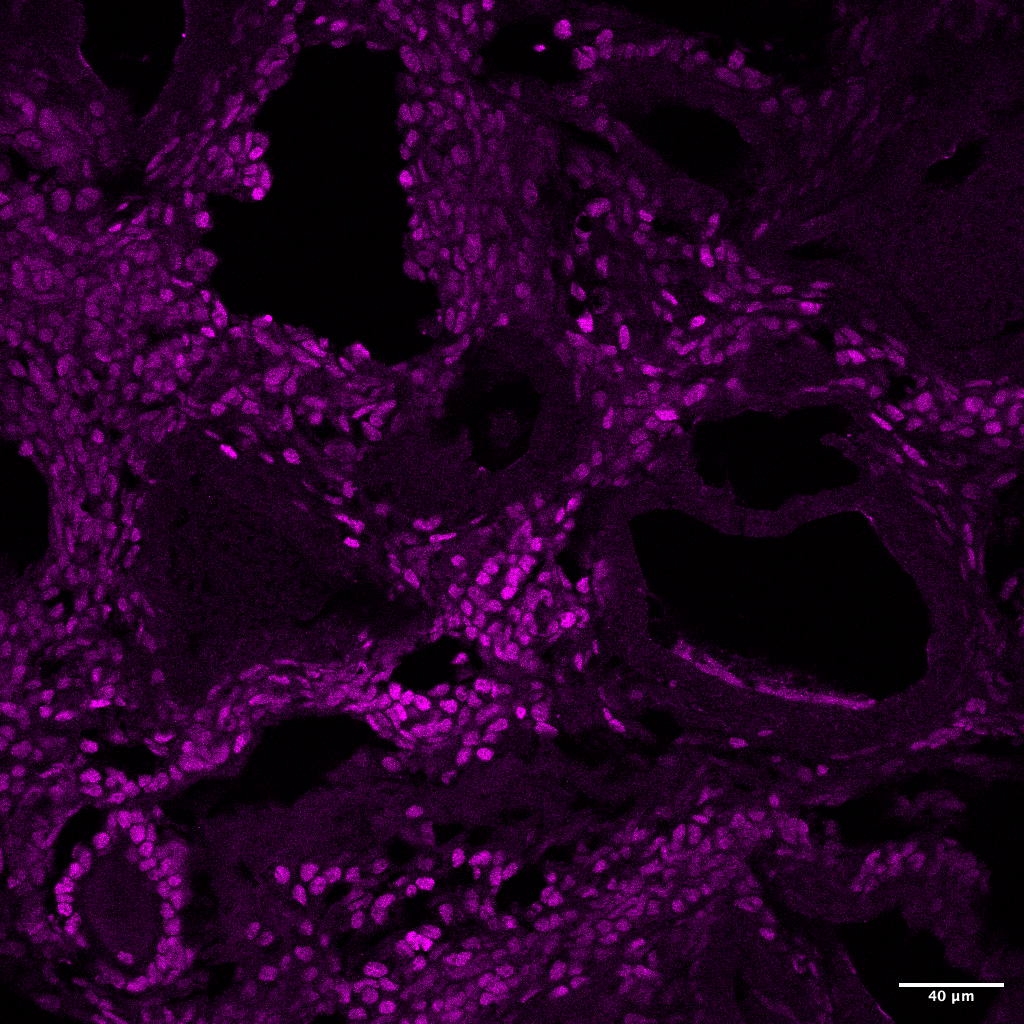

Supplement: Figure 5—source data 4. [file elife-83291-fig5-data4.zip › Figure5_sourcedata_timecourse_mouse/mouse_day26_FOXL2.png]

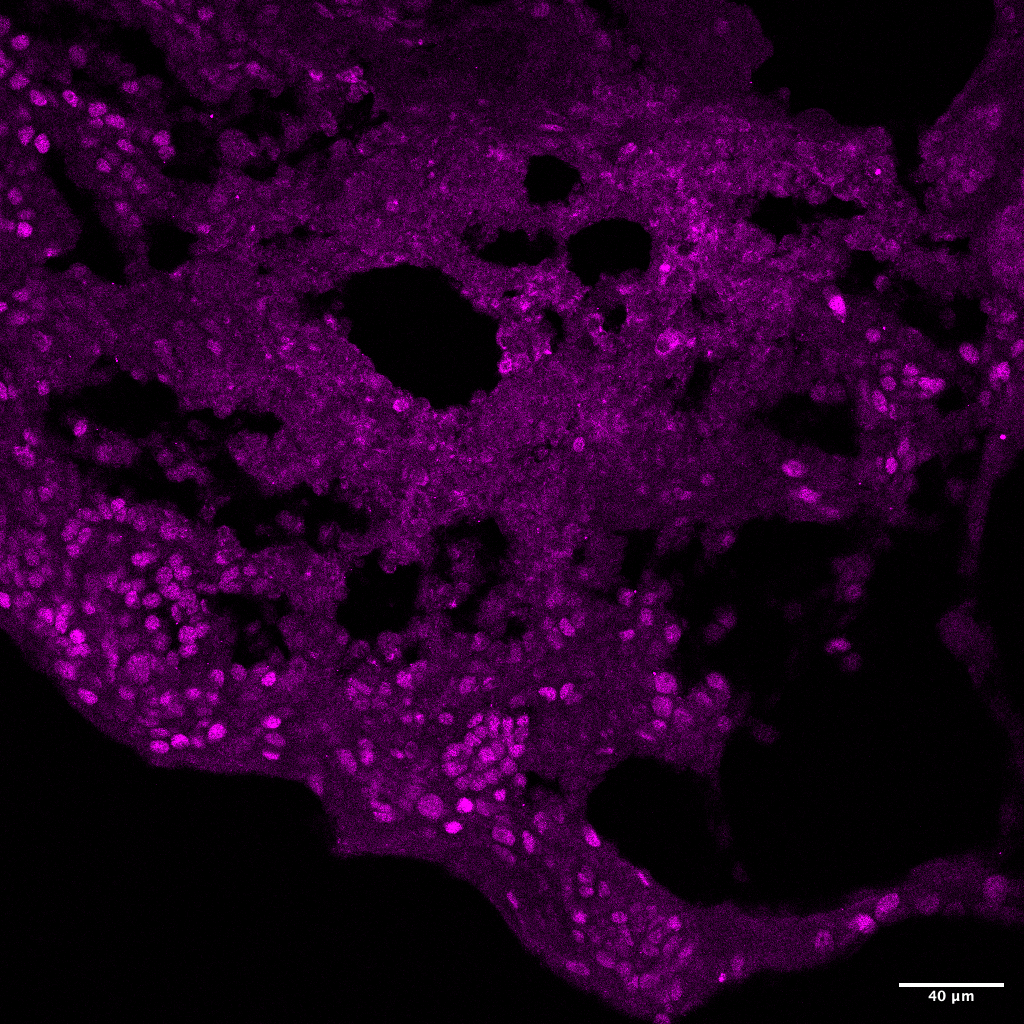

Supplement: Figure 5—source data 4. [file elife-83291-fig5-data4.zip › Figure5_sourcedata_timecourse_mouse/mouse_day32_FOXL2.png]

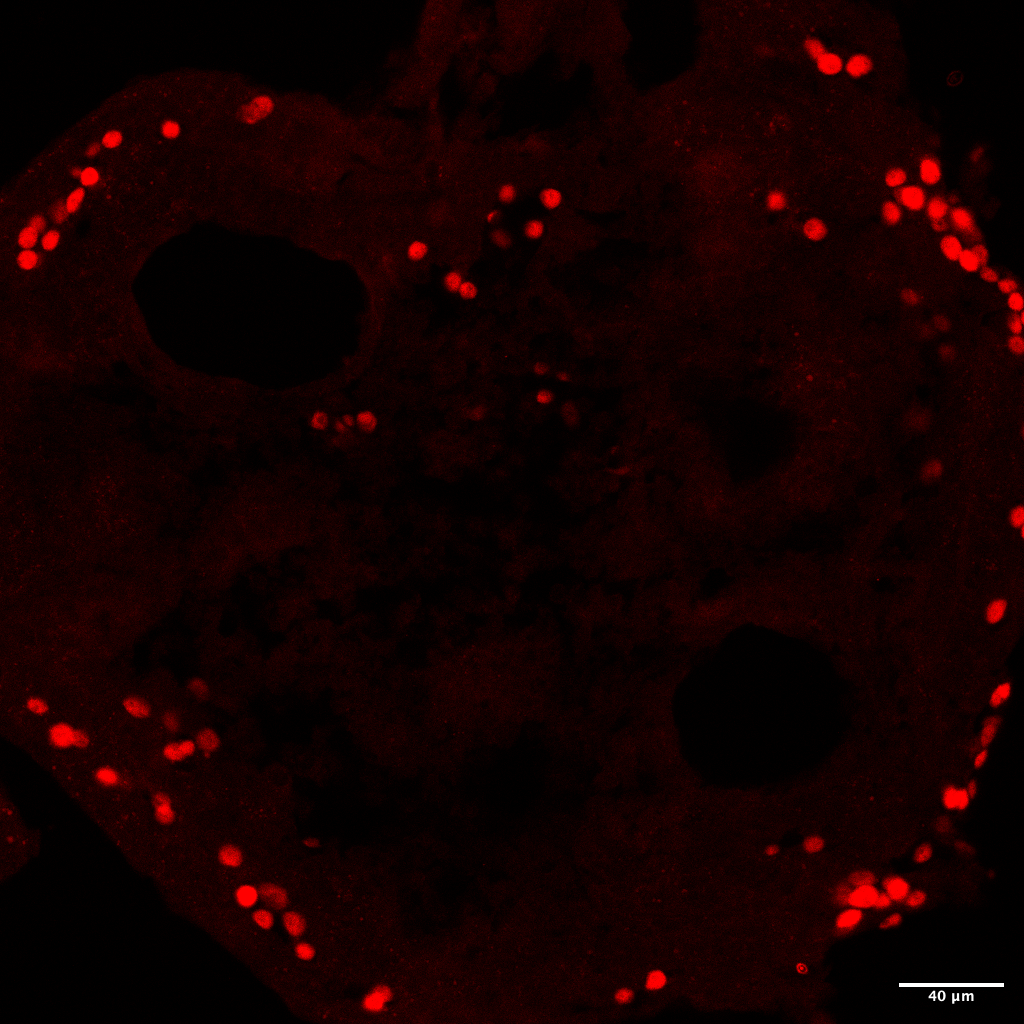

Supplement: Figure 5—source data 4. [file elife-83291-fig5-data4.zip › Figure5_sourcedata_timecourse_mouse/mouse_day8_OCT4.png]

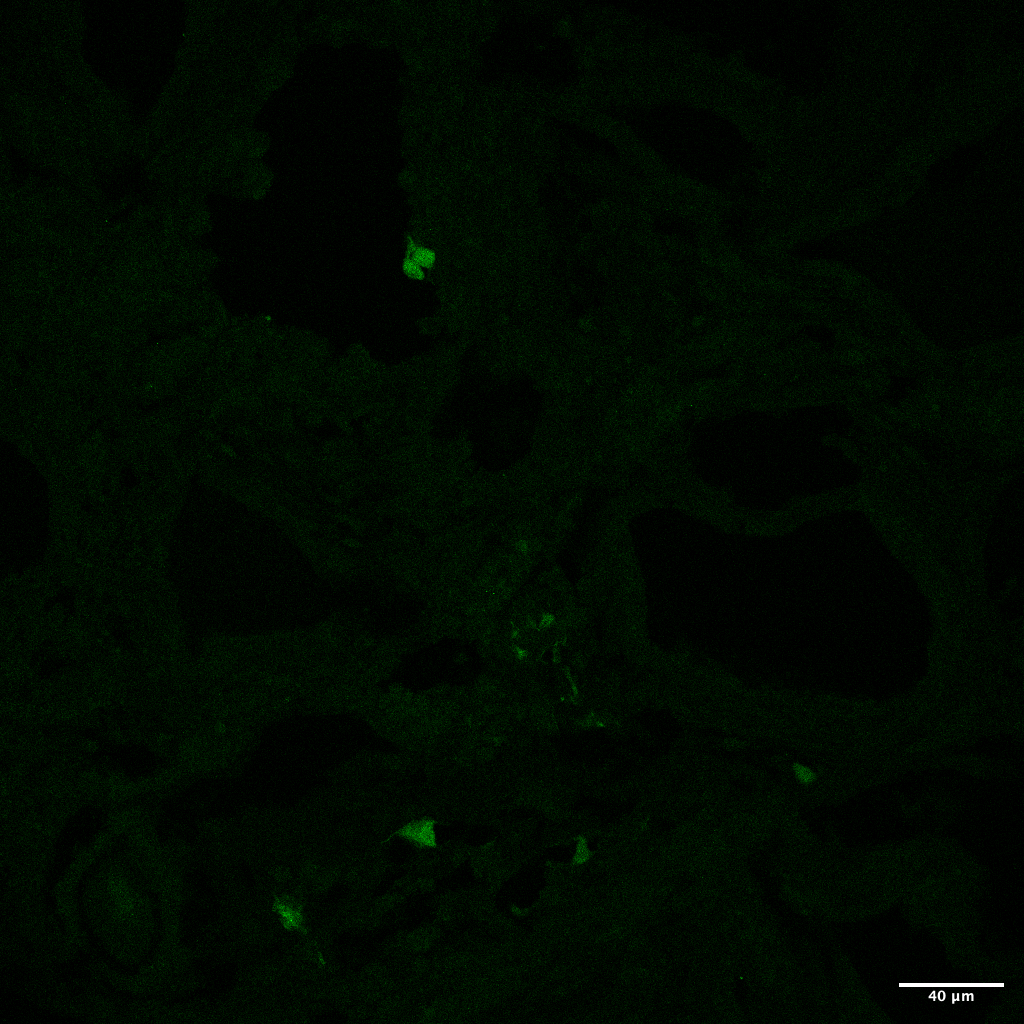

Supplement: Figure 5—source data 4. [file elife-83291-fig5-data4.zip › Figure5_sourcedata_timecourse_mouse/mouse_day26_DAZL.png]

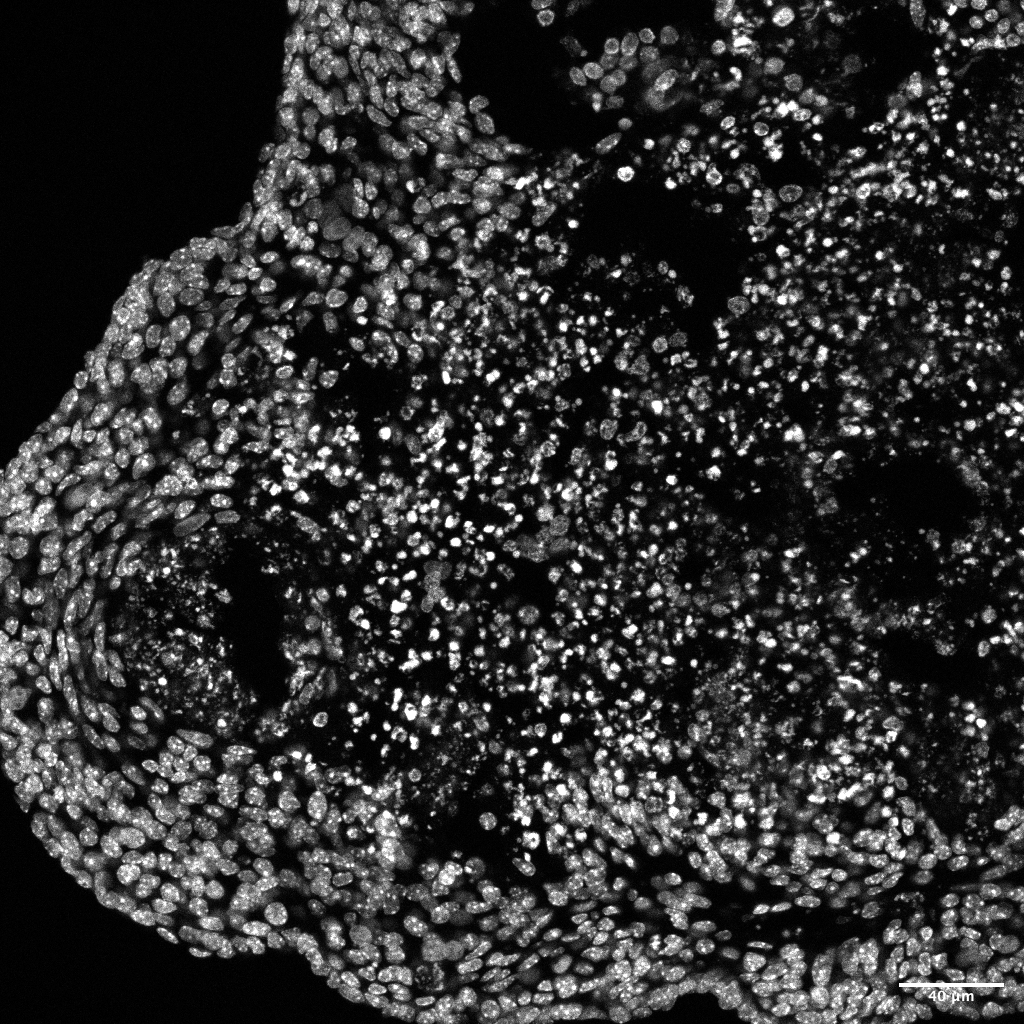

Supplement: Figure 5—source data 4. [file elife-83291-fig5-data4.zip › Figure5_sourcedata_timecourse_mouse/mouse_day14_DAPI.png]

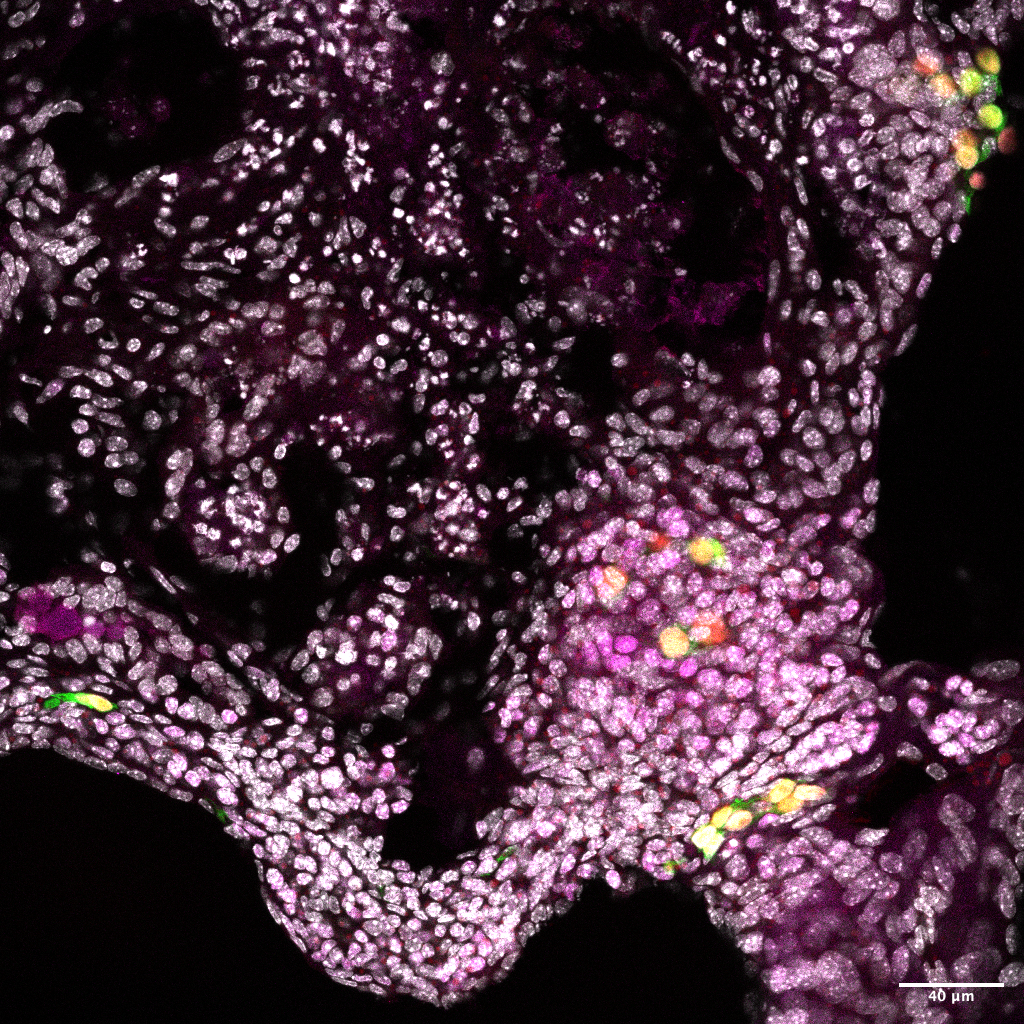

Supplement: Figure 5—source data 4. [file elife-83291-fig5-data4.zip › Figure5_sourcedata_timecourse_mouse/mouse_day38_composite.png]

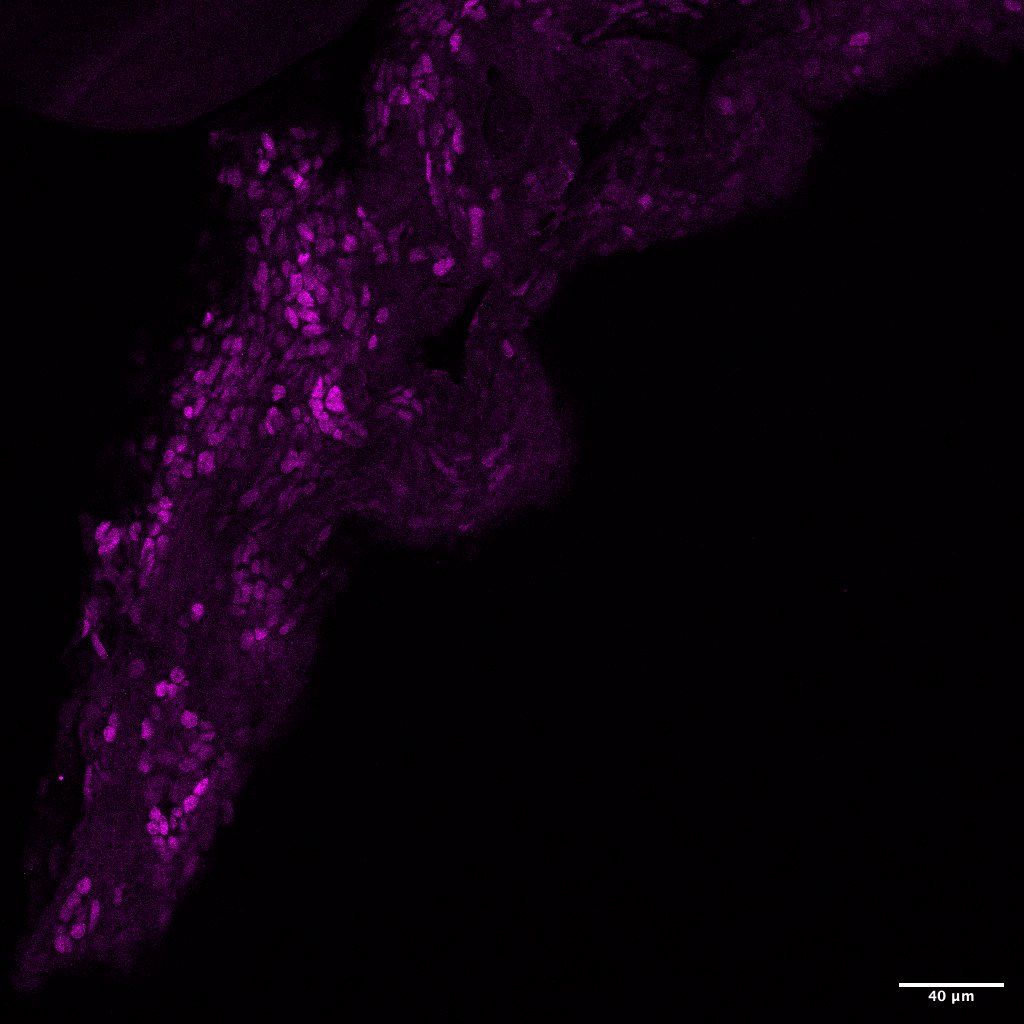

Supplement: Figure 5—source data 4. [file elife-83291-fig5-data4.zip › Figure5_sourcedata_timecourse_mouse/mouse_day20_FOXL2.png]

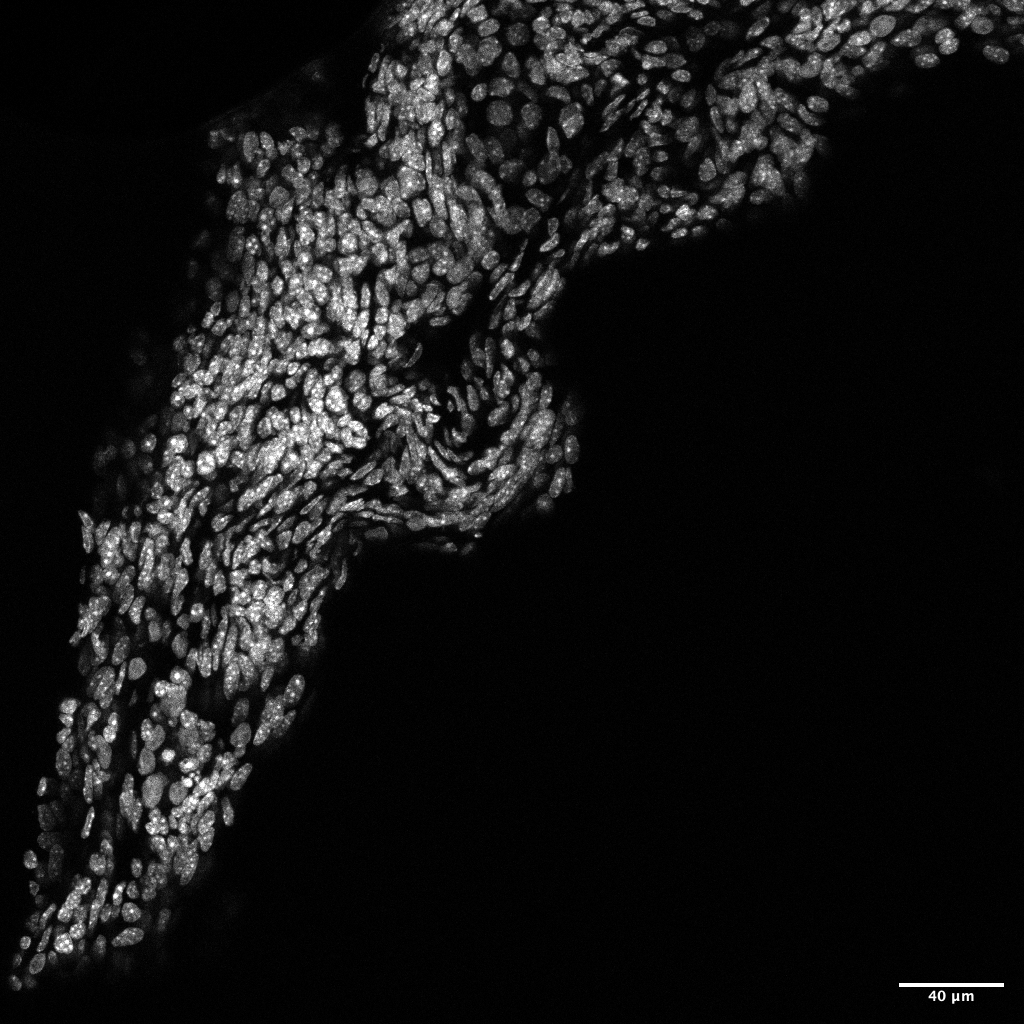

Supplement: Figure 5—source data 4. [file elife-83291-fig5-data4.zip › Figure5_sourcedata_timecourse_mouse/mouse_day20_DAPI.png]

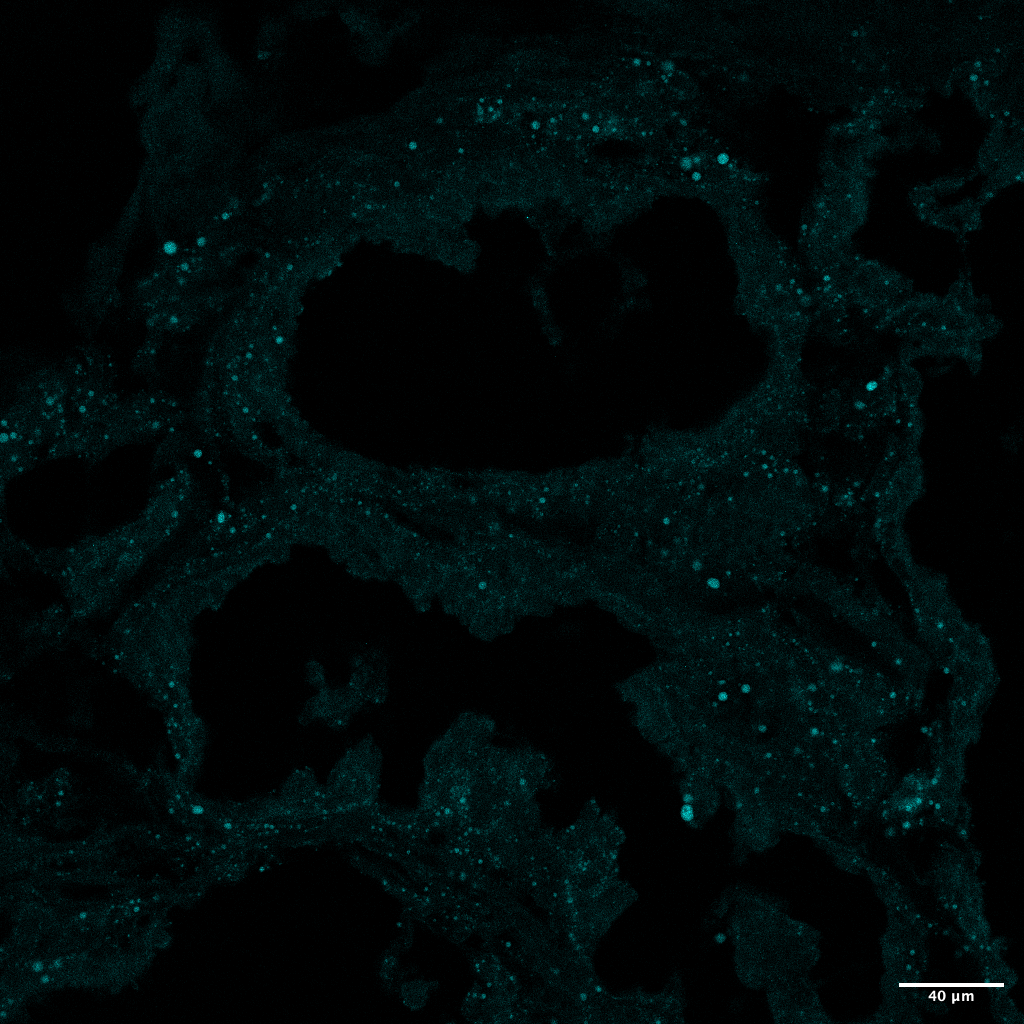

Supplement: Figure 5—source data 4. [file elife-83291-fig5-data4.zip › Figure5_sourcedata_timecourse_mouse/Day70_OCT4.png]

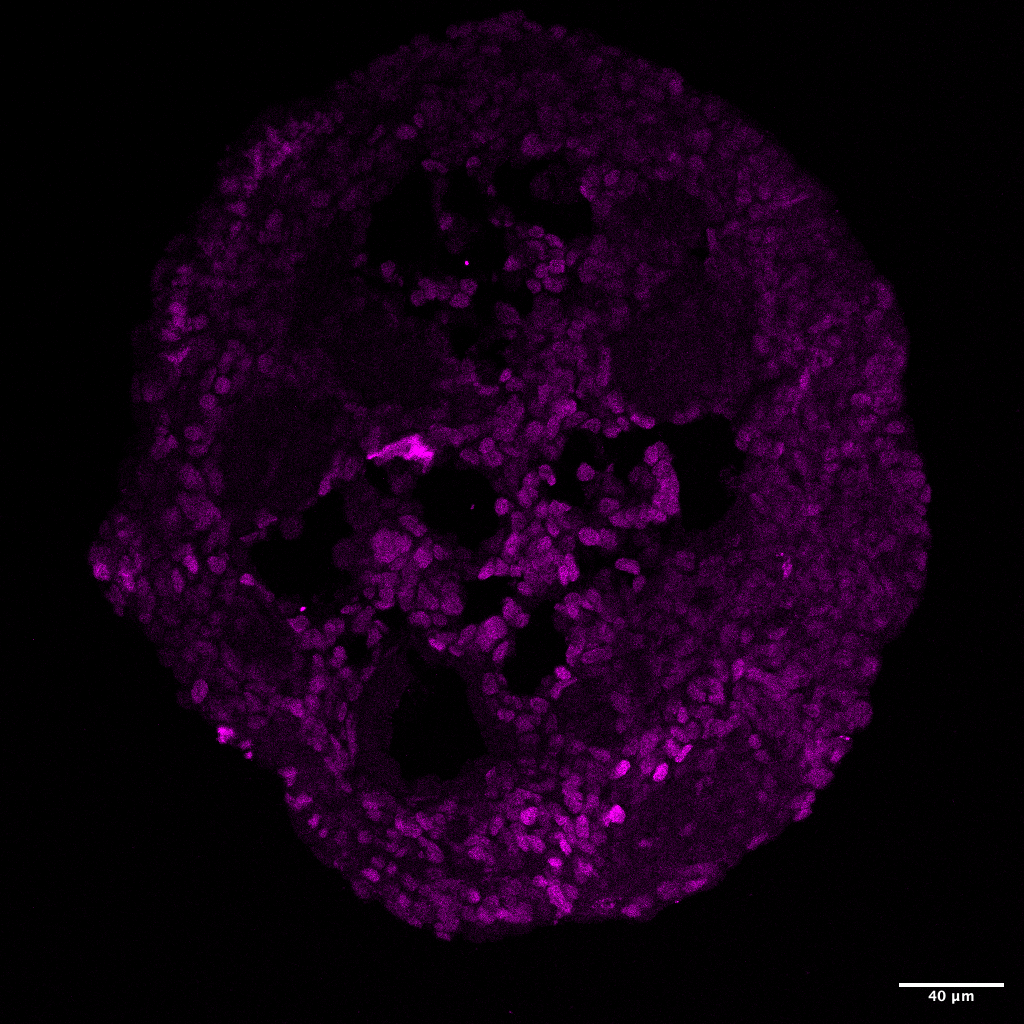

Supplement: Figure 5—source data 4. [file elife-83291-fig5-data4.zip › Figure5_sourcedata_timecourse_mouse/mouse_day4_FOXL2.png]

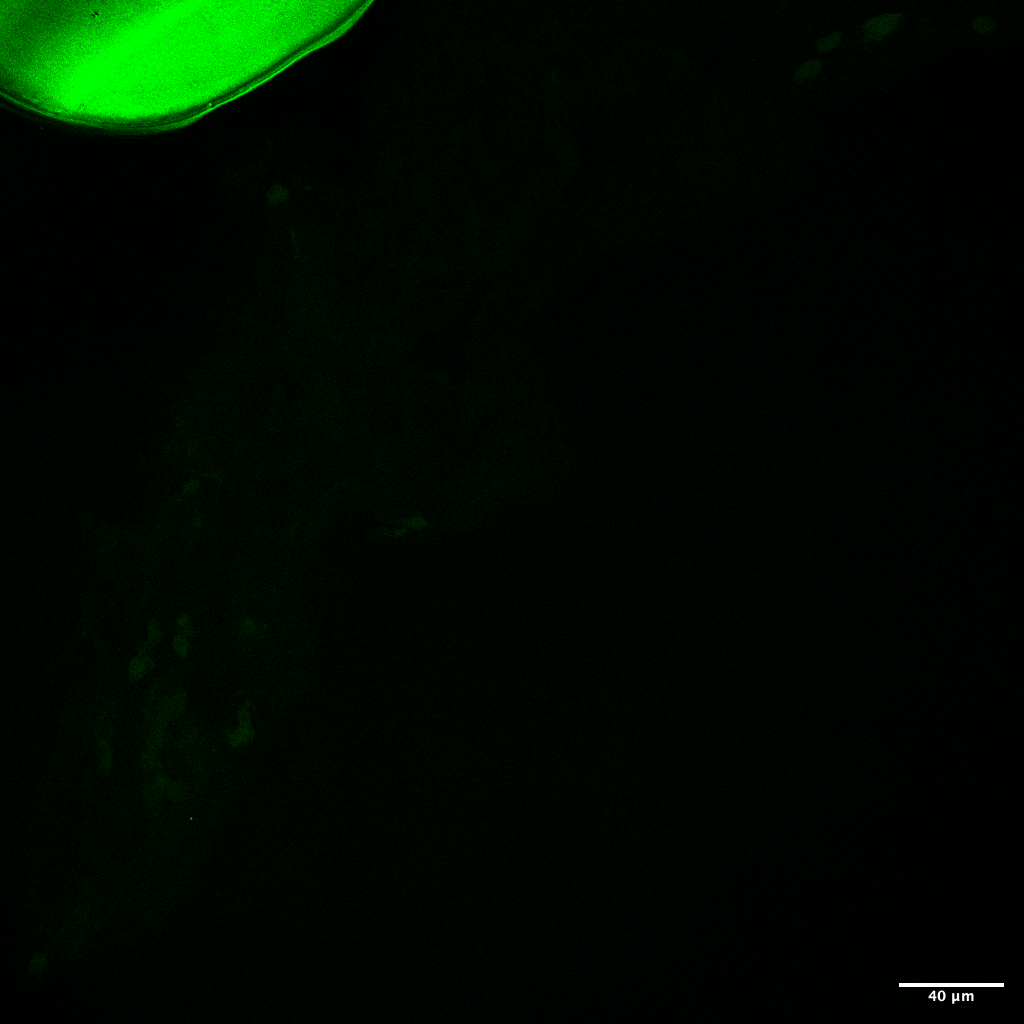

Supplement: Figure 5—source data 4. [file elife-83291-fig5-data4.zip › Figure5_sourcedata_timecourse_mouse/mouse_day20_DAZL.png]

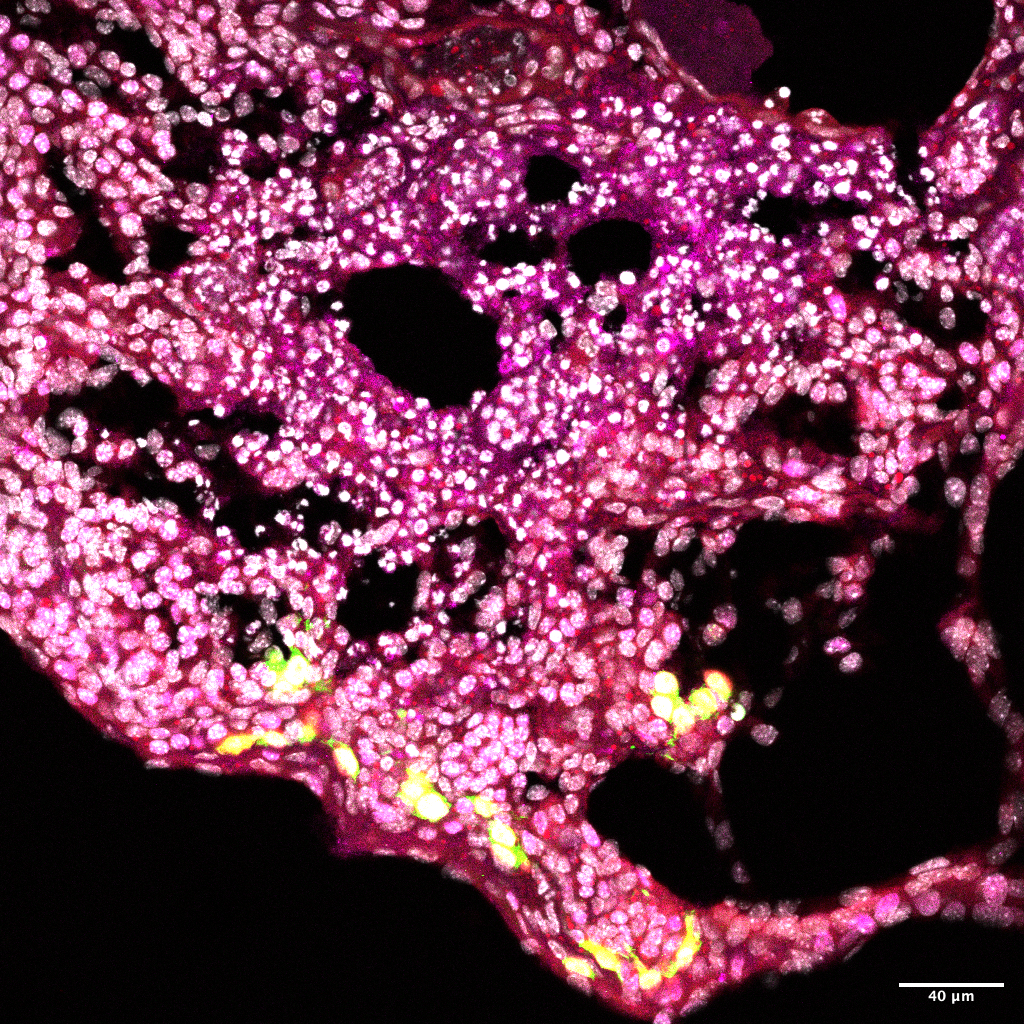

Supplement: Figure 5—source data 4. [file elife-83291-fig5-data4.zip › Figure5_sourcedata_timecourse_mouse/mouse_day32_composite.png]

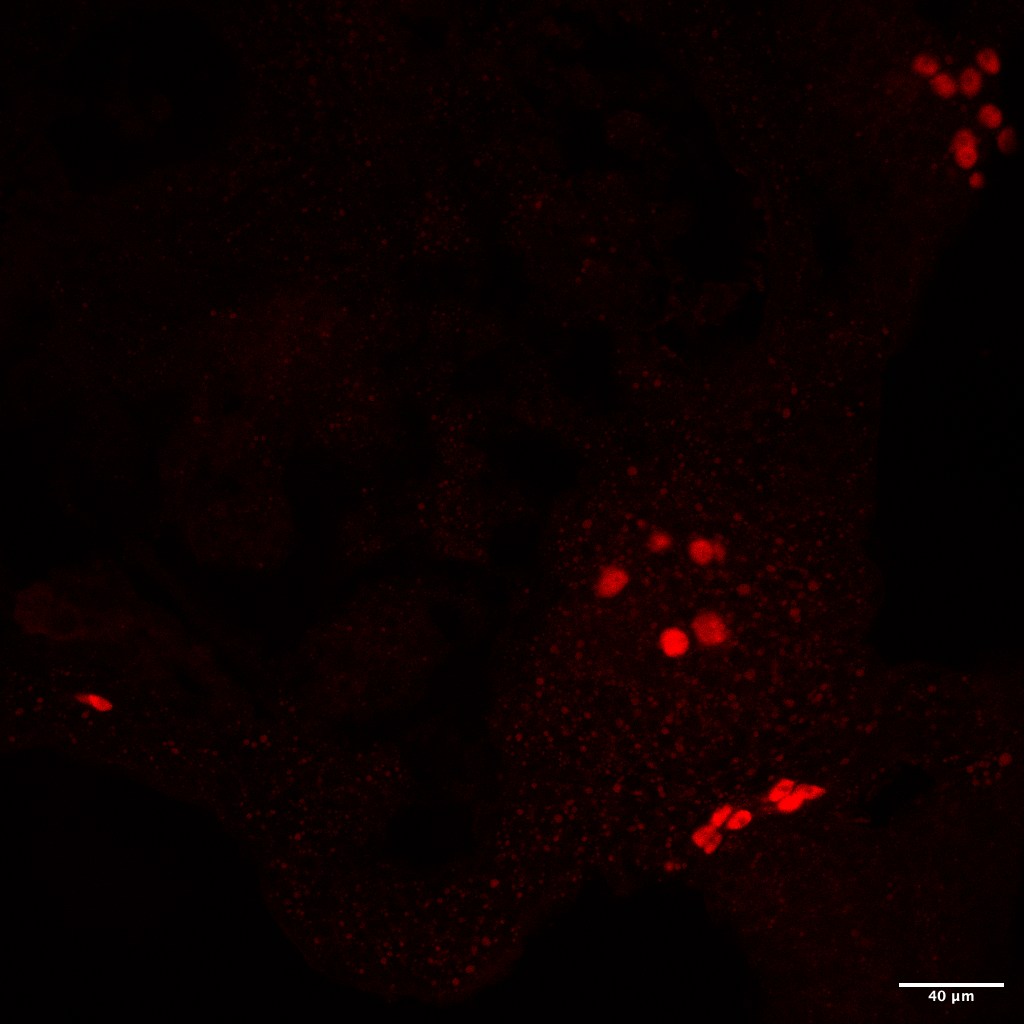

Supplement: Figure 5—source data 4. [file elife-83291-fig5-data4.zip › Figure5_sourcedata_timecourse_mouse/mouse_day38_OCT4.png]

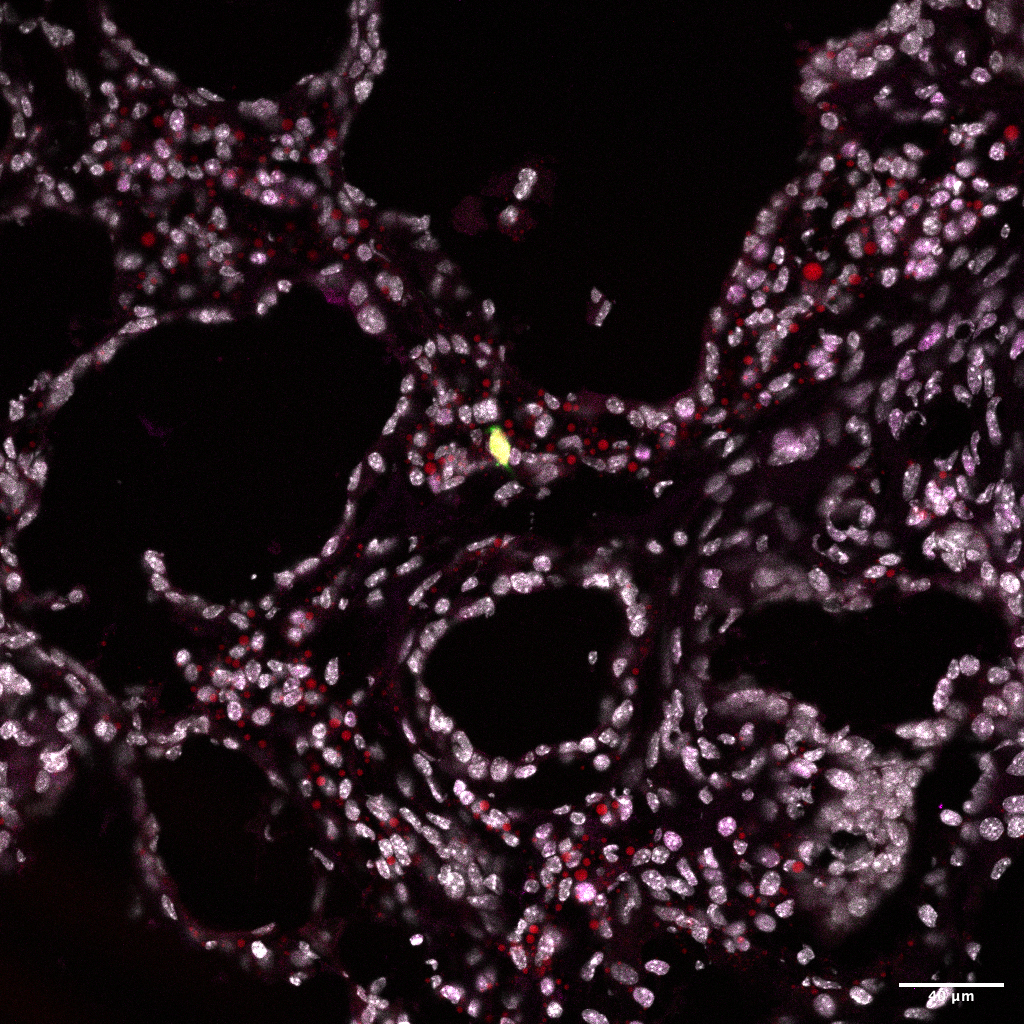

Supplement: Figure 5—source data 4. [file elife-83291-fig5-data4.zip › Figure5_sourcedata_timecourse_mouse/mouse_day46_composite.png]

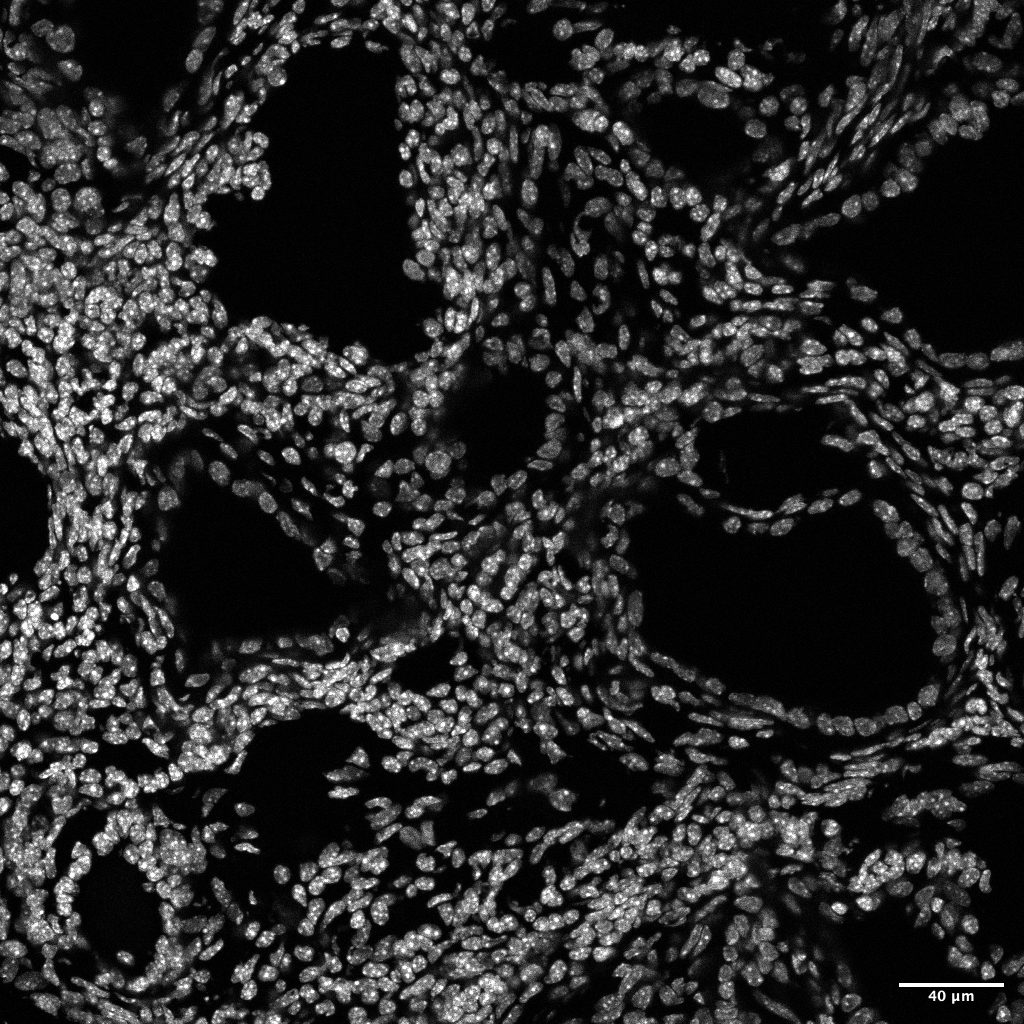

Supplement: Figure 5—source data 4. [file elife-83291-fig5-data4.zip › Figure5_sourcedata_timecourse_mouse/mouse_day26_DAPI.png]

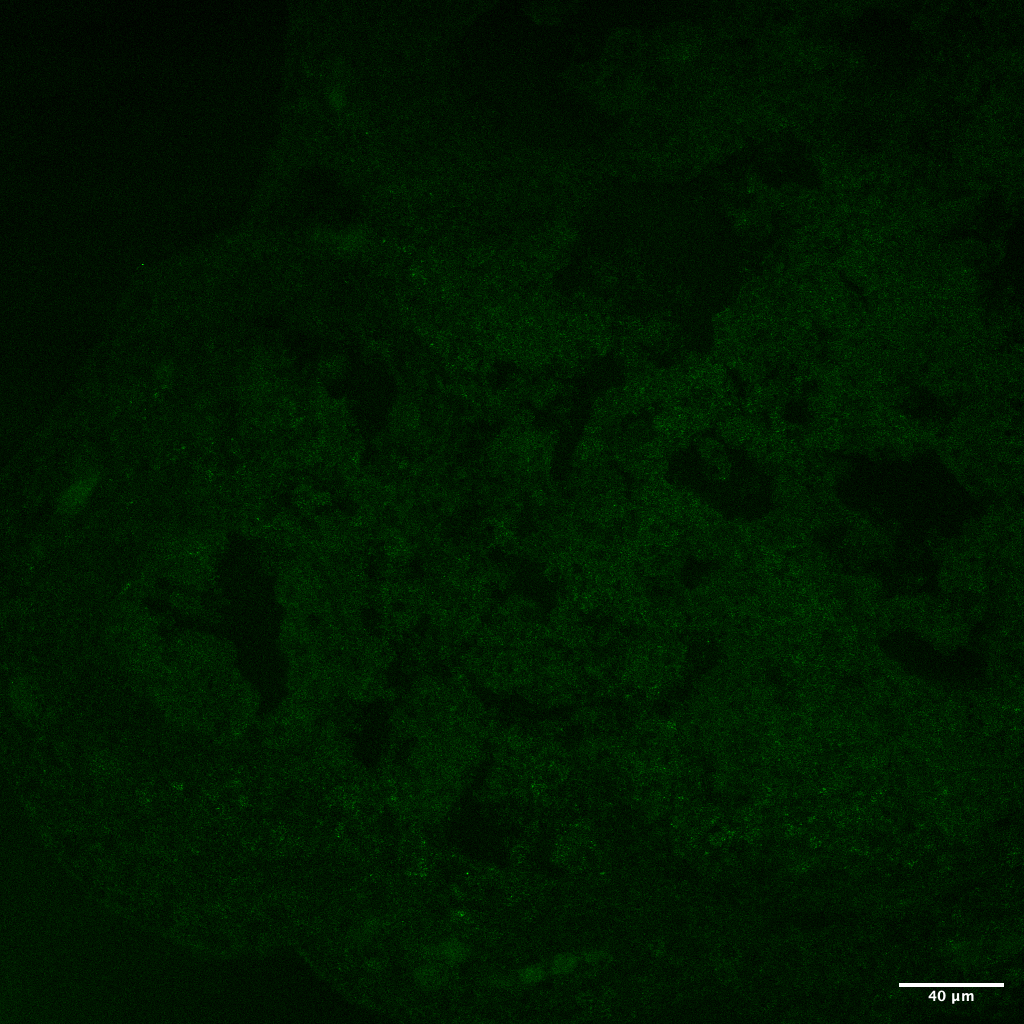

Supplement: Figure 5—source data 4. [file elife-83291-fig5-data4.zip › Figure5_sourcedata_timecourse_mouse/mouse_day14_DAZL.png]

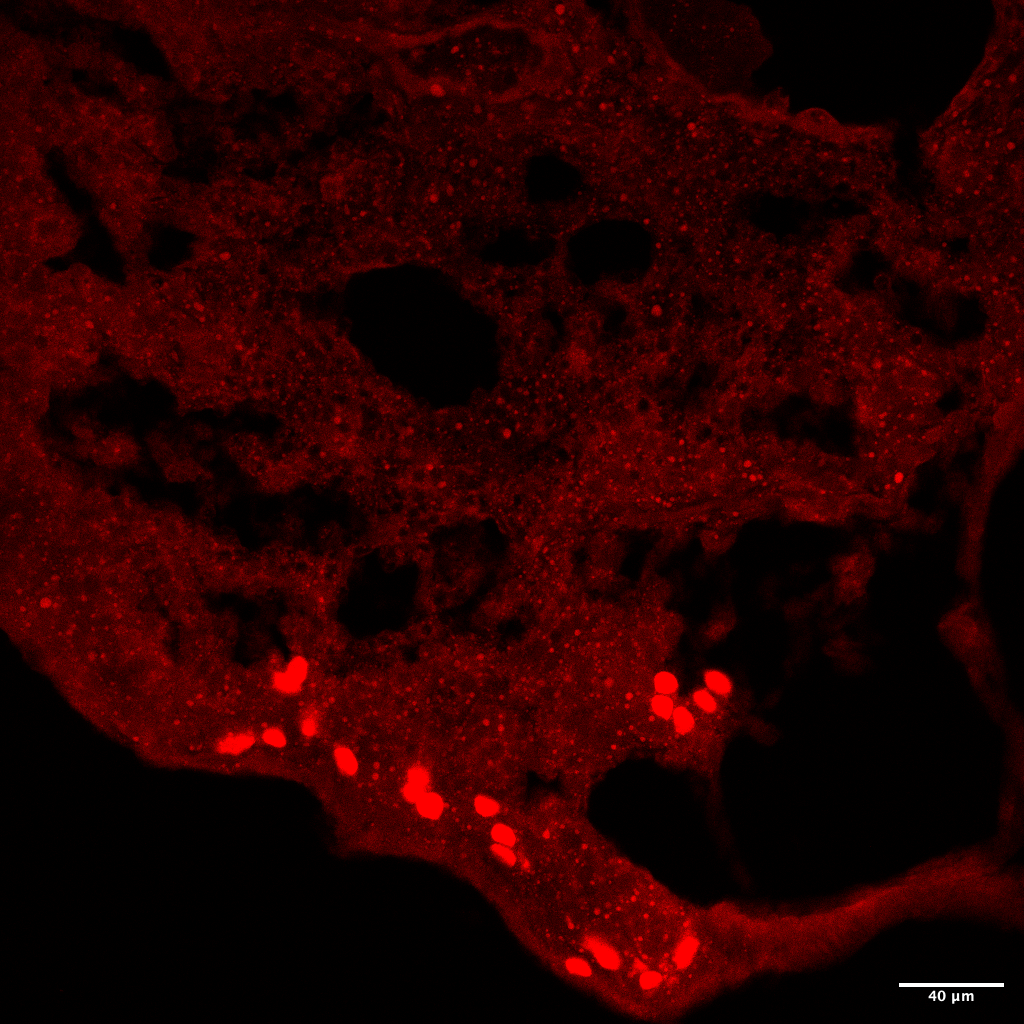

Supplement: Figure 5—source data 4. [file elife-83291-fig5-data4.zip › Figure5_sourcedata_timecourse_mouse/mouse_day32_OCT4.png]

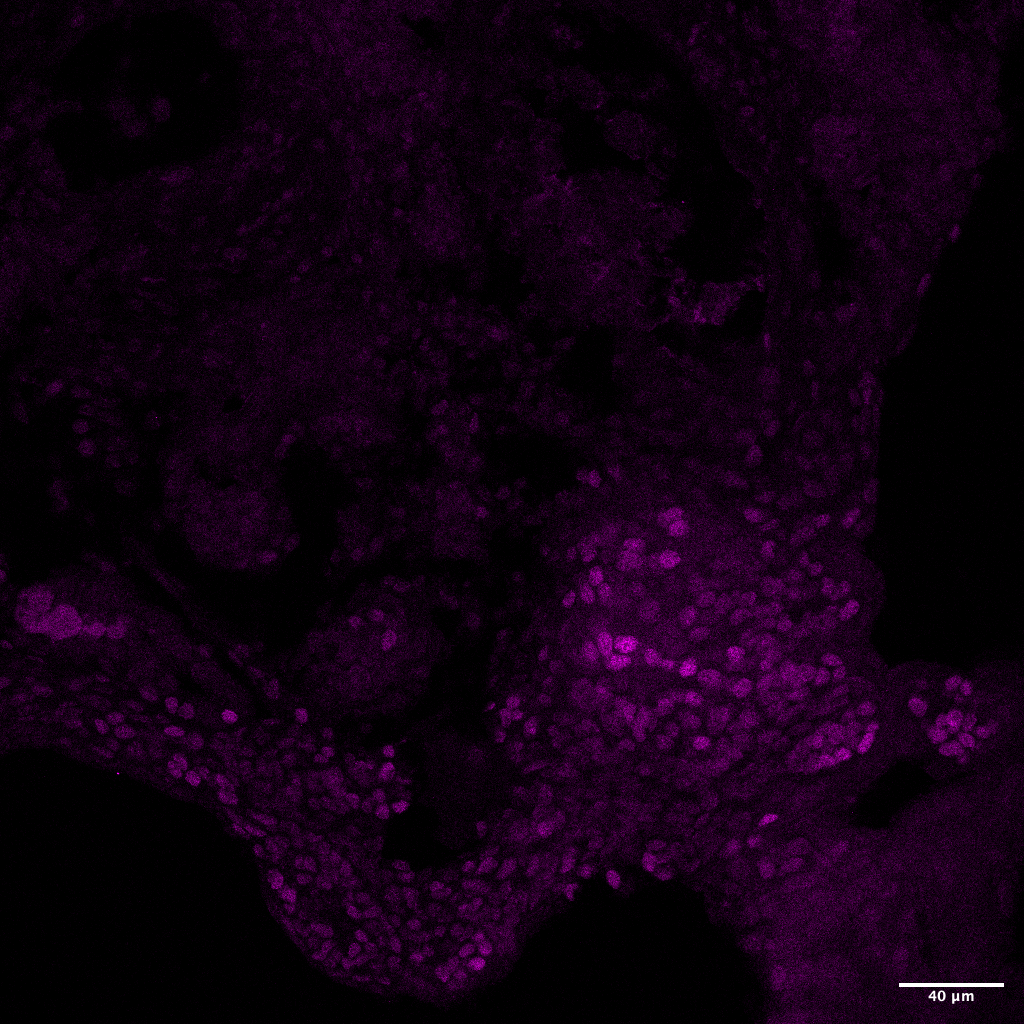

Supplement: Figure 5—source data 4. [file elife-83291-fig5-data4.zip › Figure5_sourcedata_timecourse_mouse/mouse_day38_FOXL2.png]

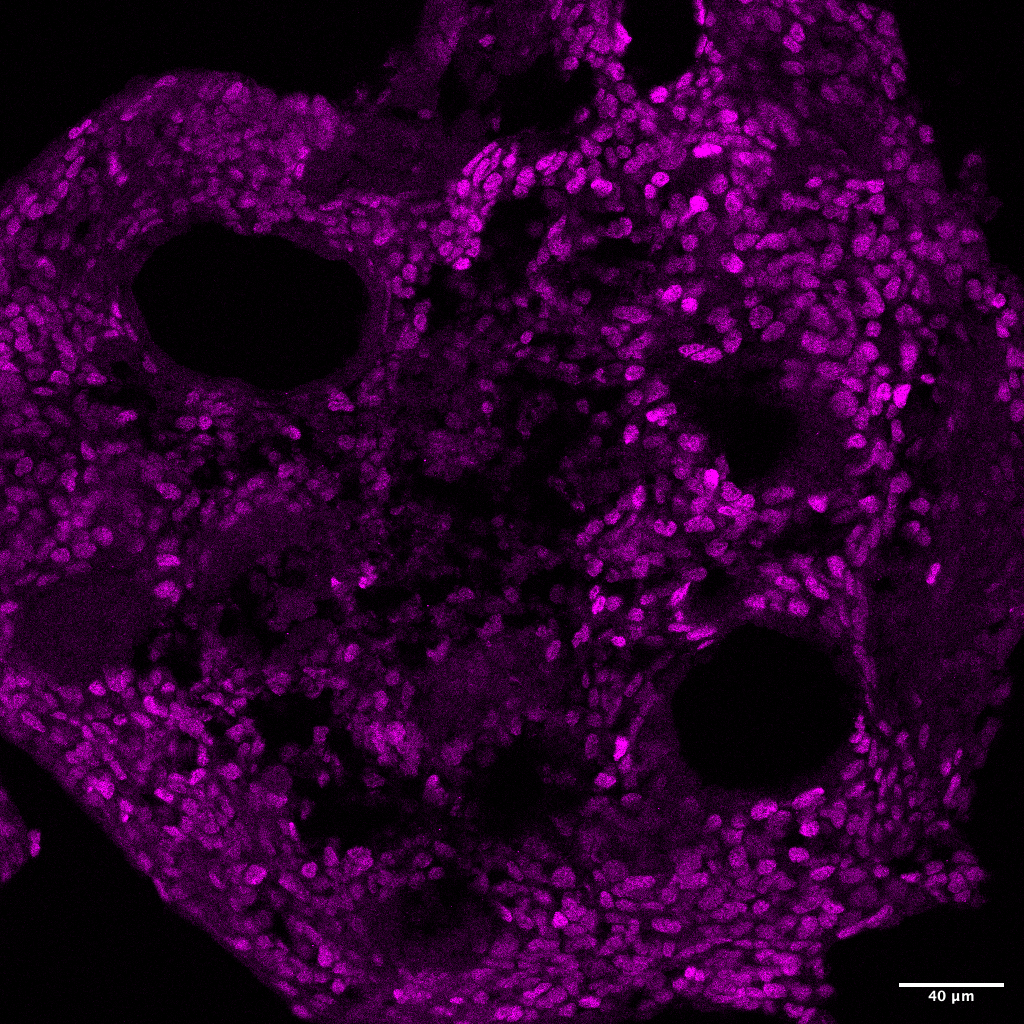

Supplement: Figure 5—source data 4. [file elife-83291-fig5-data4.zip › Figure5_sourcedata_timecourse_mouse/mouse_day8_FOXL2.png]

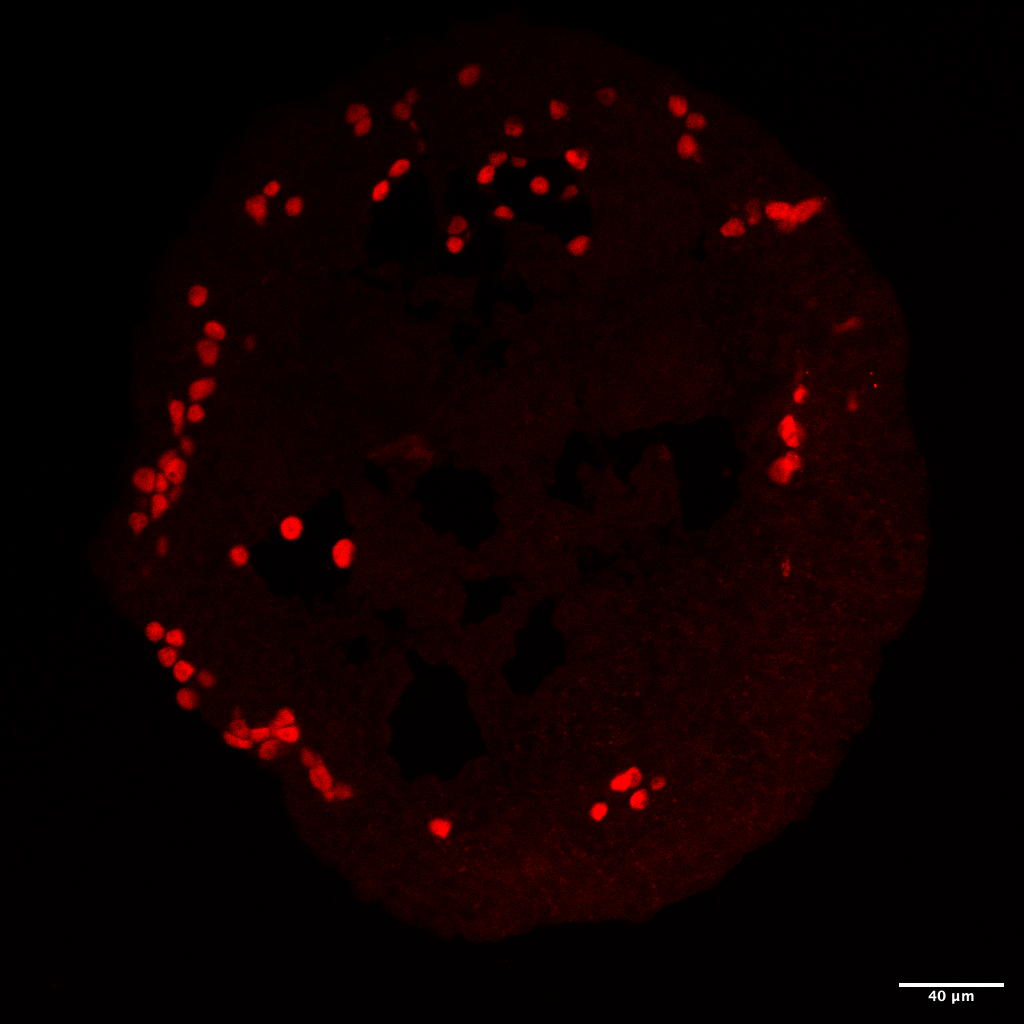

Supplement: Figure 5—source data 4. [file elife-83291-fig5-data4.zip › Figure5_sourcedata_timecourse_mouse/mouse_day4_OCT4.png]

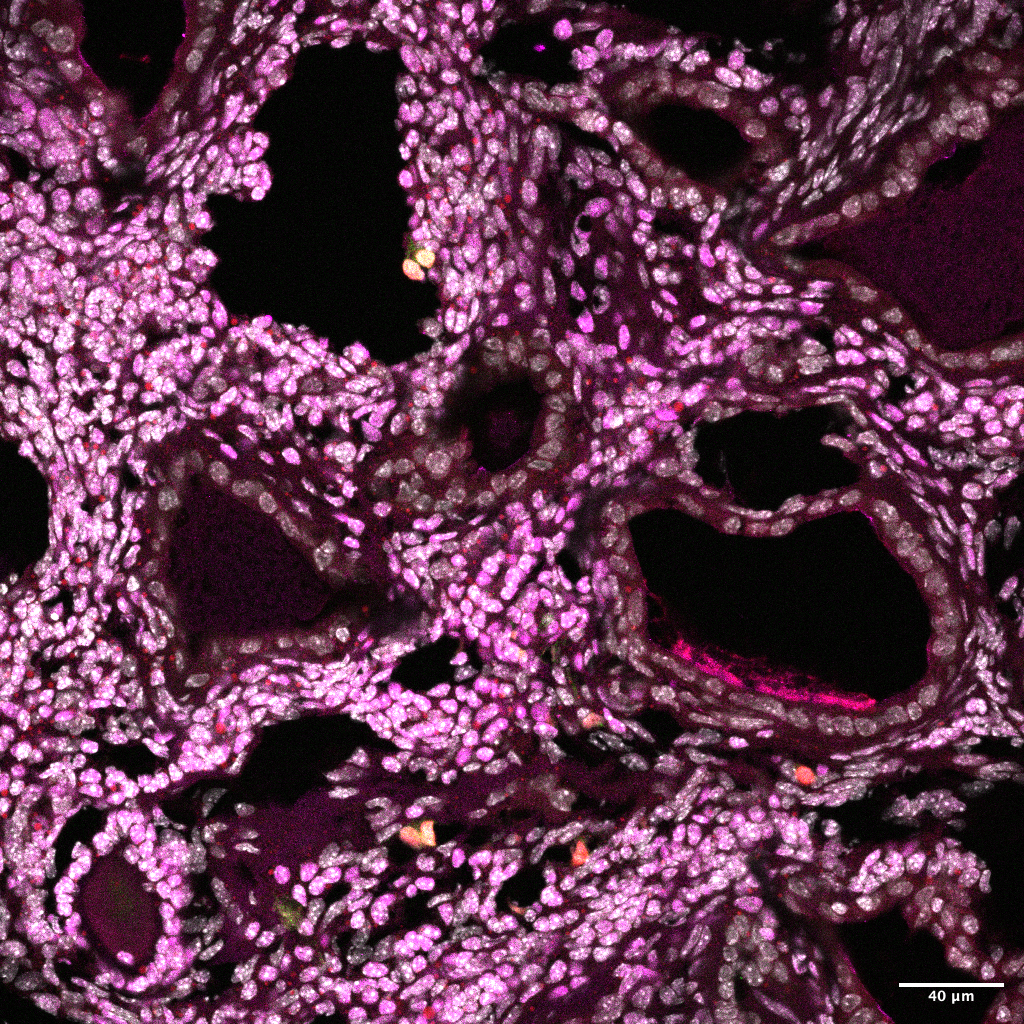

Supplement: Figure 5—source data 4. [file elife-83291-fig5-data4.zip › Figure5_sourcedata_timecourse_mouse/mouse_day26_composite.png]

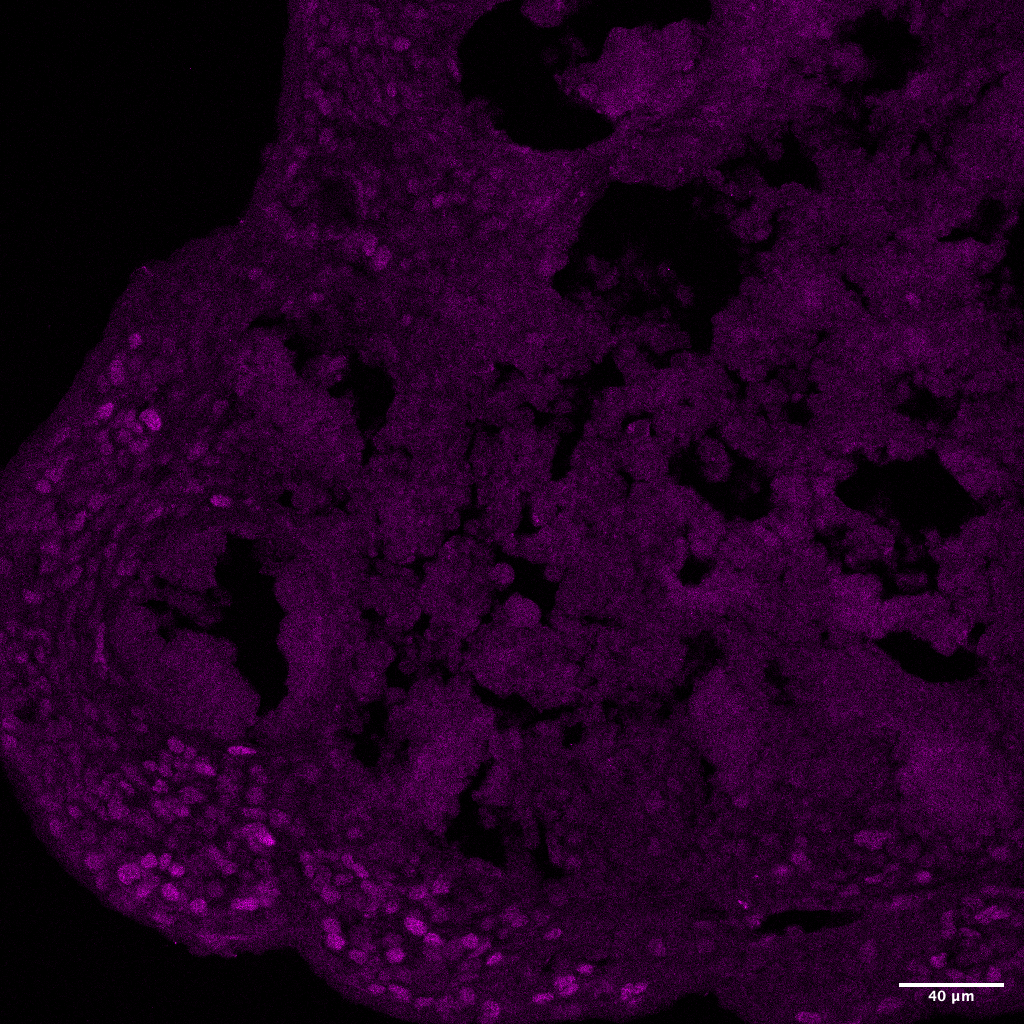

Supplement: Figure 5—source data 4. [file elife-83291-fig5-data4.zip › Figure5_sourcedata_timecourse_mouse/mouse_day14_FOXL2.png]

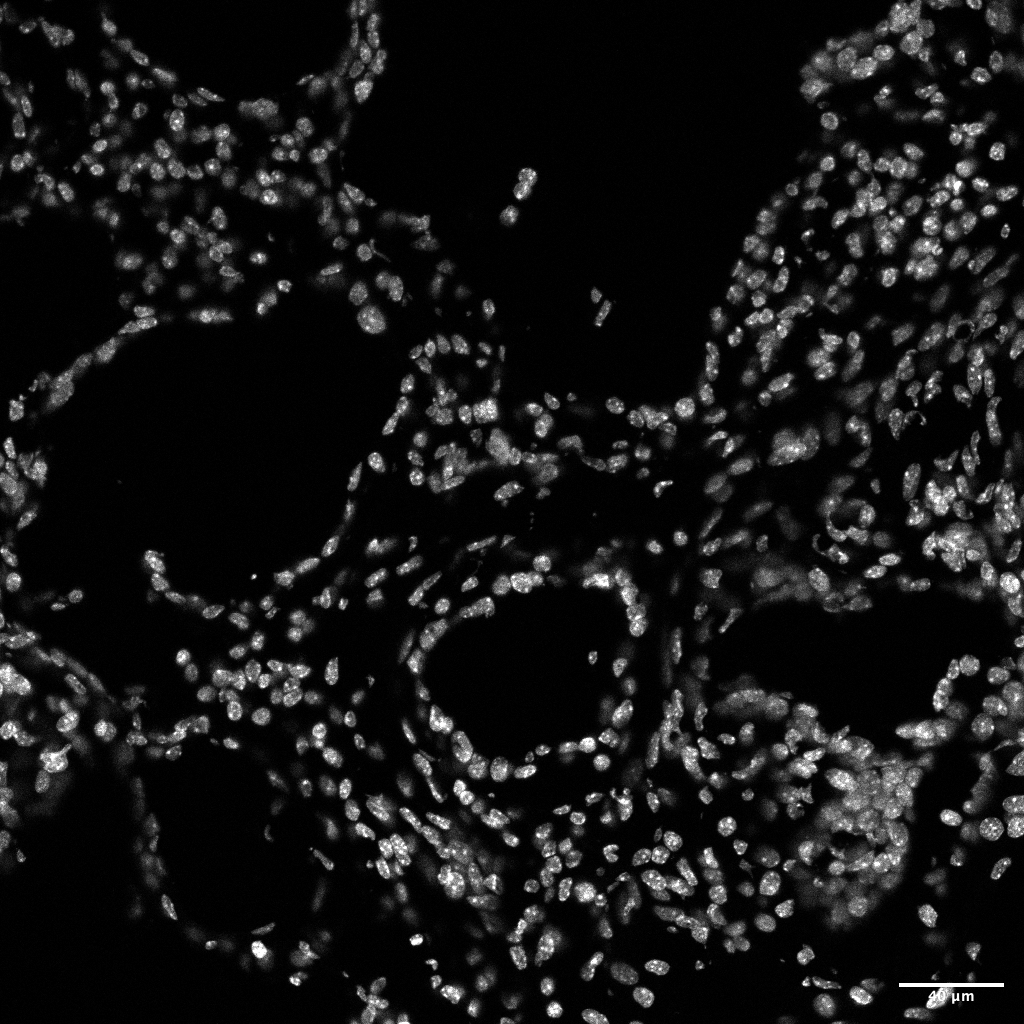

Supplement: Figure 5—source data 4. [file elife-83291-fig5-data4.zip › Figure5_sourcedata_timecourse_mouse/mouse_day46_DAPI.png]

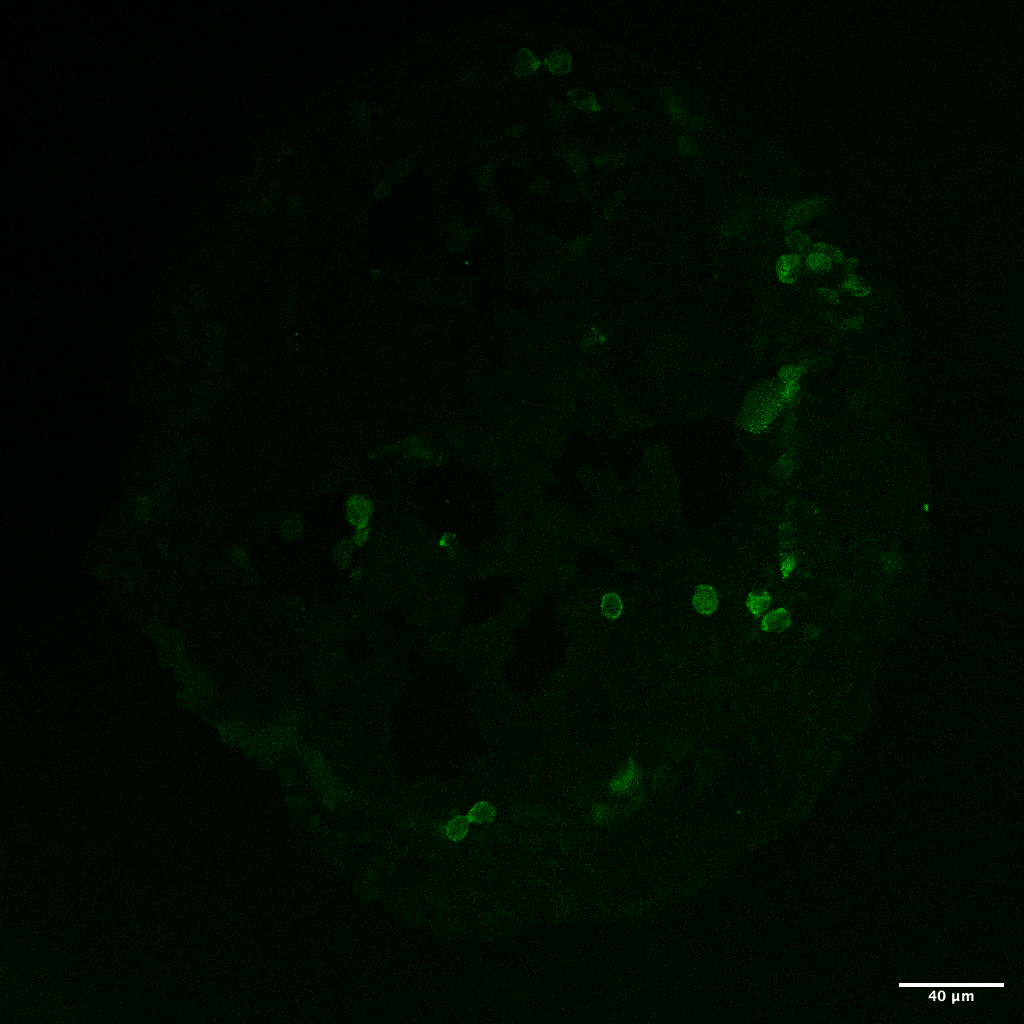

Supplement: Figure 5—source data 4. [file elife-83291-fig5-data4.zip › Figure5_sourcedata_timecourse_mouse/mouse_day4_DAZL.png]

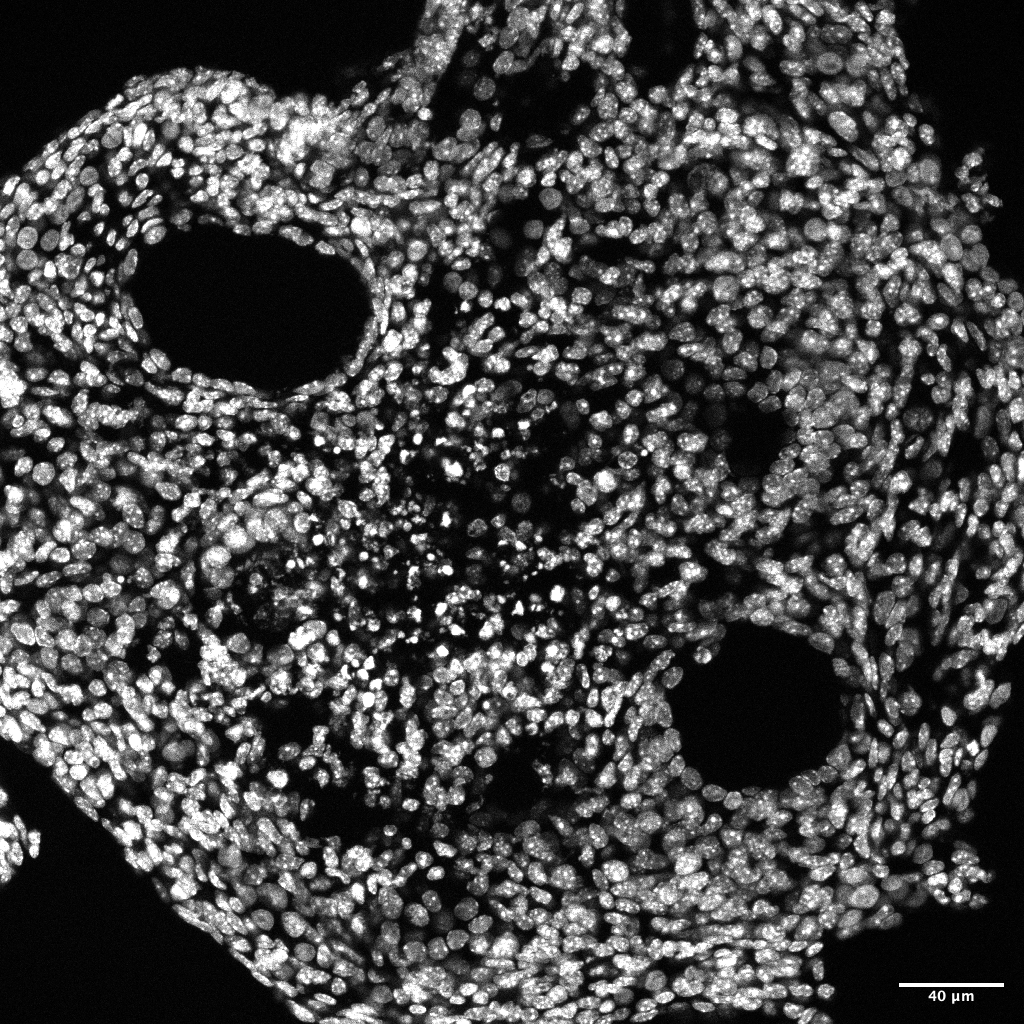

Supplement: Figure 5—source data 4. [file elife-83291-fig5-data4.zip › Figure5_sourcedata_timecourse_mouse/mouse_day8_DAPI.png]

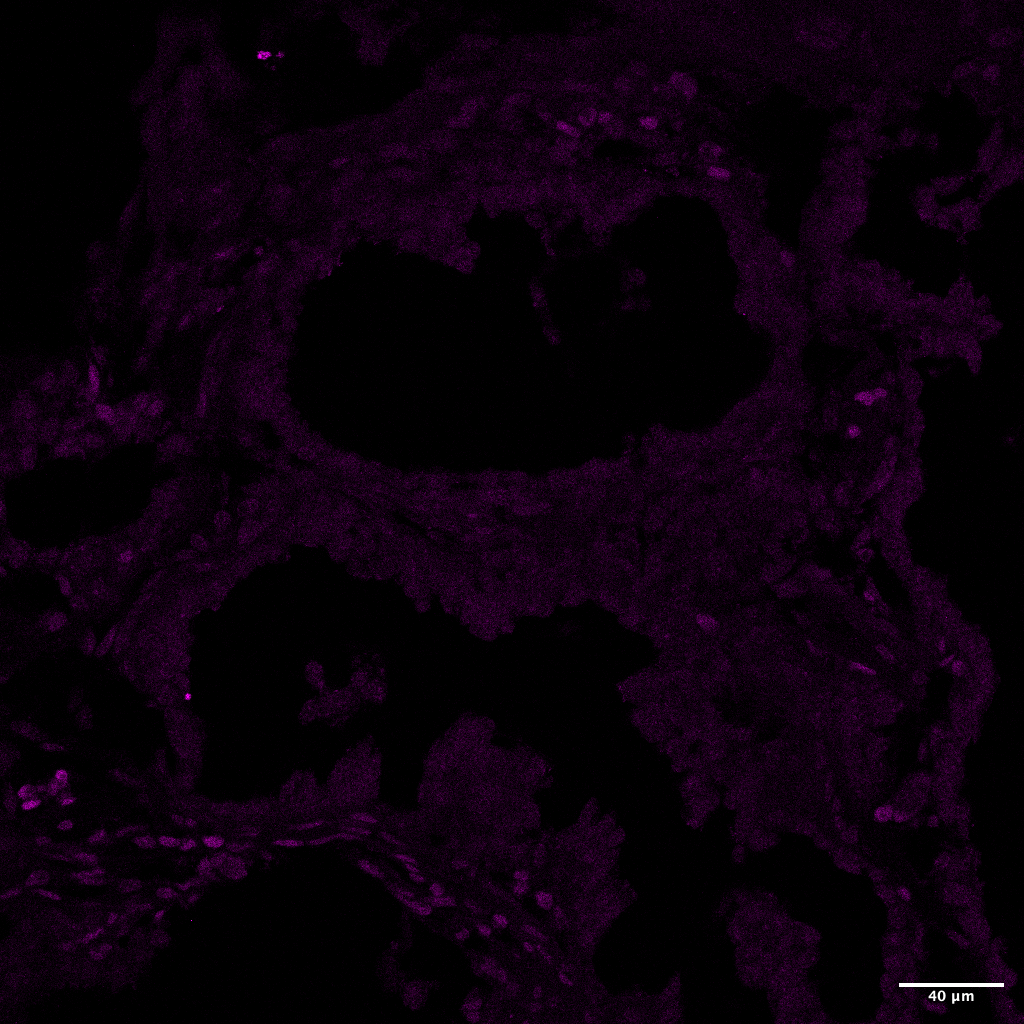

Supplement: Figure 5—source data 4. [file elife-83291-fig5-data4.zip › Figure5_sourcedata_timecourse_mouse/Day70_FOXL2.png]

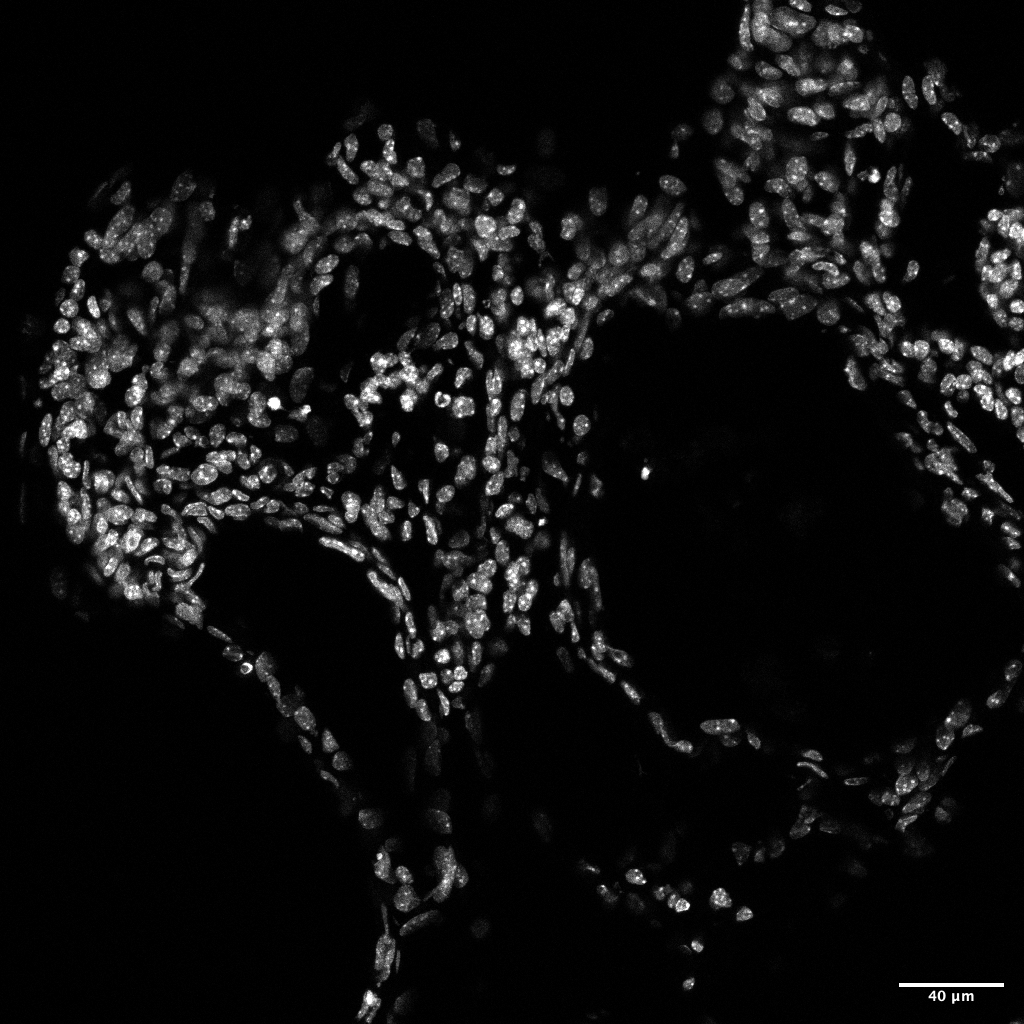

Supplement: Figure 5—source data 4. [file elife-83291-fig5-data4.zip › Figure5_sourcedata_timecourse_mouse/mouse_day54_DAPI.png]

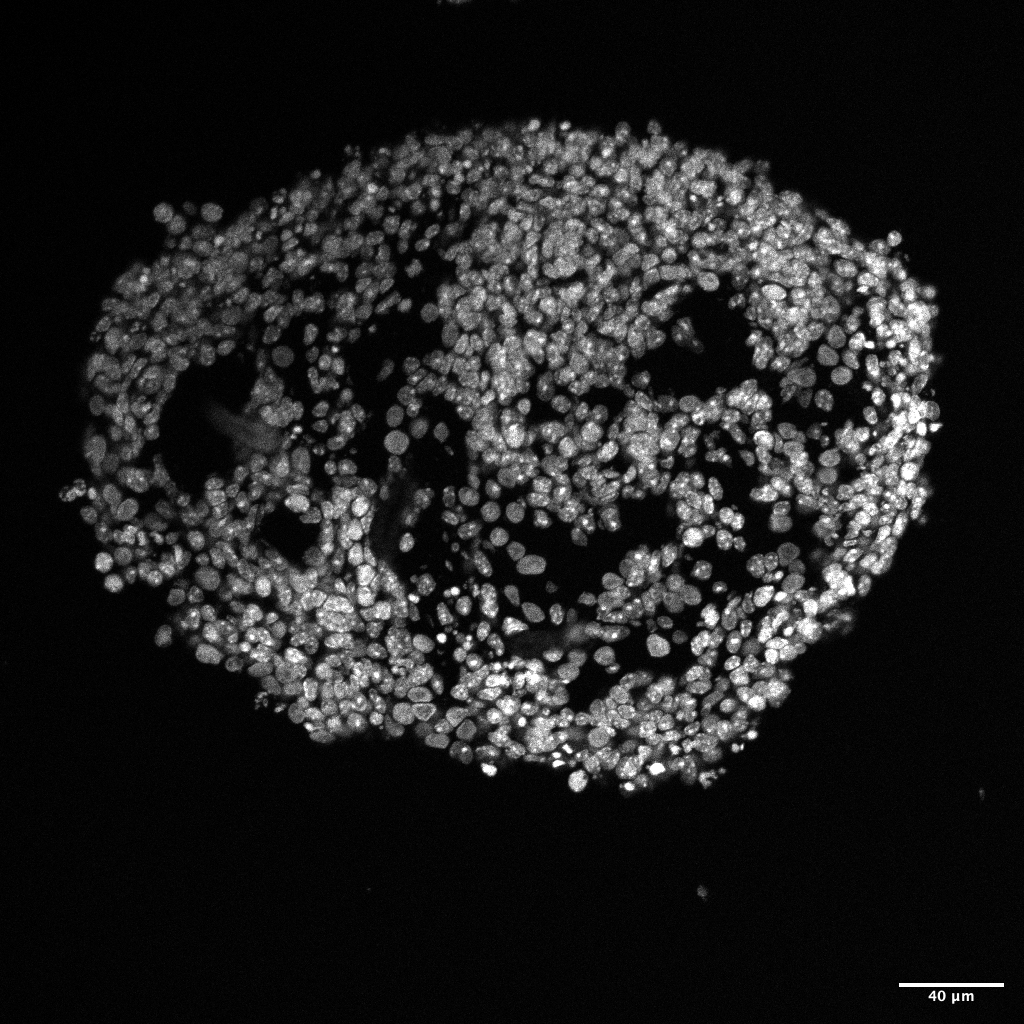

Supplement: Figure 5—source data 4. [file elife-83291-fig5-data4.zip › Figure5_sourcedata_timecourse_mouse/mouse_day2_DAPI.png]

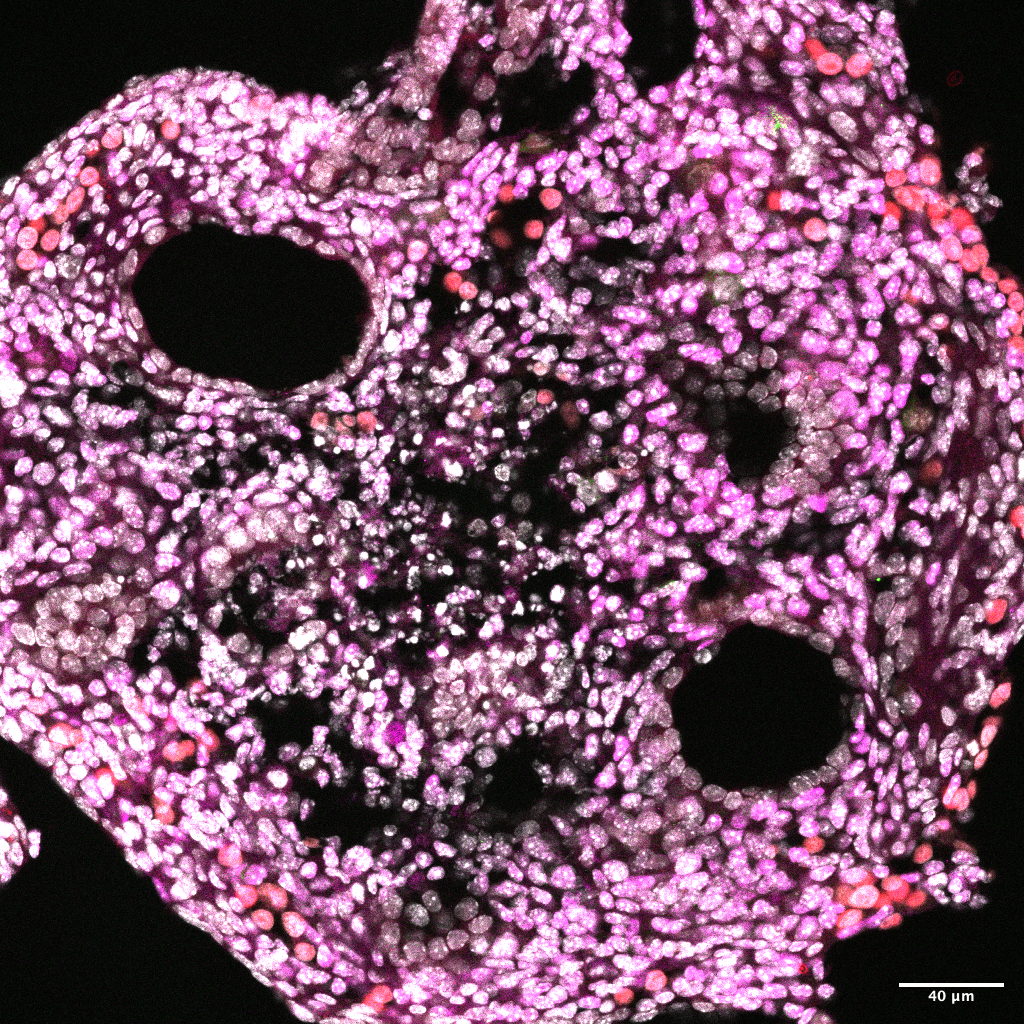

Supplement: Figure 5—source data 4. [file elife-83291-fig5-data4.zip › Figure5_sourcedata_timecourse_mouse/mouse_day8_composite.png]

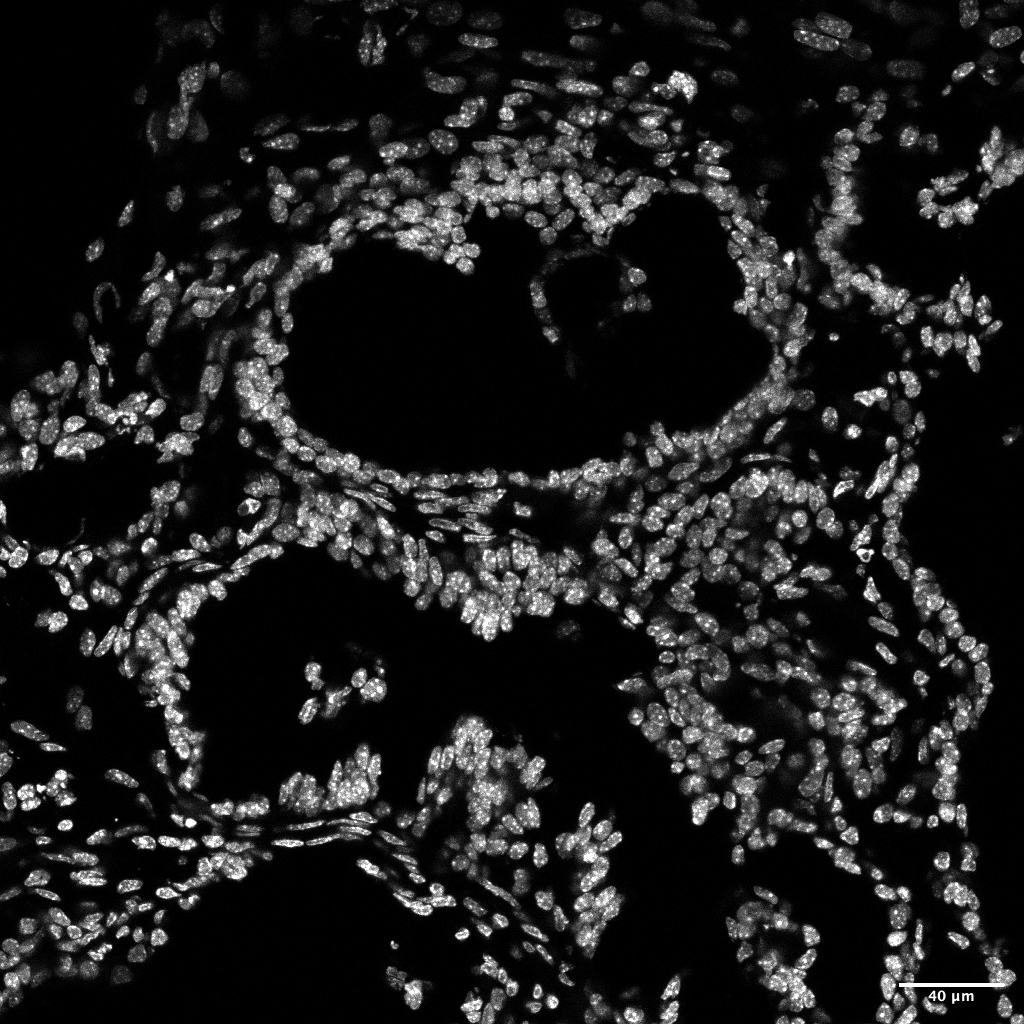

Supplement: Figure 5—source data 4. [file elife-83291-fig5-data4.zip › Figure5_sourcedata_timecourse_mouse/Day70_DAPI.png]

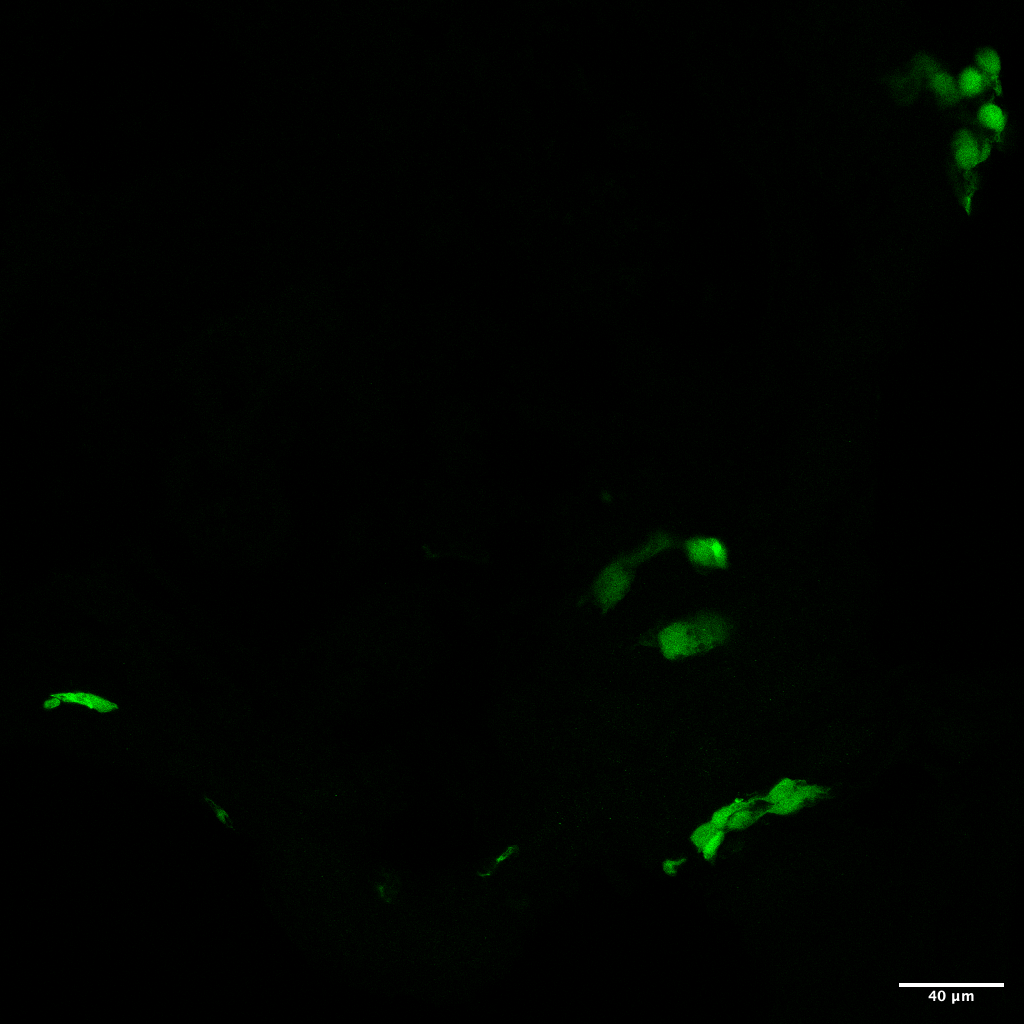

Supplement: Figure 5—source data 4. [file elife-83291-fig5-data4.zip › Figure5_sourcedata_timecourse_mouse/mouse_day38_DAZL.png]

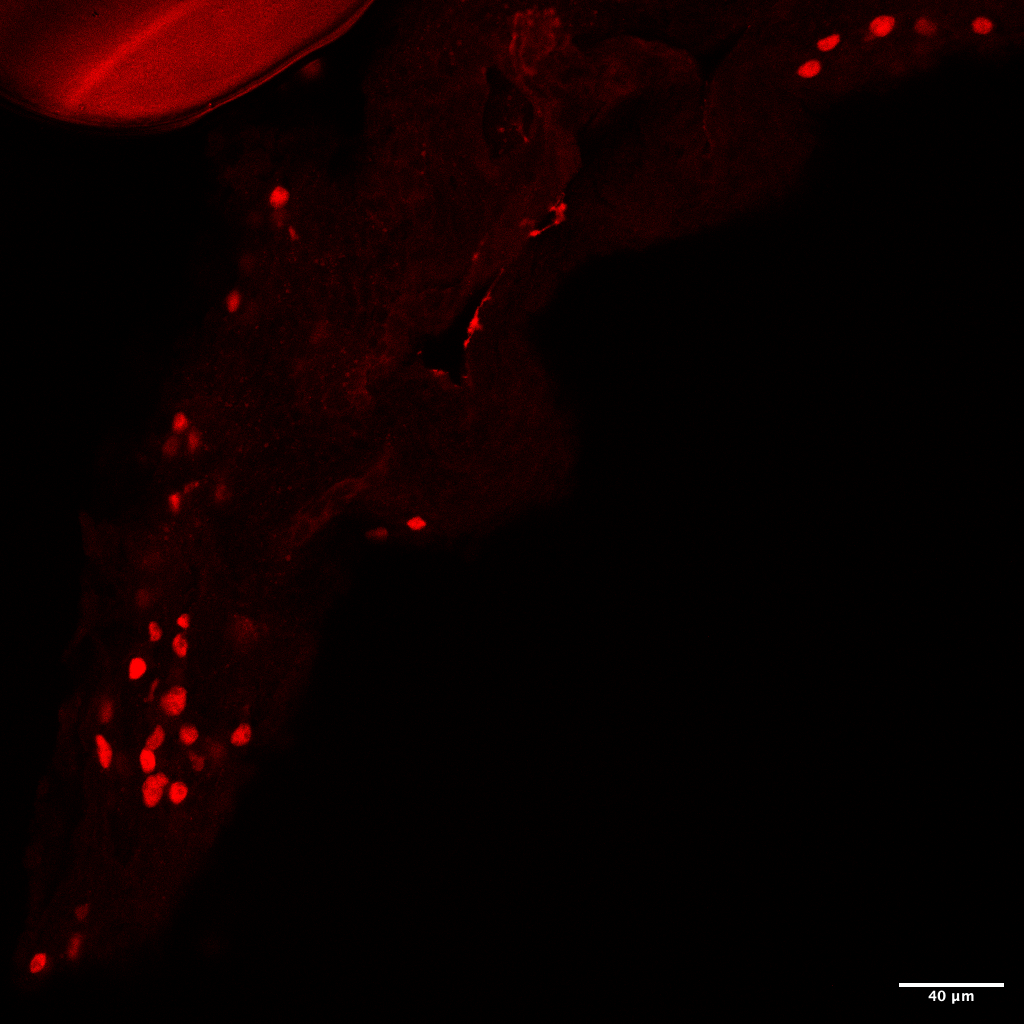

Supplement: Figure 5—source data 4. [file elife-83291-fig5-data4.zip › Figure5_sourcedata_timecourse_mouse/mouse_day20_OCT4.png]

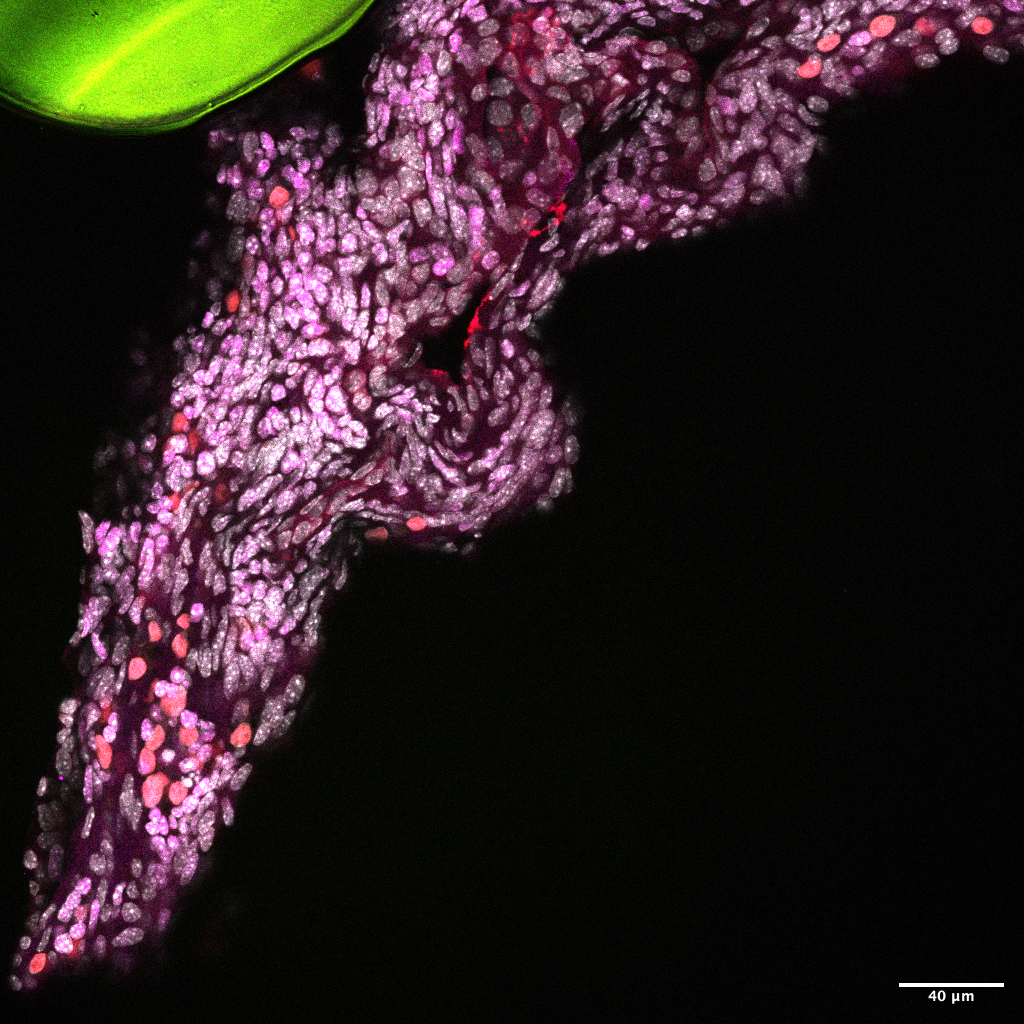

Supplement: Figure 5—source data 4. [file elife-83291-fig5-data4.zip › Figure5_sourcedata_timecourse_mouse/mouse_day20_composite.png]

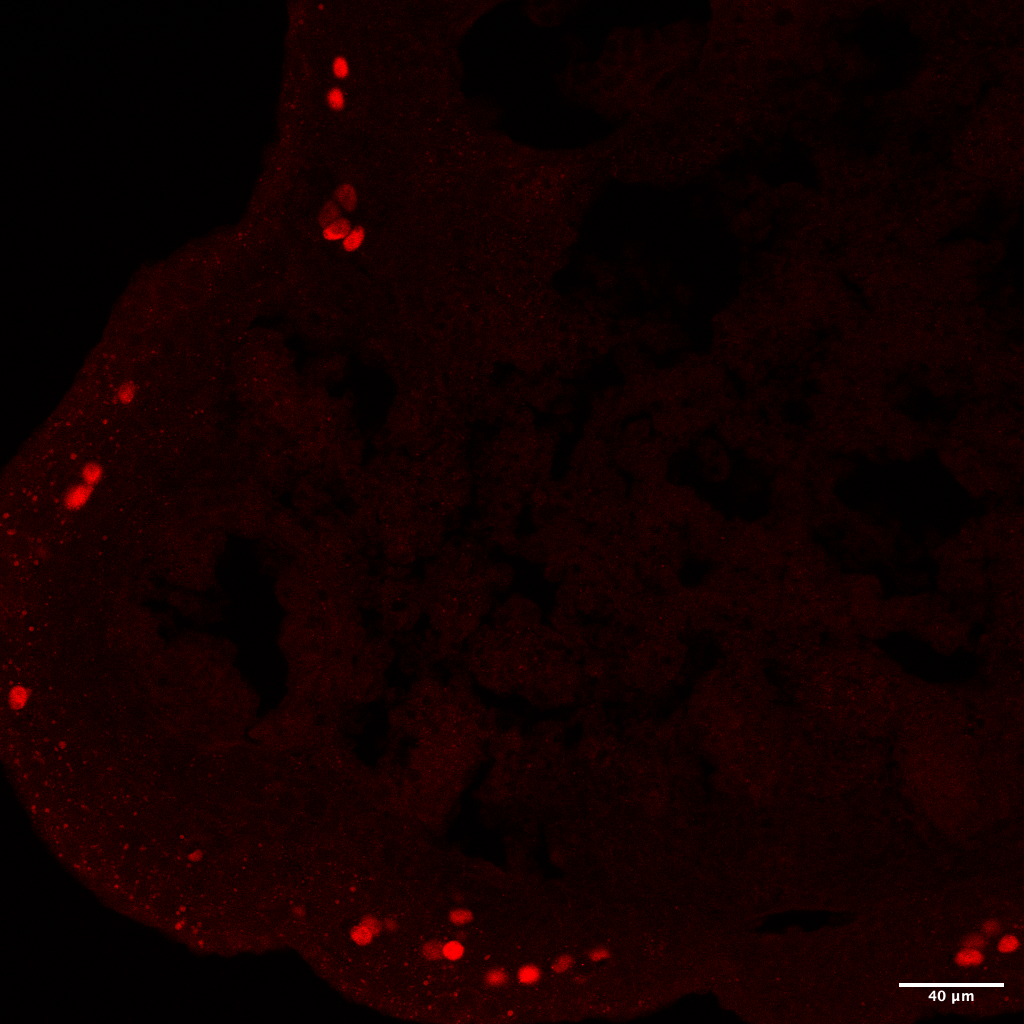

Supplement: Figure 5—source data 4. [file elife-83291-fig5-data4.zip › Figure5_sourcedata_timecourse_mouse/mouse_day14_OCT4.png]

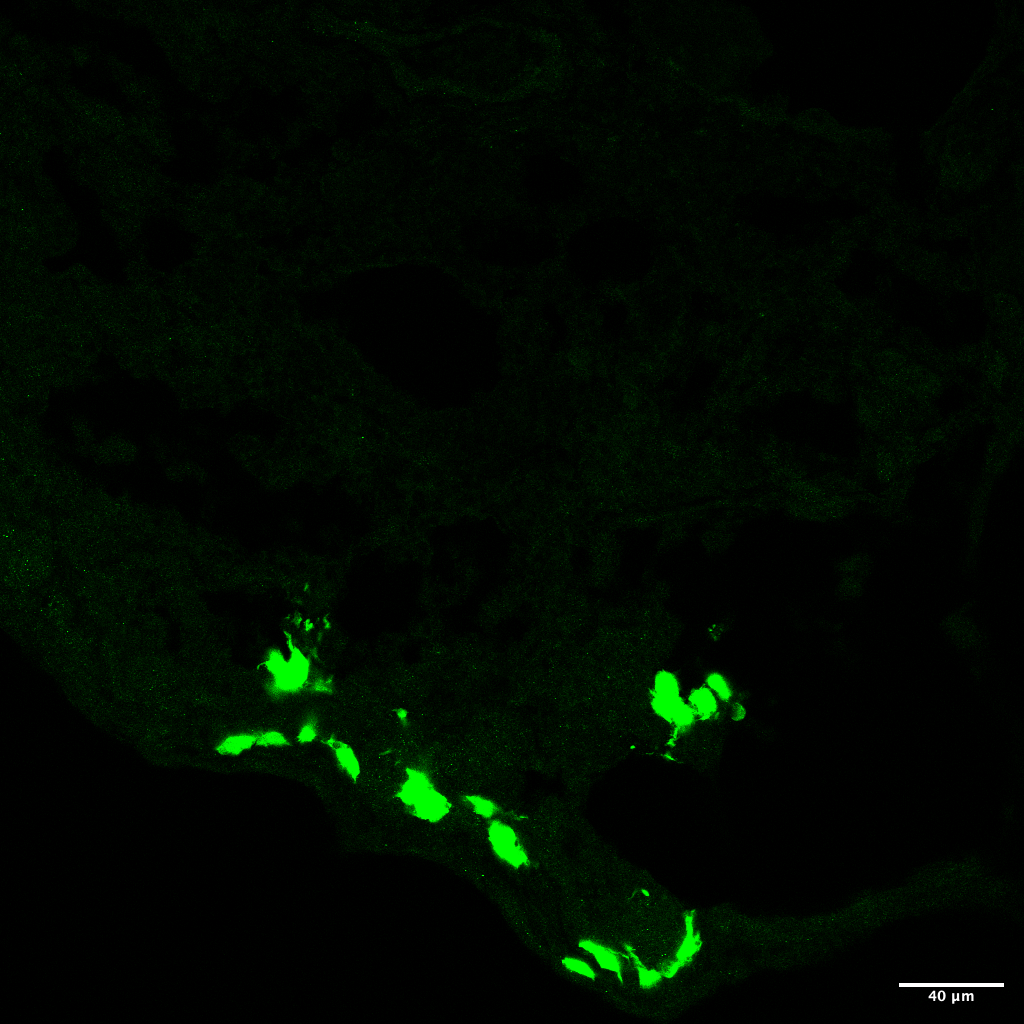

Supplement: Figure 5—source data 4. [file elife-83291-fig5-data4.zip › Figure5_sourcedata_timecourse_mouse/mouse_day32_DAZL.png]

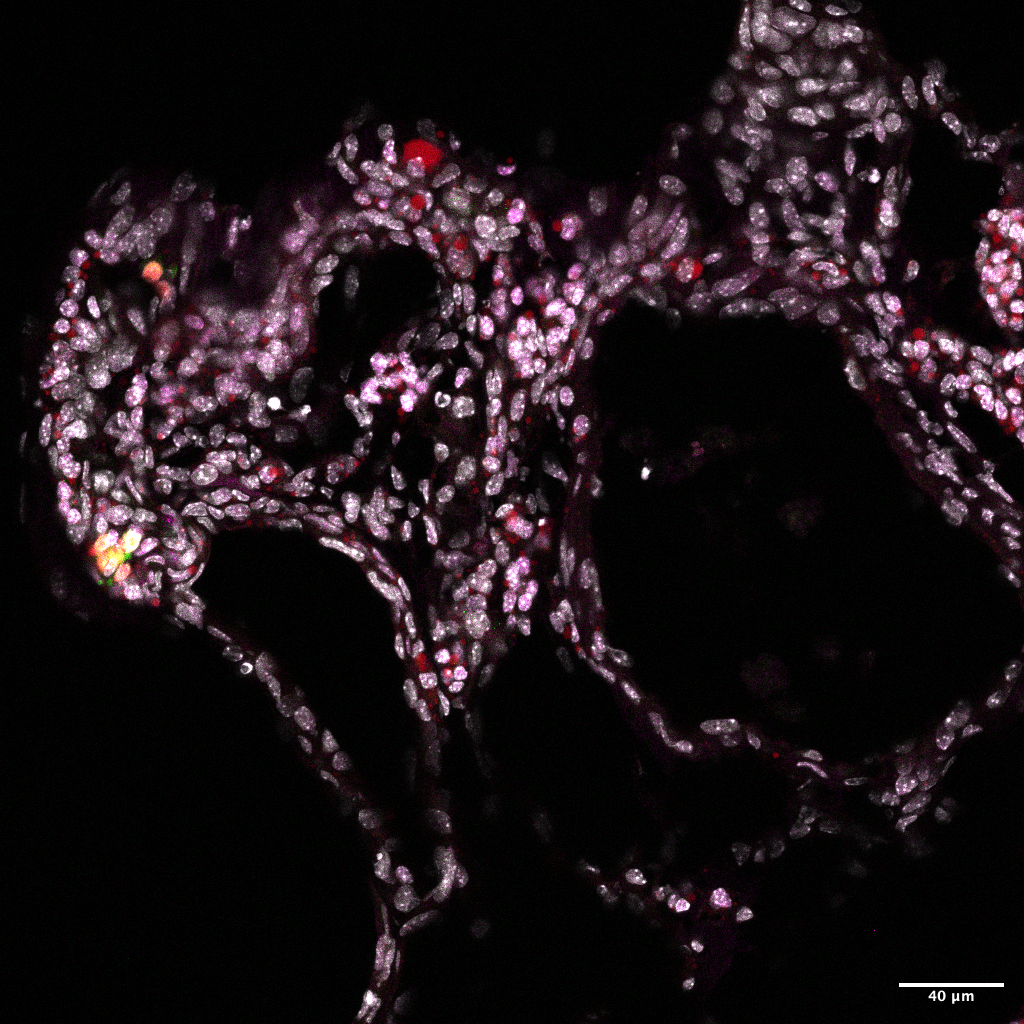

Supplement: Figure 5—source data 4. [file elife-83291-fig5-data4.zip › Figure5_sourcedata_timecourse_mouse/mouse_day54_composite.png]

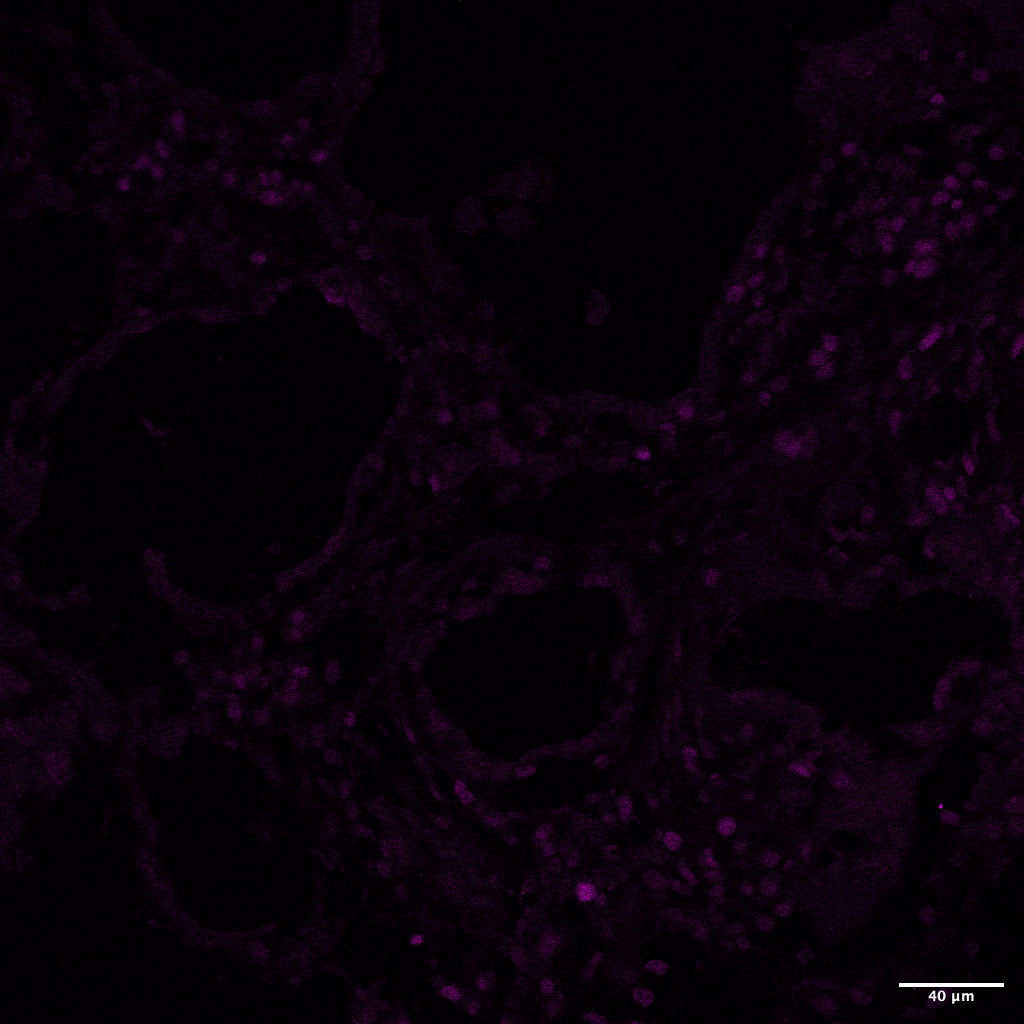

Supplement: Figure 5—source data 4. [file elife-83291-fig5-data4.zip › Figure5_sourcedata_timecourse_mouse/mouse_day46_FOXL2.png]

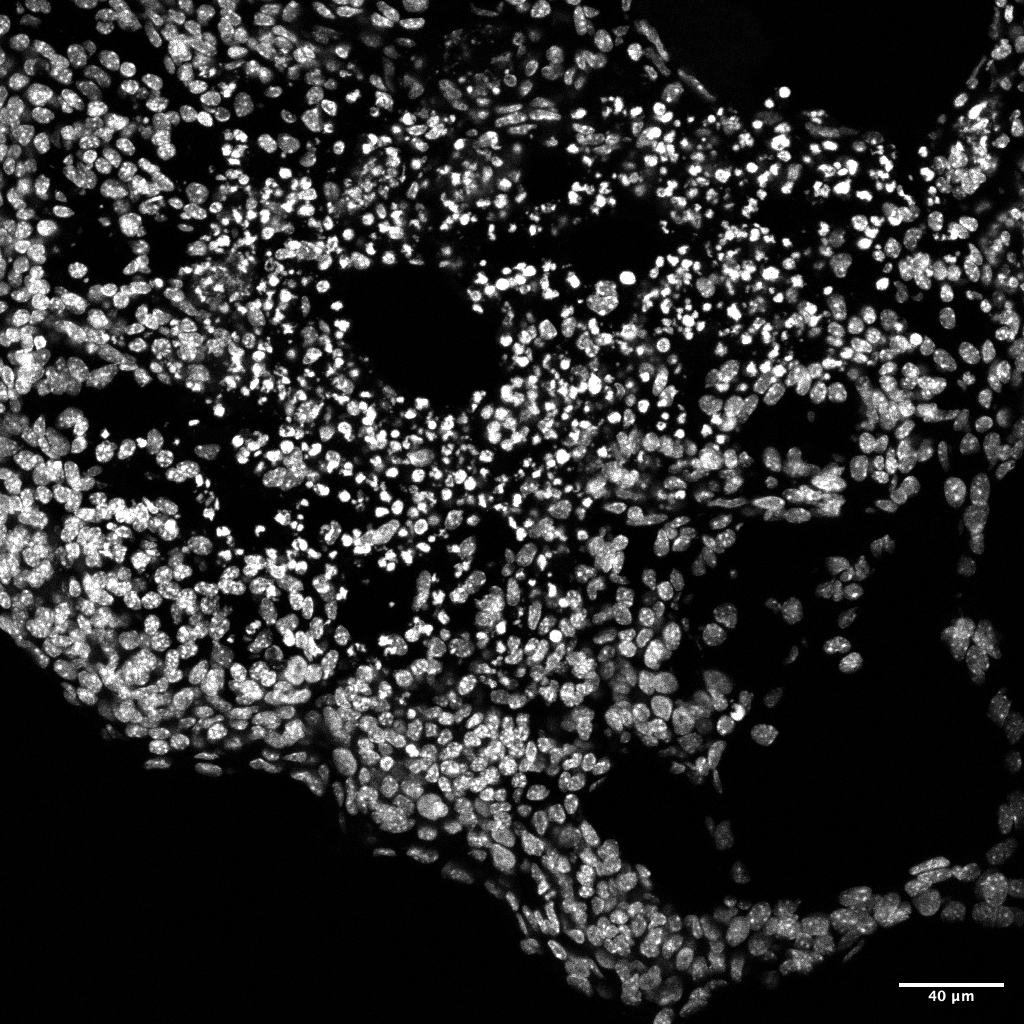

Supplement: Figure 5—source data 4. [file elife-83291-fig5-data4.zip › Figure5_sourcedata_timecourse_mouse/mouse_day32_DAPI.png]

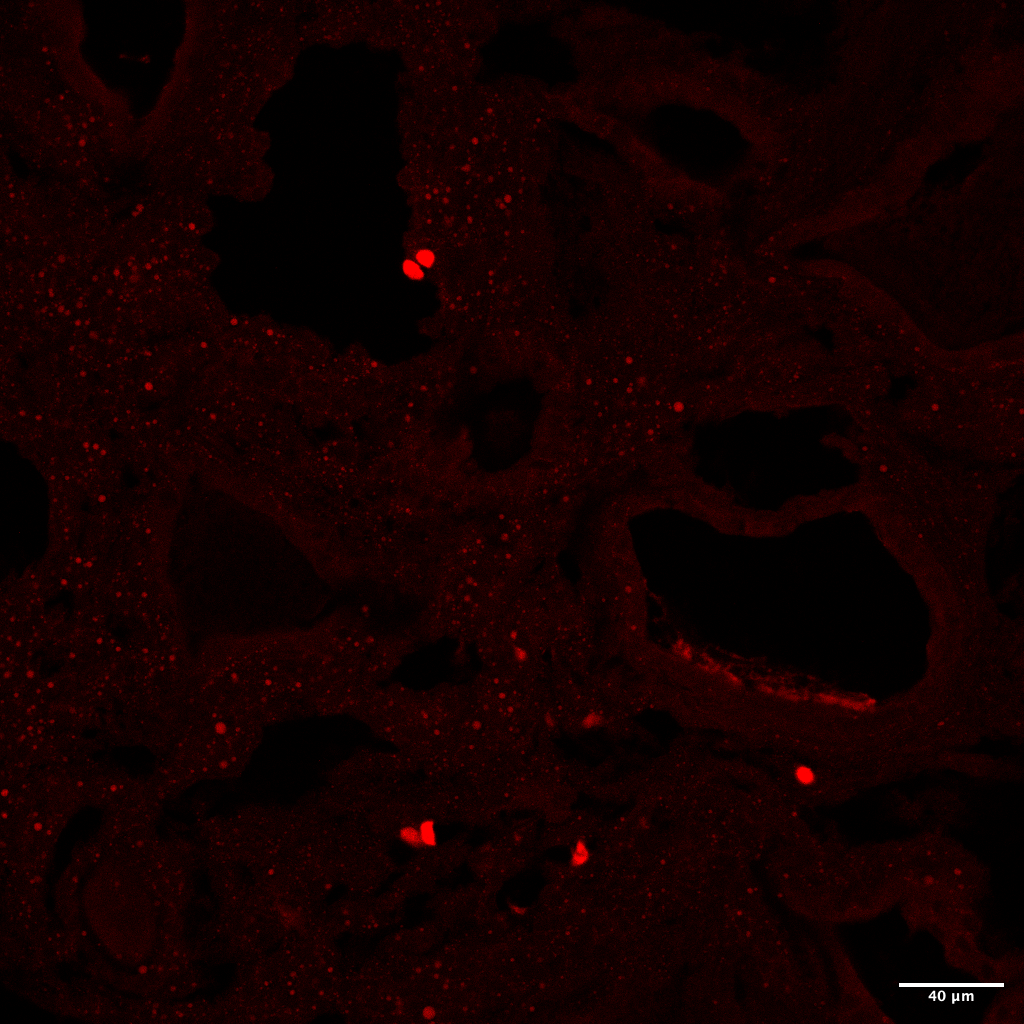

Supplement: Figure 5—source data 4. [file elife-83291-fig5-data4.zip › Figure5_sourcedata_timecourse_mouse/mouse_day26_OCT4.png]

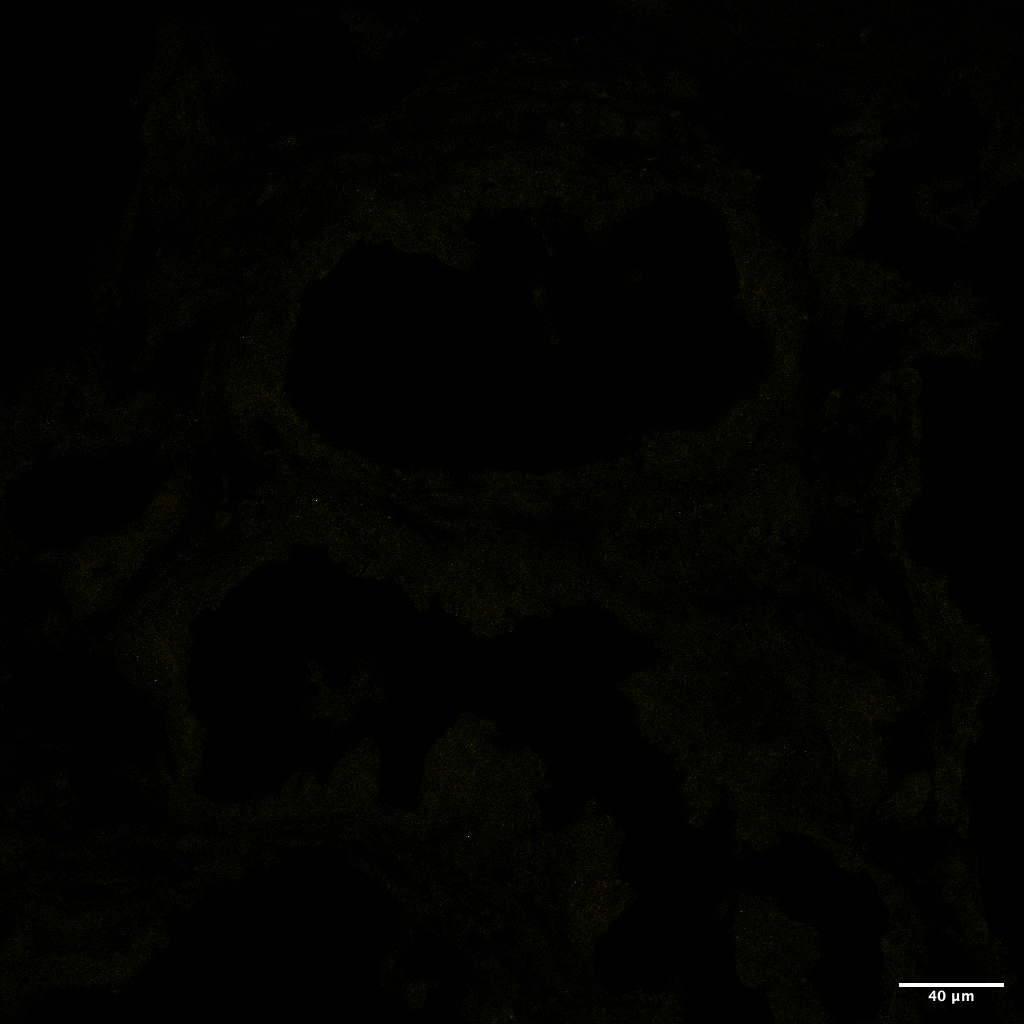

Supplement: Figure 5—source data 4. [file elife-83291-fig5-data4.zip › Figure5_sourcedata_timecourse_mouse/Day70_DAZL.png]

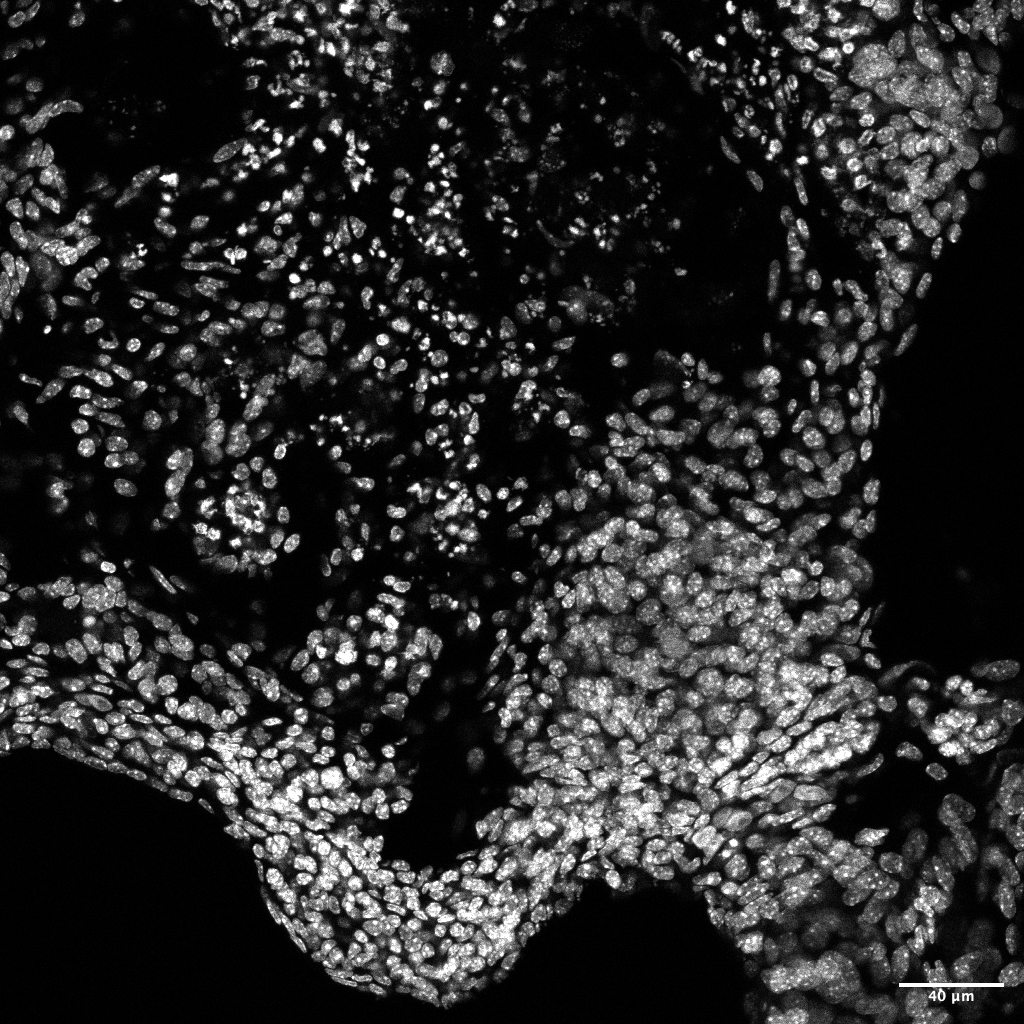

Supplement: Figure 5—source data 4. [file elife-83291-fig5-data4.zip › Figure5_sourcedata_timecourse_mouse/mouse_day38_DAPI.png]

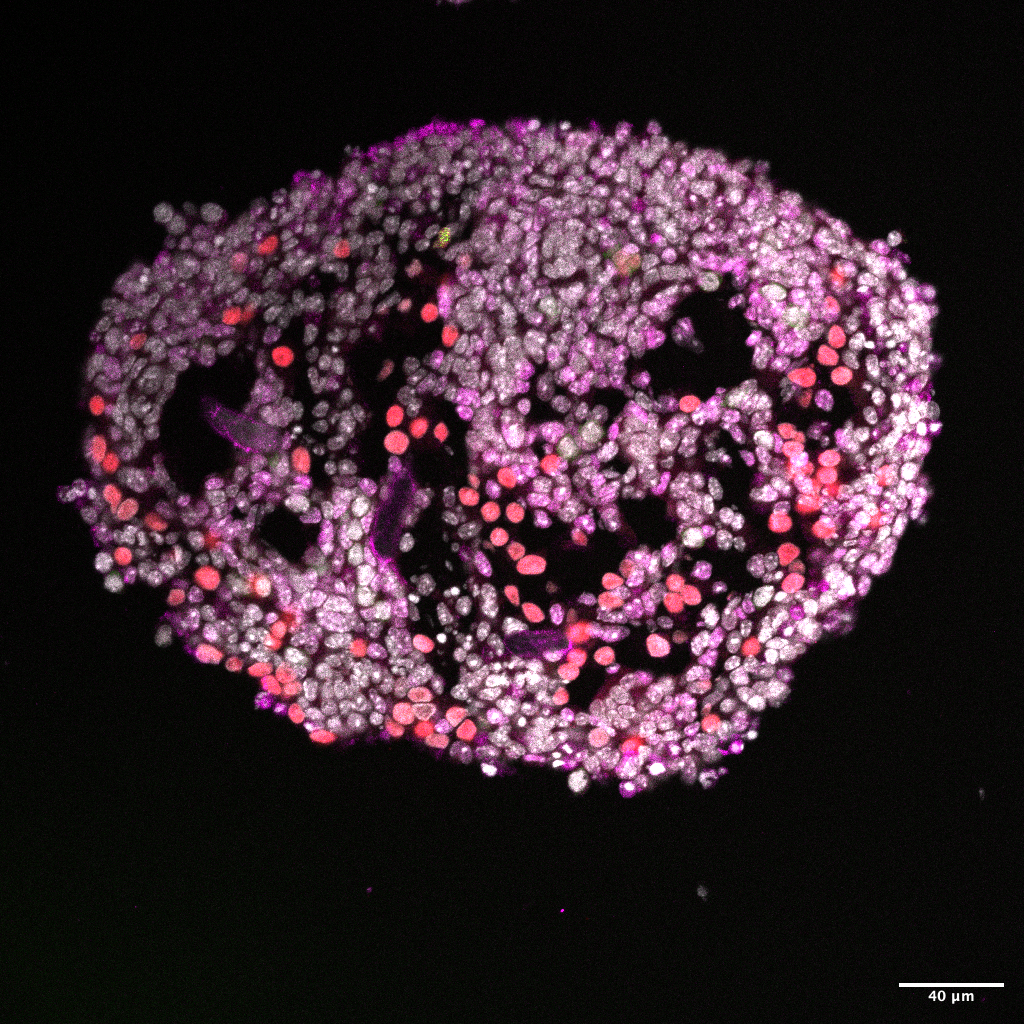

Supplement: Figure 5—source data 4. [file elife-83291-fig5-data4.zip › Figure5_sourcedata_timecourse_mouse/mouse_day2_composite.png]

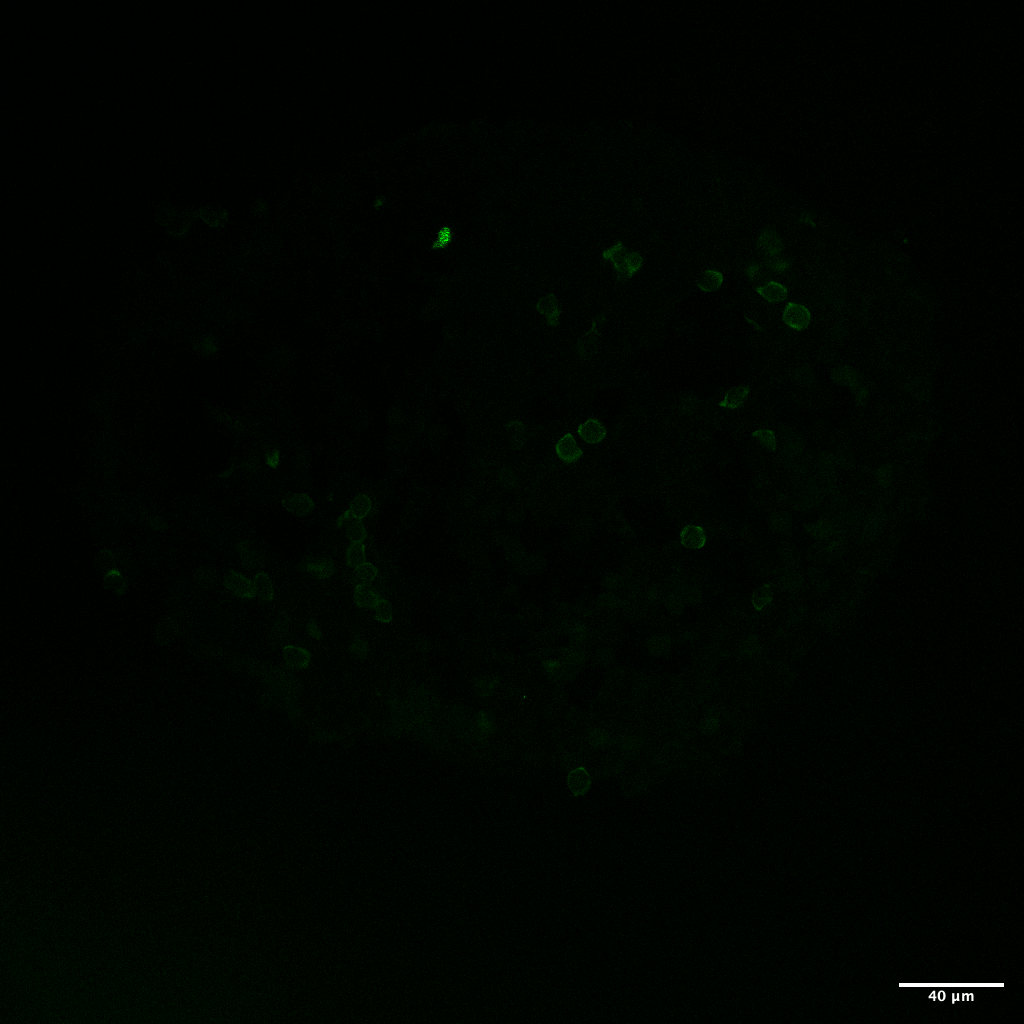

Supplement: Figure 5—source data 4. [file elife-83291-fig5-data4.zip › Figure5_sourcedata_timecourse_mouse/mouse_day2_DAZL.png]

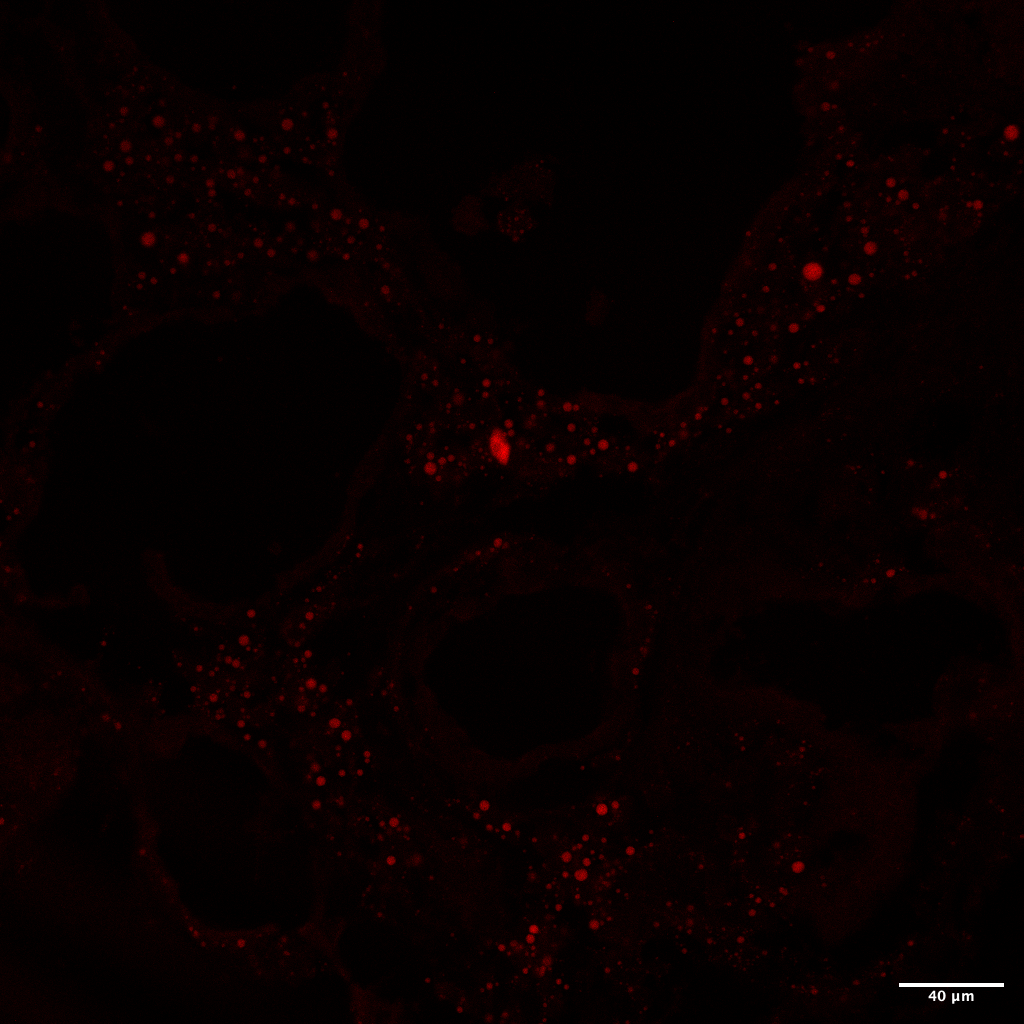

Supplement: Figure 5—source data 4. [file elife-83291-fig5-data4.zip › Figure5_sourcedata_timecourse_mouse/mouse_day46_OCT4.png]

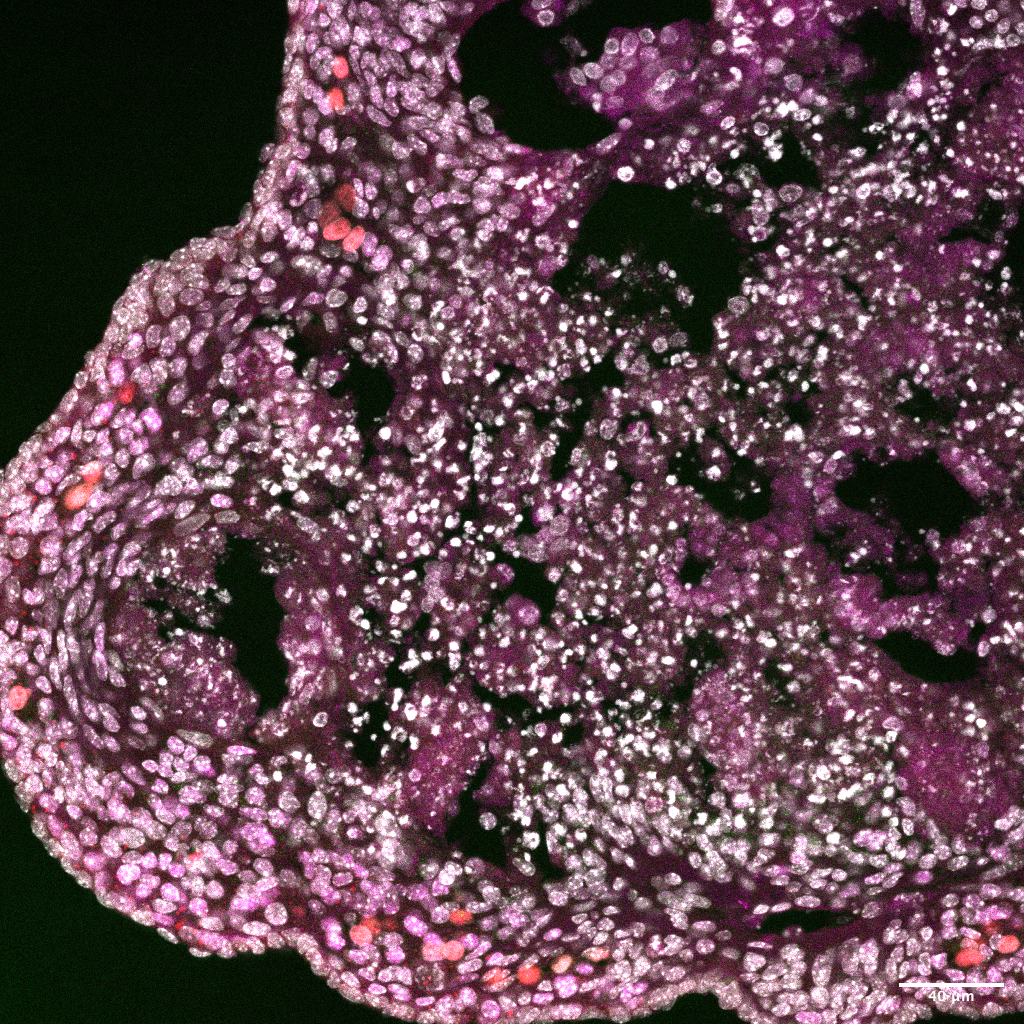

Supplement: Figure 5—source data 4. [file elife-83291-fig5-data4.zip › Figure5_sourcedata_timecourse_mouse/mouse_day14_composite.png]

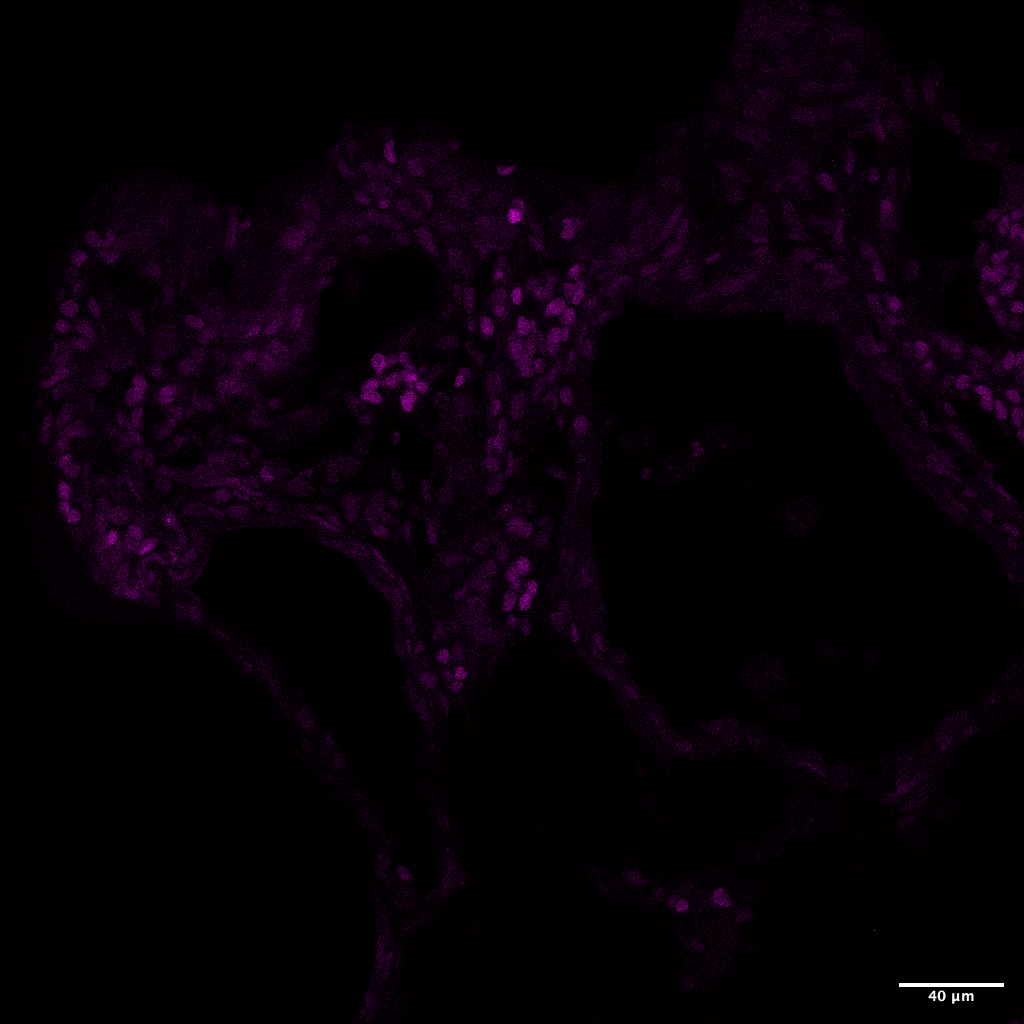

Supplement: Figure 5—source data 4. [file elife-83291-fig5-data4.zip › Figure5_sourcedata_timecourse_mouse/mouse_day54_FOXL2.png]

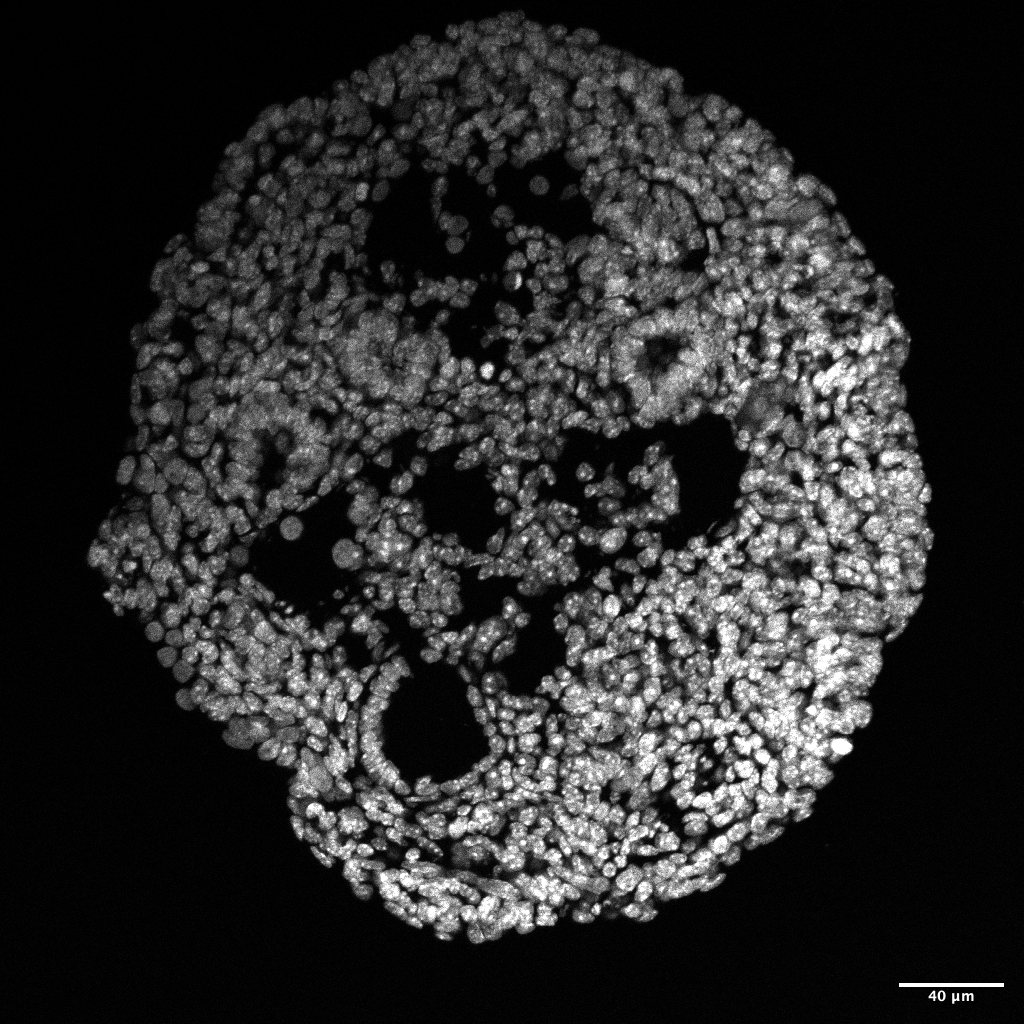

Supplement: Figure 5—source data 4. [file elife-83291-fig5-data4.zip › Figure5_sourcedata_timecourse_mouse/mouse_day4_DAPI.png]

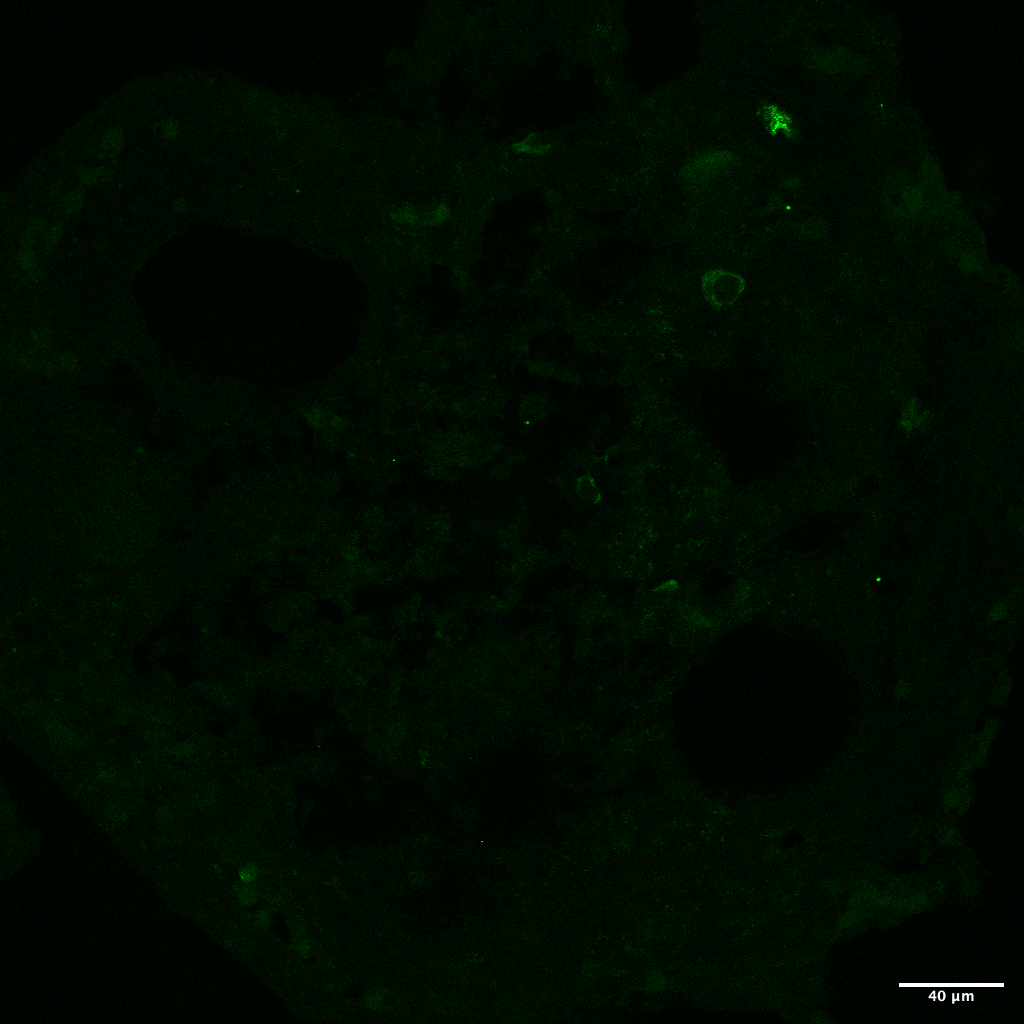

Supplement: Figure 5—source data 4. [file elife-83291-fig5-data4.zip › Figure5_sourcedata_timecourse_mouse/mouse_day8_DAZL.png]

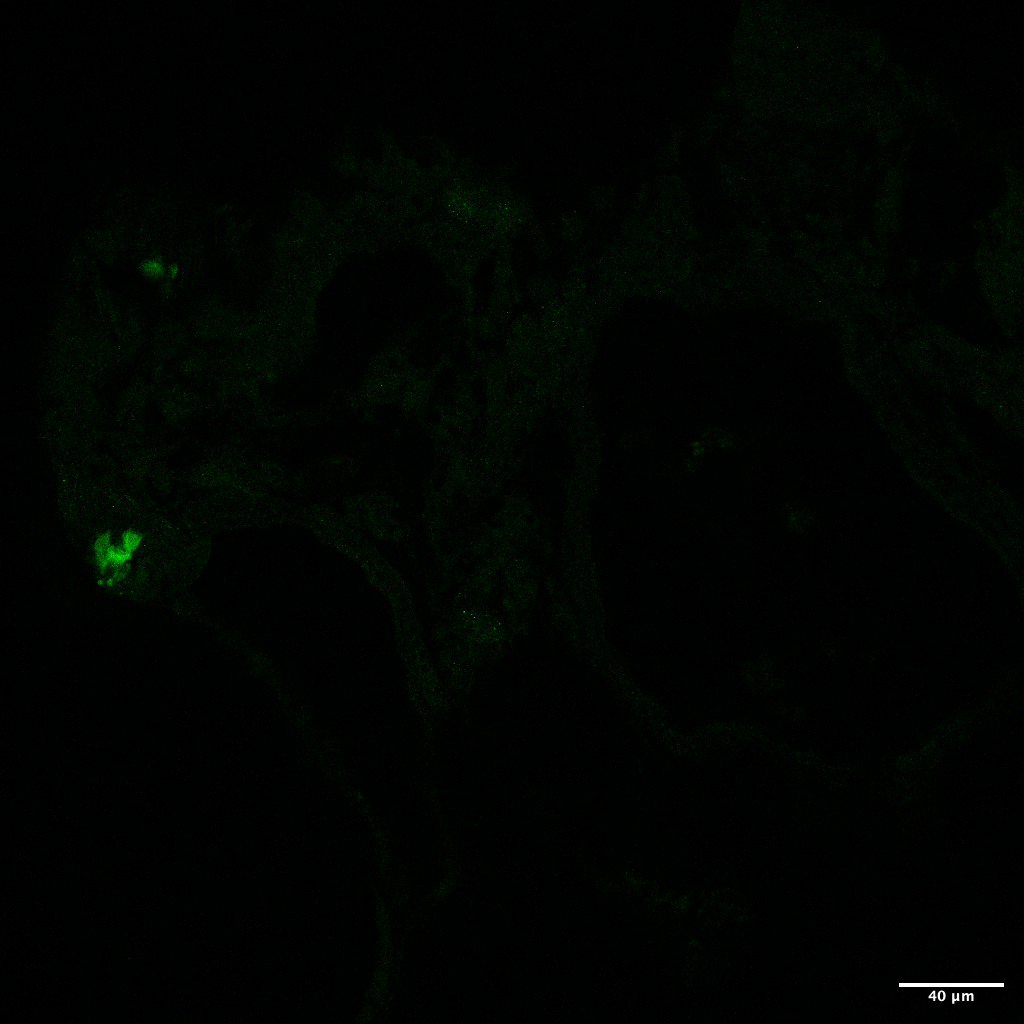

Supplement: Figure 5—source data 4. [file elife-83291-fig5-data4.zip › Figure5_sourcedata_timecourse_mouse/mouse_day54_DAZL.png]

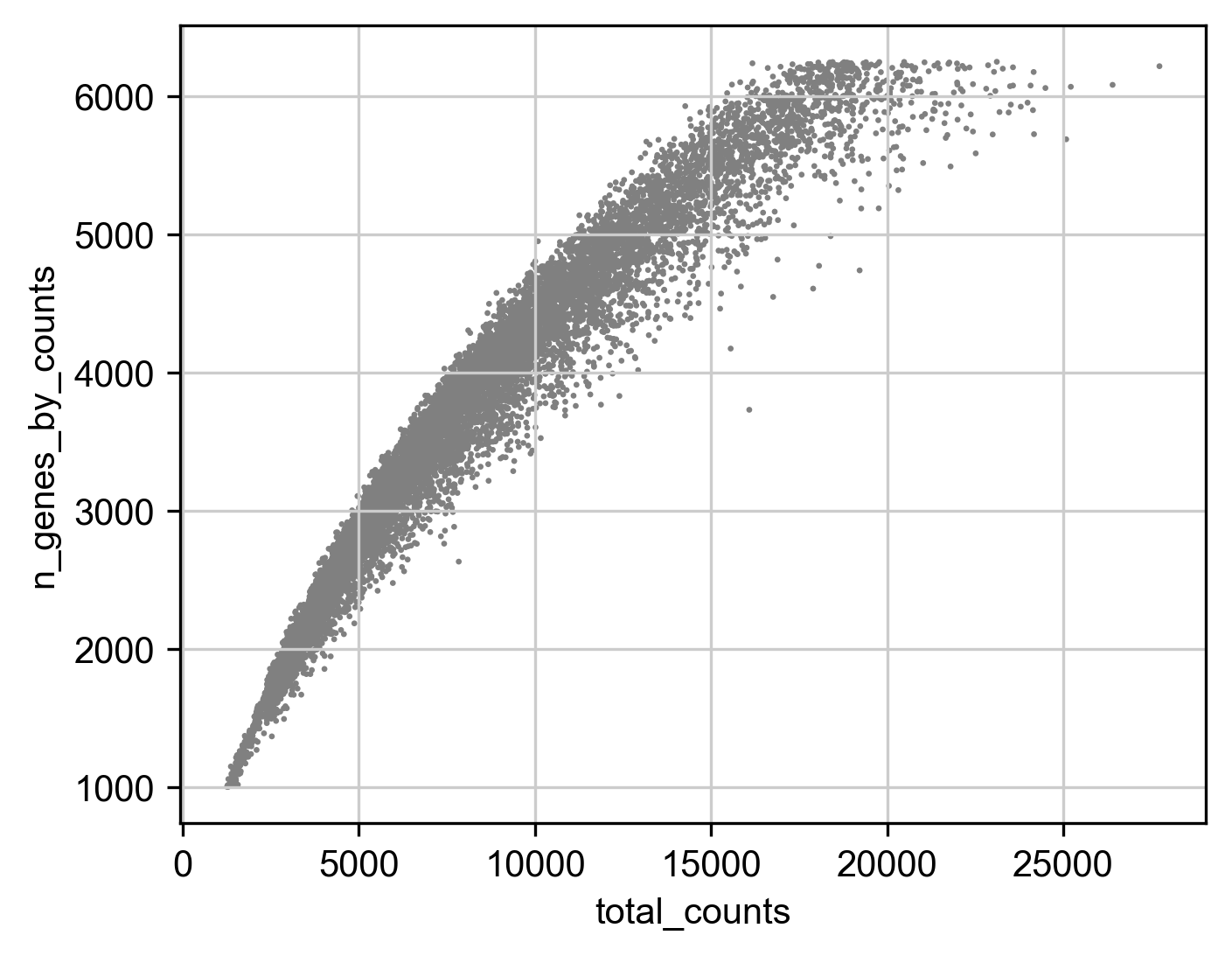

Supplement: Figure 7—source data 1. [file elife-83291-fig7-data1.zip › Figure7_A_and_B/scanpy-figures-D4-ovaroids-only_v2022-12-04/scatter_gene_vs_transcript_counts.png]

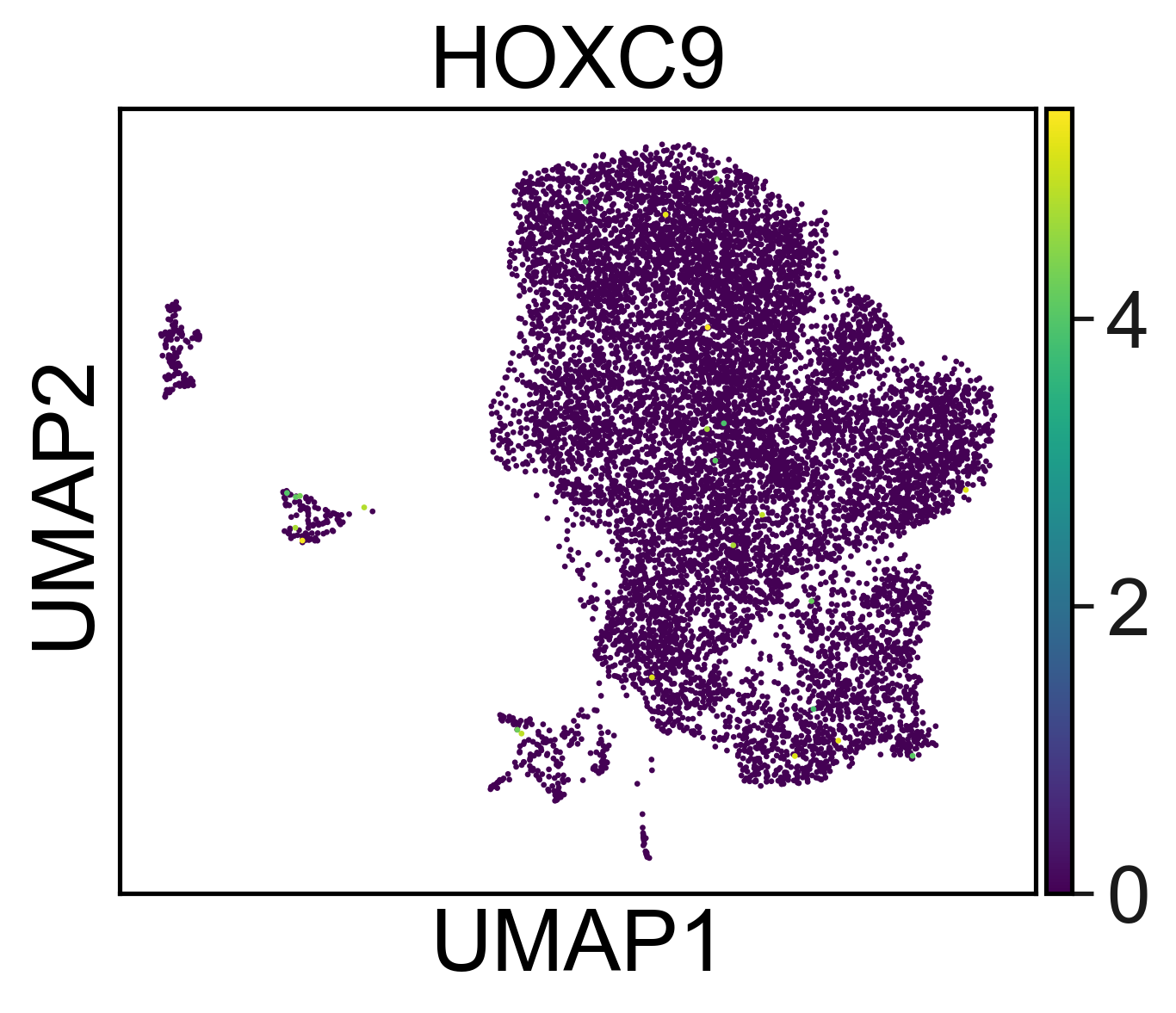

Supplement: Figure 7—source data 1. [file elife-83291-fig7-data1.zip › Figure7_A_and_B/scanpy-figures-D4-ovaroids-only_v2022-12-04/umap_regress_HOXC9.png]

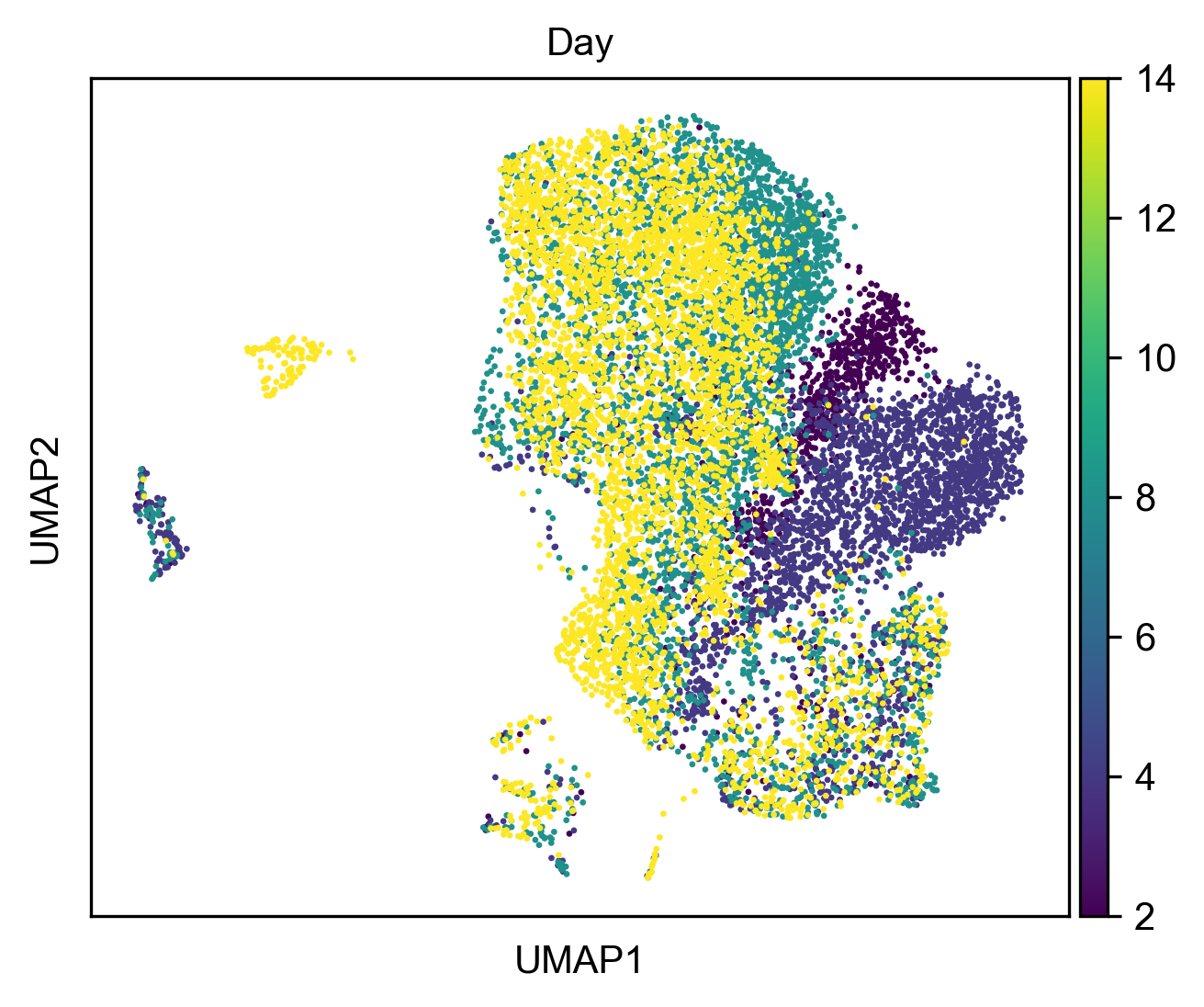

Supplement: Figure 7—source data 1. [file elife-83291-fig7-data1.zip › Figure7_A_and_B/scanpy-figures-D4-ovaroids-only_v2022-12-04/umap_Day_regress.png]

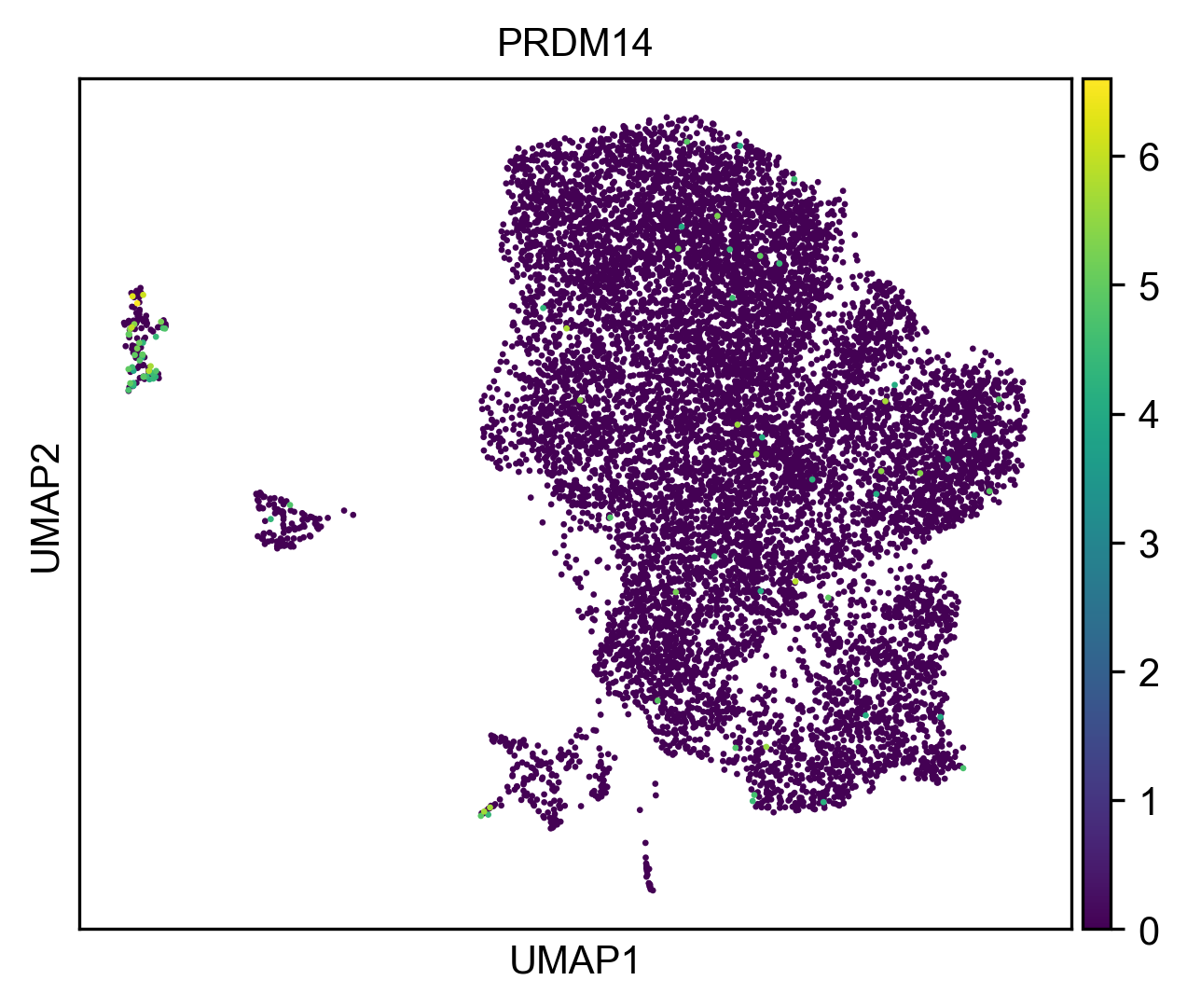

Supplement: Figure 7—source data 1. [file elife-83291-fig7-data1.zip › Figure7_A_and_B/scanpy-figures-D4-ovaroids-only_v2022-12-04/umap_regress_PRDM14.png]

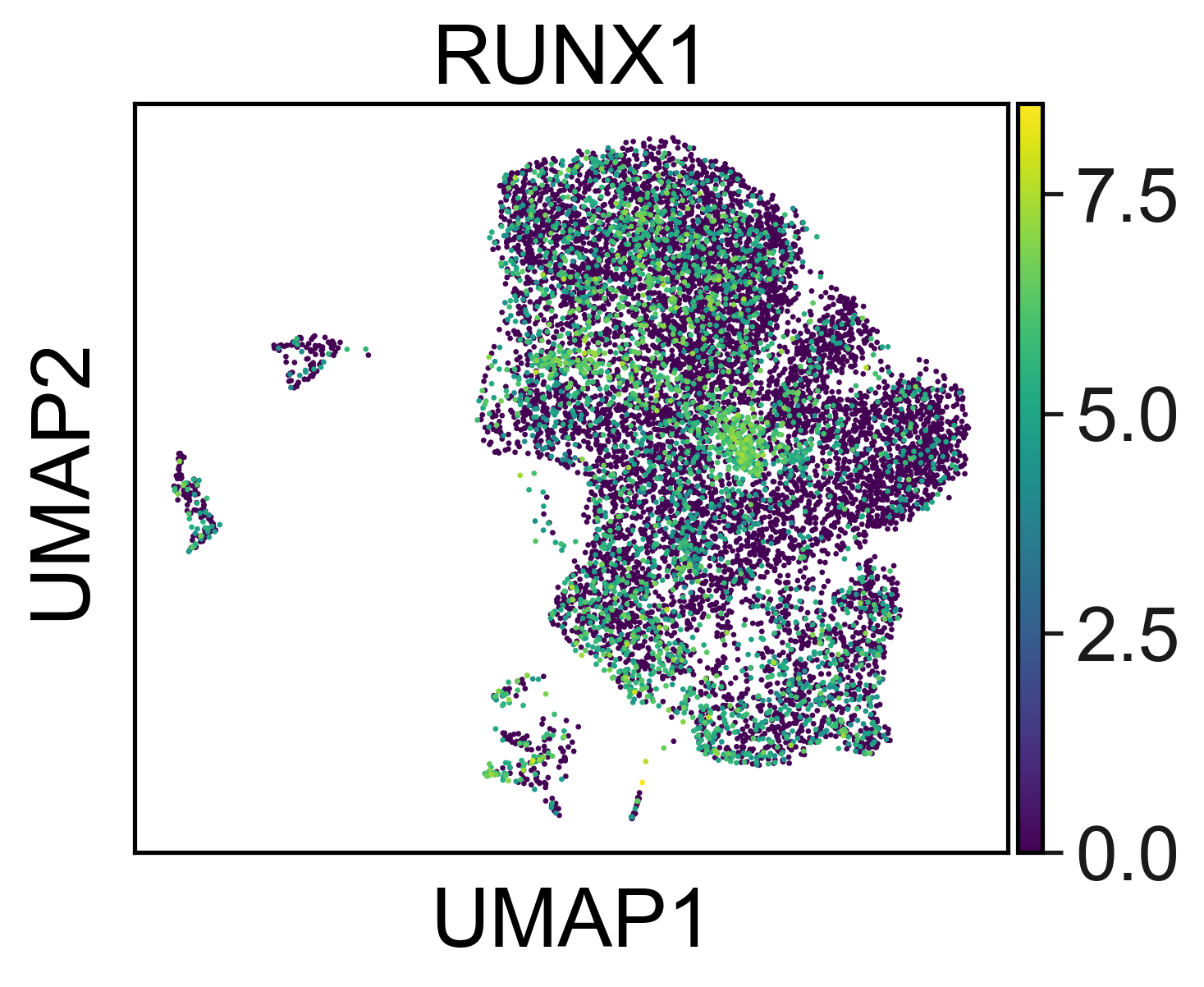

Supplement: Figure 7—source data 1. [file elife-83291-fig7-data1.zip › Figure7_A_and_B/scanpy-figures-D4-ovaroids-only_v2022-12-04/umap_regress_RUNX1.png]

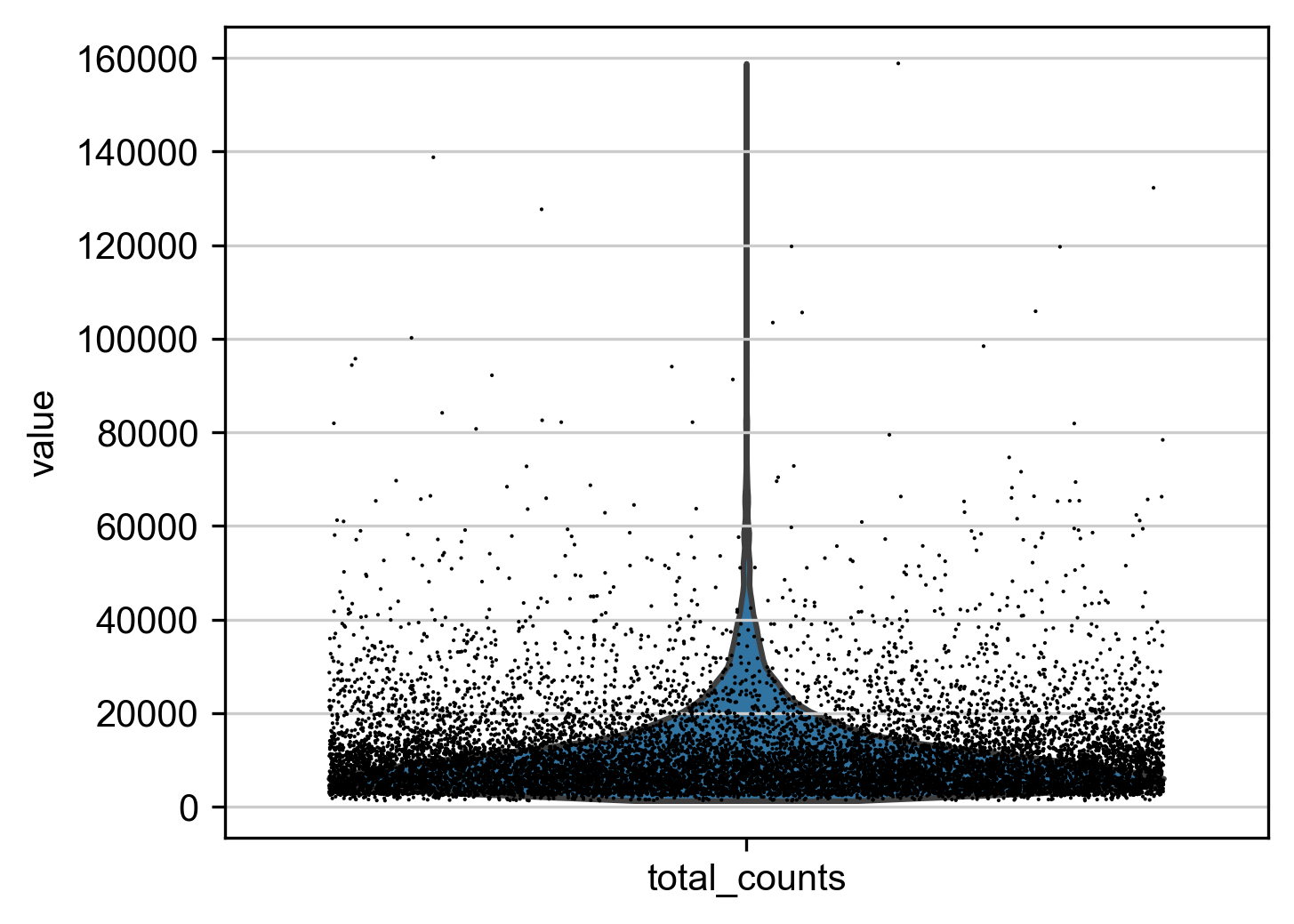

Supplement: Figure 7—source data 1. [file elife-83291-fig7-data1.zip › Figure7_A_and_B/scanpy-figures-D4-ovaroids-only_v2022-12-04/violin_total_counts.png]

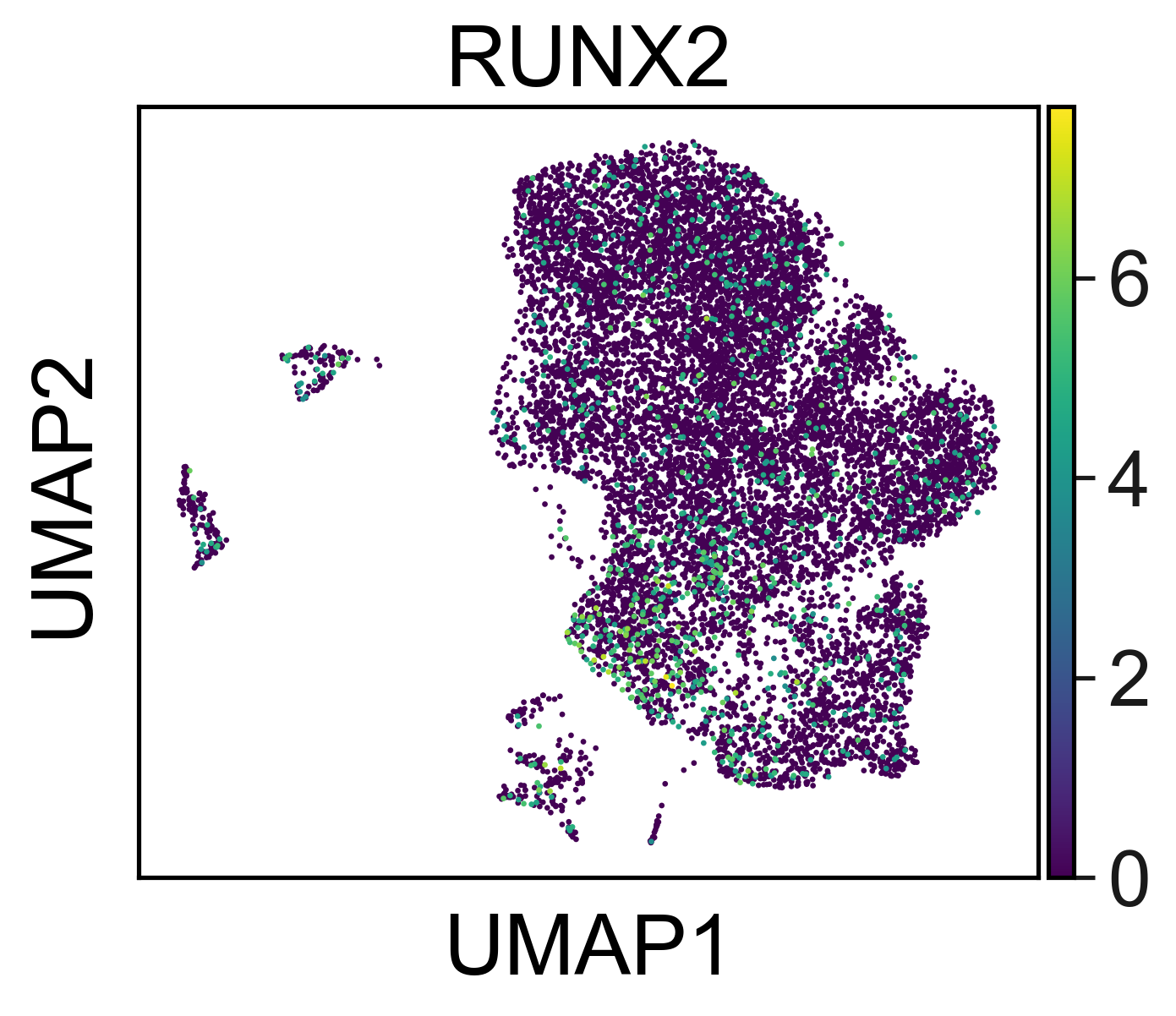

Supplement: Figure 7—source data 1. [file elife-83291-fig7-data1.zip › Figure7_A_and_B/scanpy-figures-D4-ovaroids-only_v2022-12-04/umap_regress_RUNX2.png]

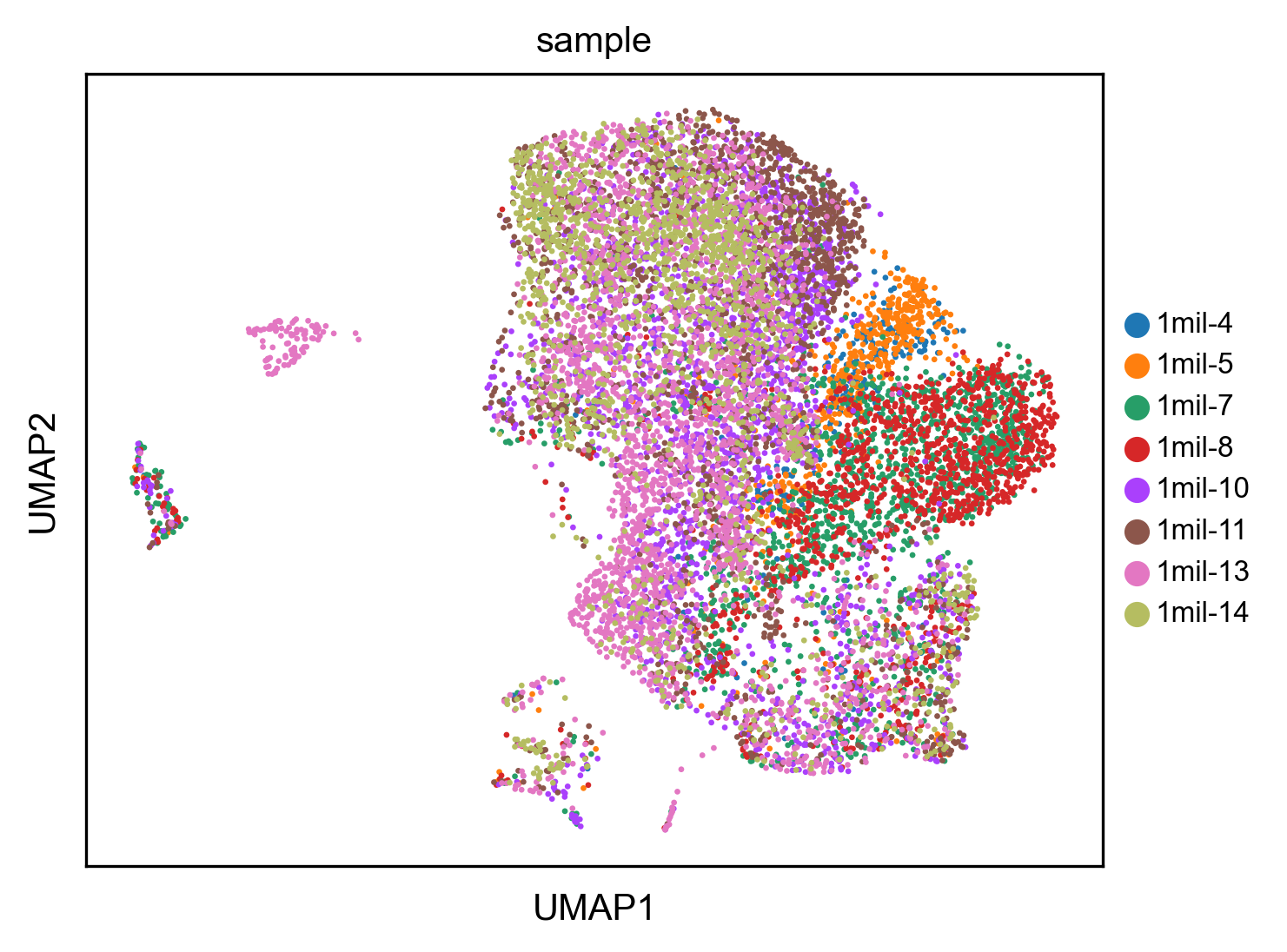

Supplement: Figure 7—source data 1. [file elife-83291-fig7-data1.zip › Figure7_A_and_B/scanpy-figures-D4-ovaroids-only_v2022-12-04/umap_sample_regress.png]

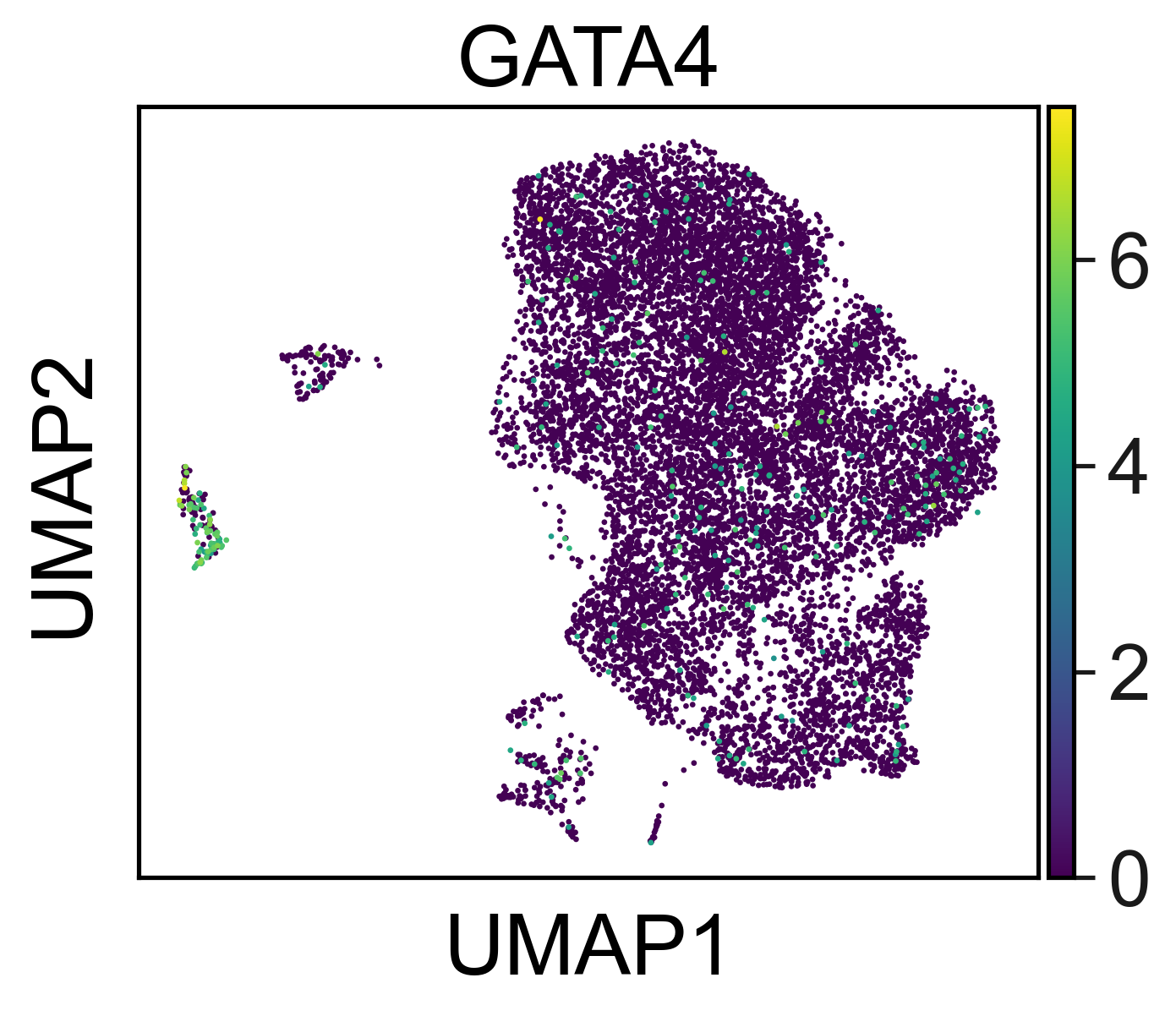

Supplement: Figure 7—source data 1. [file elife-83291-fig7-data1.zip › Figure7_A_and_B/scanpy-figures-D4-ovaroids-only_v2022-12-04/umap_regress_GATA4.png]

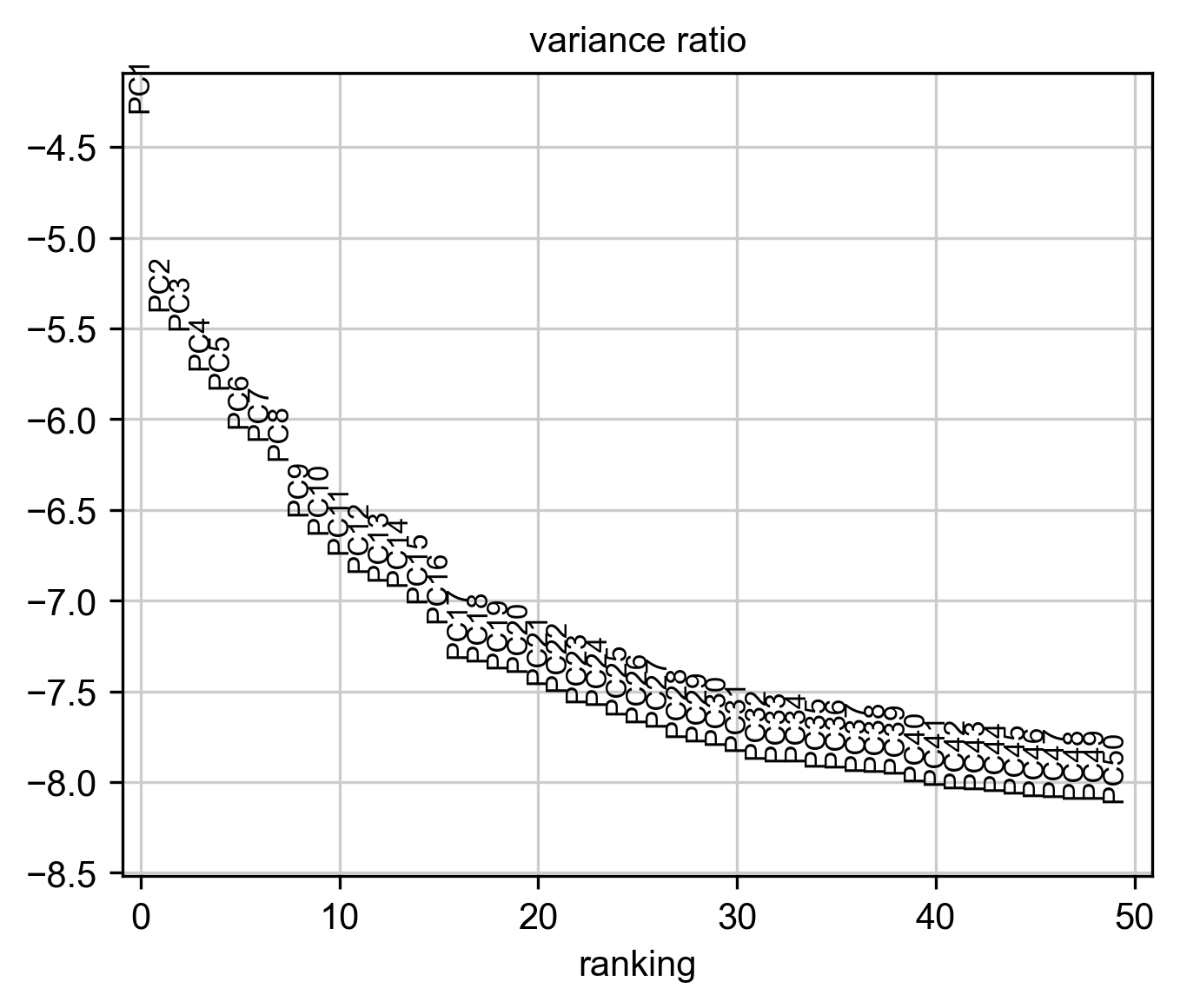

Supplement: Figure 7—source data 1. [file elife-83291-fig7-data1.zip › Figure7_A_and_B/scanpy-figures-D4-ovaroids-only_v2022-12-04/pca_variance_ratio.png]

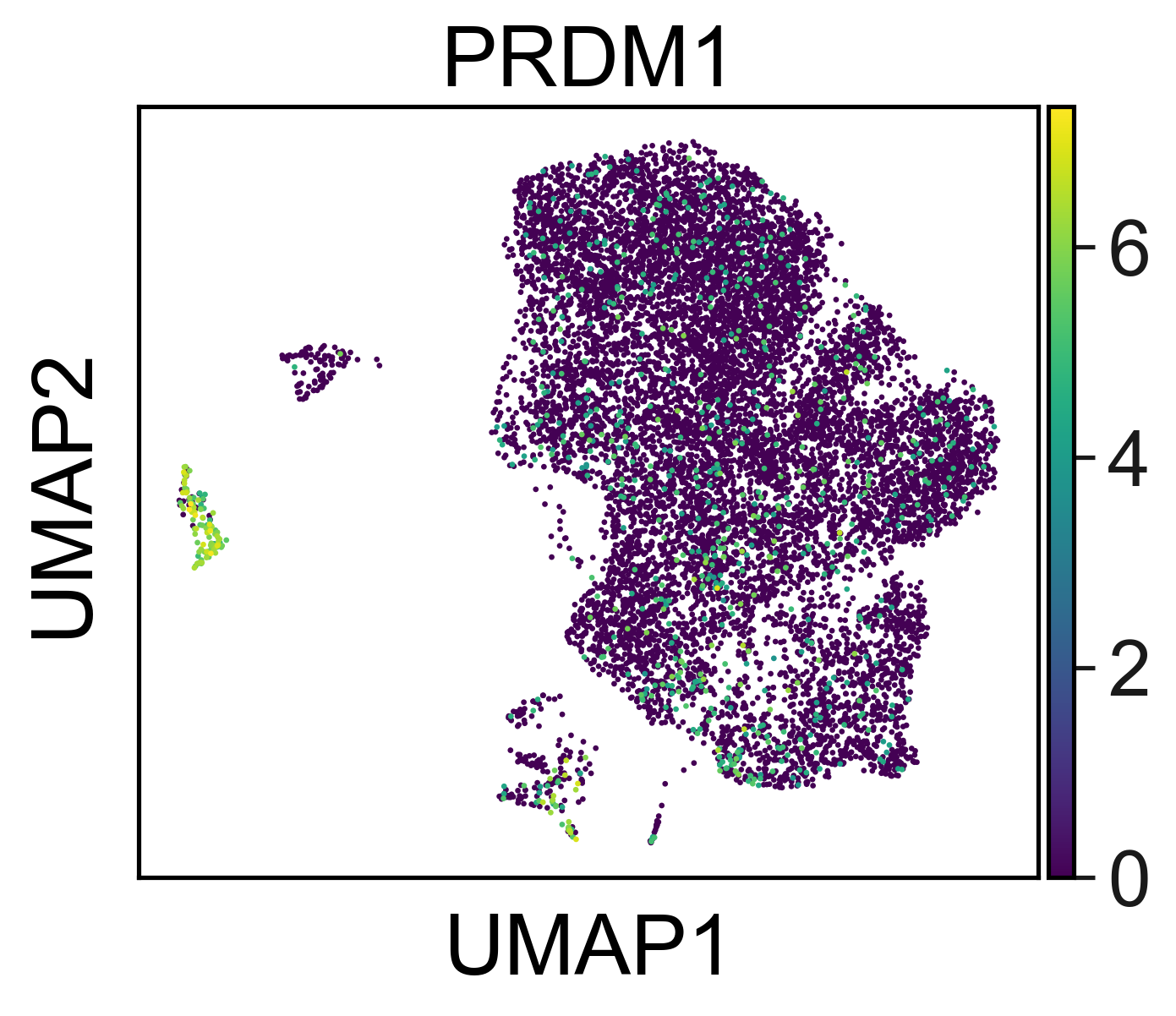

Supplement: Figure 7—source data 1. [file elife-83291-fig7-data1.zip › Figure7_A_and_B/scanpy-figures-D4-ovaroids-only_v2022-12-04/umap_regress_PRDM1.png]

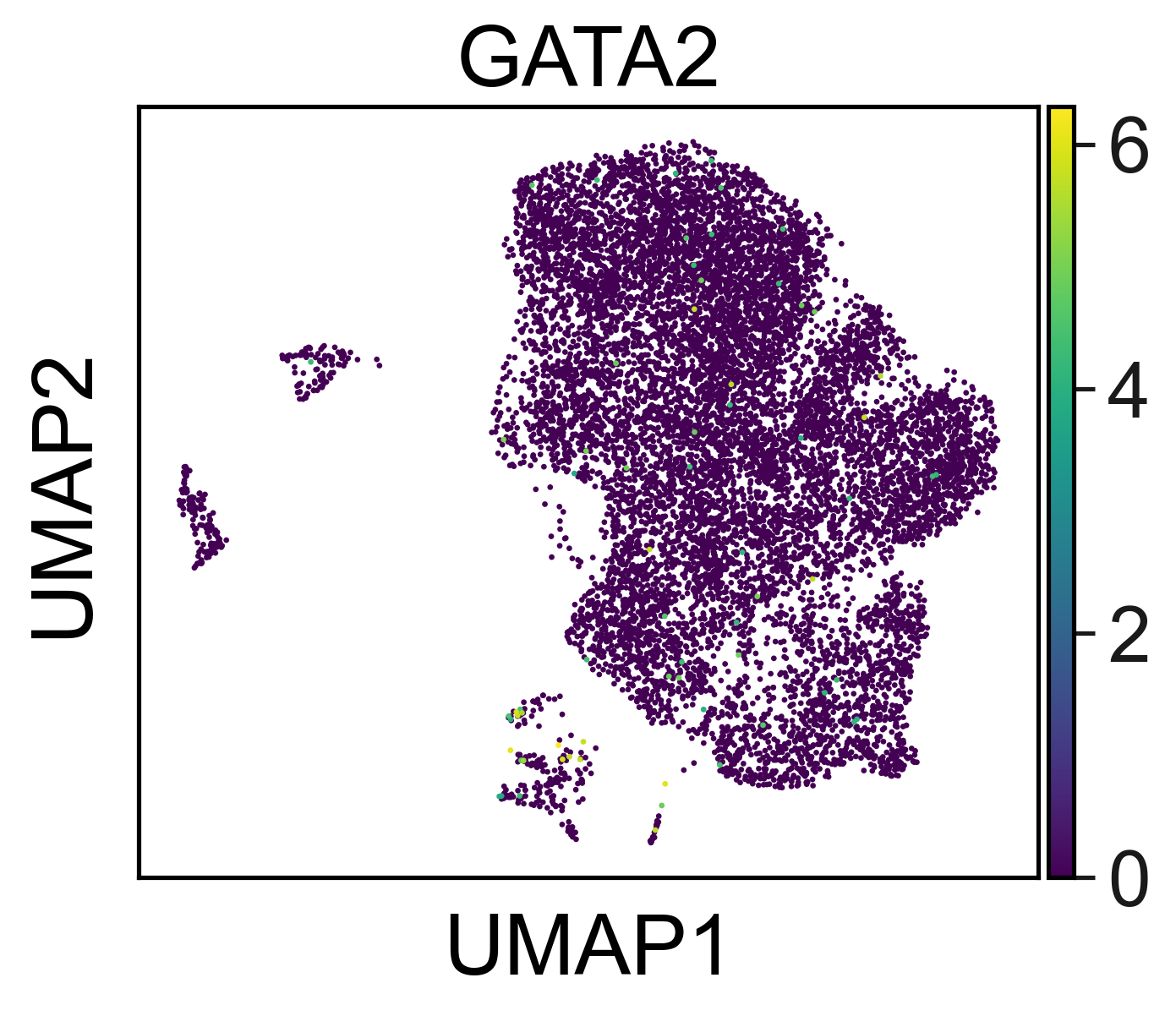

Supplement: Figure 7—source data 1. [file elife-83291-fig7-data1.zip › Figure7_A_and_B/scanpy-figures-D4-ovaroids-only_v2022-12-04/umap_regress_GATA2.png]

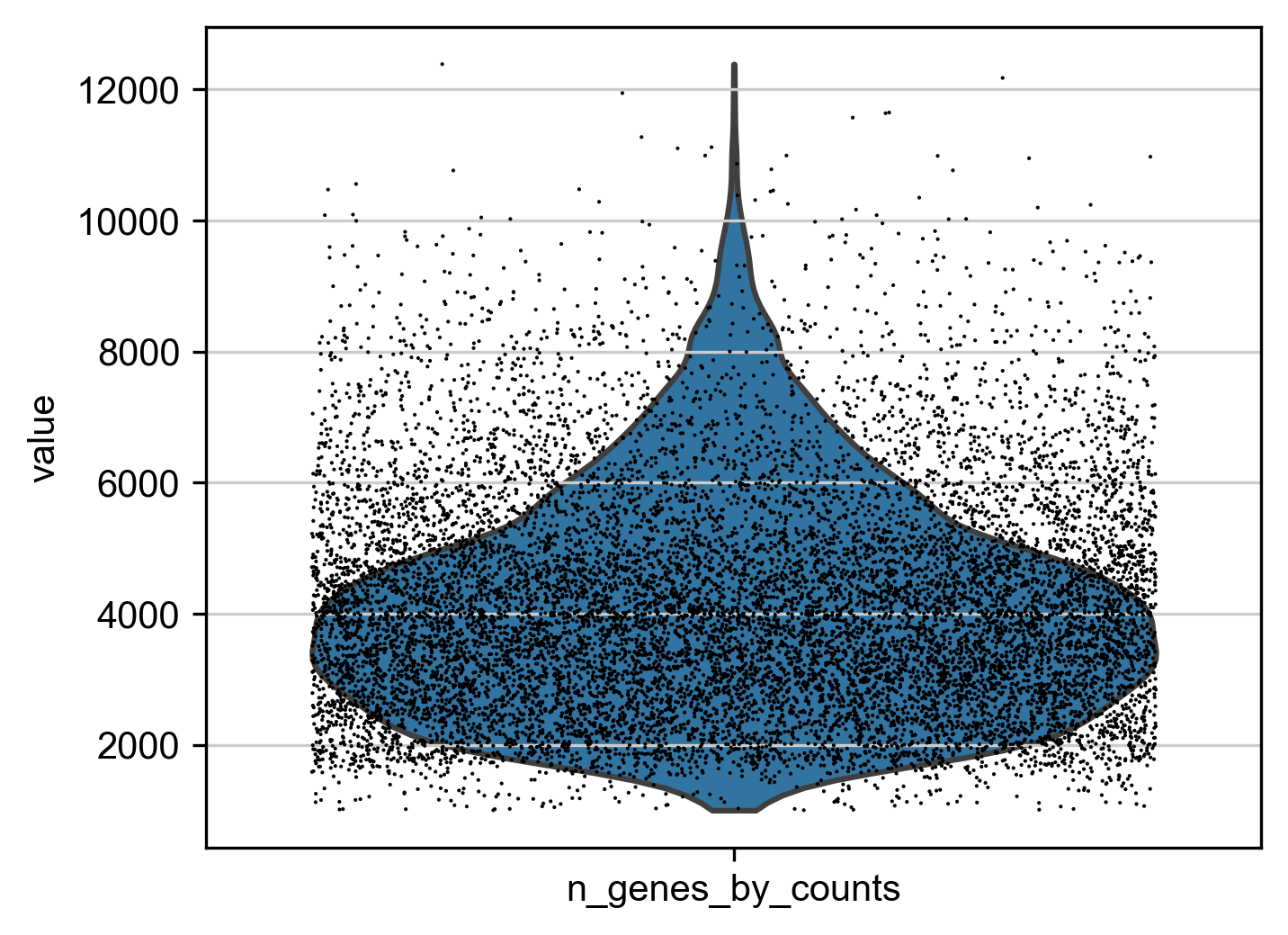

Supplement: Figure 7—source data 1. [file elife-83291-fig7-data1.zip › Figure7_A_and_B/scanpy-figures-D4-ovaroids-only_v2022-12-04/violin_n_genes.png]

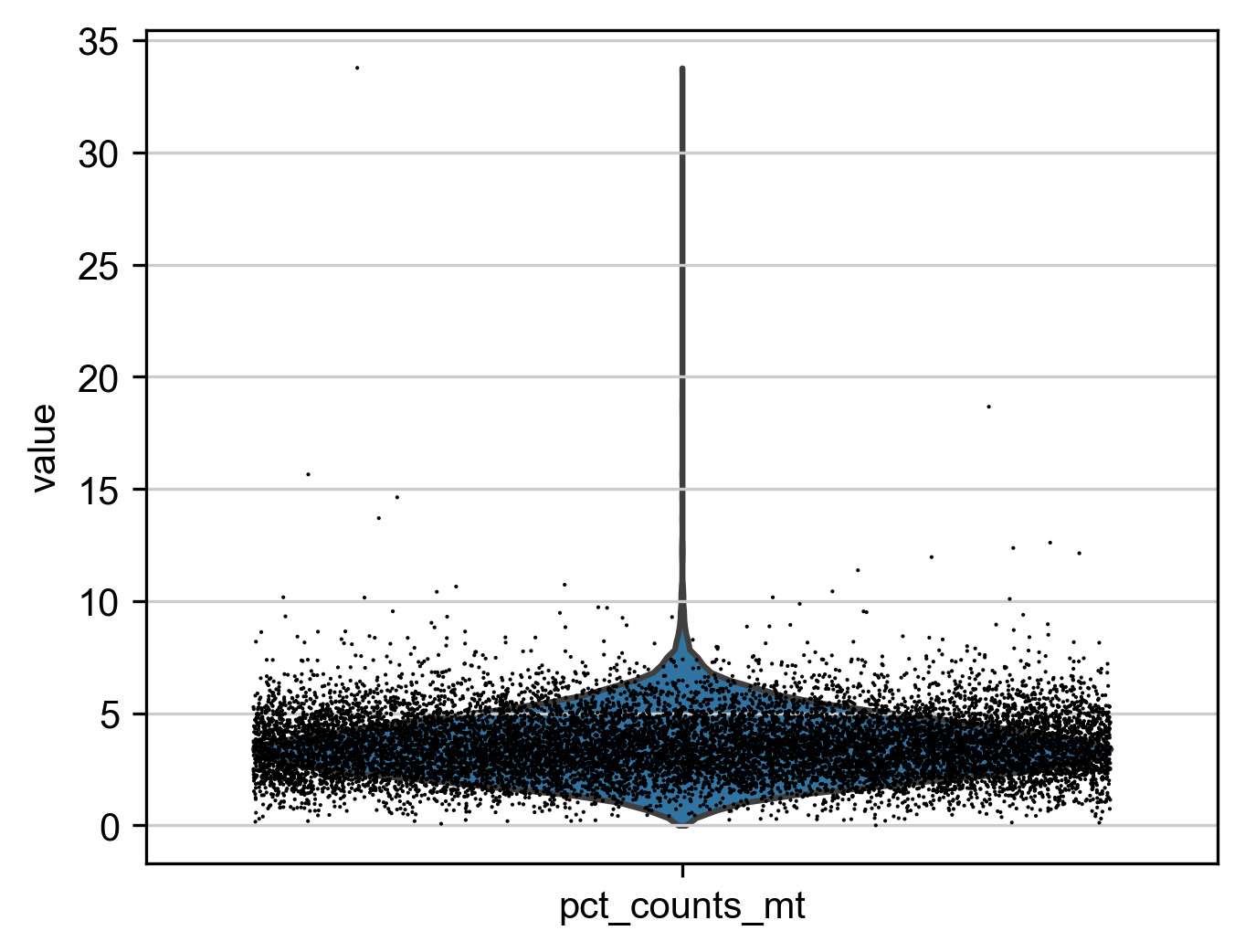

Supplement: Figure 7—source data 1. [file elife-83291-fig7-data1.zip › Figure7_A_and_B/scanpy-figures-D4-ovaroids-only_v2022-12-04/violin_mito_pct.png]

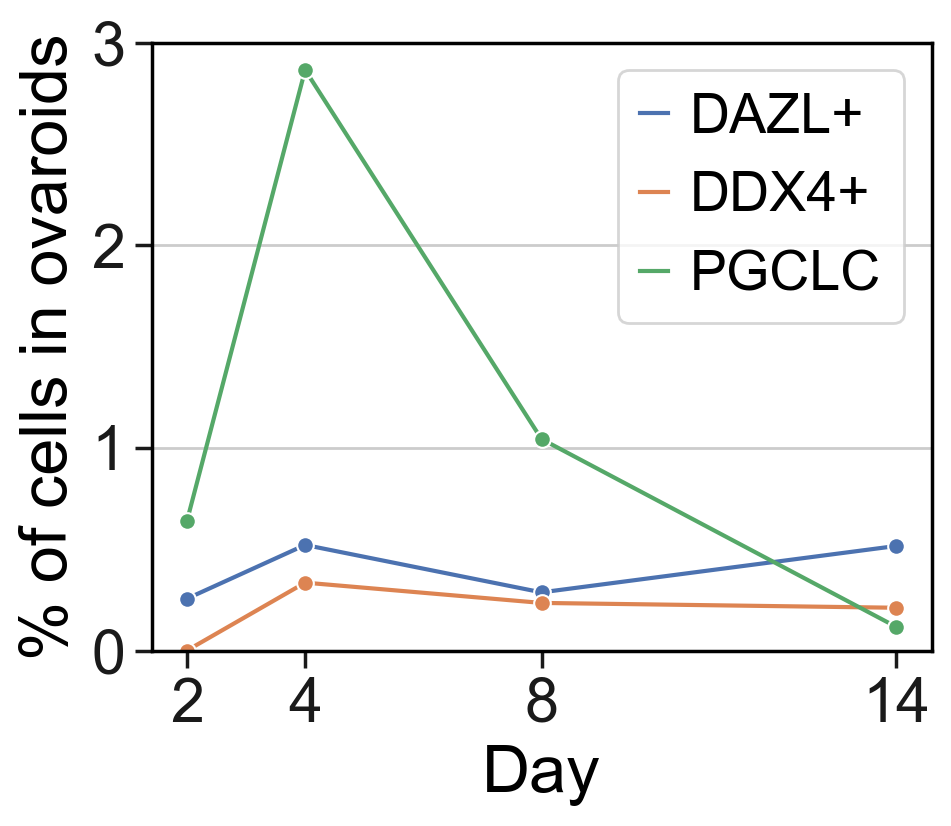

Supplement: Figure 7—source data 1. [file elife-83291-fig7-data1.zip › Figure7_A_and_B/scanpy-figures-D4-ovaroids-only_v2022-12-04/ovaroid_time_plot.png]

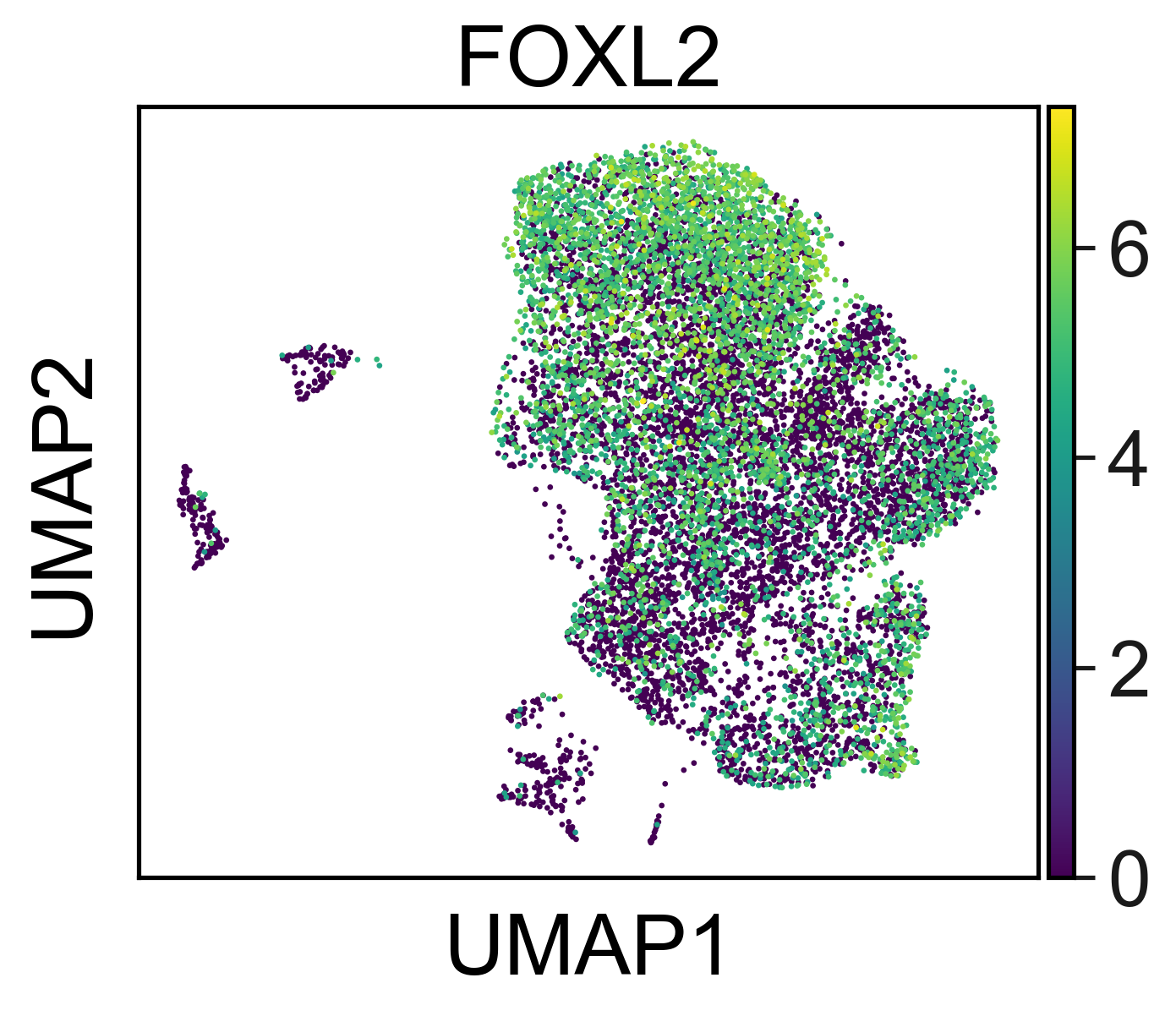

Supplement: Figure 7—source data 1. [file elife-83291-fig7-data1.zip › Figure7_A_and_B/scanpy-figures-D4-ovaroids-only_v2022-12-04/umap_regress_FOXL2.png]

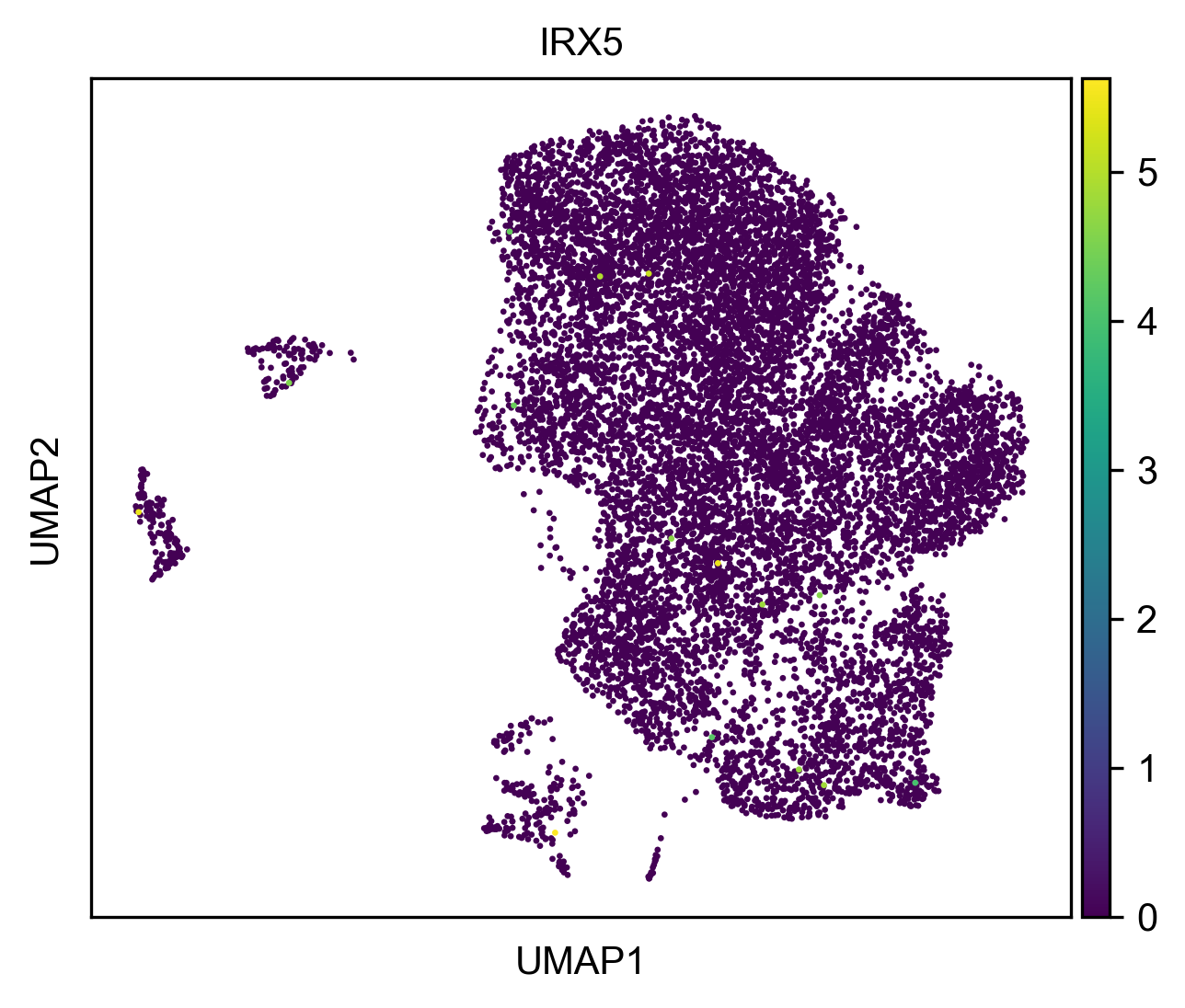

Supplement: Figure 7—source data 1. [file elife-83291-fig7-data1.zip › Figure7_A_and_B/scanpy-figures-D4-ovaroids-only_v2022-12-04/umap_regress_IRX5.png]

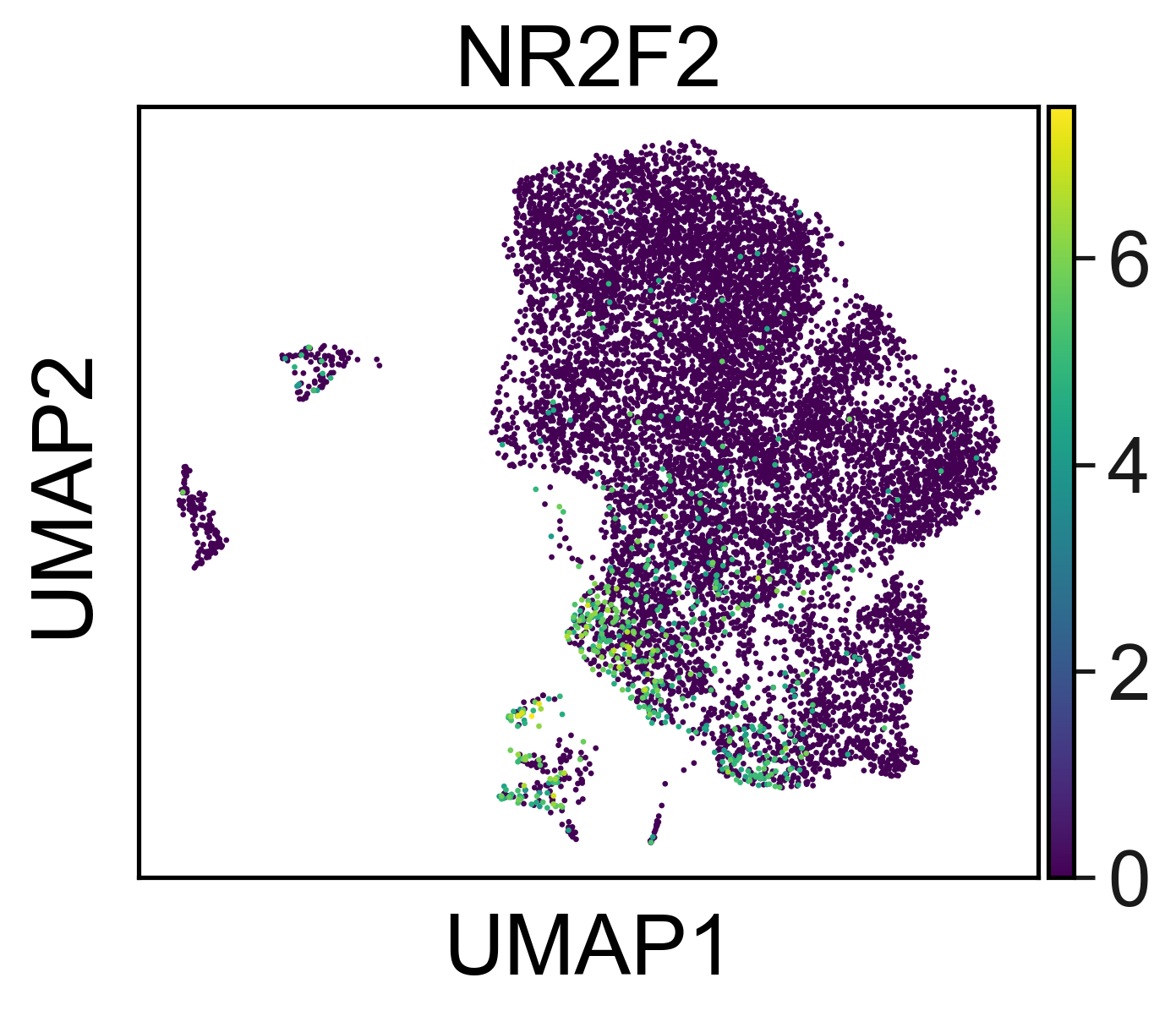

Supplement: Figure 7—source data 1. [file elife-83291-fig7-data1.zip › Figure7_A_and_B/scanpy-figures-D4-ovaroids-only_v2022-12-04/umap_regress_NR2F2.png]

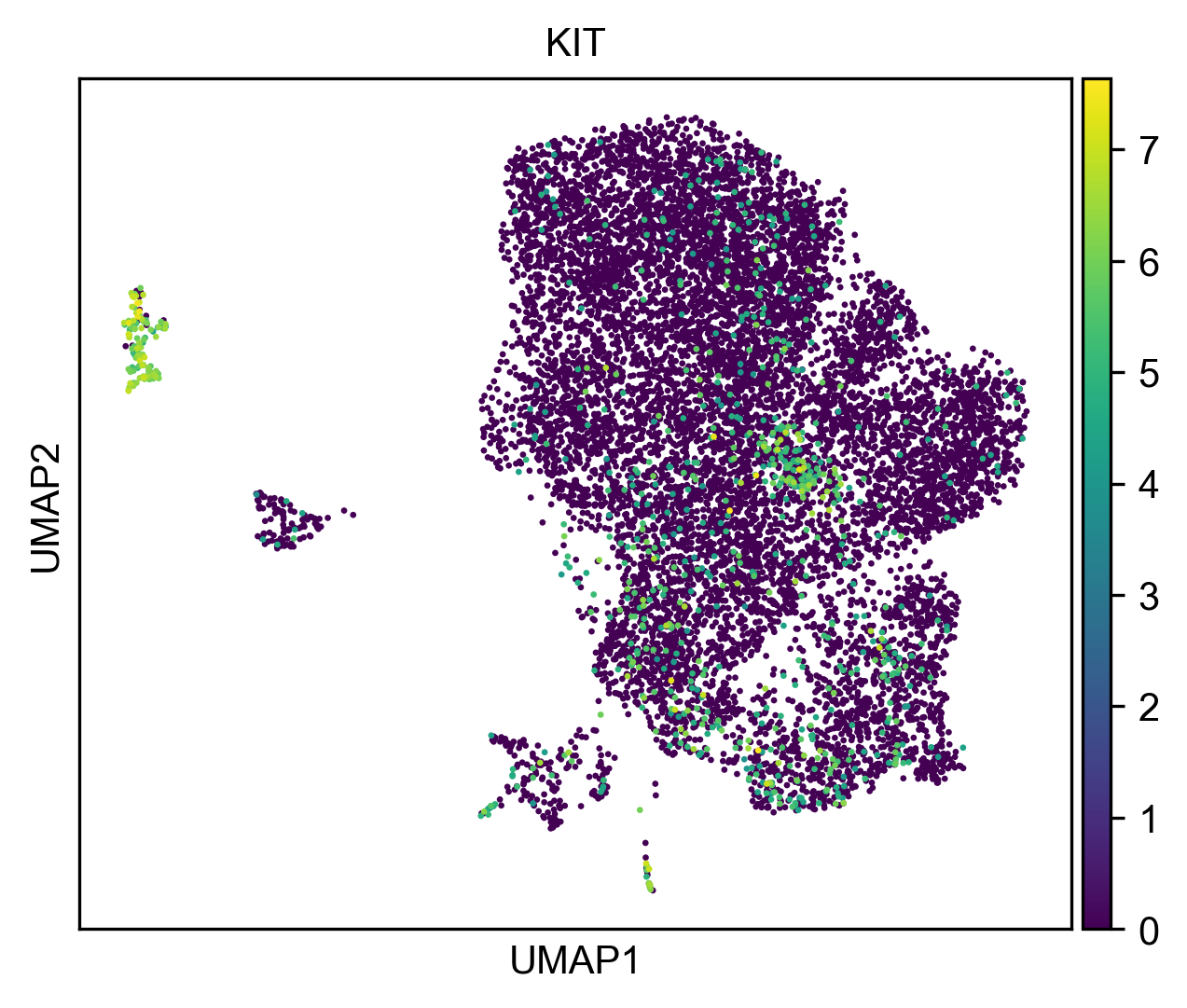

Supplement: Figure 7—source data 1. [file elife-83291-fig7-data1.zip › Figure7_A_and_B/scanpy-figures-D4-ovaroids-only_v2022-12-04/umap_regress_KIT.png]

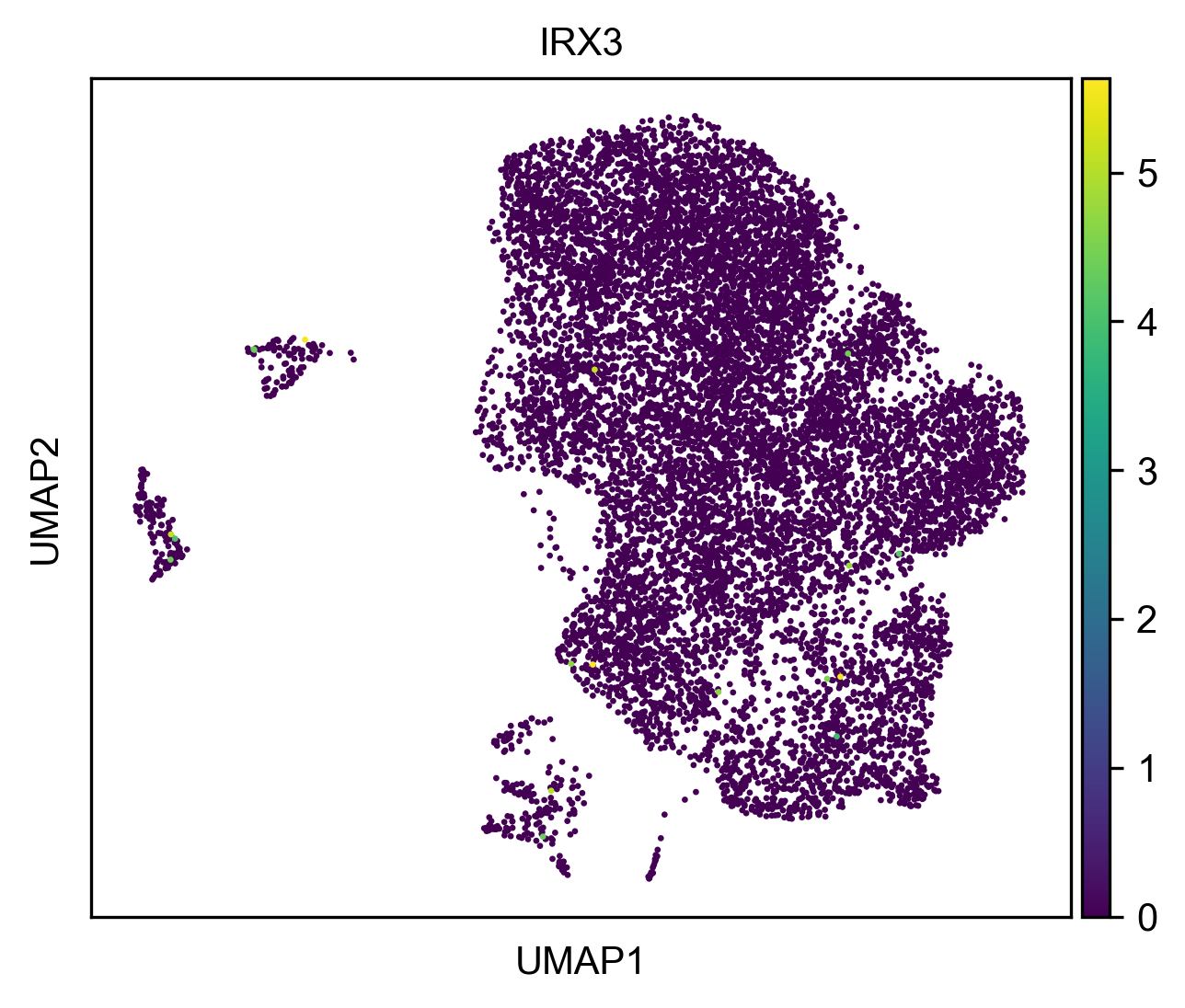

Supplement: Figure 7—source data 1. [file elife-83291-fig7-data1.zip › Figure7_A_and_B/scanpy-figures-D4-ovaroids-only_v2022-12-04/umap_regress_IRX3.png]
