# Supplementary figures and images for: Directed differentiation of human iPSCs to functional ovarian granulosa-like cells via transcription factor overexpression (part 3 of 3)
Source: eLife. 2023 Feb 21;12:e83291. doi: 10.7554/eLife.83291 (PMC9943069; doi:10.7554/eLife.83291)

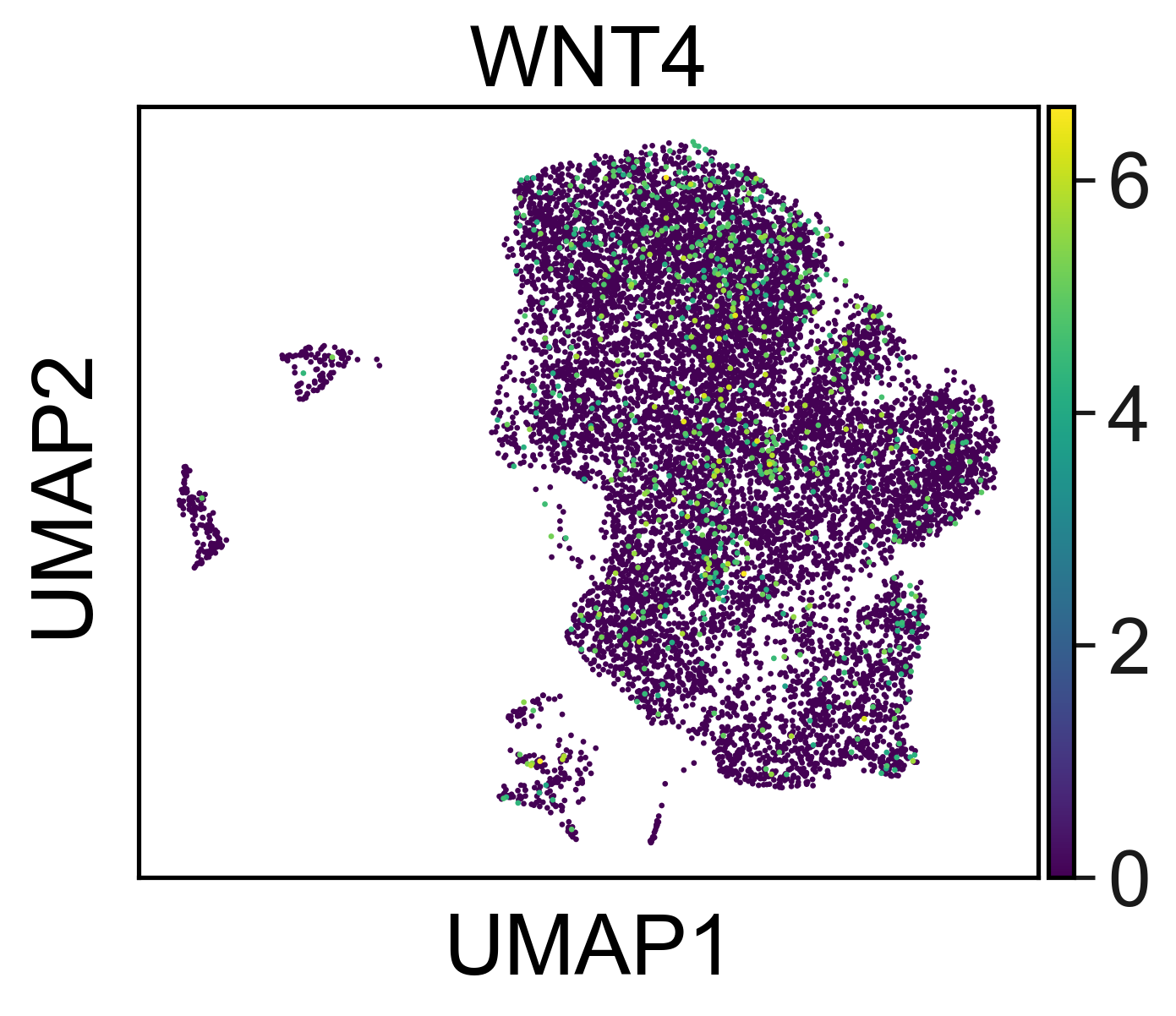

Supplement: Figure 7—source data 1. [file elife-83291-fig7-data1.zip › Figure7_A_and_B/scanpy-figures-D4-ovaroids-only_v2022-12-04/umap_regress_WNT4.png]

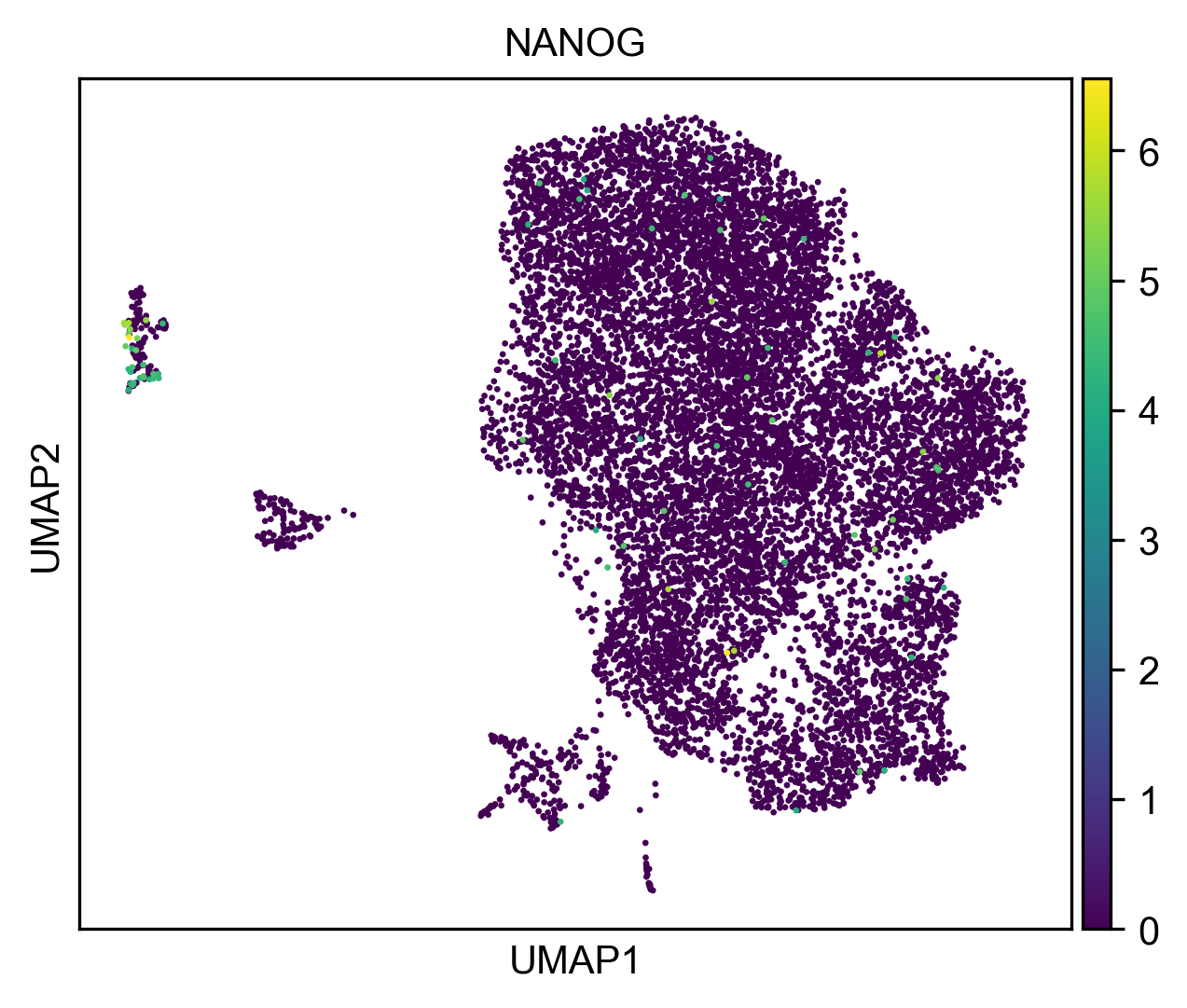

Supplement: Figure 7—source data 1. [file elife-83291-fig7-data1.zip › Figure7_A_and_B/scanpy-figures-D4-ovaroids-only_v2022-12-04/umap_regress_NANOG.png]

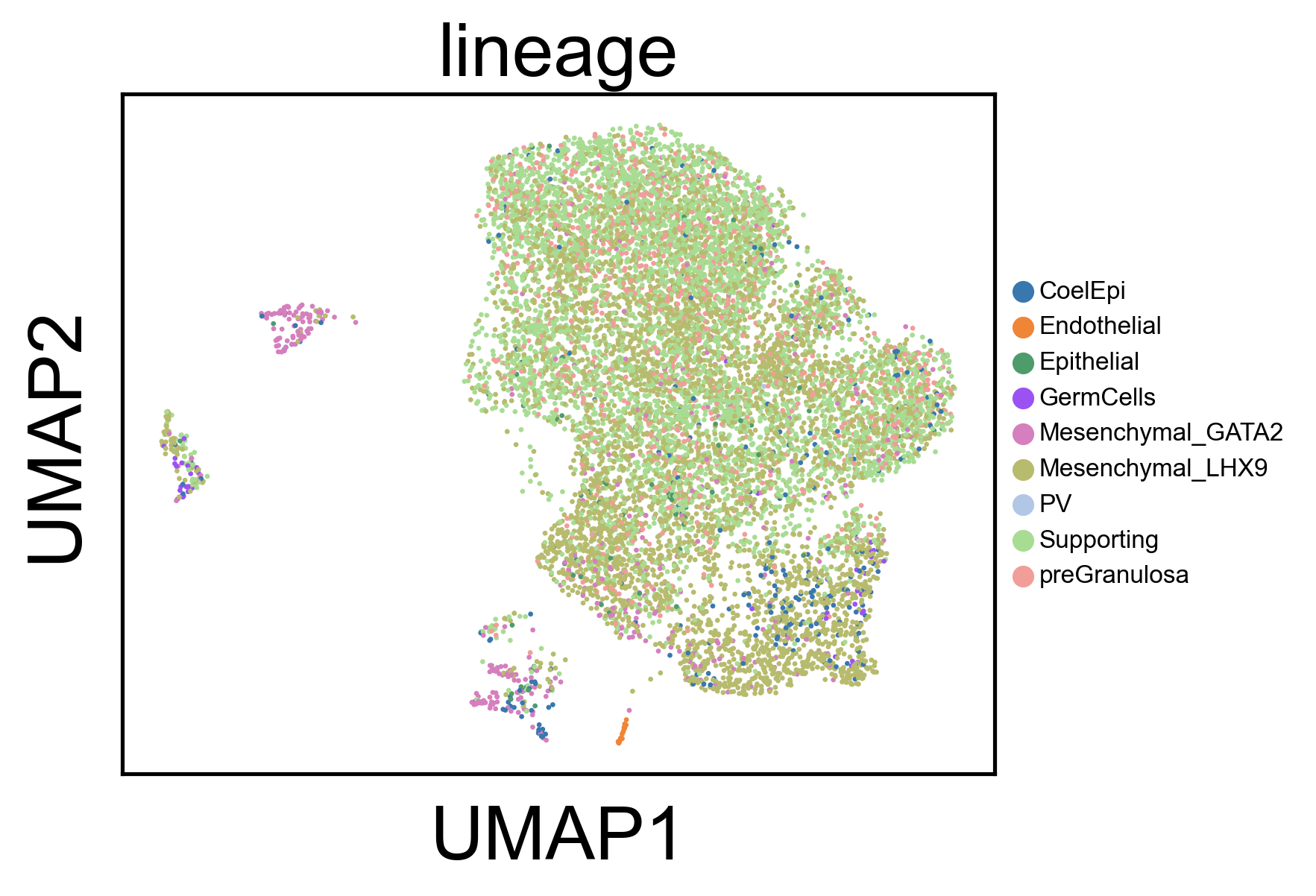

Supplement: Figure 7—source data 1. [file elife-83291-fig7-data1.zip › Figure7_A_and_B/scanpy-figures-D4-ovaroids-only_v2022-12-04/umapumap_project_lineage.png]

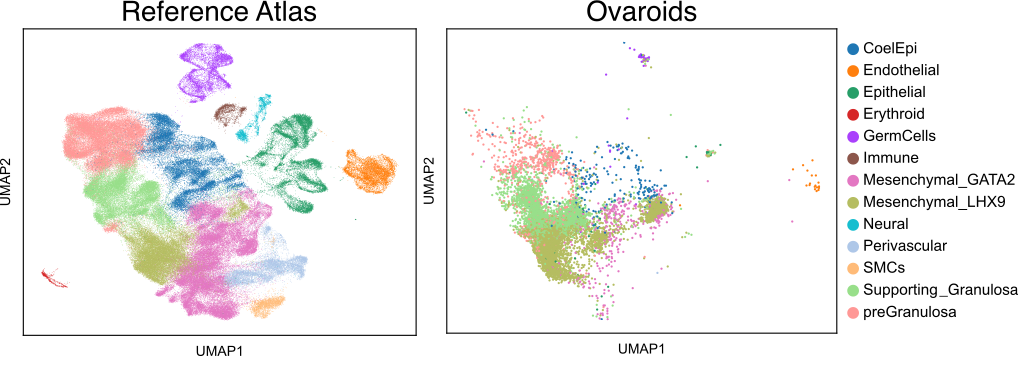

Supplement: Figure 7—source data 1. [file elife-83291-fig7-data1.zip › Figure7_C_and_D/INGEST.png]

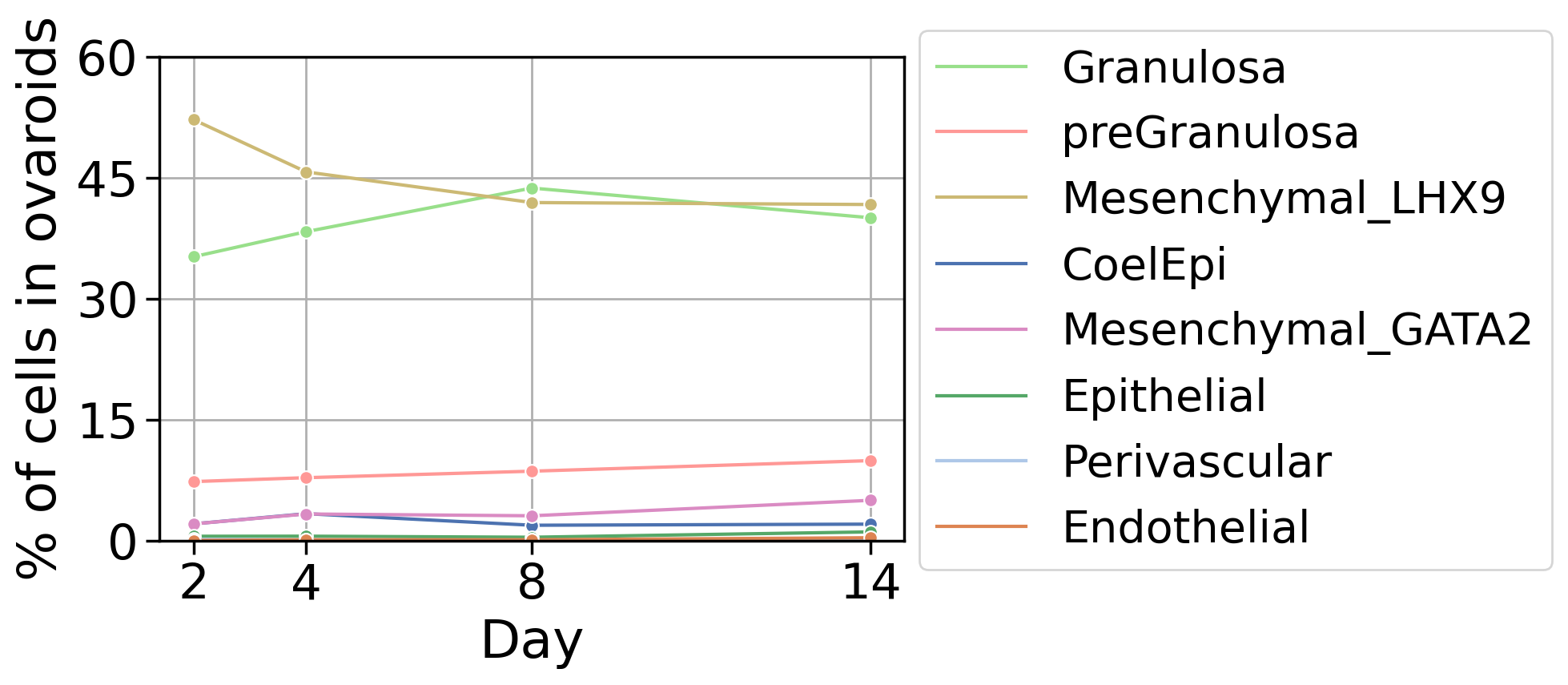

Supplement: Figure 7—source data 1. [file elife-83291-fig7-data1.zip › Figure7_C_and_D/ovaroid_soma_time_plot.png]

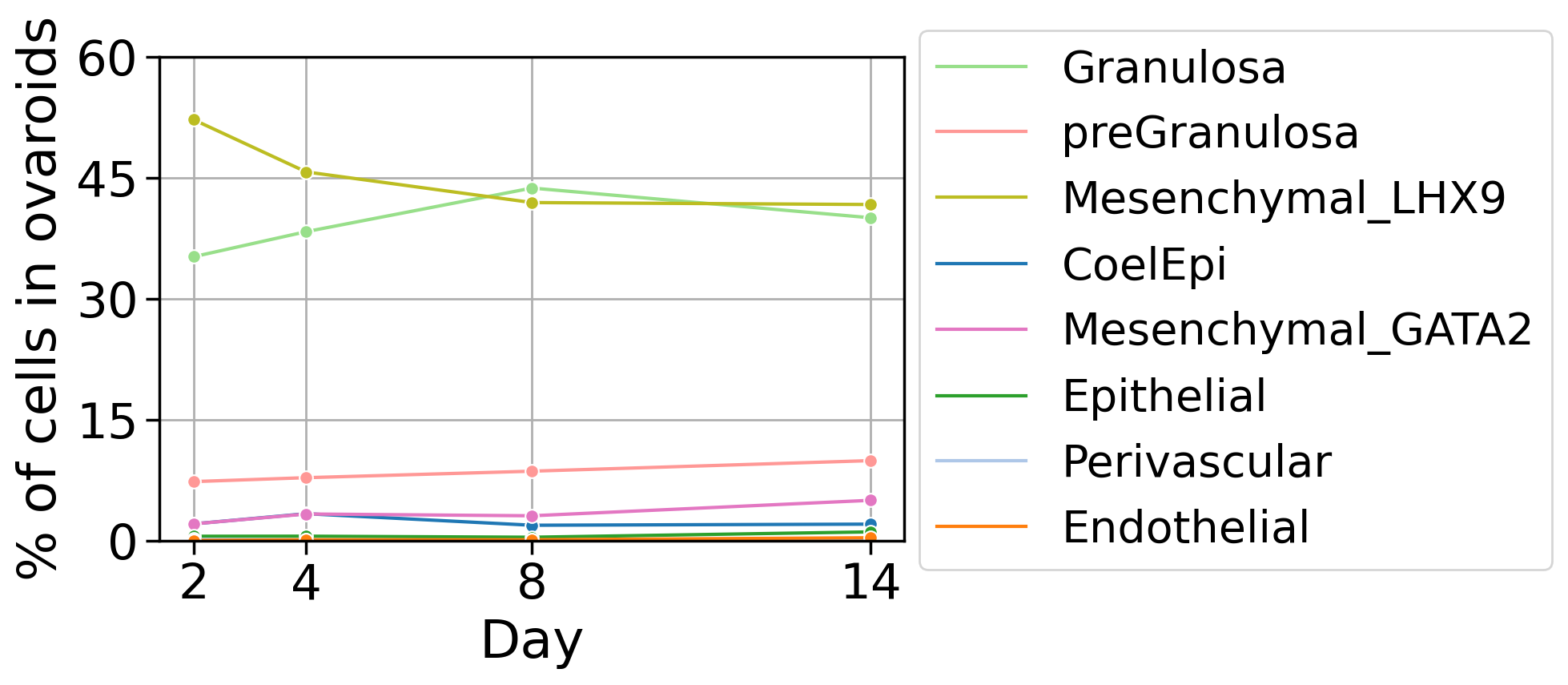

Supplement: Figure 7—source data 1. [file elife-83291-fig7-data1.zip › Figure7_C_and_D/ovaroid_soma_time_plot_colorblind.png]

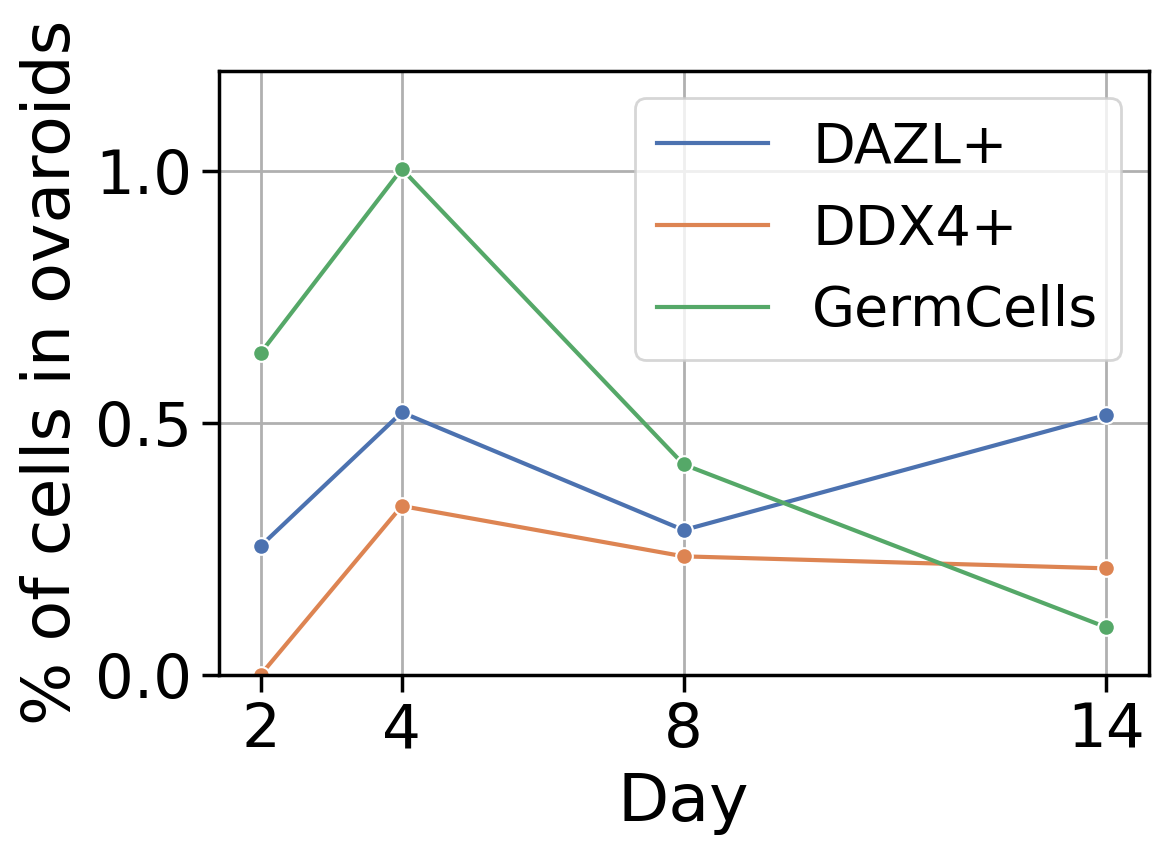

Supplement: Figure 7—source data 1. [file elife-83291-fig7-data1.zip › Figure7_C_and_D/ovaroid_germcell_time_plot.png]

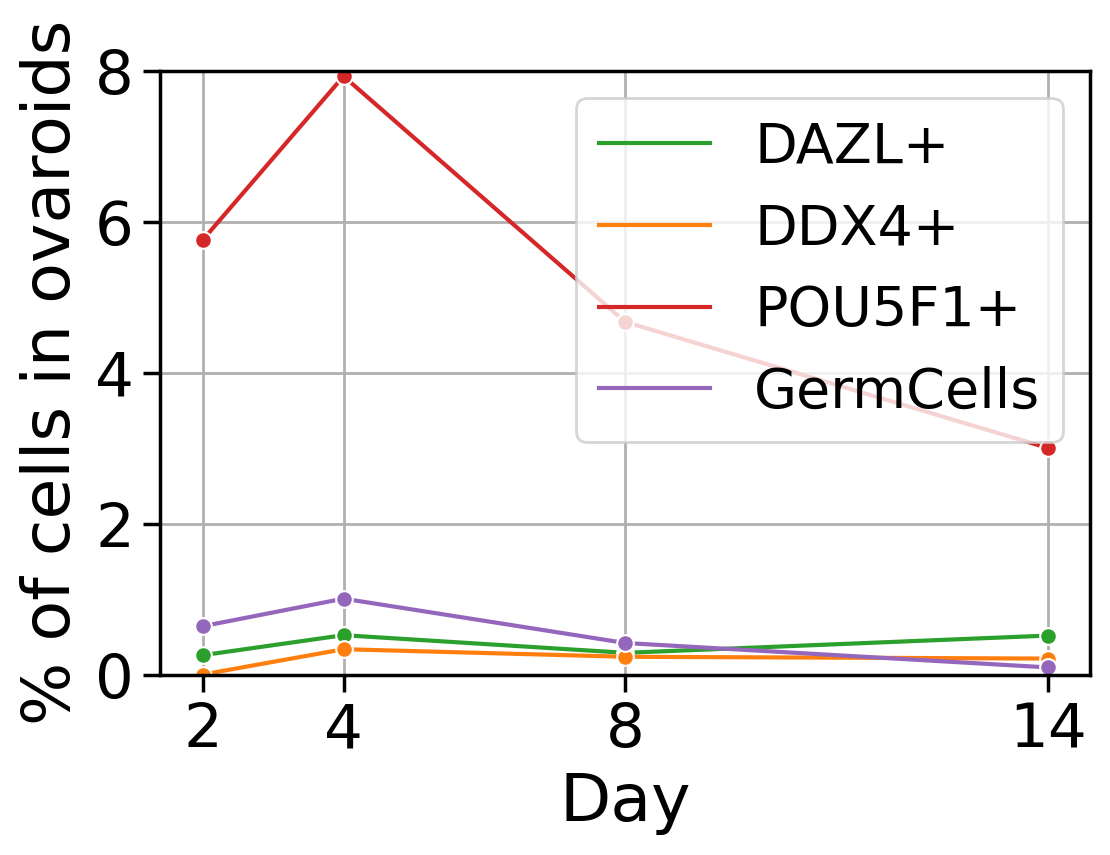

Supplement: Figure 7—source data 1. [file elife-83291-fig7-data1.zip › Figure7_C_and_D/ovaroid_germcell_time_plot_colorblind.png]

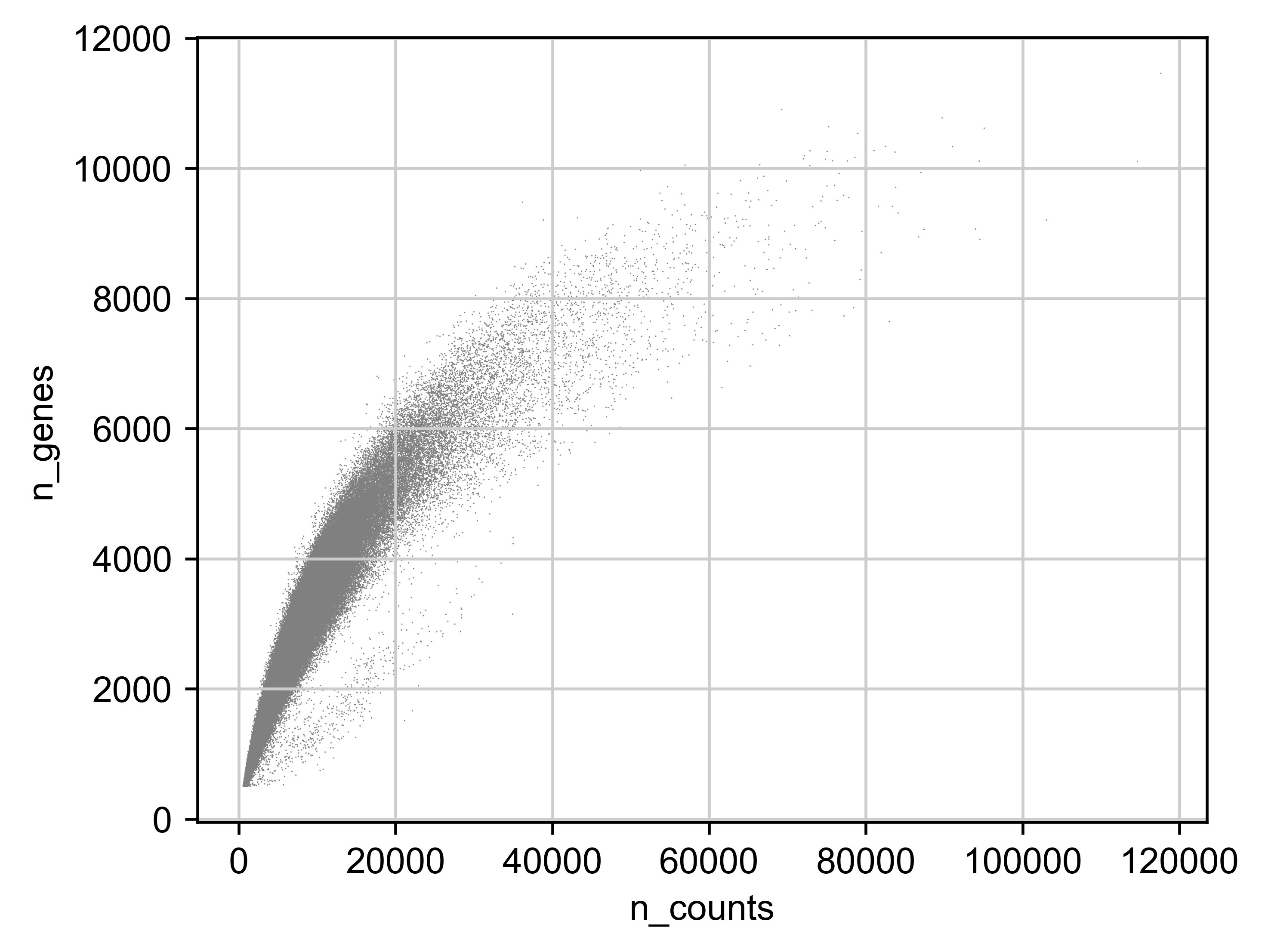

Supplement: Figure 7—source data 1. [file elife-83291-fig7-data1.zip › Figure7_C_and_D/ATLAS_ingest_ovaroids_2022-12-06/scatter_gene_vs_transcript_counts_ATLAS.png]

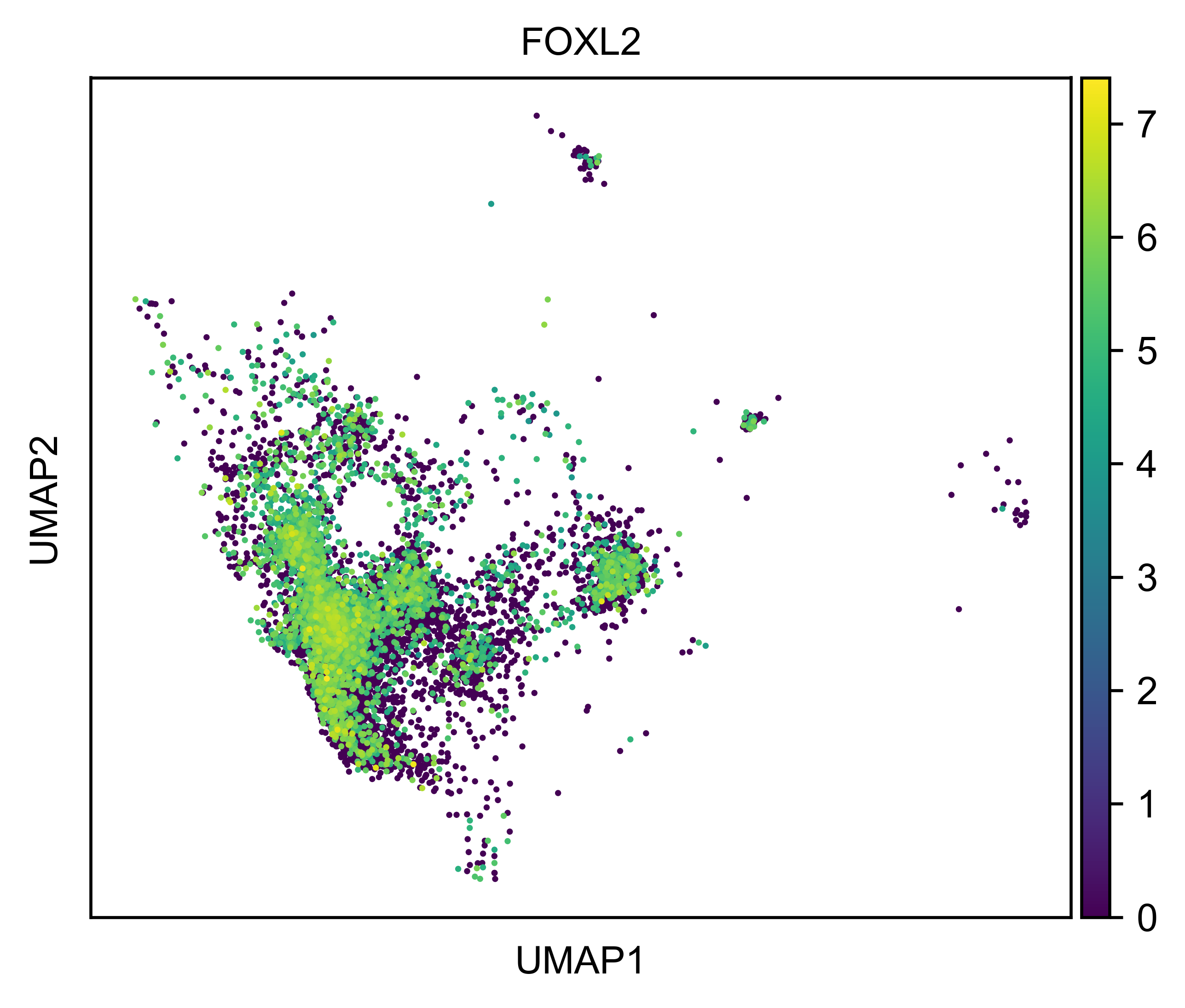

Supplement: Figure 7—source data 1. [file elife-83291-fig7-data1.zip › Figure7_C_and_D/ATLAS_ingest_ovaroids_2022-12-06/umap_FOXL2.png]

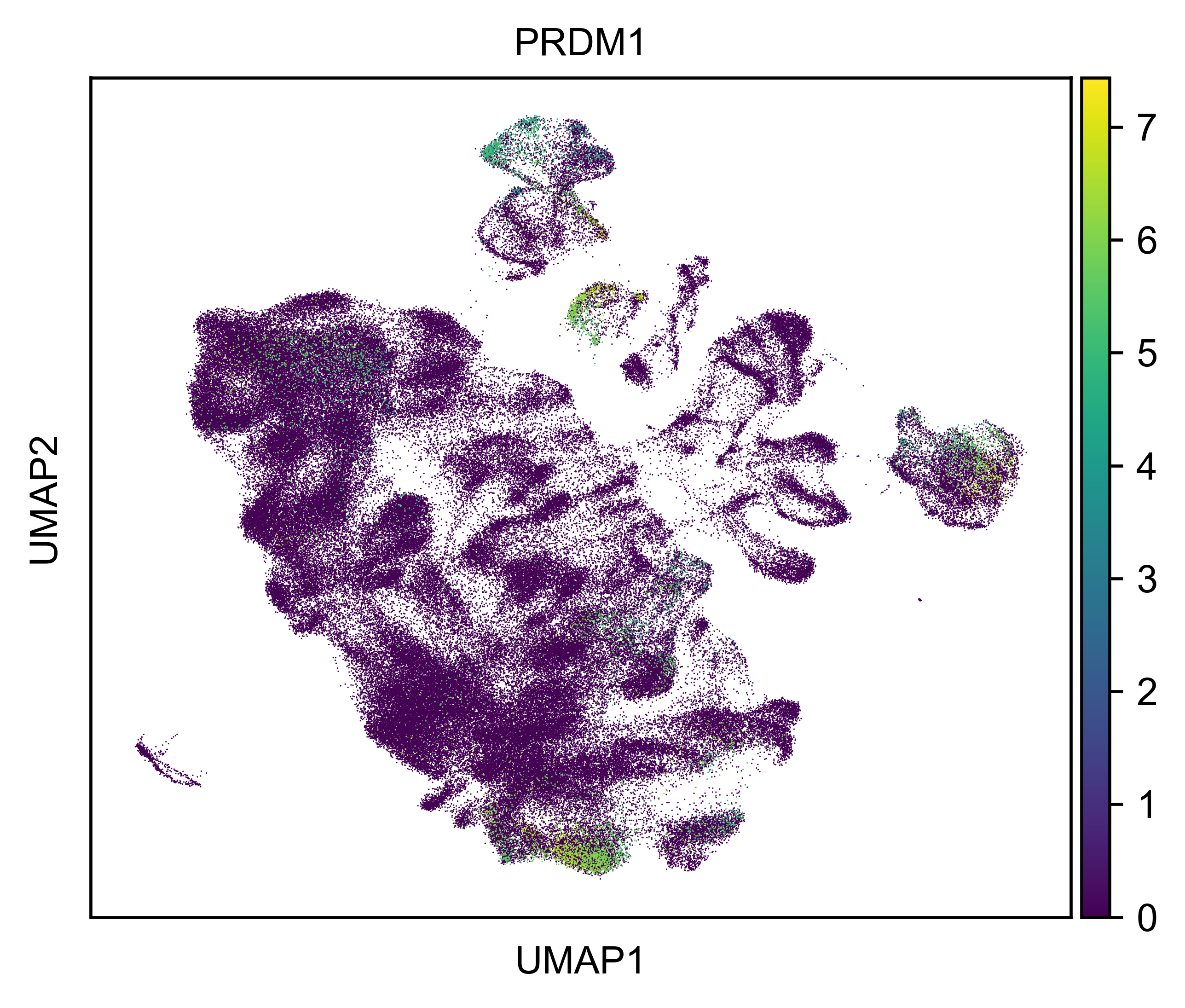

Supplement: Figure 7—source data 1. [file elife-83291-fig7-data1.zip › Figure7_C_and_D/ATLAS_ingest_ovaroids_2022-12-06/umap_atlas_PRDM1.png]

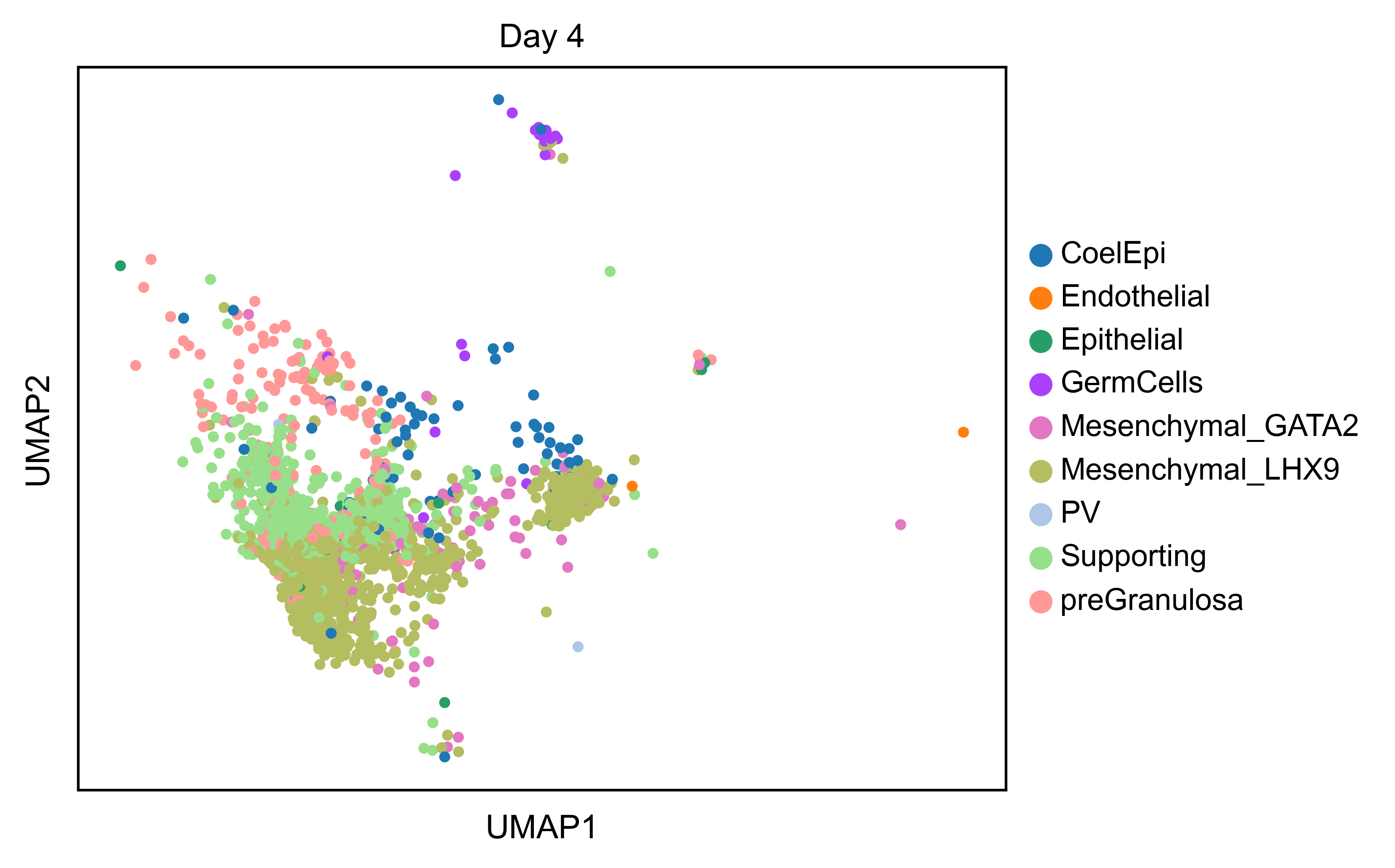

Supplement: Figure 7—source data 1. [file elife-83291-fig7-data1.zip › Figure7_C_and_D/ATLAS_ingest_ovaroids_2022-12-06/umap_scanpy_ingest_embedding_sample_Day4.png]

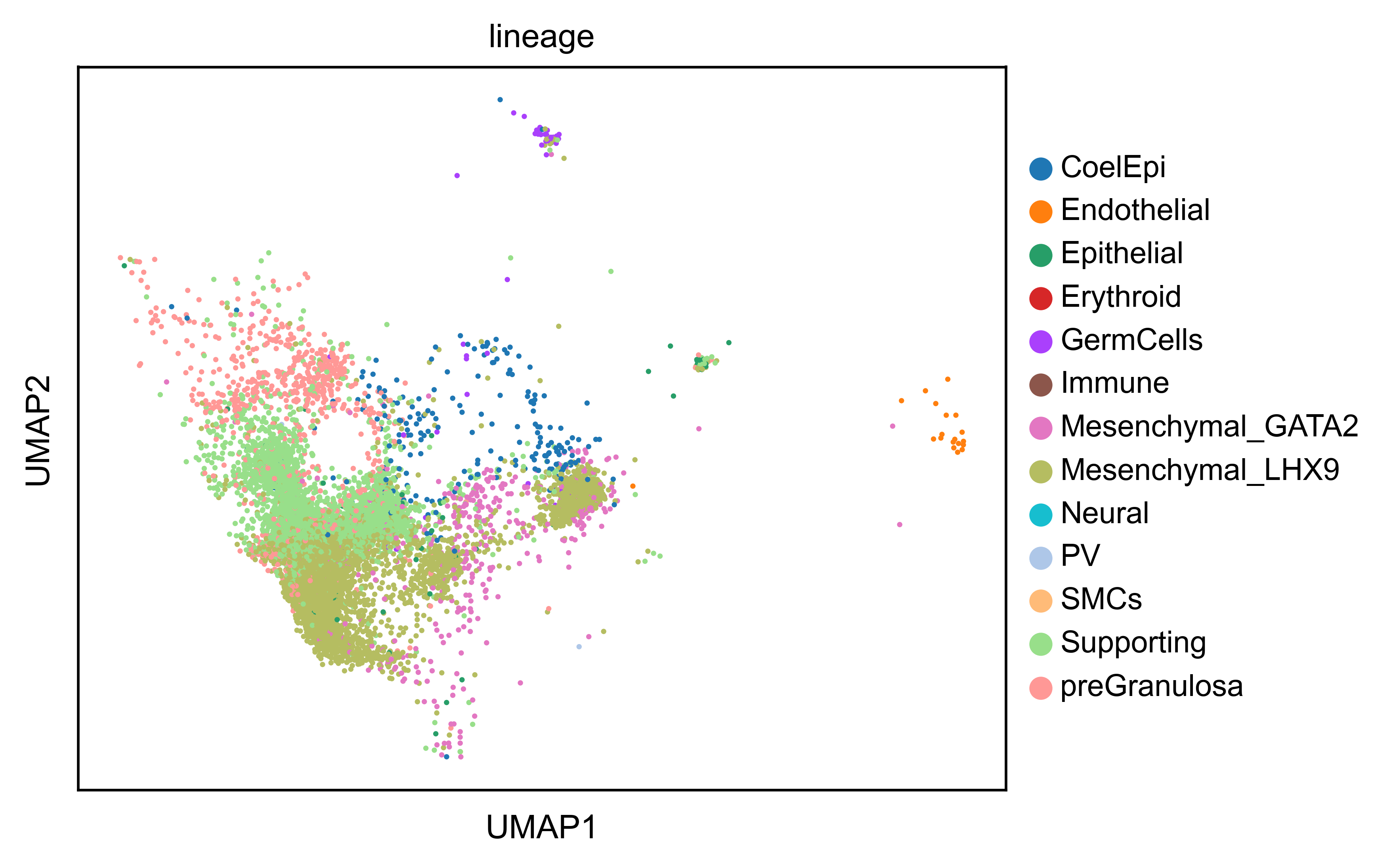

Supplement: Figure 7—source data 1. [file elife-83291-fig7-data1.zip › Figure7_C_and_D/ATLAS_ingest_ovaroids_2022-12-06/umap_scanpy_ingest_embedding.png]

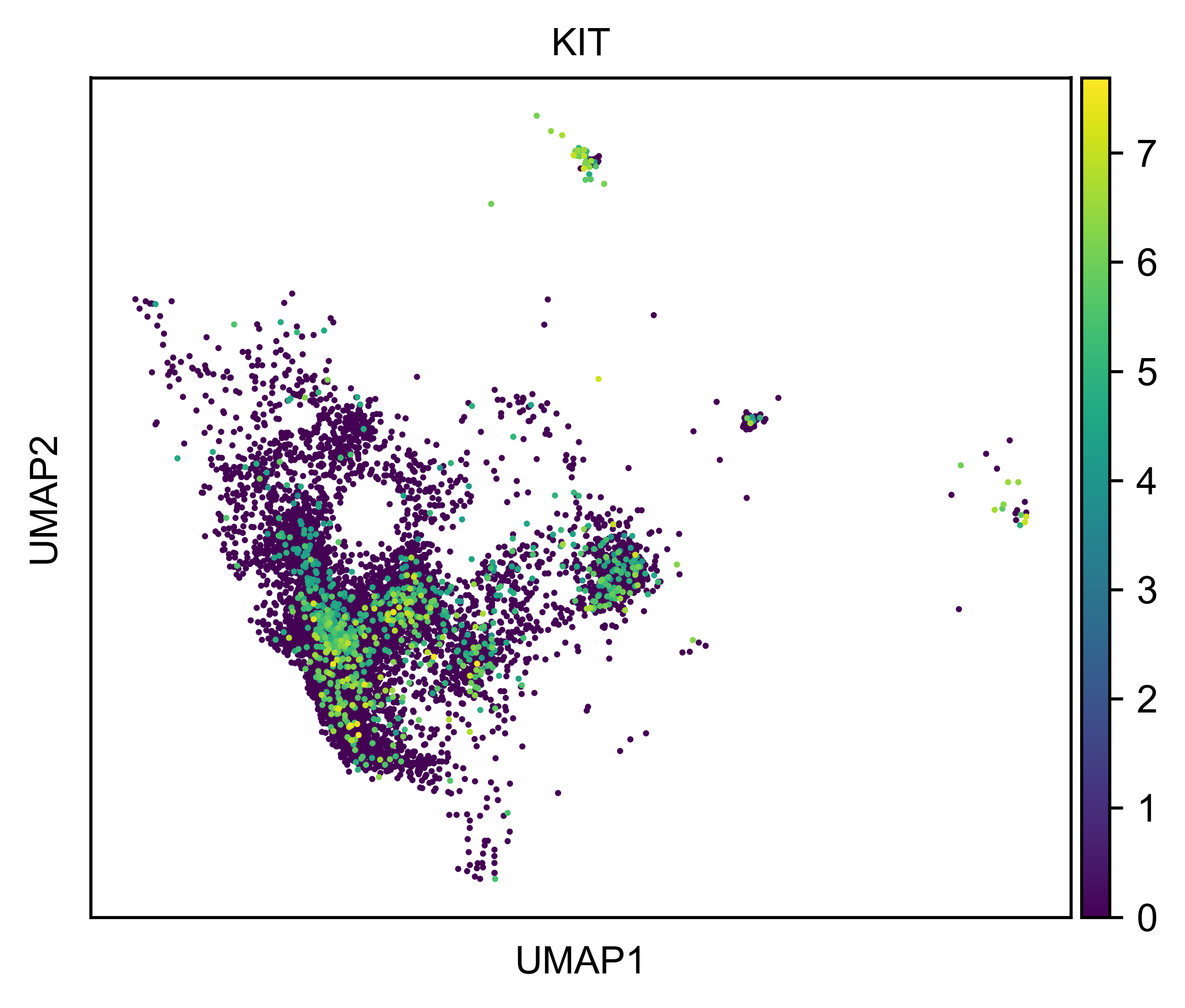

Supplement: Figure 7—source data 1. [file elife-83291-fig7-data1.zip › Figure7_C_and_D/ATLAS_ingest_ovaroids_2022-12-06/umap_KIT.png]

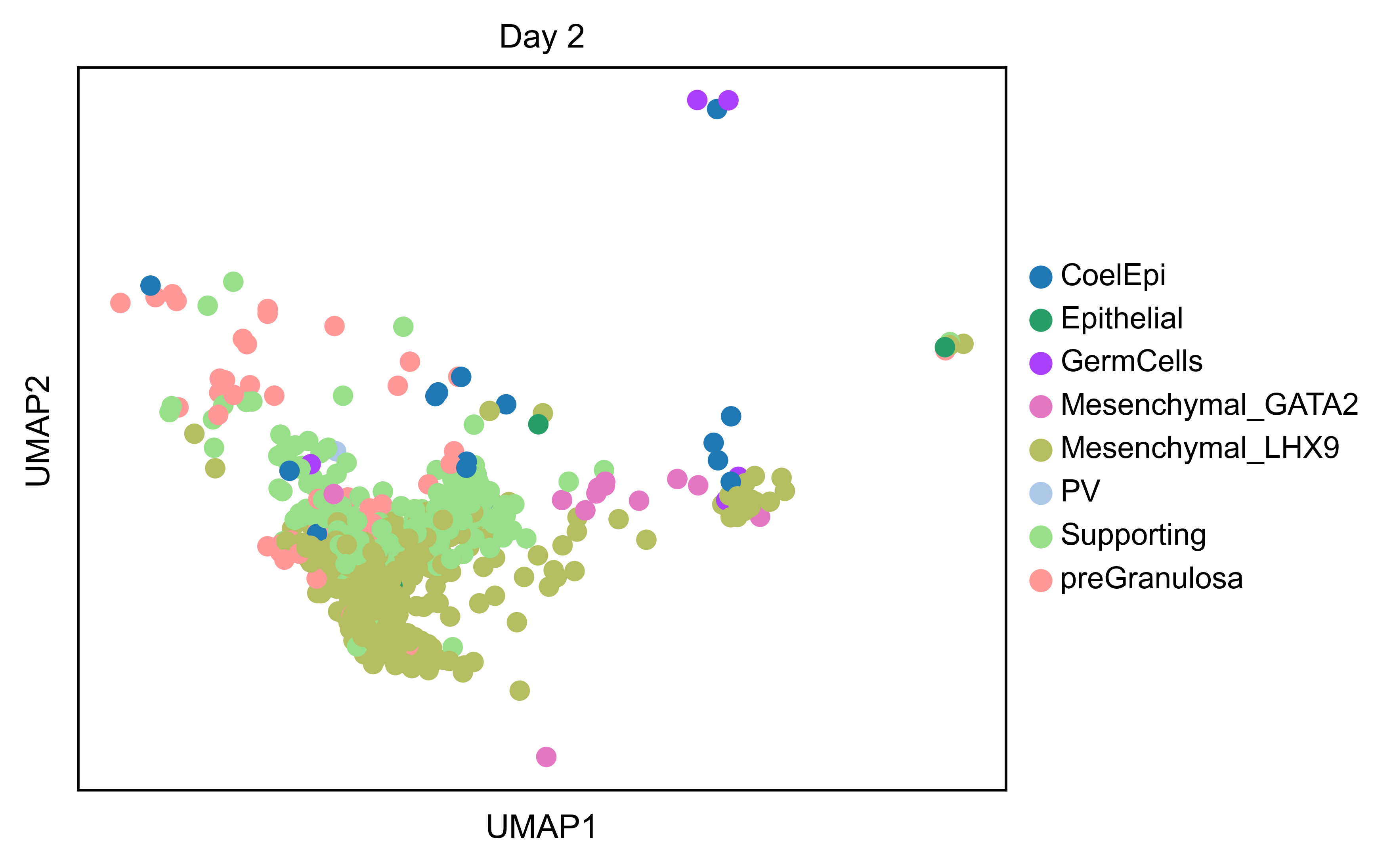

Supplement: Figure 7—source data 1. [file elife-83291-fig7-data1.zip › Figure7_C_and_D/ATLAS_ingest_ovaroids_2022-12-06/umap_scanpy_ingest_embedding_sample_Day2.png]

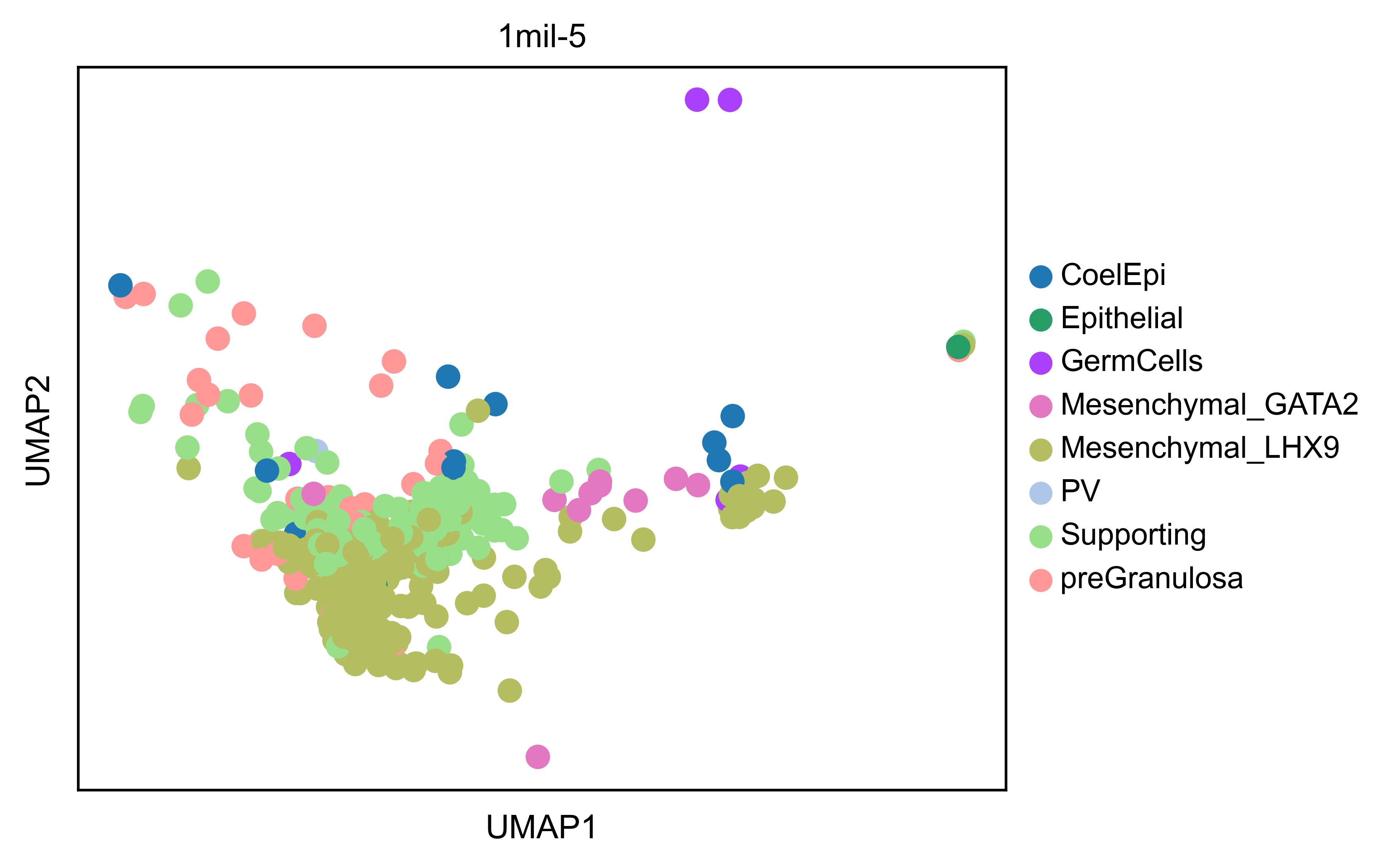

Supplement: Figure 7—source data 1. [file elife-83291-fig7-data1.zip › Figure7_C_and_D/ATLAS_ingest_ovaroids_2022-12-06/umap_scanpy_ingest_embedding_sample_1mil-5.png]

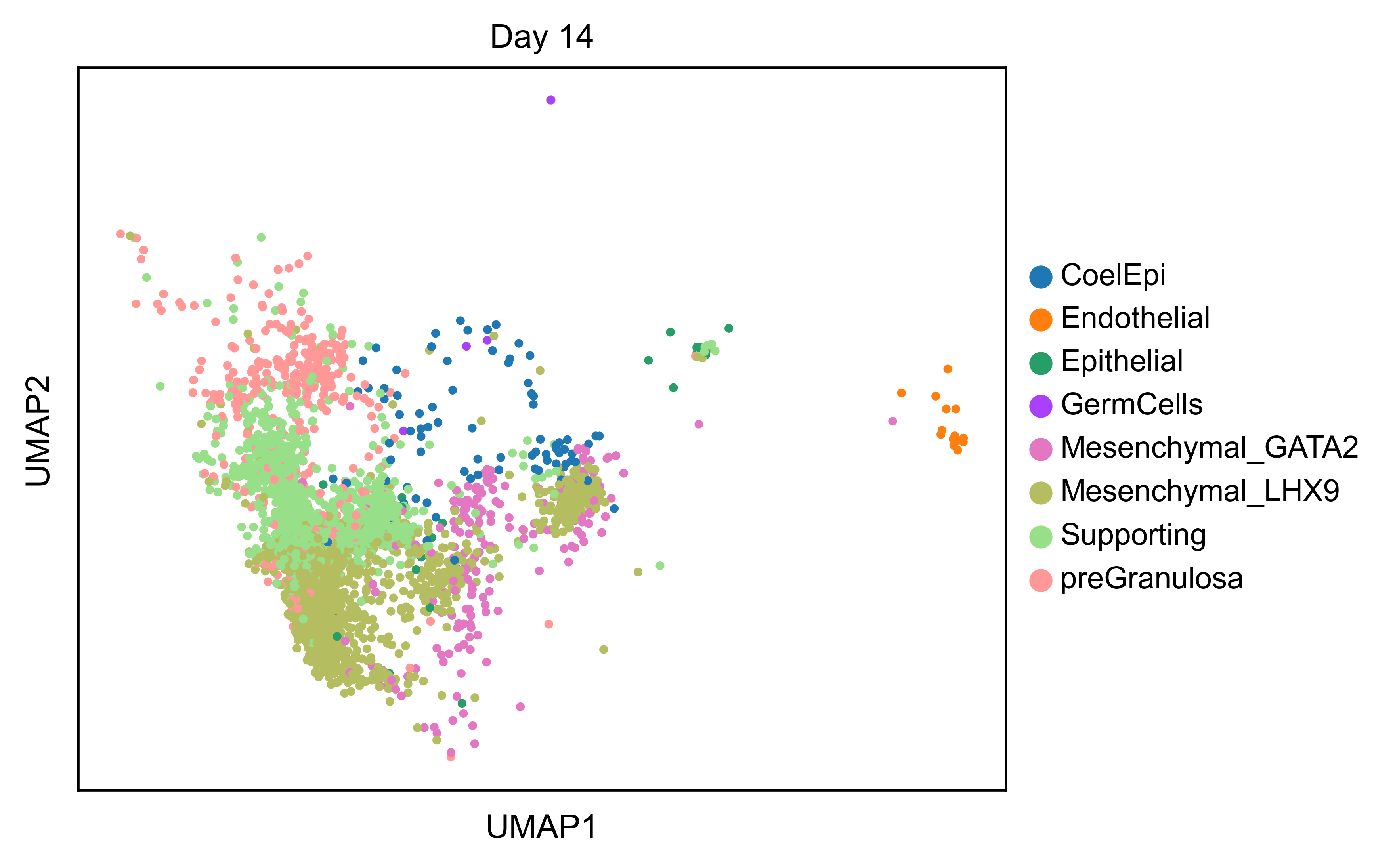

Supplement: Figure 7—source data 1. [file elife-83291-fig7-data1.zip › Figure7_C_and_D/ATLAS_ingest_ovaroids_2022-12-06/umap_scanpy_ingest_embedding_sample_Day14.png]

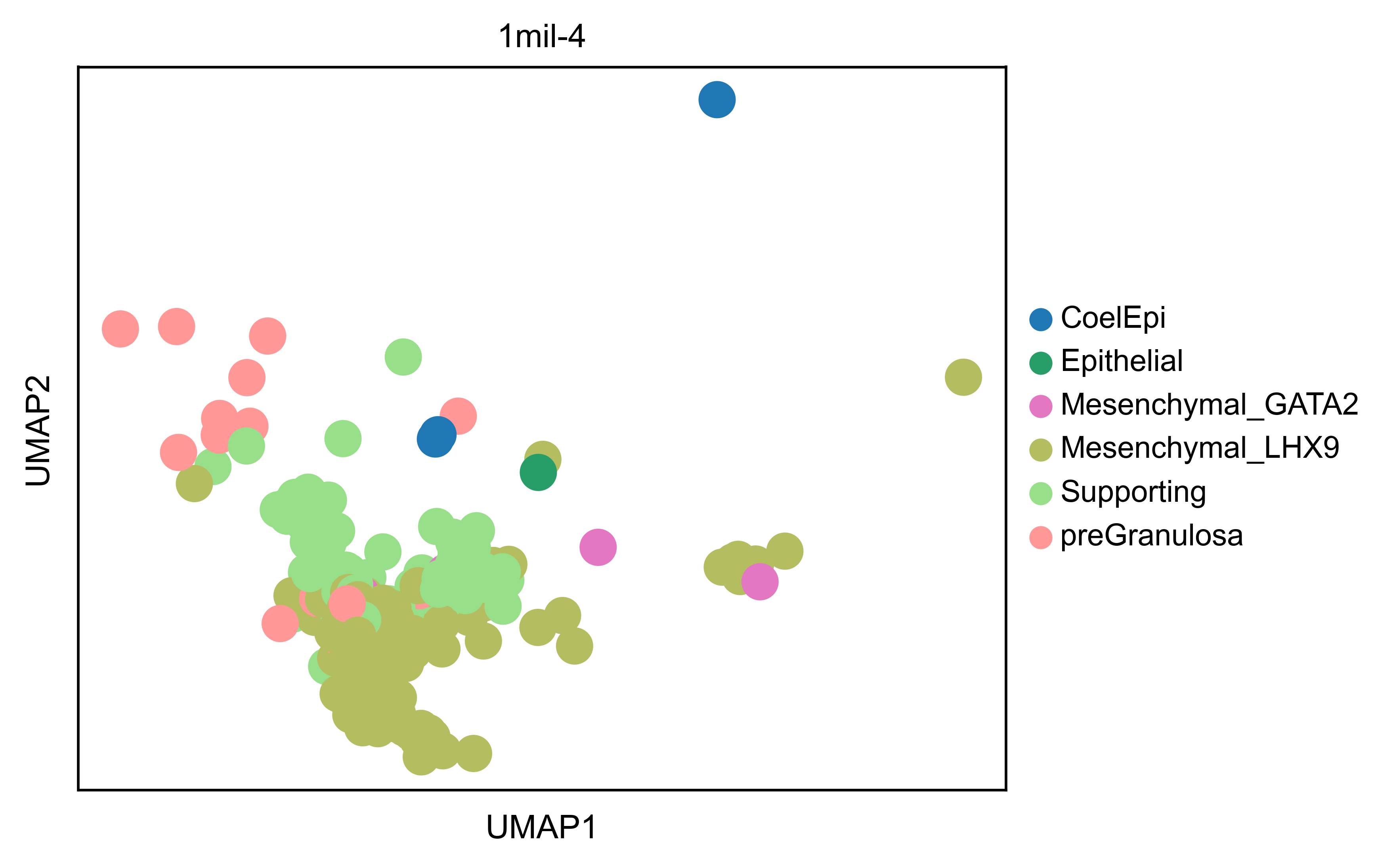

Supplement: Figure 7—source data 1. [file elife-83291-fig7-data1.zip › Figure7_C_and_D/ATLAS_ingest_ovaroids_2022-12-06/umap_scanpy_ingest_embedding_sample_1mil-4.png]

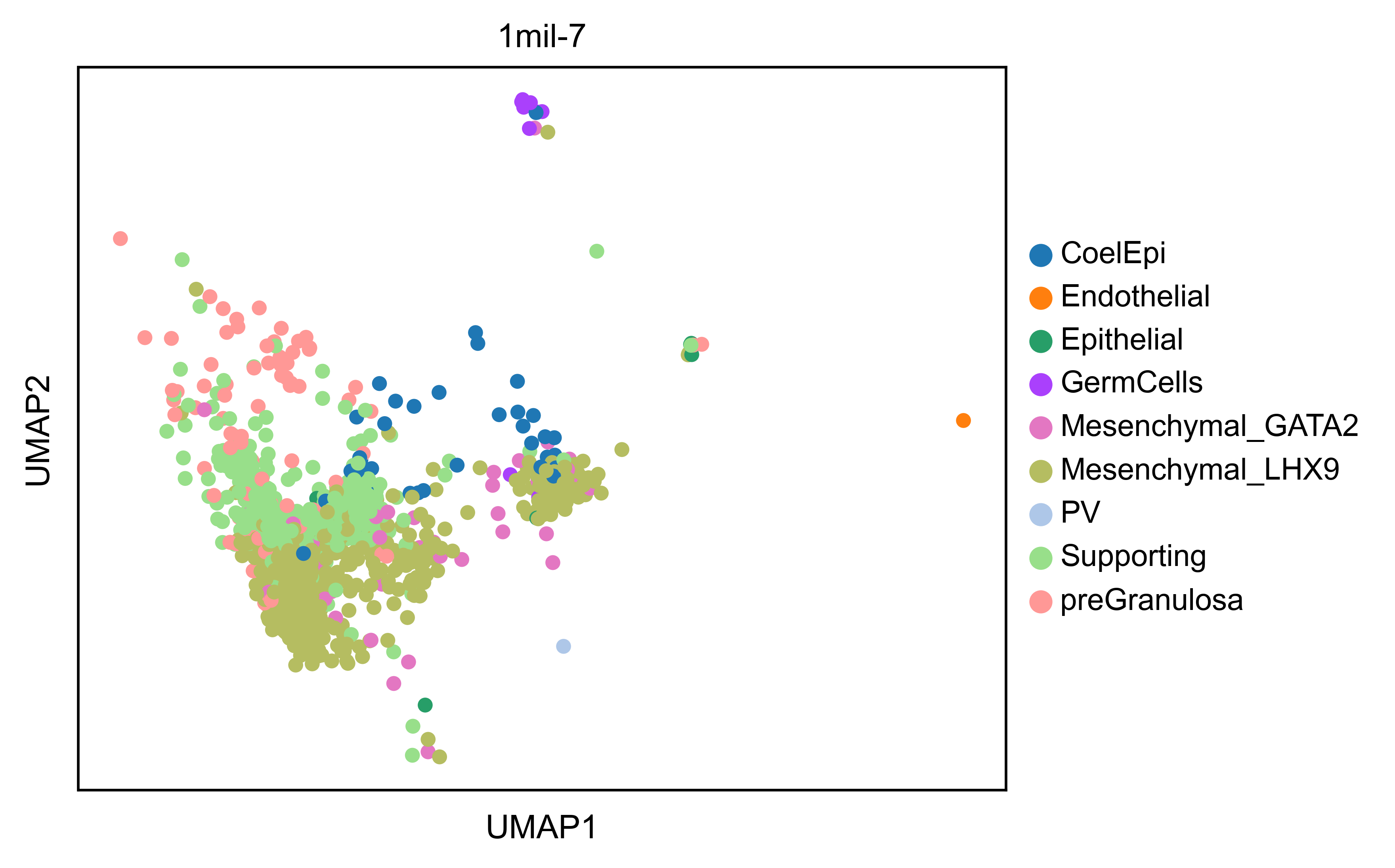

Supplement: Figure 7—source data 1. [file elife-83291-fig7-data1.zip › Figure7_C_and_D/ATLAS_ingest_ovaroids_2022-12-06/umap_scanpy_ingest_embedding_sample_1mil-7.png]

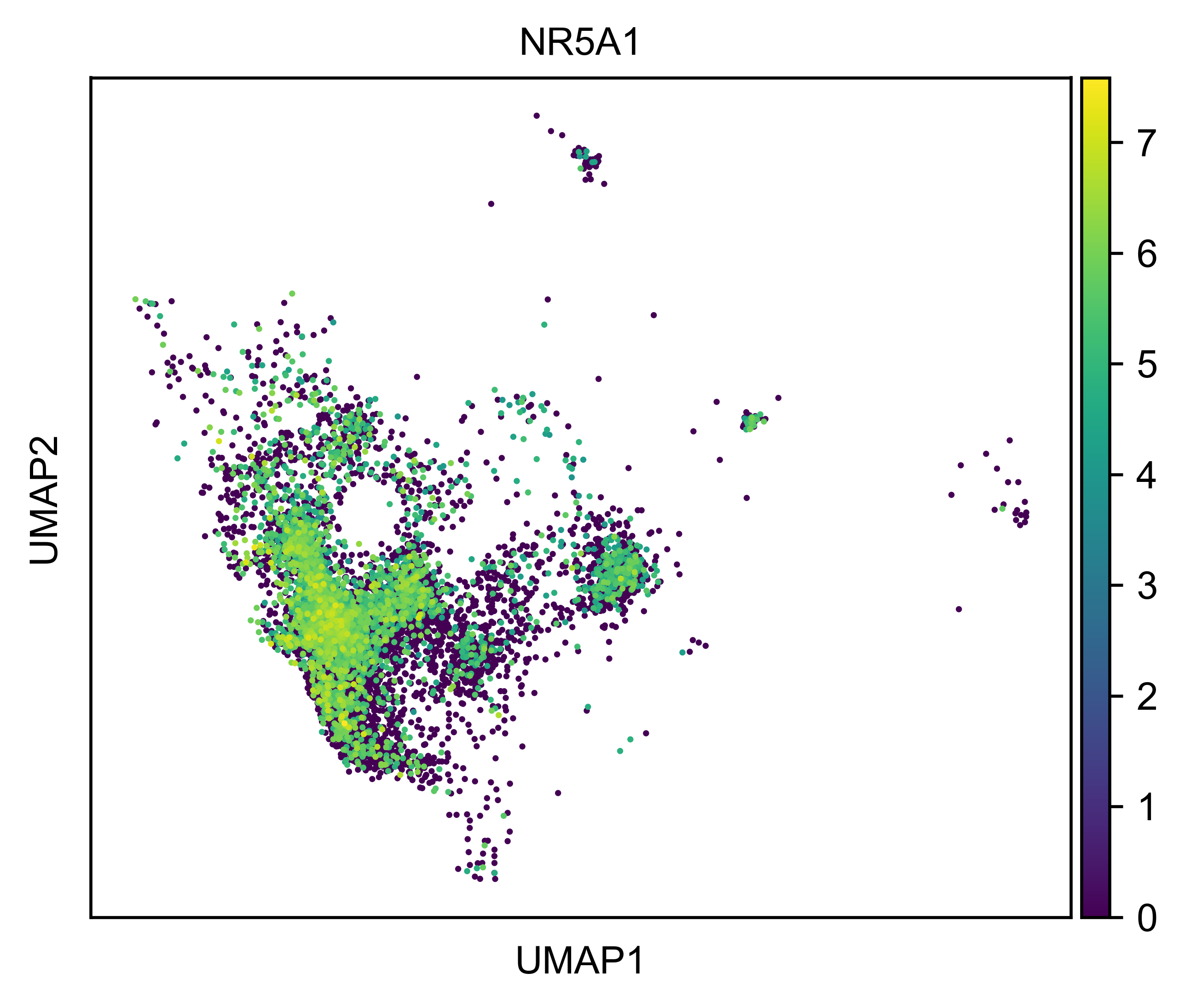

Supplement: Figure 7—source data 1. [file elife-83291-fig7-data1.zip › Figure7_C_and_D/ATLAS_ingest_ovaroids_2022-12-06/umap_NR5A1.png]

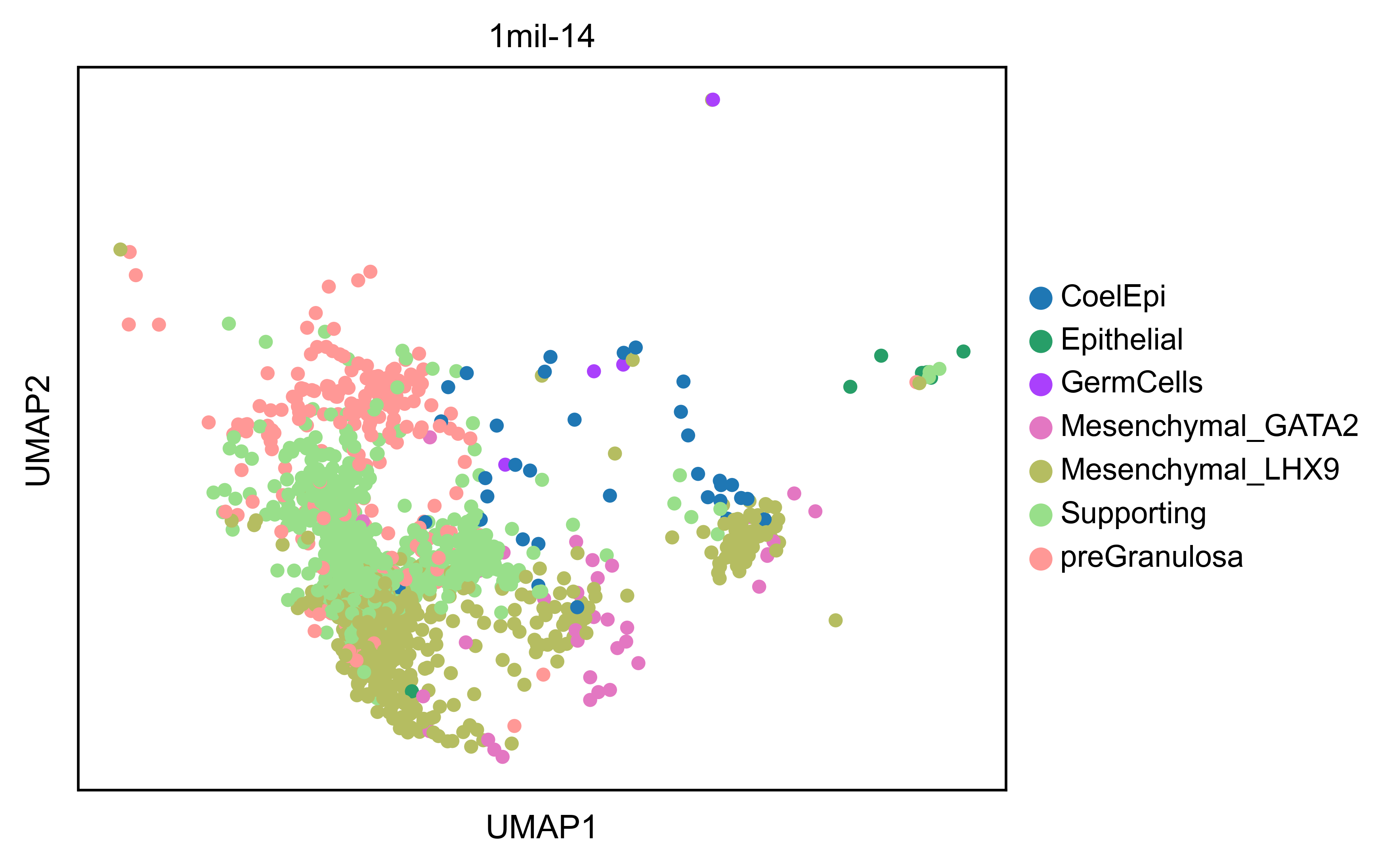

Supplement: Figure 7—source data 1. [file elife-83291-fig7-data1.zip › Figure7_C_and_D/ATLAS_ingest_ovaroids_2022-12-06/umap_scanpy_ingest_embedding_sample_1mil-14.png]

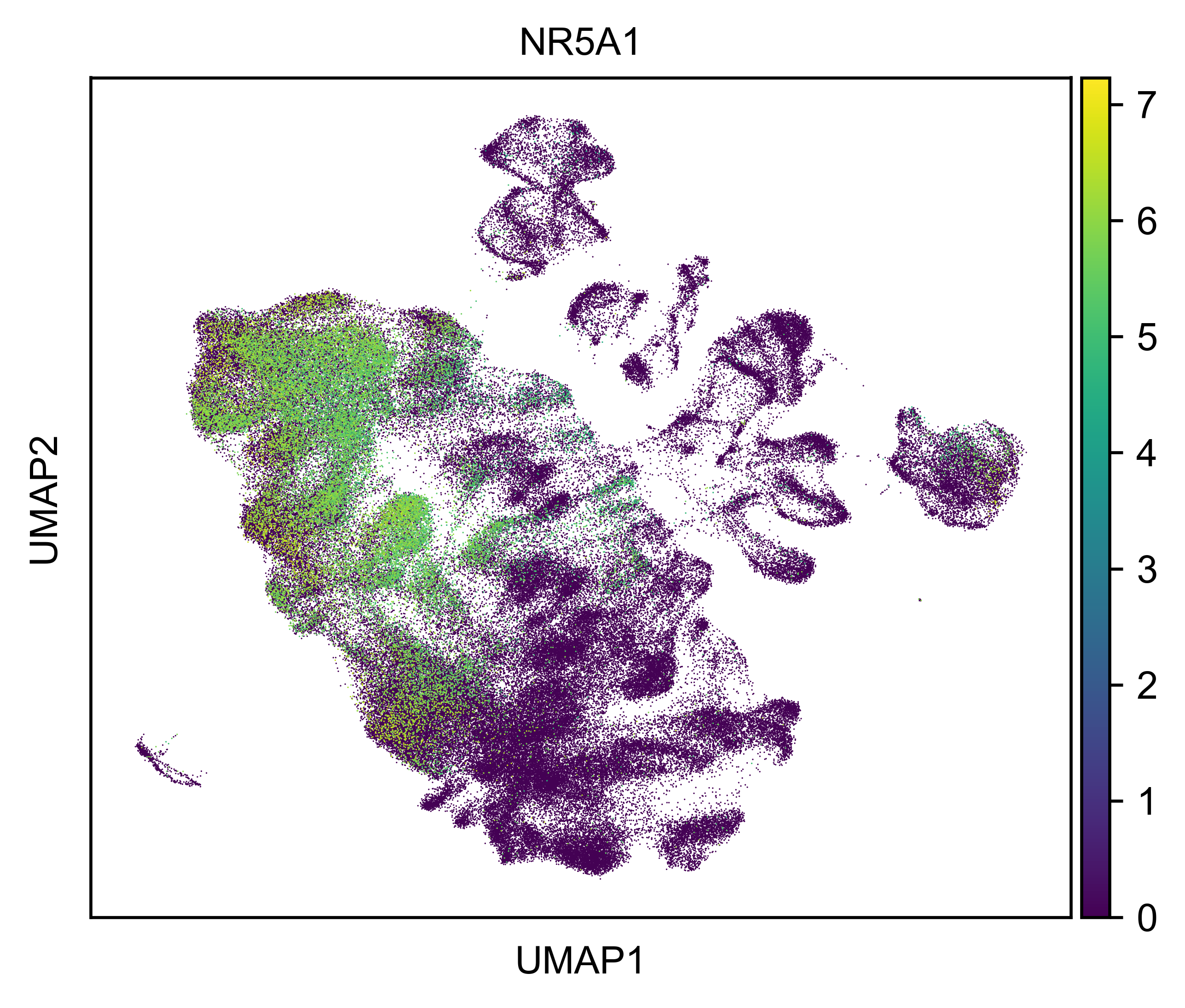

Supplement: Figure 7—source data 1. [file elife-83291-fig7-data1.zip › Figure7_C_and_D/ATLAS_ingest_ovaroids_2022-12-06/umap_atlas_NR5A1.png]

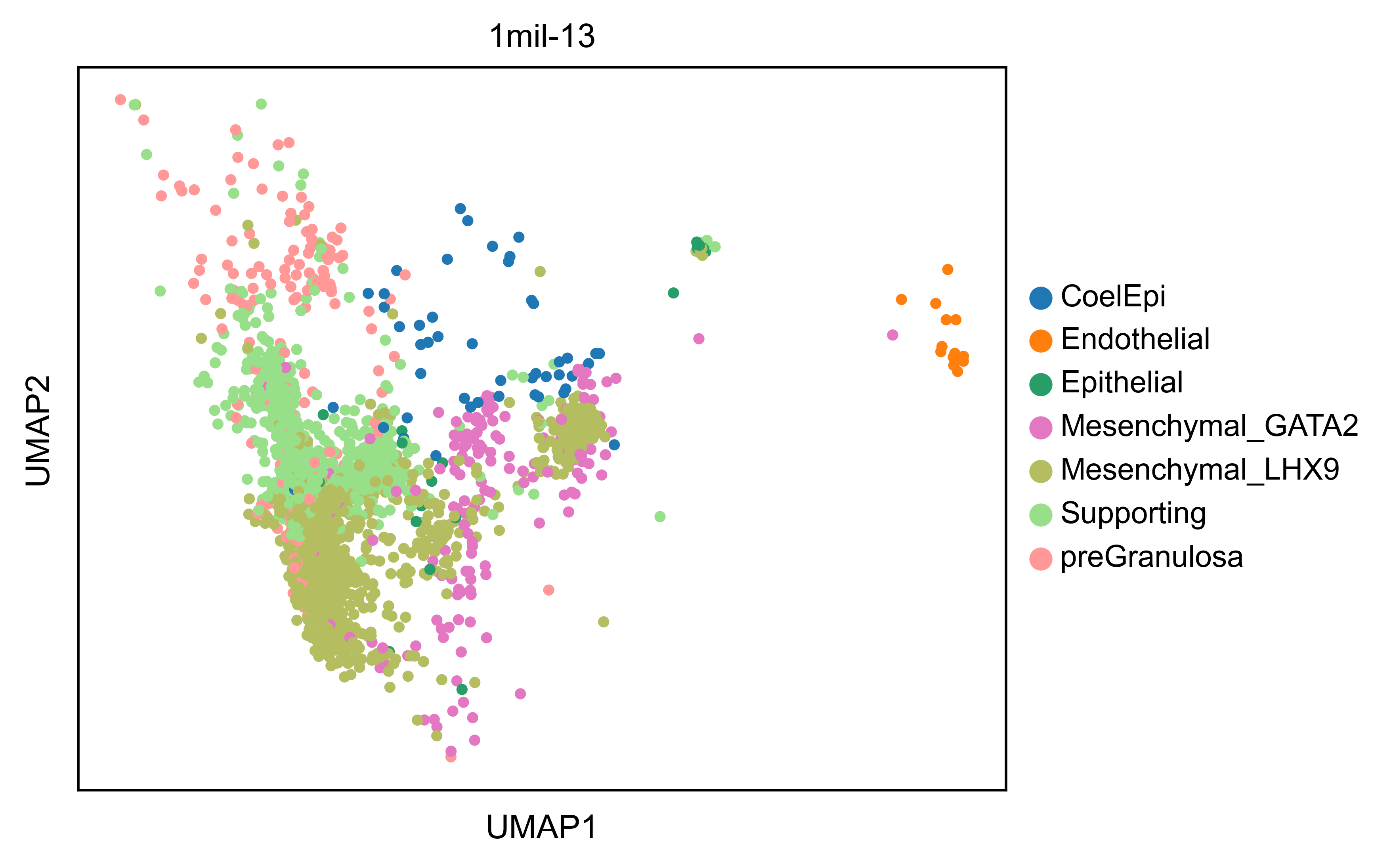

Supplement: Figure 7—source data 1. [file elife-83291-fig7-data1.zip › Figure7_C_and_D/ATLAS_ingest_ovaroids_2022-12-06/umap_scanpy_ingest_embedding_sample_1mil-13.png]

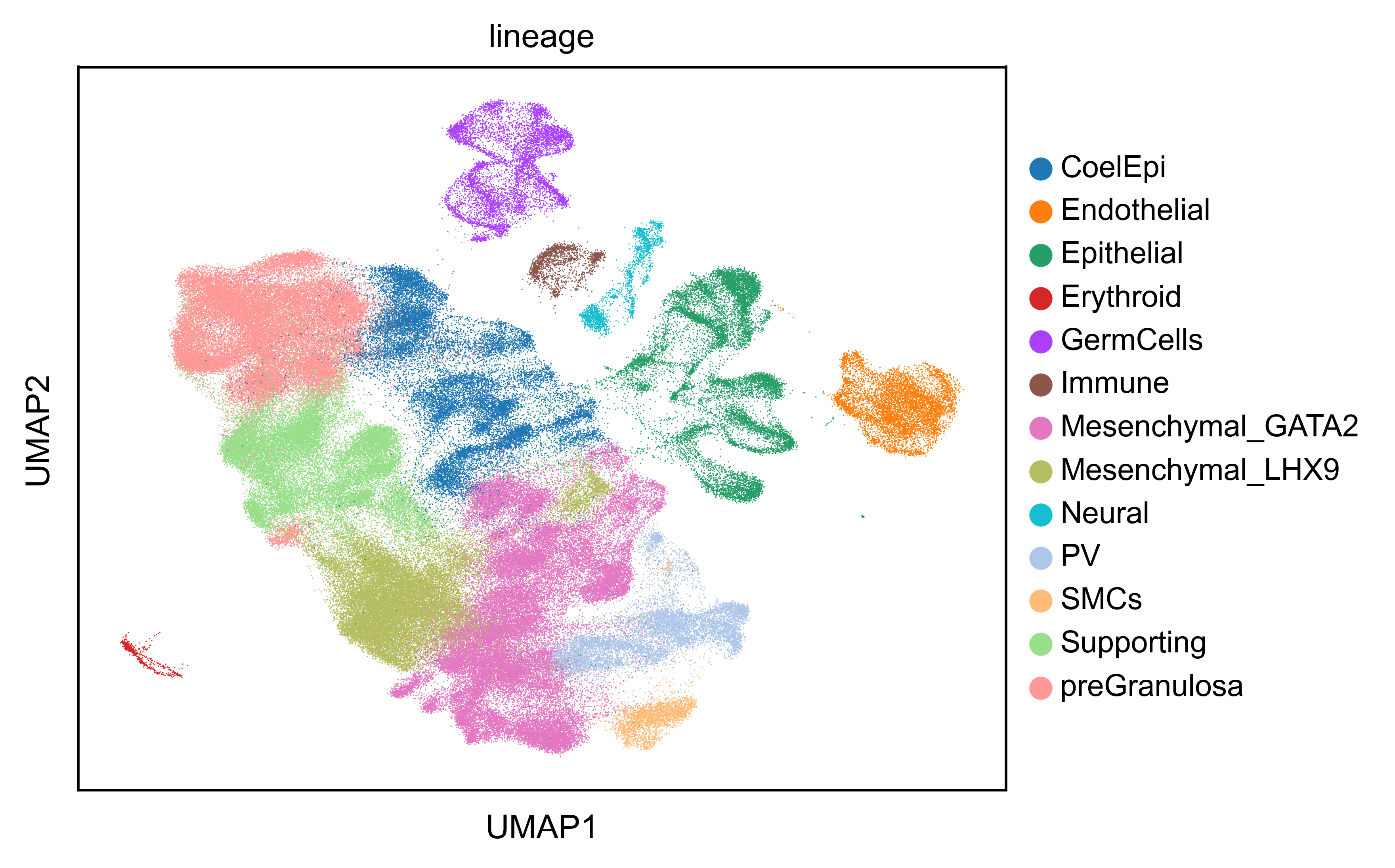

Supplement: Figure 7—source data 1. [file elife-83291-fig7-data1.zip › Figure7_C_and_D/ATLAS_ingest_ovaroids_2022-12-06/umap_scanpy_ingest_atlas.png]

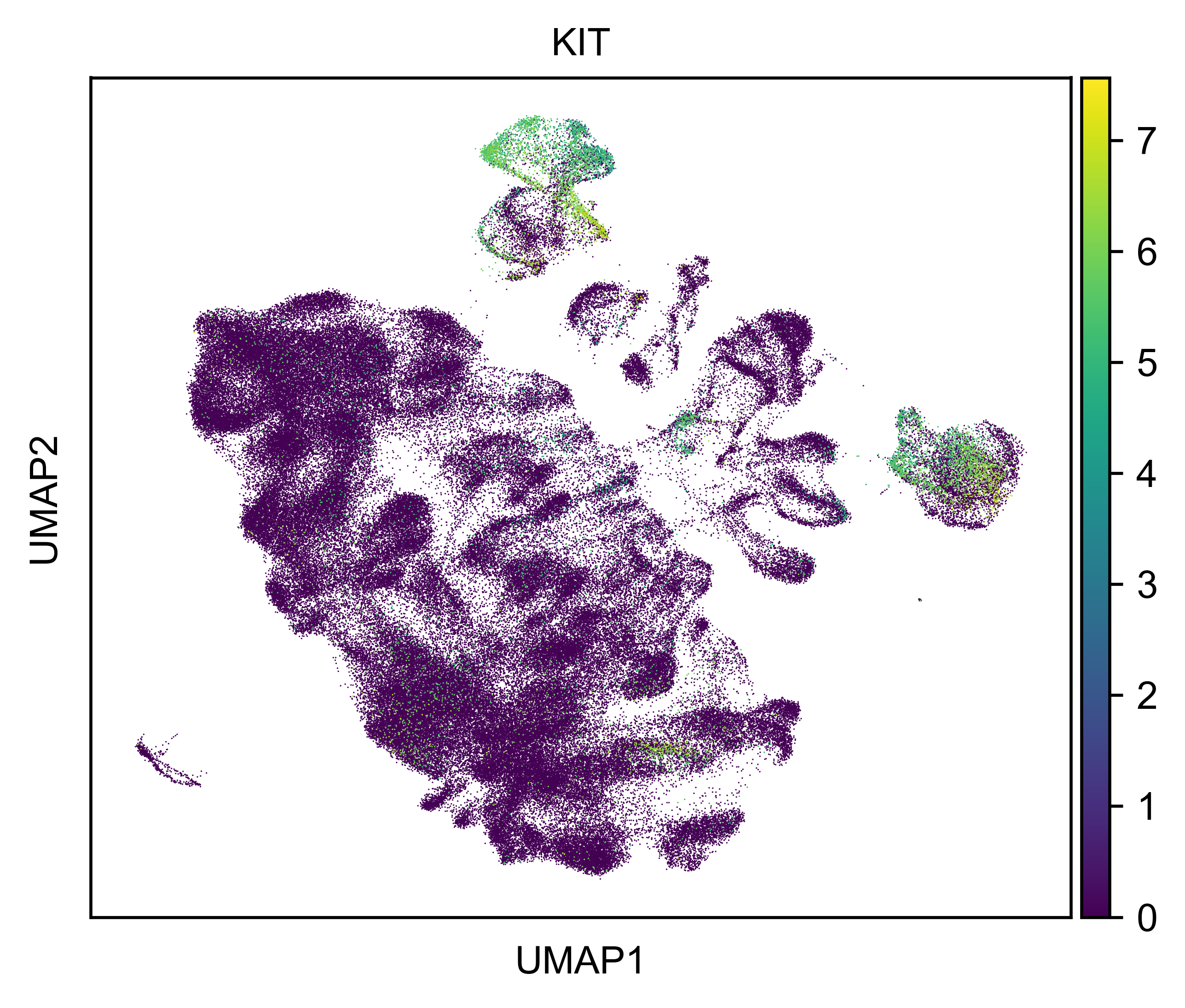

Supplement: Figure 7—source data 1. [file elife-83291-fig7-data1.zip › Figure7_C_and_D/ATLAS_ingest_ovaroids_2022-12-06/umap_atlas_KIT.png]

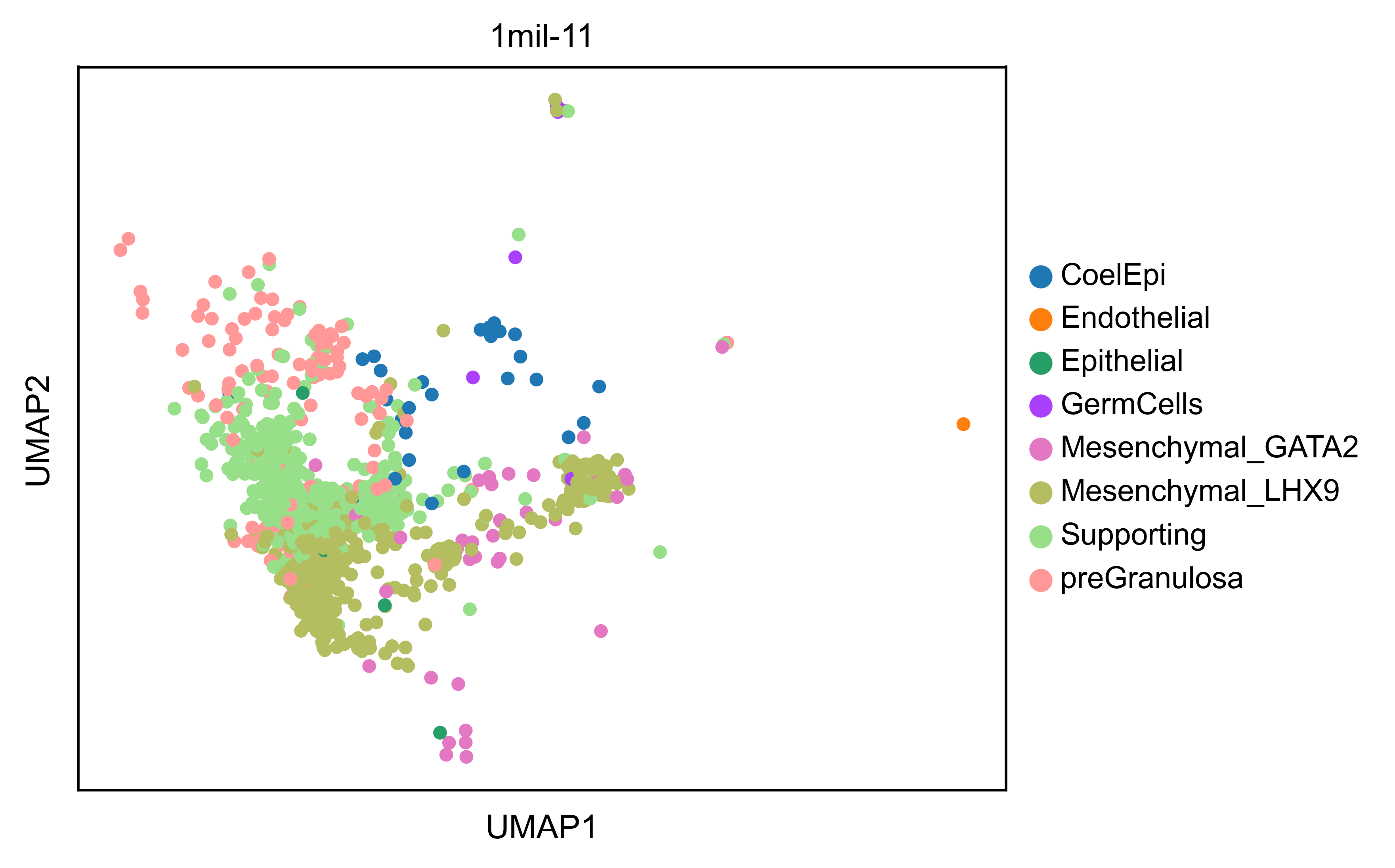

Supplement: Figure 7—source data 1. [file elife-83291-fig7-data1.zip › Figure7_C_and_D/ATLAS_ingest_ovaroids_2022-12-06/umap_scanpy_ingest_embedding_sample_1mil-11.png]

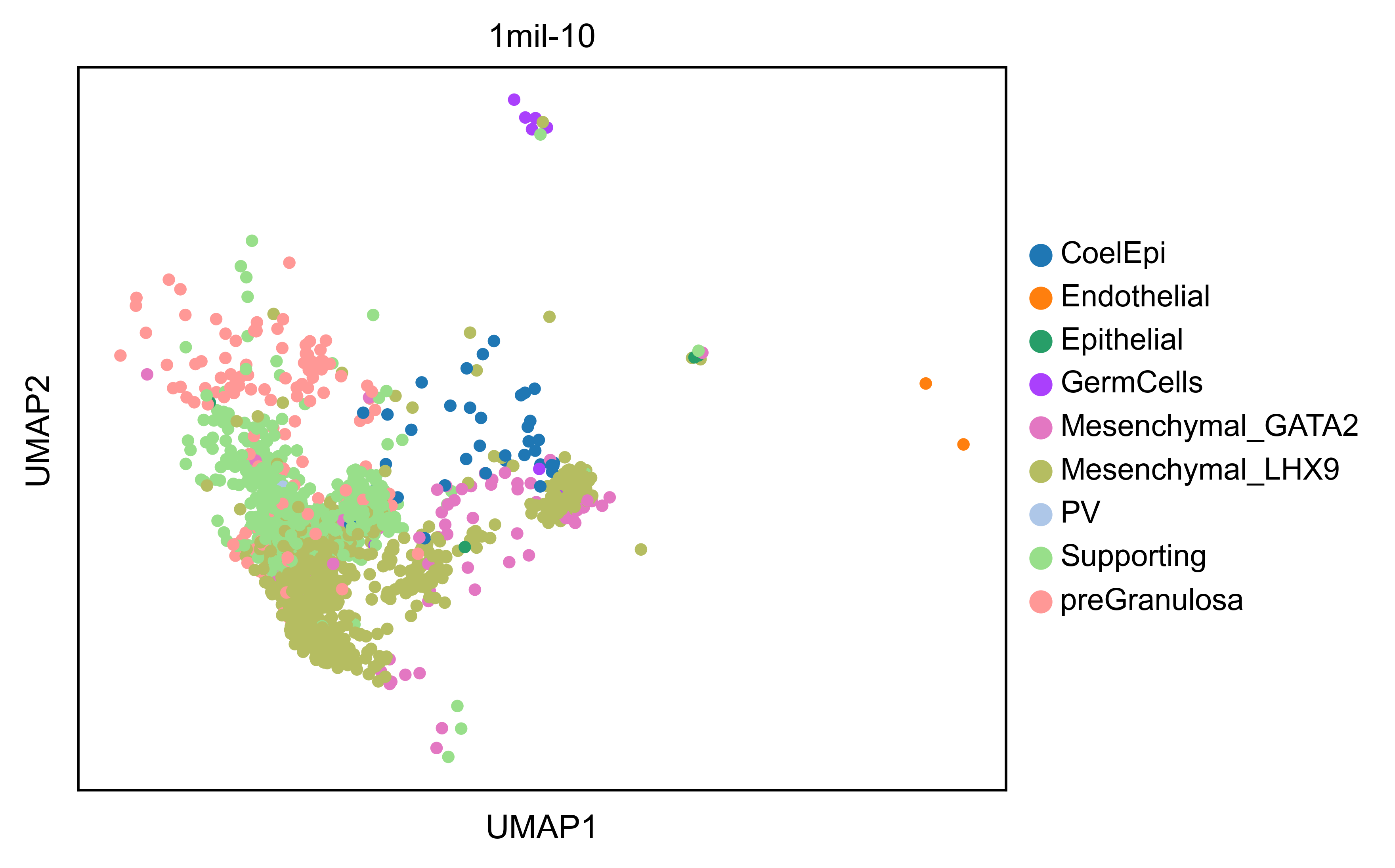

Supplement: Figure 7—source data 1. [file elife-83291-fig7-data1.zip › Figure7_C_and_D/ATLAS_ingest_ovaroids_2022-12-06/umap_scanpy_ingest_embedding_sample_1mil-10.png]

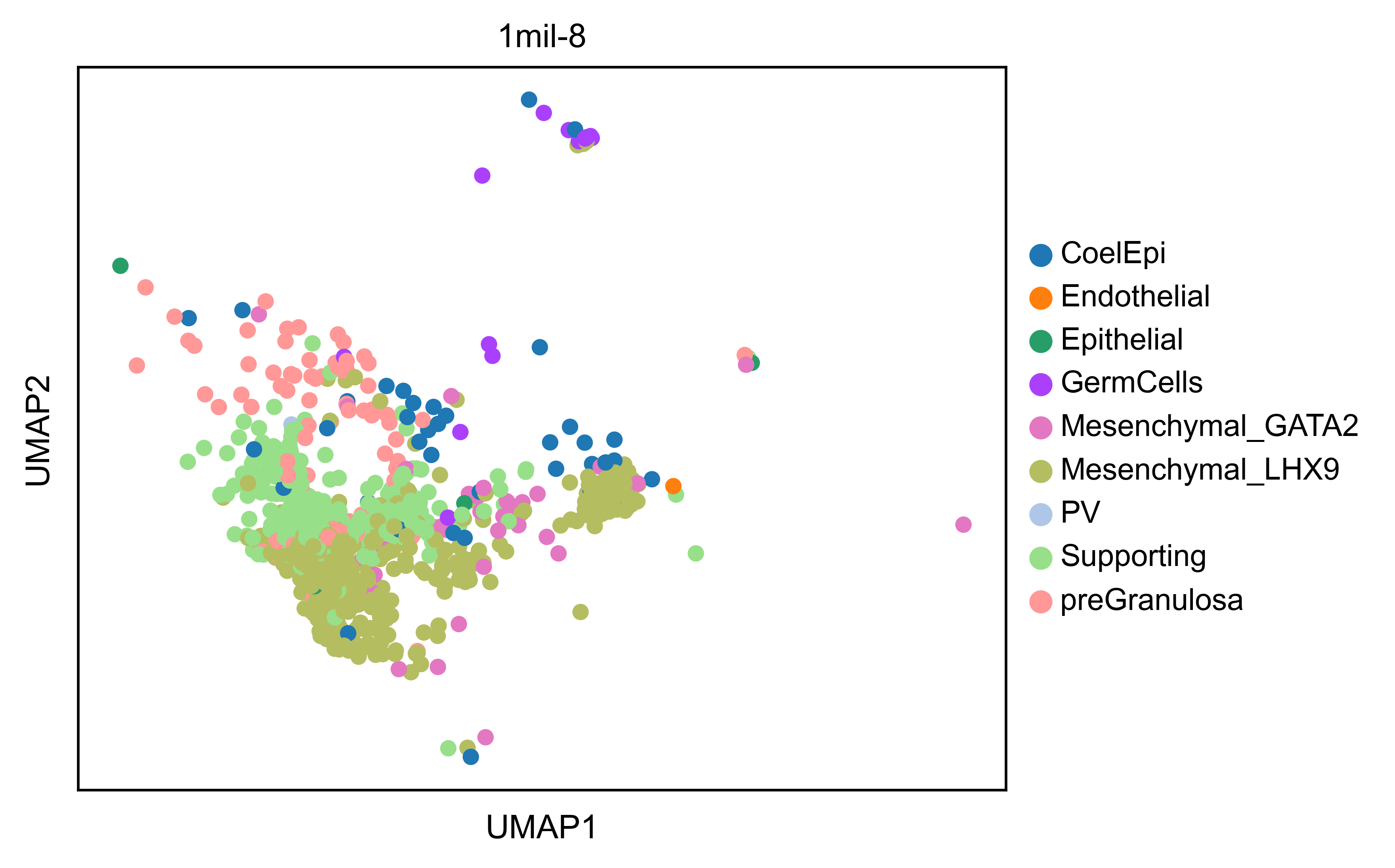

Supplement: Figure 7—source data 1. [file elife-83291-fig7-data1.zip › Figure7_C_and_D/ATLAS_ingest_ovaroids_2022-12-06/umap_scanpy_ingest_embedding_sample_1mil-8.png]

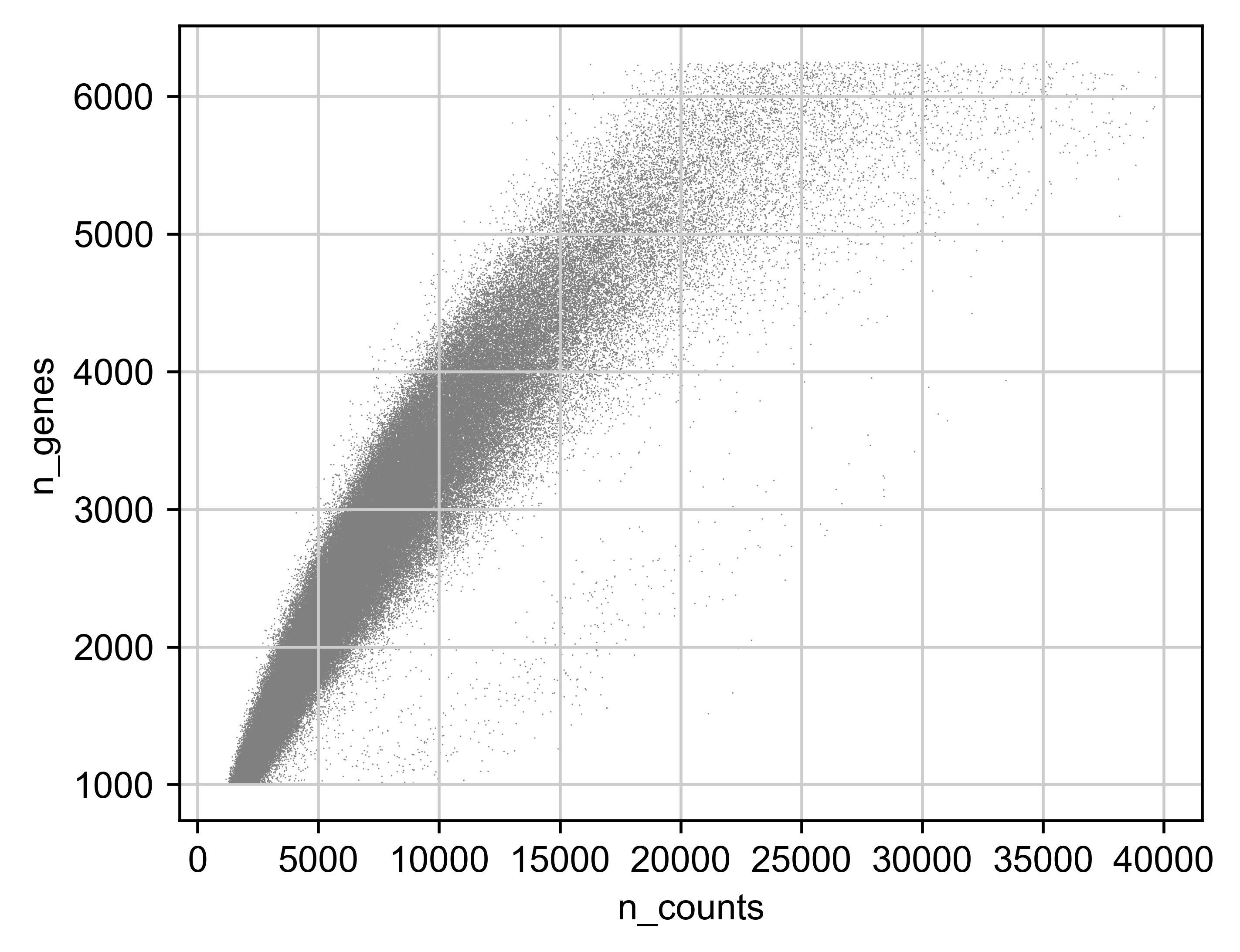

Supplement: Figure 7—source data 1. [file elife-83291-fig7-data1.zip › Figure7_C_and_D/ATLAS_ingest_ovaroids_2022-12-06/scatter_gene_vs_transcript_counts_ATLAS_filtered.png]

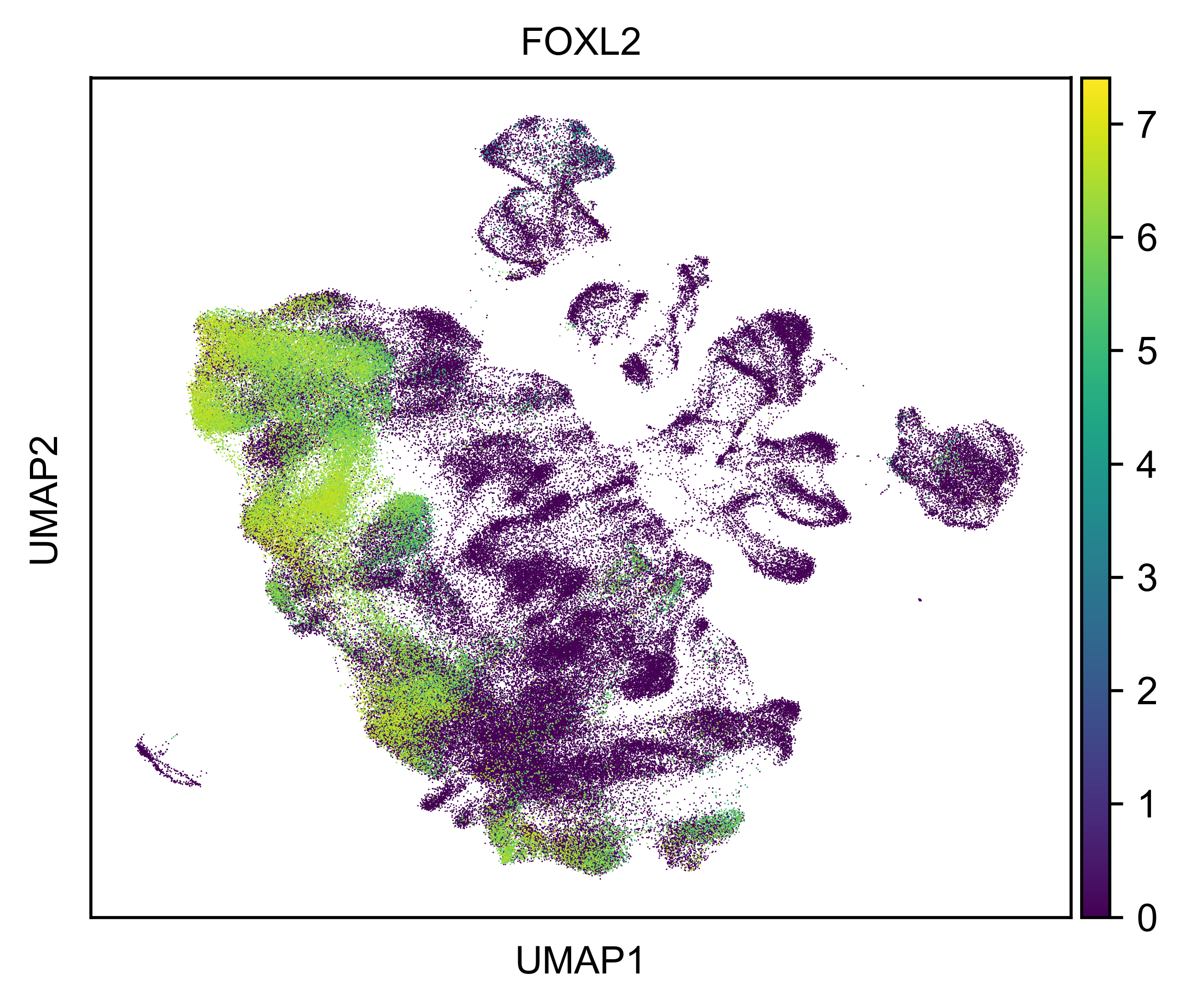

Supplement: Figure 7—source data 1. [file elife-83291-fig7-data1.zip › Figure7_C_and_D/ATLAS_ingest_ovaroids_2022-12-06/umap_atlas_FOXL2.png]

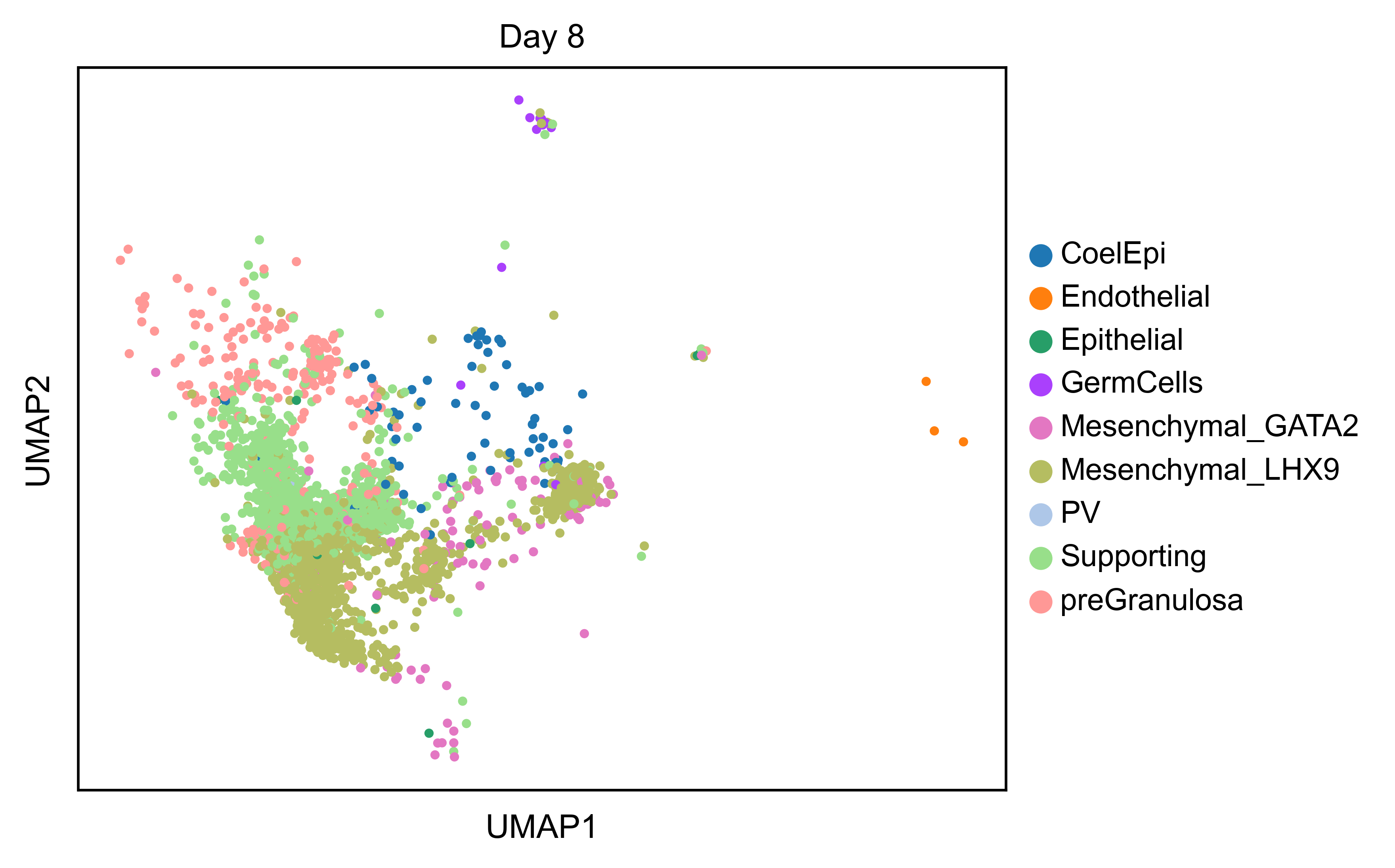

Supplement: Figure 7—source data 1. [file elife-83291-fig7-data1.zip › Figure7_C_and_D/ATLAS_ingest_ovaroids_2022-12-06/umap_scanpy_ingest_embedding_sample_Day8.png]

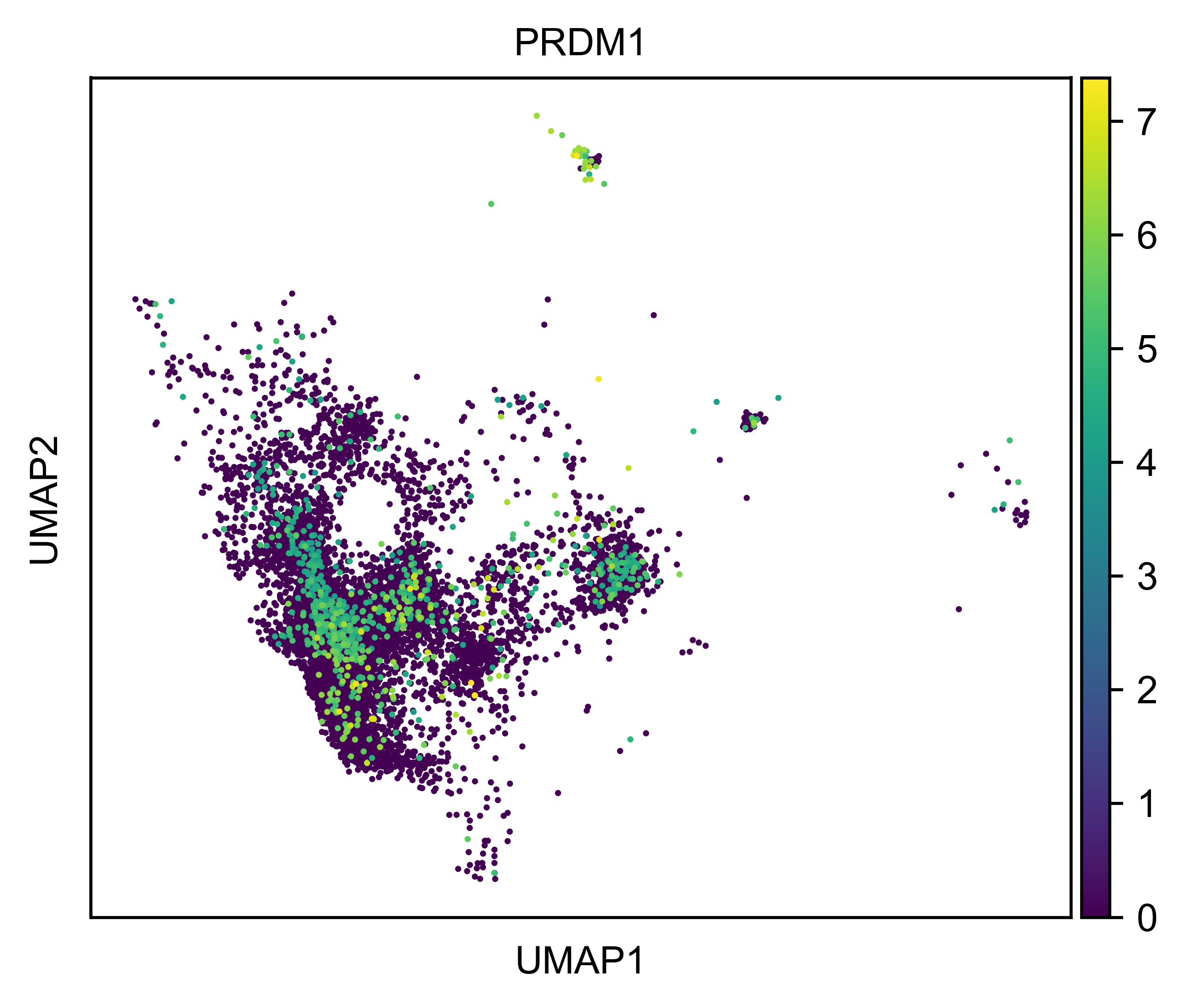

Supplement: Figure 7—source data 1. [file elife-83291-fig7-data1.zip › Figure7_C_and_D/ATLAS_ingest_ovaroids_2022-12-06/umap_PRDM1.png]
